# A genome-wide CRISPR knockout screen identified host genes essential for Brucella invasion and intracellular survival

**DOI:** 10.1080/22221751.2026.2713322

**Published:** 2026-08-19

**Authors:** Xinhui Wang, Sujun Wu, Yige Ding, Jiabo Ding, Peng Li, Zhengxing Lian

**Affiliations:** a State Key Laboratory of Animal Biotech Breeding, Beijing Key Laboratory for Animal Genetic Improvement, National Engineering Laboratory for Animal Breeding, Key Laboratory of Animal Genetics and Breeding of the Ministry of Agriculture, College of Animal Science and Technology, China Agricultural University, Beijing, People’s Republic of China; b Key Laboratory of Animal Biosafety Risk Prevention and Control (North) of MARA, Institute of Animal Science, Chinese Academy of Agricultural Sciences, Beijing, People’s Republic of China; c State Key Laboratory of Chemistry for NBC Hazards Protection, Beijing, People’s Republic of China

**Keywords:** *Brucella*, genome-wide screen, host genes, intracellular survival, chronic infections

## Abstract

For *Brucella spp*., the ability to invade and survive within host macrophages is essential for causing chronic infections in their mammalian hosts. In this study, a genome-wide CRISPR knockout screen was performed for the first time in human THP-1 macrophages to identify host genes mediating resistance to *Brucella* invasion and intracellular survival. Results showed that the screening identified 35 candidate genes, 11 of which were selected to generate monoclonal knockout cell lines for functional validation. This study demonstrated that knockout of *WDR4*, *ZNF532*, or *MTHFD1* significantly restricted *Brucella* invasion and early intracellular survival. In addition, *TRAPPC2* knockout restricted *Brucella* invasion and, crucially, its intracellular survival throughout infection, exerting the most potent antibacterial effect. Mechanistically, *TRAPPC2* deficiency suppresses *Brucella* infection by inhibiting autophagosome formation in macrophages. Furthermore, *TRAPPC2* knockout decreases macrophage apoptosis and improves host cell viability following *Brucella* infection. These results provide therapeutic targets for combating *Brucella* infection and offer novel insights into the molecular mechanisms associated with *Brucella*-induced chronic infections.

## Introduction

Brucellosis is a severe zoonotic disease caused by Gram-negative bacteria of the genus *Brucella*, currently prevalent in at least 97 countries worldwide [1,2]. Human brucellosis cases primarily originate from infected livestock (e.g. sheep, cattle, goats), which are the main hosts of *Brucella*. The main routes of human infection include direct contact with infected livestock (e.g. assisting delivery, feeding) and the consumption of unsterilized livestock-derived dairy products (e.g. raw milk, fresh cheese) [3]. *Brucella* causes chronic symptoms such as fever, arthritis and orchitis in humans and induces abortion and reduced milk production in cattle and sheep. Refractory persistent infection and even lifelong sequelae may occur in severe cases, as the nervous and cardiovascular systems become involved [2,4–6]. Currently, the prevention and control of brucellosis face several key challenges: (1) although existing livestock vaccines offer good protective efficacy, their use is largely limited by safety concerns; (2) no human vaccine has yet been approved, so controlling the disease in humans depends heavily on managing infection in animal reservoirs; (3) the treatment of chronic infections remains difficult, highlighting the urgent need for more effective therapeutic approaches [7].

The virulence of *Brucella* mainly depends on its ability to invade and survive within host phagocytic cells including macrophages and dendritic cells. Macrophages, as key effector cells of the innate immune system, act not only as the primary site for intracellular colonization and replication of *Brucella* but also as critical intracellular niche that allow the bacterium to evade immune killing and establish chronic infection [8–10]. However, the intracellular survival mechanisms of *Brucella* have not yet been fully elucidated. In particular, the biological processes underlying its persistent infection in host cells and the associated molecular mechanisms require further clarification. Consequently, systematically identifying the key host genes that regulate invasion and early intracellular survival of *Brucella*, along with deciphering the molecular mechanisms underlying host-*Brucella* interactions, is essential for advancing our understanding of its intracellular lifestyle.

Genome-wide CRISPR knockout screen is a core technique for dissecting host gene regulatory networks in host–pathogen interactions [11,12]. Multiple key host genes regulating the intracellular survival of Mycobacterium tuberculosis , including *VPS18*, *GID8*, *YPEL5*, have been identified via genome-wide CRISPR knockout screen [13–15]. A genome-wide CRISPR knockout screen revealed that *PTEN* was identified as a host factor that promotes *Listeria monocytogenes* adhesion to macrophages via its lipid phosphatase activity [16]. Genome-wide CRISPR knockout screen was performed to identify the key host factors regulating Salmonella Typhimurium infection, including *TMEM127*, *WWP2*, *ATG4D*, *ACTR3*, *ARPC4*, *NHLRC2* [17–19]. A genome-wide CRISPR knockout screen revealed that host genes *C1orf43* and *KIAA1109* regulate *Legionella pneumophila* phagocytosis, while *RAB10* and its chaperone *RABIF* mediate the maturation of Legionella-containing vacuoles (LCV) and bacterial replication [20]. Based on HeLa epithelial cells and their cell viability phenotypes, host genes *GOLGA6L6* and *DEFB103B* affecting *Brucella* intracellular survival were identified via a genome-wide CRISPR knockout screen [21]. However, systematic, genome-wide data are still lacking to further elucidate the host genes that affect invasion and intracellular survival of *Brucella*. In this study, a genome-wide CRISPR knockout library was constructed in the *Brucella*-susceptible human monocytic macrophage cell line THP-1. After infecting this library with red fluorescently labelled *Brucella*, flow cytometry was used to sort uninfected cells, enabling systematic screening and identification of key host genes that regulate the invasion and intracellular survival of *Brucella*, including *WDR4*, *ZNF532*, *MTHFD1*, and *TRAPPC2*.

## Results

### A genome-wide CRISPR knockout screen was performed to identifies host genes associated with Brucella invasion and intracellular survival

**Figure 1.**
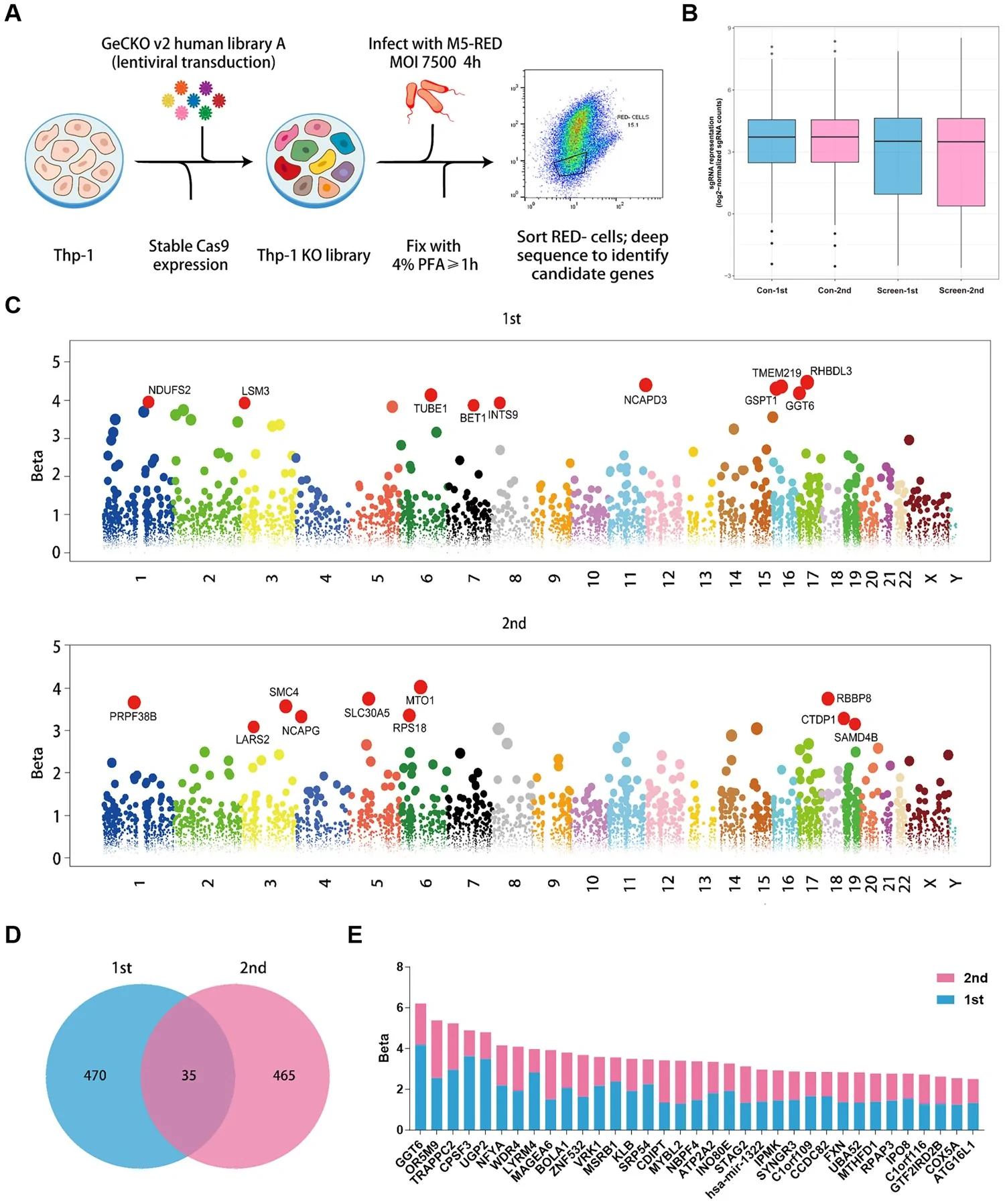
Genome-wide CRISPR Knockout screen in *Brucella* infection. (A) Schematic workflow of genome-wide CRISPR knockout screening in THP-1 cells. A pooled CRISPR knockout library was constructed using the Human Gecko v2 Library A lentiviral library, followed by loss-of-function screening upon *Brucella* infection. (B) Box plots of normalized sgRNA counts in control and experimental groups from two independent screens. (C) Manhattan plots of enriched genes from two parallel positive screens. Each dot represents a single gene; the x-axis indicates genomic position, and the y-axis represents the Beta. Labelled genes represent the top 10 hits in each screen. (D) Venn diagram illustrating the overlap of enriched genes from the two parallel positive screens, identifying 35 candidate host genes. (E) Ranking of the 35 candidate genes based on their mean beta scores across the two independent screens.

To identify host genes associated with *Brucella* invasion and intracellular survival, the Human GeCKO v2 Library A, covering 19,050 human target genes and 65,383 single-guide RNAs (sgRNAs), was transfected into human monocytic THP-1 Cas9 cells to generate a THP-1 knockout library. Subsequently, the THP-1 knockout library cells were infected with red fluorescently labelled *B.melitensis* M5 (M5-RED) at a multiplicity of infection (MOI) of 7500 for 4 h. Under these conditions, M5-RED invasion rate reached 93.6% and THP-1 cell viability remained at 99.8% (Figure S1). The control THP-1 knockout library cells were not subjected to infection treatment. After eliminating extracellular bacteria, genomic DNA (gDNA) was extracted from both the sorted RED-negative cells and the control THP-1 knockout library cells for deep sequencing (Figure 1(A)). Results showed that a subset of sgRNAs was strongly enriched in the sorted RED-negative cells (Figure 1(B), Table S1). Summary reports of corresponding host genes from 2 independent experiments were generated for enriched sgRNAs identified from each screen using MAGeCK [22] (Figure 1(C), Figure S2, Table S2). This identified 505 and 500 differential candidate host genes, respectively (Figure 1(D)). A total of 35 overlapping genes were ranked by mean beta values (Figure 1(D,E)). Here, positive beta scores indicate genes conferring infection resistance when knocked out, while negative scores indicate susceptibility.

### Host genes TRAPPC2, NFYA, WDR4, ZNF532, and MTHFD1 were associated with Brucella invasion and intracellular survival

**Figure 2.**
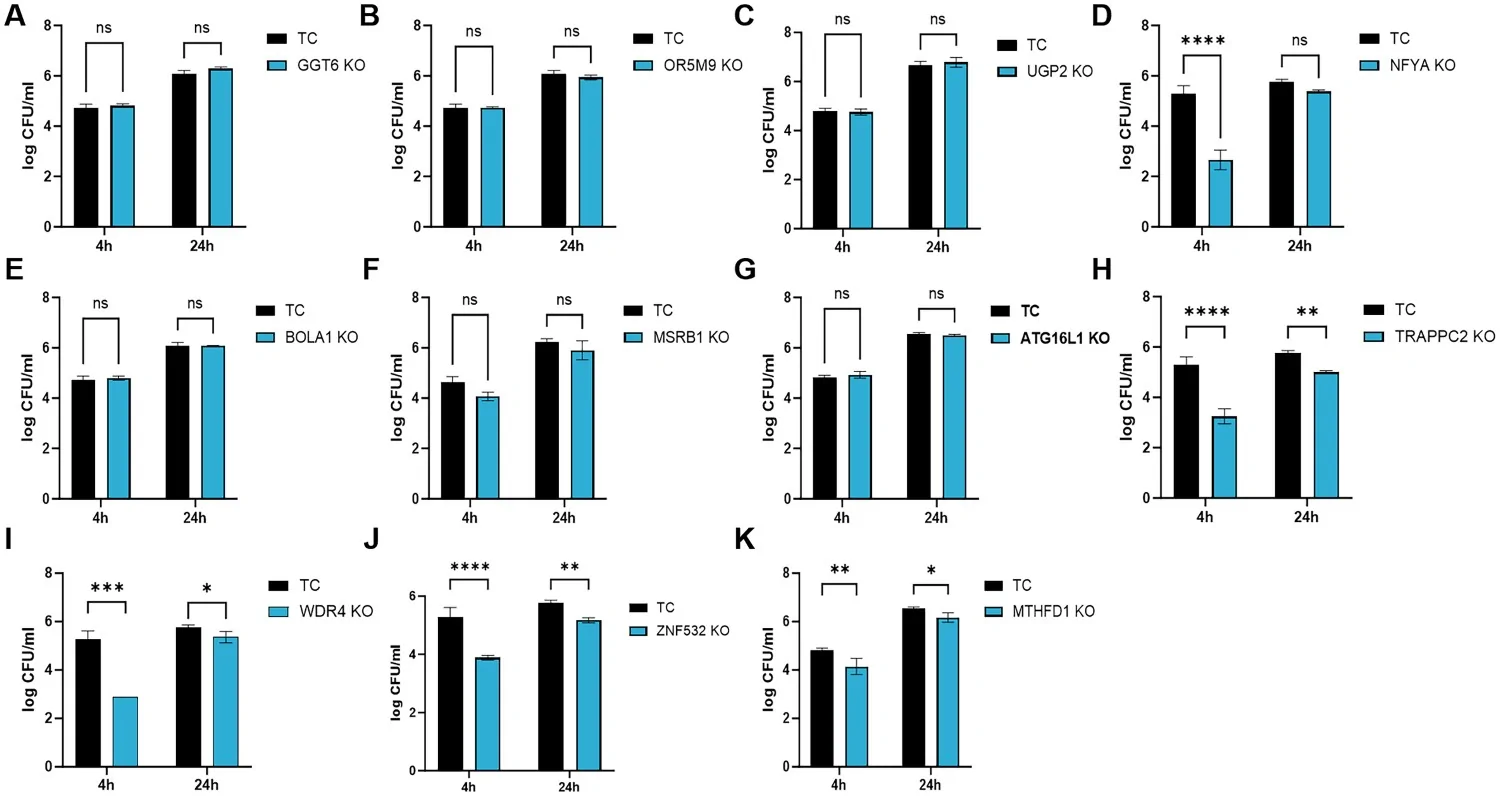
Intracellular survival of M5 in THP-1 knockout cell lines. THP-1 monoclonal knockout cell lines targeting 11 candidate host genes were infected with M5 at an MOI of 100. Intracellular bacterial loads were assessed by CFU enumeration at 4 and 24 h post-infection. Data are presented as mean ± SD. Statistical analysis was performed using Student’s t-test. TC, THP-1 Cas9 control cells; ns, not significant; *P < 0.05; **P < 0.01; ***P < 0.001; ****P < 0.0001.

To validate the genome-wide CRISPR knockout screening results, 11 candidate host genes, *GGT6* (Gamma-Glutamyltransferase 6), *OR5M9* (Olfactory Receptor Family 5 Subfamily M Member 9), *UGP2* (UDP-Glucose Pyrophosphorylase 2), *NFYA* (Nuclear Transcription Factor Y Subunit α), *BOLA1* (BolA Family Member 1), *MSRB1* (Methionine Sulfoxide Reductase B1), (Autophagy Related 16 Like 1), *TRAPPC2* (Trafficking Protein Particle Complex Subunit 2), *WDR4* (*WDR4* tRNA N7-Guanosine Methyltransferase Non-Catalytic Subunit), *ZNF532* (Zinc Finger Protein 532), and *MTHFD1* (Methylenetetrahydrofolate Dehydrogenase, Cyclohydrolase and Formyltetrahydrofolate Synthetase 1), were respectively knocked out in THP-1 cells (Figure S3). After the knockout, THP-1 cells were infected with *B. melitensis* M5 (M5), and the intracellular bacterial loads were quantified at 4 and 24 h post-infection. The results showed that knockout of *NFYA*, *TRAPPC2*, *WDR4*, *ZNF532*, or *MTHFD1* significantly reduced intracellular bacterial loads at 4 h post-infection compared with parental cells (Figure 2(D, H–K)), whereas this reduction was not observed in *GGT6*, *OR5M9*, *UGP2*, *BOLA1*, *MSRB1*, and *ATG16L1* knockout cells (Figure 2(A–C,E–G)). Further, *TRAPPC2*, *WDR4*, *ZNF532*, and *MTHFD1* knockout were also significantly reduced intracellular bacterial loads at 24 h post-infection compared with parental cells (Figure 2(H–K)).

### Knockout of WDR4 significantly reduced Brucella invasion and early intracellular replication

**Figure 3.**
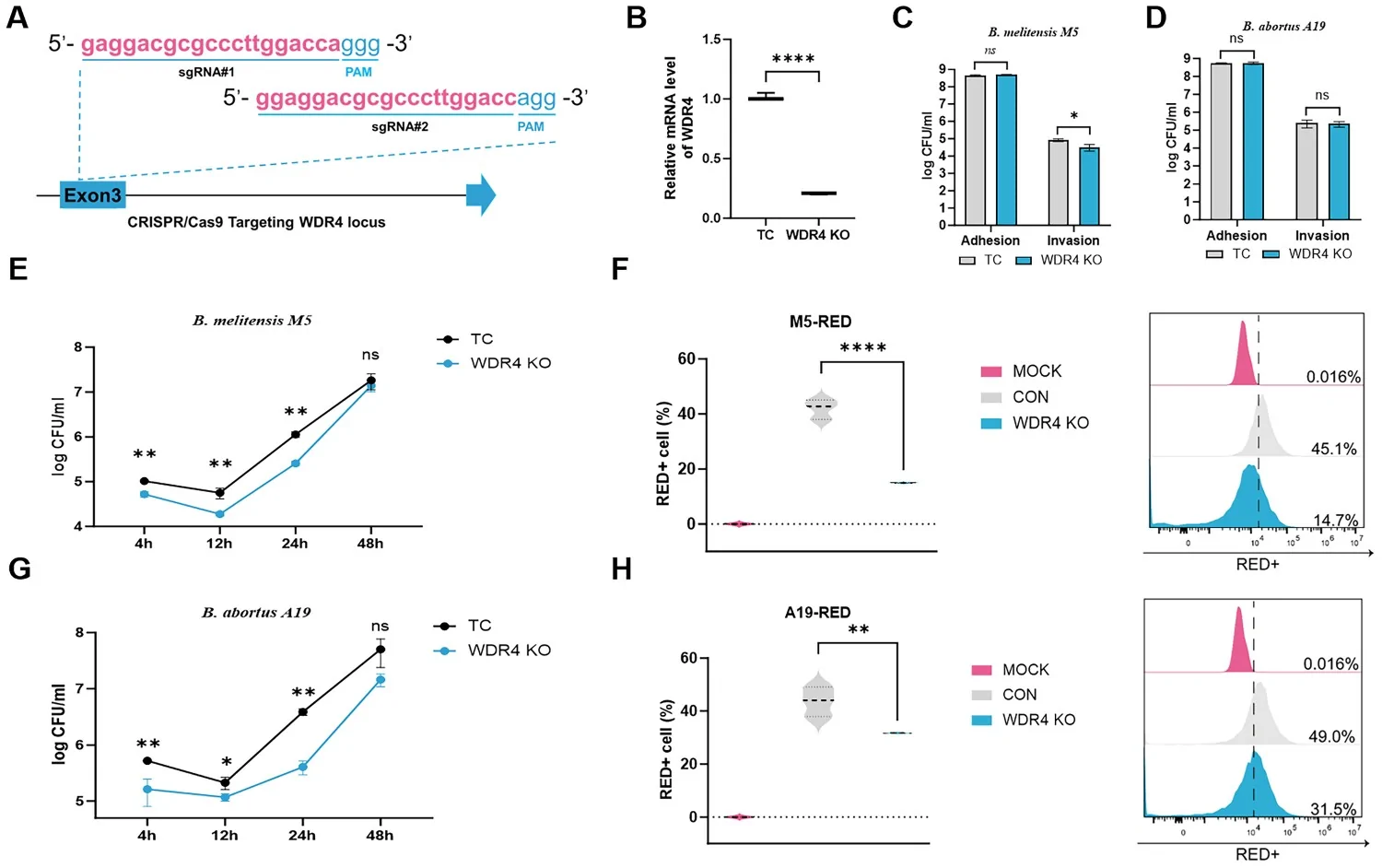
Functional validation of *WDR4* during *Brucella* infection in THP-1 cells. (A) Schematic of the CRISPR/Cas9 targeting strategy for *WDR4* knockout. (B) Validation of *WDR4* knockout efficiency by RT-qPCR. (C, D) Adhesion and invasion abilities of M5 and A19 in control and *WDR4* KO cells. (E, G) Intracellular survival of M5 and A19 in control and *WDR4* KO cells. (F, H) Invasion rate of M5 and A19 in control and *WDR4* KO cells. Data are presented as mean ± SD. Statistical analysis was performed using Student’s *t*-test. TC, THP-1 Cas9 control cells; ns, not significant; *P < 0.05; **P < 0.01; ***P < 0.001; ****P < 0.0001.

Figure 3(A,B) showed that the host gene *WDR4* was successfully knocked out in THP-1 cells. To further investigate the role of *WDR4* during *Brucella* infection, *WDR4* KO cells were infected with M5 and *B. abortus* A19 (A19), respectively. The results demonstrated that knockout of *WDR4* did not affect the adhesion of M5 or A19 to host cells (Figure 3(C,D)). However, it significantly reduced both the invasion of M5 and intracellular bacterial loads of M5 and A19 at 4, 12, and 24 h post-infection (Figure 3(E,G)). Furthermore, *WDR4* KO cells were also infected with M5-RED and red fluorescently labelled *B. abortus* A19-RED (A19-RED), and then the bacterial invasion rates were analyzed by flow cytometry. Consistent with these findings, *WDR4* deficiency significantly decreased the invasion rate of both M5-RED and A19-RED, compared with the parental cells (Figure 3(F,H)).

### Knockout of ZNF532 significantly reduced Brucella invasion and early intracellular replication

**Figure 4.**
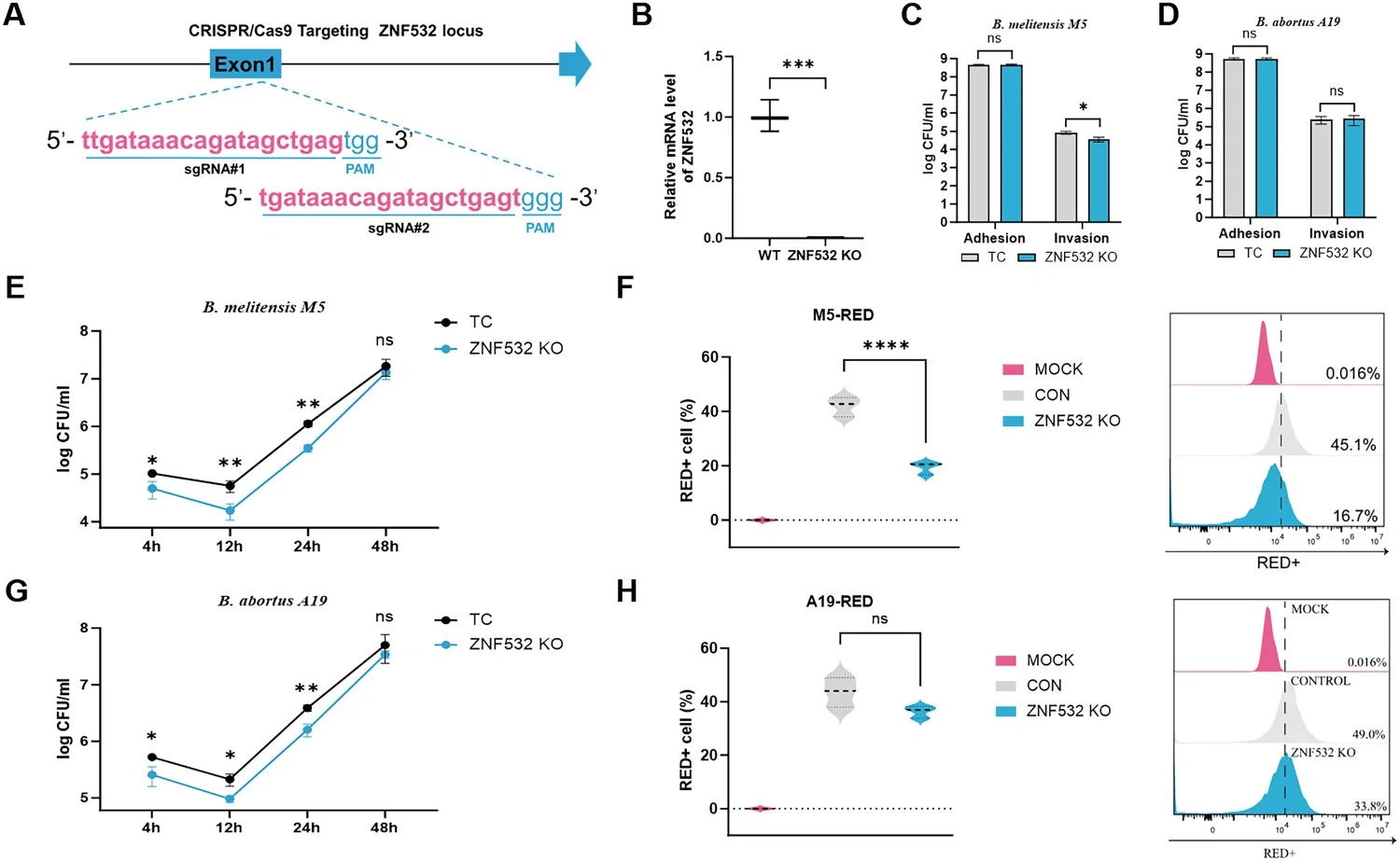
Functional validation of *ZNF532* during *Brucella* infection in THP-1 cells. (A) Schematic of the CRISPR/Cas9 targeting strategy for *ZNF532* knockout. (B) Validation of *ZNF532* knockout efficiency by RT-qPCR. (C, D) Adhesion and invasion abilities of M5 and A19 in control and *ZNF532* KO cells. (E, G) Intracellular survival of M5 and A19 in control and *ZNF532* KO cells. (F, H) Invasion rate of M5 and A19 in control and *ZNF532* KO cells. Data are presented as mean ± SD. Statistical analysis was performed using Student’s *t*-test. TC, THP-1 Cas9 control cells; ns, not significant; *P < 0.05; **P < 0.01; ***P < 0.001; ****P < 0.0001.

Figure 4(A,B) showed that the host gene *ZNF532* was successfully knocked out in THP-1 cells. To further investigate the function of *ZNF532* during *Brucella* infection, *ZNF532* KO cells were infected with M5 and A19, respectively. The results showed that *ZNF532* deficiency did not affect the adhesion of M5 or A19 to host cells (Figure 4(C,D)), but it significantly reduced both the invasion of M5 and the intracellular loads of M5 and A19 at 4, 12, and 24 h post-infection (Figure 4(E,G)). Furthermore, *ZNF532* KO cells were also infected with M5-RED and A19-RED, and then the bacterial invasion rates were analyzed by flow cytometry. Compared with parental cells, *ZNF532* deficiency significantly decreased the M5 RED invasion rate, but did not affect the A19 RED invasion rate (Figure 4(F,H)).

### Knockout of MTHFD1 significantly reduced Brucella invasion and early intracellular replication

**Figure 5.**
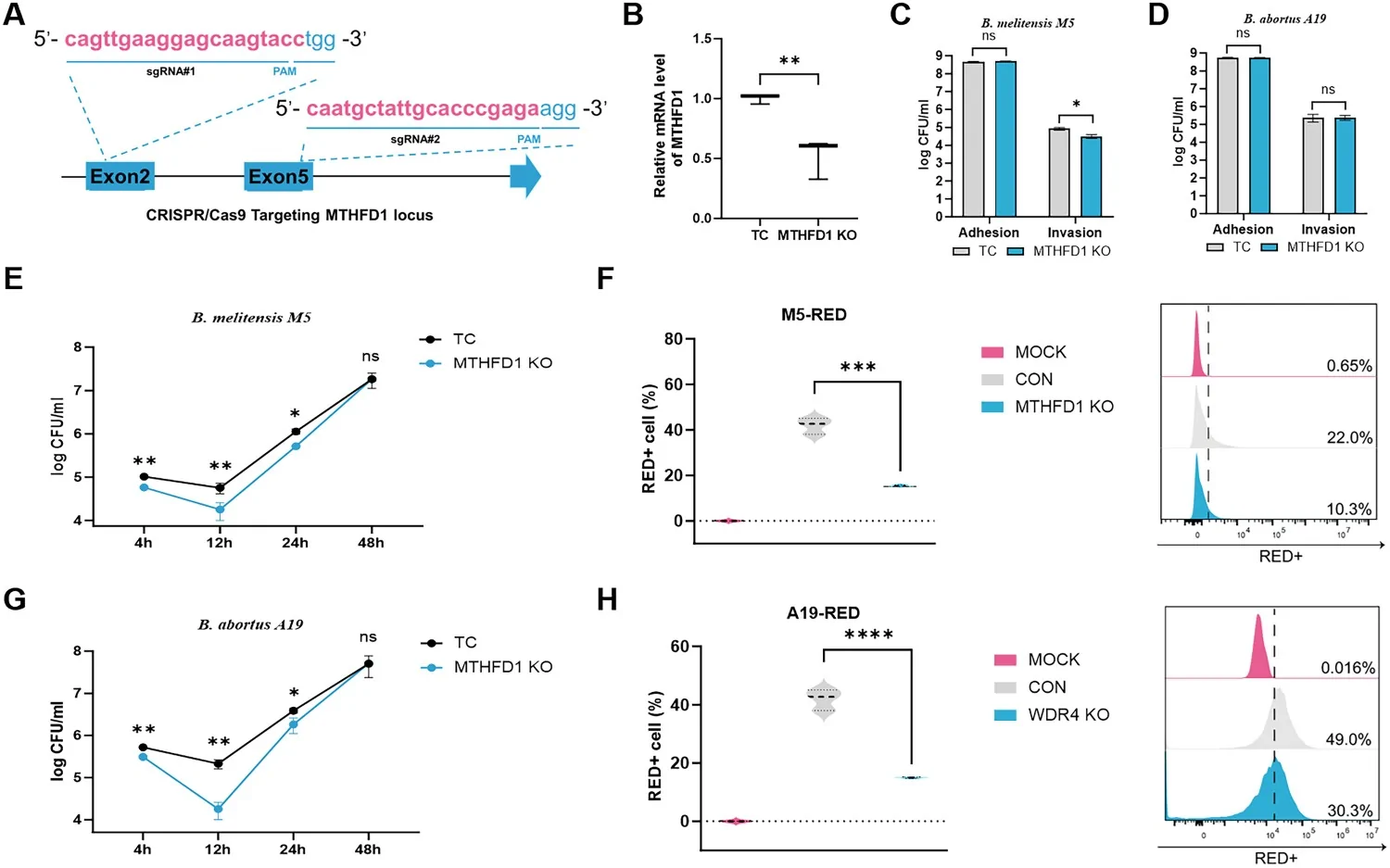
Functional validation of *MTHFD1* during *Brucella* infection in THP-1 cells. (A) Schematic of the CRISPR/Cas9 targeting strategy for *MTHFD1* knockout. (B) Validation of *MTHFD1* knockout efficiency by RT-qPCR. (C, D) Adhesion and invasion abilities of M5 and A19 in control and *MTHFD1* KO cells. (E, G) Intracellular survival of M5 and A19 in control and *MTHFD1* KO cells. (F, H) Invasion rate of M5 and A19 in control and *MTHFD1* KO cells. Data are presented as mean ± SD. Statistical analysis was performed using Student’s *t*-test. TC, THP-1 Cas9 control cells; ns, not significant; *P < 0.05; **P < 0.01; ***P < 0.001; ****P < 0.0001.

Figure 5(A,B) showed that the host gene *MTHFD1* was successfully knocked out in THP-1 cells. To further investigate the role of *MTHFD1* during *Brucella* infection, *MTHFD1* KO cells were infected with M5 and A19, respectively. The results showed that *MTHFD1* deficiency did not affect the adhesion of M5 or A19 to host cells (Figure 5(C,D)), but it significantly reduced both M5 invasion and intracellular bacterial loads of M5 and A19 at 4, 12, and 24 h post-infection (Figure 5(E,G)). Furthermore, *MTHFD1* KO cells were also infected with M5-RED and A19-RED, and then the bacterial infection rates were analyzed by flow cytometry. The results also showed that, compared with the parental cells, *MTHFD1* knockout significantly reduced the infection rates of both M5-RED and A19-RED (Figure 5(F,H)).

### Knockout of TRAPPC2 significantly reduced Brucella invasion and intracellular survival

**Figure 6.**
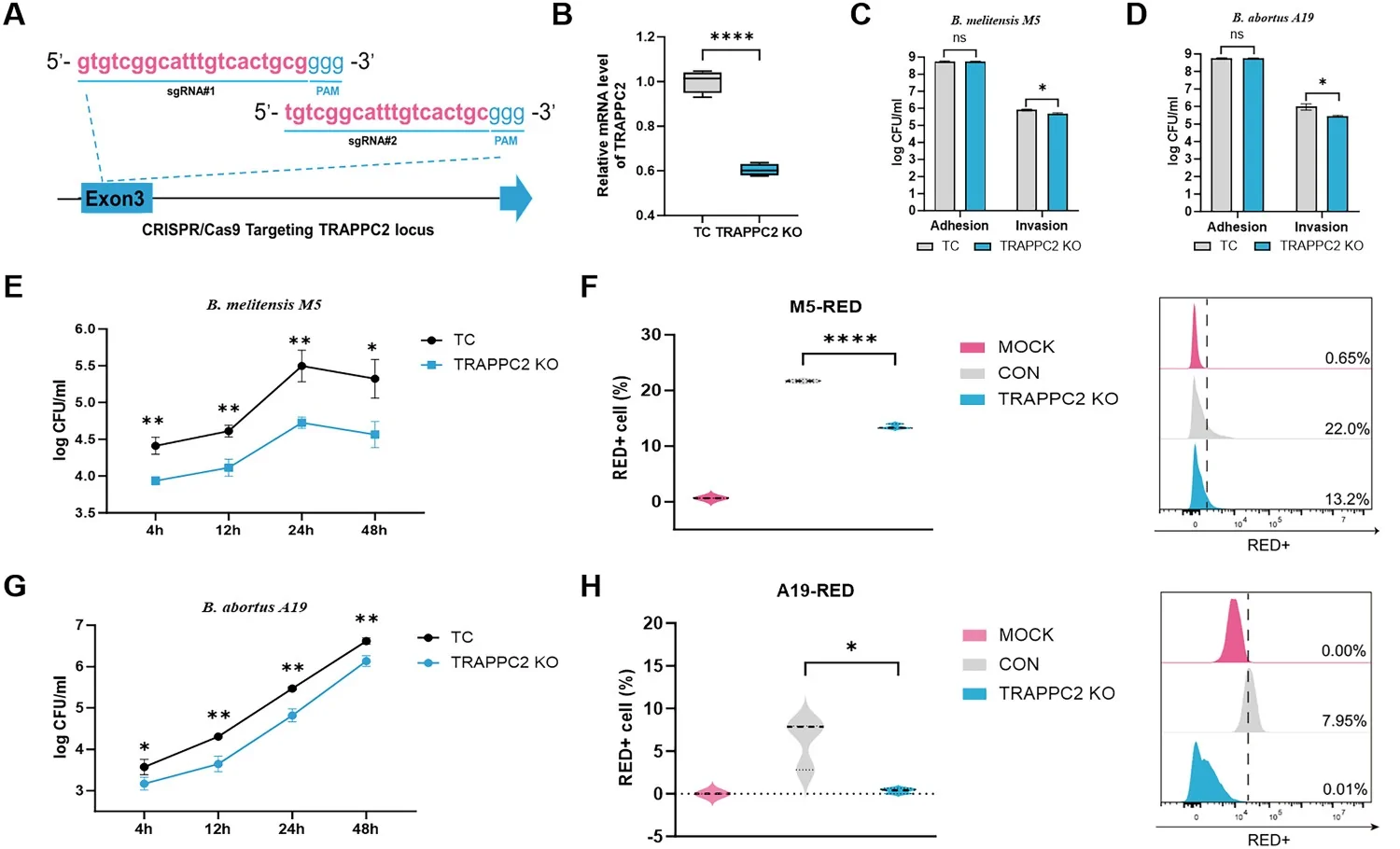
Functional validation of *TRAPPC2* during *Brucella* infection in THP-1 cells. (A) Schematic of the CRISPR/Cas9 targeting strategy for *TRAPPC2* knockout. (B) Validation of *TRAPPC2* knockout efficiency by RT-qPCR. (C, D) Adhesion and invasion abilities of M5 and A19 in control and *TRAPPC2* KO cells. (E, G) Intracellular survival of M5 and A19 in control and *TRAPPC2* KO cells. (F, H) Invasion rate of M5 and A19 in control and *TRAPPC2* KO cells. Data are presented as mean ± SD. Statistical analysis was performed using Student’s *t*-test. TC, THP-1 Cas9 control cells; ns, not significant; *P < 0.05; **P < 0.01; ***P < 0.001; ****P < 0.0001.

Figure 6(A,B) showed that the host gene *TRAPPC2* was successfully knocked out in THP-1 cells. To further investigate the function of *TRAPPC2* during *Brucella* infection, *TRAPPC2* KO cells were infected with M5 and A19, respectively. The results showed that *TRAPPC2* deficiency did not affect the adhesion of M5 or A19 to host cells (Figure 6(C,D)), but it significantly decreased both invasion by M5 and A19 and their intracellular bacterial loads at 4, 12, 24, and 48 h post-infection (Figure 6(E,G)). Furthermore, *TRAPPC2* KO cells were also infected with M5-RED and A19-RED, and then the bacterial infection rates were analyzed by flow cytometry. The results also showed that, compared with the parental cells, *TRAPPC2* deficiency significantly reduced infection rates of both M5-RED and A19-RED (Figure 6(F,H)).

### TRAPPC2 regulates Brucella invasion and intracellular replication by modulating cellular autophagy

**Figure 7.**
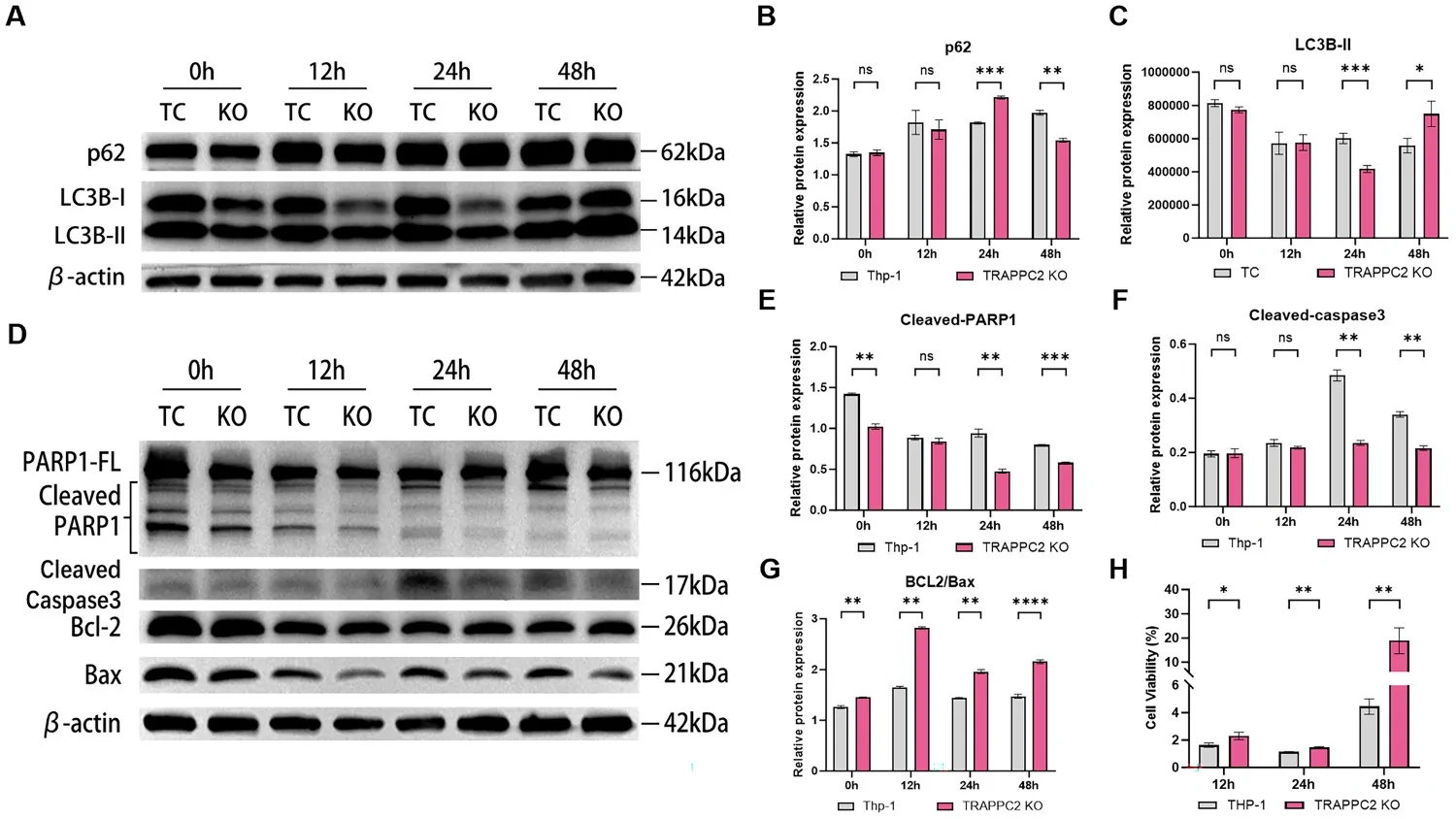
*TRAPPC2* deficiency regulates autophagy and apoptosis in *Brucella*-infected THP-1 cells. (A, D) Western blot analysis of autophagy markers (p62, LC3B) and apoptosis markers (PARP1, caspase-3, Bcl-2/Bax) in control and *TRAPPC2* KO cells at various time points following *Brucella* infection. (B, C, E, F, G) Relative protein expression levels were quantified by densitometric analysis. (H) Viability of control and *TRAPPC2* KO cells post-infection. Data are presented as mean ± SD. Statistical analysis was performed using Student’s *t*-test. TC, THP-1 Cas9 control cells; ns, not significant; *P < 0.05; **P < 0.01; ***P < 0.001; ****P < 0.0001.

Among *TRAPPC2*, *NFYA*, *WDR4*, *ZNF532*, and *MTHFD1*, the *TRAPPC2* gene knockout resulted in the most significant reduction in *Brucella* invasion and intracellular survival. Therefore, the underlying mechanism of *TRAPPC2*’s effect was further investigated. *TRAPPC2* KO cells were infected with M5 and the expression of autophagy- and apoptosis-related proteins was detected at 0, 12, 24, and 48 h post-infection. The results showed that the expression of autophagy substrate p62 in *TRAPPC2* KO cells was significantly increased at 24 h post-infection compared with the parental cells, but it significantly decreased at 48 h post-infection (Figure 7(A,B)). Meanwhile, the level of the autophagy marker LC3B-II in *TRAPPC2* KO cells was significantly lower than that in parental cells at 24 h post-infection, but it was significantly higher than that in parental cells at 48 h post-infection (Figure 7(A,C)). These results indicated that *TRAPPC2* knockout suppresses autophagosome formation and causes autophagy substrate accumulation 24 h post-infection, but a reversible effect was observed at 48 h post-infection.

Furthermore, analysis of apoptosis-related proteins revealed that levels of cleaved-PARP1 and cleaved-Caspase-3 in *TRAPPC2* KO cells were significantly decreased at 24 and 48 h post-infection, compared with the parental cells (Figure 7(D–F)). Moreover, *TRAPPC2* knockout significantly increased the ratio of the anti-apoptotic protein Bcl-2 to the pro-apoptotic protein Bax at 0, 12, 24, and 48 h post-infection (Figure 7(G)), indicating that *TRAPPC2* knockout enhanced anti-apoptotic capacity and blockaded *Brucella*-induced host cell apoptosis. CCK-8 cell viability assays confirmed that *TRAPPC2* KO cells exhibited significantly higher viability than the parental cells at 0, 12, 24, and 48 h post-infection, demonstrating that *TRAPPC2* knockout alleviates *Brucella*-induced cell death and promotes host cell survival (Figure 7(H)).

To determine whether *TRAPPC2* is uniquely required by *Brucella*, THP-1 Cas9 and *TRAPPC2*-KO cells were infected with *S. enterica serovar Abortusovis* BAA-1835, another intracellular bacterium. *TRAPPC2* deficiency did not affect *S. enterica serovar Abortusovis BAA-*1835 survival at 4, 12, or 24 h post-infection. Taken together, given that autophagy is critical for macrophage clearance of intracellular *Brucella* [9,23–26], *TRAPPC2* specifically regulates *Brucella* invasion and intracellular replication by modulating cellular autophagy, whereas its deficiency protects host cells from *Brucella*-induced apoptosis.

## Discussion

To investigate the mechanisms of *Brucella* invasion and intracellular survival, we combined a genome-wide CRISPR knockout screen with flow cytometry sorting in THP-1 cells. Based on a previous high-MOI model where *Brucella melitensis* at an MOI of 5000 did not compromise cell viability [27], our use of a high MOI (7500) achieved over 90% infection and survival rates, successfully ensuring maximal selection coverage and minimizing false positives in the CRISPR screen without inducing non-specific cytotoxicity. We ensured that both infected and non-infected groups underwent identical experimental procedures, including cell culture maintenance, buffer exposure, and high-speed fluorescence-activated cell sorting (FACS). To further minimize false positives, we employed a strict intersection strategy across two independent parallel screens, yielding 35 candidate genes. For individual validation, we selected 11 genes from the 35 overlapping candidates based on their biological relevance to host–pathogen interactions (Table S3) and potential novelty [28,29]. These candidates systematically represent three core functional modules, independent of their primary screen scores: metabolic reprogramming (*MTHFD1*, *GGT6*, *UGP2*, *MSRB1*), epigenetic and transcriptional regulation (*WDR4*, *ZNF532*, *NFYA*), and organelle homeostasis (*ATG16L1*, *BOLA1*, *TRAPPC2*). Notably, we first identified key host genes that regulate bacterial invasion and survival, including *TRAPPC2*, *WDR4*, *ZNF532*, and *MTHFD1*.

In macrophages lacking *WDR4*, *ZNF532*, or *MTHFD1*, the early inhibitory effect on strain A19 was less pronounced than on strain M5 (Figures 3–5(C, D, F, H)). Specifically, while *ZNF532* deficiency diminished intracellular loads for both strains at 4 h post-infection, flow cytometry revealed that this reduction in A19 was attributable to compromised early intracellular survival rather than defective entry (Figure 4(G, H)). This phenotypic discrepancy reflects the divergent intracellular trafficking of these strains: M5 actively evades lysosomal degradation, whereas A19 clearance remains contingent upon lysosomal and pro-inflammatory pathways [30]. Thus, *ZNF532* may selectively mediate early survival by modulating these clearance mechanisms, thereby dictating strain-specific host defense.

*WDR4*, *ZNF532*, and *MTHFD1* act as critical host factors that orchestrate early defense against *Brucella* by regulating immune signalling and metabolic homeostasis. *WDR4*, a component of the m⁷G methyltransferase complex [31,32], appears to modulate cholesterol efflux and the translation of immune effectors, as its deficiency was associated with compromised pro-inflammatory cytokine expression and phagocytic capacity [33,34]. Although systemic or tissue-specific *WDR4* deletion has been associated with severe physiological abnormalities [35,36], making it unsuitable for in vivo modelling, it remains a potential target for early anti-*Brucella* intervention. *ZNF532* may function as a ceRNA for miR-20b-5p to sustain STAT3 signalling [28,37–39]. Its role in stabilizing *PIK3CD* is potentially linked to efficient endosome maturation [39], and its loss might hinder the formation of the lipid-modified niche required for bacterial immune evasion [40,41]. Furthermore, *MTHFD1*-mediated folate-dependent one-carbon metabolism is thought to provide essential nucleotides for pathogen replication [42–44]. Given the limited exogenous nucleotides in the phagosomal environment, *MTHFD1* depletion appears to restrict the availability of endogenous precursors, thereby constraining bacterial colonization [45–47]. Notably, *MTHFD1* has been identified as a broad-spectrum anti-RNA virus target [43], suggesting its regulated pathway may also represent a potential anti-*Brucella* strategy.

Among all screened genes, *TRAPPC2* knockout exhibited the strongest inhibitory effect on *Brucella* intracellular infection. This study first demonstrated that *TRAPPC2* knockout significantly reduces *Brucella* intracellular survival and infection in macrophages by decreasing host autophagy. As an essential component of TRAPPⅢ complex assembly, *TRAPPC2* localizes to the pre-autophagosomal structure (PAS), activates GTPase Ypt1/Rab1, recruits Atg1, and regulates autophagosome formation/maturation [48,49]. Functionally, *TRAPPC2* knockout directly abrogates LC3-II lipidation, LC3 puncta accumulation, and autophagosome formation in macrophages [50,51]. Given that autophagy is critical for macrophage clearance of intracellular *Brucella* [9,23–26], and based on our experimental results, this early autophagy attenuation induced by *TRAPPC2* knockout accounts for the reduced bacterial survival and infection. Interestingly, the *TRAPPC2* knockout cell line showed an upregulation of autophagy levels at 48 h post-infection, suggesting that *Brucella* may exploit alternative pathways to manipulate host autophagy during later infection stages, potentially mediated by T4SS effector proteins [24,26]. Apart from this autophagy dynamic, *TRAPPC2* KO cells also showed downregulated apoptosis post-infection, a previously unreported phenomenon whose mechanism requires further investigation. Notably, *Brucella* typically inhibits host apoptosis to favour survival [52]. This indicates that in *TRAPPC2*-KO macrophages, the antibacterial effect of impaired autophagy overrides the pro-survival benefits of attenuated apoptosis, identifying the *TRAPPC2*-mediated autophagic axis as the primary determinant of infection restriction. However, mutations in *TRAPPC2*, such as D47Y, are known to cause severe X-linked spondyloepiphyseal dysplasia tarda (SEDT) [53], which highlights the risks of direct protein inhibition. Accordingly, CRISPR base-editing screens could be used to identify specific mutations that resist *Brucella* infection while preserving essential cellular trafficking, offering a safer therapeutic strategy.

## Conclusion

In summary, our genome-wide CRISPR knockout screen identifies *TRAPPC2*, *ZNF532*, *MTHFD1*, and *WDR4* as novel host factors essential for *Brucella* invasion and survival. Notably, we demonstrate for the first time that *TRAPPC2* deficiency restricts invasion and survival by inhibiting host autophagy.

## Materials and methods

### Strains and cell lines

THP-1 and THP-1 Cas9 (EDITGENE, EDC01205) were maintained at 37°C with 5% CO₂ in RPMI 1640 medium (Gibco, C11875500BT) supplemented with either 10% or 1% heat-inactivated fetal bovine serum (Shuang Ru Biotech, S711-001S) and 1% penicillin–streptomycin (Gibco, 15140122), along with 100 μg/ml penicillin and 100 μg/ml streptomycin.

All *Brucella* strains were routinely cultured at 37°C with 5% CO₂ in tryptic soy agar (BD, 236950) or on tryptic soy broth (BD, 211825) in a BSL2 biosafety laboratory. *Brucella melitensis* M5 and *Brucella abortus* A19 are preserved in our laboratory. The strain *Salmonella enterica serovar Abortusovis* BAA-1835 was obtained from the American Type Culture Collection (ATCC). When necessary, kanamycin (Solarbio, K1030) was added to the culture medium at a final concentration of 50 µg/ml.

### Construction of THP-1 knockout library

THP-1 cells were used as the model, and the Human GeCKOv2 CRISPR Knockout Pooled Library A was adopted to construct a genome-wide knockout library cell line. The construction and preparation of lentivirus were referred to previous reports [21]. THP-1 cas9 cells stably expressing cas9 protein were seeded into 2 T175 culture flasks, and the cells were infected with lentivirus at a multiplicity of infection (MOI) of 0.3 to construct the library. 24 h after transduction, 2.5 µg/mL puromycin was added for 7 days of screening. The surviving cells were sorted as library cells and expanded in culture for subsequent *Brucella* infection screening.

### Genome-wide CRISPR screen for Brucella infection

2.0 × 10⁷ THP-1 knockout library cells were collected as the uninfected control group for analyzing the initial single-guide RNA (sgRNA) abundance. Meanwhile, 1 × 10⁸ of these cells were seeded into 6-well plates (2 × 10⁶ cells/well, RPMI 1640 medium with 2% serum and no antibiotics), and infected with M5-RED at an MOI of 7500. The infected plates were centrifuged at 400 g for 5 min, followed by incubation at 37 °C for 4 h. After infection, 2 μl of 50 μg/ml gentamicin was added to each well to kill extracellular bacteria for 1 h. The cells were collected, fixed with 4% PFA fixative for more than 30 min, and then 2 × 10⁷ RED^−^ cells in the experimental group were sorted by flow cytometer. Genomic DNA extraction from the cells of both groups, along with deep targeted sequencing, sgRNA enrichment analysis, and significant target gene screening (MAGeCK), was commissioned to Tangtang Tianxia Biotechnology Co., Ltd. The experiments were conducted in parallel twice, and host genes identified as positively selected in both independent experiments were regarded as candidate genes.

### Construction of monoclonal knockout cell lines

SgRNA oligonucleotide pairs targeting the target gene were synthesized (Table S4), annealed, and cloned into the BsmBI-linearized lentiviral Lenti-sg-eGFP plasmid (Addgene, 51760). According to the instructions of JetPrime (Polyplus Transfection, 101000046), the sgRNA-containing vector was co-transfected with psPAX2 and pMD2G plasmids into HEK293 T cells to prepare lentivirus, which was then used to infect THP-1 cas9 cells. Forty-eight hours after infection, eGFP-positive monoclonal cells were sorted into 96-well plates by flow cytometry; after single-cell colonies formed, they were gradually passaged to 24-well plates for expansion culture. Using the primers in Table S4, the genomic DNA fragment of the target gene region targeted by sgRNA was amplified by PCR, and Sanger sequencing was performed to identify the knockout efficiency. The successfully knocked-out cells were expanded and cryopreserved for functional verification of candidate genes.

### Validation of gene knockout by RT-qPCR

THP-1 cells and monoclonal knockout cell lines were collected. Total RNA was extracted using the Total RNA Extraction Kit (Vazyme, RC112-01), and reverse-transcribed into cDNA using the Reverse Transcription Kit (Vazyme, R323-01) according to the instructions. Using cDNA as the template, gene-specific primers and internal reference gene primers (Table S4) were used for amplification reaction with the Quantitative Real-Time PCR Kit (Vazyme, Q711-02). The relative expression level of *TRAPPC2* gene was calculated by the 2–ΔΔCt method, and the expression difference between wild-type cells and knockout cells was compared to determine the gene knockout effect.

### Adhesion, invasion, and intracellular survival assays

THP-1 and *TRAPPC2* KO cells were seeded into 24-well plates at a density of 3 × 10⁵ cells per well and cultured in RPMI 1640 medium supplemented with 2% FBS. Cells were infected with M5 and A19 strains at a MOI of 100 colony-forming units (CFU) per cell for 1 h. To synchronize the infection, the infected plates were centrifuged at 400 g for 5 min, followed by incubation at 37 °C for 1 h. After infection, 1 μl of 50 μg/ml gentamicin was added to each well to eliminate extracellular bacteria. Cell samples were collected as follows: the non-sterilized group at 1 h post-infection (for adhesion), the sterilized group at 1 h post-sterilization (for invasion), and samples at 4, 12, 24, and 48 h post-infection (for intracellular survival). Cells were centrifuged at 1100 rpm for 5 min, the supernatant was discarded, and the pellets were resuspended and lysed in 500 μl of 0.1% Triton X-100-PBS. After 10 min of incubation at room temperature, lysates were serially diluted 10-fold and plated to determine CFUs. All assays were conducted in triplicate.

### Detection of invasion rate

THP-1 and *TRAPPC2* KO cells were seeded into 6-well plates at 1 × 10⁶ cells/well and cultured in RPMI 1640 medium supplemented with 2% FBS. Cells were infected with M5-RED and A19-RED strains at an MOI of 1000. The infected plates were centrifuged at 400 g for 5 min, followed by incubation at 37 °C for 4 h. After infection, the addition of 2 μl of 50 μg/ml gentamicin to each well was followed by incubation for 1 h to eliminate extracellular bacteria. Cells were collected by pipetting and mixing, centrifuged at 1100 rpm for 5 min, and the supernatant was discarded. Cells were gently resuspended and washed twice with PBS, then fixed with 300 μl of 4% paraformaldehyde fixative for more than 30 min. After filtration through a mesh, the proportion of RED + cells was detected by flow cytometry to calculate the bacterial invasion rate. Experiments were performed in triplicate.

### Cell viability assay

THP-1 and *TRAPPC2* KO cells were seeded into 96-well plates at 1 × 10⁴ cells/well and cultured in RPMI 1640 medium supplemented with 2% FBS. Cells were infected with M5 at an MOI of 100 for 1 h. After infection, 1 μl of 5 μg/ml gentamicin was added to each well to kill extracellular bacteria. Cell viability was detected using the Cell Counting Kit-8 (APExBIO, K2268) at 12 h, 24 h, and 48 h post-infection.

### Western blotting assay

Cells from each group were collected and lysed with 5×SDS-PAGE loading buffer (Solarbio, P1043), denatured by boiling at 100°C for 10 min. Protein concentration was quantified using the BCA Kit (Thermo Fisher Scientific, A55864). A total of 20 μg of protein was separated by SDS-PAGE and transferred to a 0.2 μm PVDF membrane (bio-rad, 1620177). The membrane was blocked with 5% non-fat milk-TBST at room temperature for 2 h, then incubated with primary antibodies at 4°C overnight. After washing three times with PBST, the membrane was incubated with HRP-conjugated secondary antibodies at room temperature (both primary and secondary antibodies were diluted according to the manufacturer’s instructions). Bands were visualized by chemiluminescence, scanned, and gray values were analyzed using ImageJ software. The relative expression level of the target protein was calculated with the internal reference as the benchmark. Primary antibodies used in the experiment included: anti-P62 antibody (T59081S, abmart), anti-LC3B antibody (T55992S, abmart), anti-PARP1 antibody (S0B0540, STARTER), anti-CLEAVED CASPASE3 antibody (9661, CST), anti-BAX antibody (S0B0230, STARTER), anti-BCL2 antibody (T40056, abmart), and anti-β-ACTIN antibody (66009-1-Ig, Proteintech). Secondary antibodies were ProteinFind® Goat Anti-Rabbit IgG (H + L), HRP Conjugate (HS101, TransGen) and ProteinFind® Goat Anti-Mouse IgG (H + L), HRP Conjugate (HS201, TransGen).

### Statistical analysis

Experimental data were expressed as mean ± standard deviation (mean ± SD). No experimental samples were excluded, and no randomization or blinding was performed. Comparisons involving two independent variables (genotype and time) were performed using two-way ANOVA. Single-variable comparisons used unpaired Student’s t-tests. Significance was set at *P < 0.05; **P < 0.01; ***P < 0.001; ****P < 0.0001.

## Supplementary Material

Table_S4.pdf

Fig S1.tif

Table_S3.pdf

Fig S4.tif

Fig S2.tif

Fig S3.tif

Table_S2.pdf

Table_S1.pdf

## Data Availability

All data supporting the findings of this study are available within the paper.
