# Supplementary material for: A genome-wide CRISPR knockout screen identified host genes essential for Brucella invasion and intracellular survival
Source: Emerg Microbes Infect. 2026 Aug 19;15(1):2713322. doi: 10.1080/22221751.2026.2713322 (PMC13491901; doi:10.1080/22221751.2026.2713322)
Supplement: Supplementary file 2 [file TEMI_A_2713322_SM6412.pdf]

Table S2 Positive candidate genes in 1st screen

Gene	Gene Name	sgRNA	group|beta	group|z	group|p-value	group|fdr	group|wald-p-value	group|wald-fdr	Description	sgRNA ID
ENSG00000167741	GGT6	1	4.1826	0.051609	0.00045954	0.6875	0.95884	0.99255	gamma-glutamyltransferase 6 [Source:HGNC Symbol;Acc:HGNC:26891]	HGLibA_19017
ENSG00000169744	LDB2	3	1.5352	0.52701	0.020128	0.83016	0.59818	0.95326	LIM domain binding 2 [Source:HGNC Symbol;Acc:HGNC:6533]	HGLibA_26214;HGLibA_26215;HGLibA_26213
hsa-mir-6878	hsa-mir-6878	2	1.2971	0.42139	0.038279	0.83236	0.67347	0.95326	-	HGLibA_63698;HGLibA_63695
ENSG00000105875	WDR91	1	1.3221	0.34436	0.036258	0.83236	0.73058	0.95633	WD repeat domain 91 [Source:HGNC Symbol;Acc:HGNC:24997]	HGLibA_54216
ENSG00000114529	C3orf52	2	1.4963	0.52142	0.022196	0.83016	0.60207	0.95326	chromosome 3 open reading frame 52 [Source:HGNC Symbol;Acc:HGNC:26255]	HGLibA_06247;HGLibA_06249
ENSG00000214113	LYRM4	2	2.8212	0.21384	0.0028491	0.81716	0.83068	0.96667	LYR motif containing 4 [Source:HGNC Symbol;Acc:HGNC:21365]	HGLibA_27752;HGLibA_27753
ENSG00000174428	GTF2IRD2B	3	1.2858	0.52862	0.039382	0.83398	0.59707	0.95326	GTF2I repeat domain containing 2B [Source:HGNC Symbol;Acc:HGNC:33125]	HGLibA_20574;HGLibA_20575;HGLibA_20573
ENSG00000101972	STAG2	1	1.34	0.34085	0.034465	0.83236	0.73322	0.95685	stromal antigen 2 [Source:HGNC Symbol;Acc:HGNC:11355]	HGLibA_47323
ENSG00000174437	ATP2A2	2	1.8074	0.33337	0.011213	0.82103	0.73886	0.95685	ATPase sarcoplasmic/endoplasmic reticulum Ca2+ transporting 2 [Source:HGNC Symbol;Acc:HGNC:812]	HGLibA_03609;HGLibA_03608
ENSG00000105829	BET1	1	3.865	0.065153	0.00091908	0.78846	0.94805	0.9901	Bet1 golgi vesicular membrane trafficking protein [Source:HGNC Symbol;Acc:HGNC:14562]	HGLibA_04430
ENSG00000085978	ATG16L1	2	1.3302	0.48946	0.035522	0.83236	0.62452	0.95326	autophagy related 16 like 1 [Source:HGNC Symbol;Acc:HGNC:21498]	HGLibA_03472;HGLibA_03473
ENSG00000161970	RPL26	1	1.4783	0.31207	0.023436	0.83016	0.75499	0.95728	ribosomal protein L26 [Source:HGNC Symbol;Acc:HGNC:10327]	HGLibA_41913
ENSG00000198535	C2CD4A	3	1.702	0.54609	0.013878	0.82103	0.585	0.95326	C2 calcium dependent domain containing 4A [Source:HGNC Symbol;Acc:HGNC:33627]	HGLibA_06094;HGLibA_06096;HGLibA_06095
ENSG00000157388	CACNA1D	2	2.5918	0.23061	0.0036303	0.81716	0.81762	0.96372	calcium voltage-gated channel subunit alpha1 D [Source:HGNC Symbol;Acc:HGNC:1391]	HGLibA_06883;HGLibA_06885
ENSG00000171530	TBCA	2	1.4669	0.39339	0.024126	0.83016	0.69403	0.95465	tubulin folding cofactor A [Source:HGNC Symbol;Acc:HGNC:11579]	HGLibA_48577;HGLibA_48579
ENSG00000099308	MAST3	3	1.8303	0.43588	0.010753	0.82103	0.66292	0.95326	microtubule associated serine/threonine kinase 3 [Source:HGNC Symbol;Acc:HGNC:19036]	HGLibA_28406;HGLibA_28407;HGLibA_28405
hsa-mir-4512	hsa-mir-4512	3	1.6987	0.45334	0.014016	0.82103	0.65031	0.95326	-	HGLibA_60491;HGLibA_60492;HGLibA_60490
ENSG00000138755	CXCL9	3	1.4623	0.60925	0.024677	0.83016	0.54236	0.95326	C-X-C motif chemokine ligand 9 [Source:HGNC Symbol;Acc:HGNC:7098]	HGLibA_11879;HGLibA_11877;HGLibA_11878
ENSG00000164008	C1orf50	1	1.5963	0.29104	0.017692	0.82766	0.77102	0.95808	chromosome 1 open reading frame 50 [Source:HGNC Symbol;Acc:HGNC:28795]	HGLibA_05860
ENSG00000167526	RPL13	1	1.4955	0.30869	0.022288	0.83016	0.75756	0.95738	ribosomal protein L13 [Source:HGNC Symbol;Acc:HGNC:10303]	HGLibA_41868
ENSG00000126790	L3HYPDH	2	1.2666	0.4967	0.042186	0.84121	0.6194	0.95326	trans-L-3-hydroxyproline dehydratase [Source:HGNC Symbol;Acc:HGNC:20488]	HGLibA_25893;HGLibA_25894
ENSG00000135587	SMPD2	3	1.4566	0.67205	0.025183	0.83016	0.50155	0.95326	sphingomyelin phosphodiesterase 2 [Source:HGNC Symbol;Acc:HGNC:11121]	HGLibA_45806;HGLibA_45807;HGLibA_45805
ENSG00000169764	UGP2	2	3.4872	0.13633	0.0016084	0.78846	0.89156	0.97986	UDP-glucose pyrophosphorylase 2 [Source:HGNC Symbol;Acc:HGNC:12527]	HGLibA_52874;HGLibA_52873
ENSG00000163440	PDCL2	2	1.6727	0.46978	0.014659	0.82103	0.63851	0.95326	phosducin like 2 [Source:HGNC Symbol;Acc:HGNC:29524]	HGLibA_35752;HGLibA_35750
ENSG00000176231	OR10H4	2	2.5586	0.26329	0.003998	0.81716	0.79233	0.9605	olfactory receptor family 10 subfamily H member 4 [Source:HGNC Symbol;Acc:HGNC:15388]	HGLibA_33514;HGLibA_33515
ENSG00000273559	CWC25	1	2.0011	0.22666	0.0083636	0.81716	0.82069	0.96439	CWC25 spliceosome associated protein homolog [Source:HGNC Symbol;Acc:HGNC:25989]	HGLibA_11818
ENSG00000171657	GPR82	3	1.2695	0.71568	0.04191	0.84121	0.47419	0.95326	G protein-coupled receptor 82 [Source:HGNC Symbol;Acc:HGNC:4533]	HGLibA_20089;HGLibA_20090;HGLibA_20088
ENSG00000107819	SFXN3	3	1.2306	0.72962	0.047057	0.85738	0.46562	0.95326	sideroflexin 3 [Source:HGNC Symbol;Acc:HGNC:16087]	HGLibA_43788;HGLibA_43786;HGLibA_43787
ENSG00000169592	INO80E	1	1.9158	0.2396	0.0092827	0.81716	0.81064	0.9631	INO80 complex subunit E [Source:HGNC Symbol;Acc:HGNC:26905]	HGLibA_23331
FAM195A	FAM195A	3	1.3295	0.74339	0.035614	0.83236	0.45725	0.95326	-	HGLibA_16374;HGLibA_16376;HGLibA_16375
hsa-mir-3168	hsa-mir-3168	4	1.7069	0.62175	0.013832	0.82103	0.53411	0.95326	-	HGLibA_58752;HGLibA_58751;HGLibA_58750;HGLibA_58753
ENSG00000159496	RGL4	3	1.6242	0.55673	0.016176	0.82103	0.57771	0.95326	ral guanine nucleotide dissociation stimulator like 4 [Source:HGNC Symbol;Acc:HGNC:31911]	HGLibA_41011;HGLibA_41010;HGLibA_41009
ENSG00000181409	AATK	1	2.4689	0.1677	0.0042277	0.81716	0.86682	0.97464	apoptosis associated tyrosine kinase [Source:HGNC Symbol;Acc:HGNC:21]	HGLibA_00088
ENSG00000076641	PAG1	2	1.8951	0.32032	0.0097882	0.81716	0.74872	0.95697	phosphoprotein membrane anchor with glycosphingolipid microdomains 1 [Source:HGNC Symbol;Acc:HGNC:30043]	HGLibA_35002;HGLibA_35000
ENSG00000117748	RPA2	3	2.9514	0.2591	0.0024355	0.81716	0.79556	0.96136	replication protein A2 [Source:HGNC Symbol;Acc:HGNC:10290]	HGLibA_41803;HGLibA_41801;HGLibA_41802
ENSG00000143418	CERS2	2	2.1783	0.29235	0.0067093	0.81716	0.77002	0.9579	ceramide synthase 2 [Source:HGNC Symbol;Acc:HGNC:14076]	HGLibA_09202;HGLibA_09203
ENSG00000159885	ZNF222	3	1.9948	0.41986	0.0083636	0.81716	0.67459	0.95326	zinc finger protein 222 [Source:HGNC Symbol;Acc:HGNC:13015]	HGLibA_55612;HGLibA_55611;HGLibA_55613
hsa-mir-4747	hsa-mir-4747	3	1.5357	0.66023	0.020128	0.83016	0.5091	0.95326	-	HGLibA_61084;HGLibA_61087;HGLibA_61086
hsa-mir-4744	hsa-mir-4744	4	1.2377	0.63397	0.046138	0.85666	0.5261	0.95326	-	HGLibA_61074;HGLibA_61072;HGLibA_61073;HGLibA_61075
ENSG00000170276	HSPB2	3	1.3751	0.55114	0.03134	0.83236	0.58153	0.95326	heat shock protein family B (small) member 2 [Source:HGNC Symbol;Acc:HGNC:5247]	HGLibA_22307;HGLibA_22308;HGLibA_22306
ENSG00000186526	CYP4F8	3	1.2671	0.59575	0.04214	0.84121	0.55134	0.95326	cytochrome P450 family 4 subfamily F member 8 [Source:HGNC Symbol;Acc:HGNC:2648]	HGLibA_12197;HGLibA_12195;HGLibA_12196
ENSG00000181513	ACBD4	3	1.6176	0.45046	0.016497	0.82103	0.65238	0.95326	acyl-CoA binding domain containing 4 [Source:HGNC Symbol;Acc:HGNC:23337]	HGLibA_00401;HGLibA_00402;HGLibA_00400
ENSG00000149932	TMEM219	1	4.3579	0.045259	0.00036763	0.66667	0.9639	0.99417	transmembrane protein 219 [Source:HGNC Symbol;Acc:HGNC:25201]	HGLibA_50232
ENSG00000145817	YIPF5	3	1.2647	0.67315	0.042461	0.84121	0.50085	0.95326	Yip1 domain family member 5 [Source:HGNC Symbol;Acc:HGNC:24877]	HGLibA_54681;HGLibA_54683;HGLibA_54682
ENSG00000125245	GPR18	2	1.4374	0.39631	0.026469	0.83016	0.69188	0.95446	G protein-coupled receptor 18 [Source:HGNC Symbol;Acc:HGNC:4472]	HGLibA_19981;HGLibA_19980
DUX2	DUX2	1	2.0432	0.22019	0.0080879	0.81716	0.82572	0.96506	-	HGLibA_14202
ENSG00000121957	GPSM2	3	1.2744	0.69308	0.041083	0.83944	0.48826	0.95326	G protein signaling modulator 2 [Source:HGNC Symbol;Acc:HGNC:29501]	HGLibA_20159;HGLibA_20158;HGLibA_20157
ENSG00000165300	SLITRK5	3	1.2471	0.78436	0.044713	0.85276	0.43283	0.95326	SLIT and NTRK like family member 5 [Source:HGNC Symbol;Acc:HGNC:20295]	HGLibA_45525;HGLibA_45526;HGLibA_45524
ENSG00000181074	OR52N4	3	2.1039	0.40238	0.0075824	0.81716	0.6874	0.95405	olfactory receptor family 52 subfamily N member 4 (gene/pseudogene) [Source:HGNC Symbol;Acc:HGNC:15230]	HGLibA_34198;HGLibA_34200;HGLibA_34199
ENSG00000186468	RPS23	2	1.2241	0.57653	0.047976	0.85995	0.56426	0.95326	ribosomal protein S23 [Source:HGNC Symbol;Acc:HGNC:10410]	HGLibA_42111;HGLibA_42109
ENSG00000187026	KRTAP21-2	2	1.2372	0.60467	0.046138	0.85666	0.5454	0.95326	keratin associated protein 21-2 [Source:HGNC Symbol;Acc:HGNC:18946]	HGLibA_25710;HGLibA_25712
hsa-mir-548at	hsa-mir-548at	2	1.3138	0.52263	0.036855	0.83236	0.60123	0.95326	-	HGLibA_61926;HGLibA_61923
ENSG00000245848	CEBPA	2	1.8246	0.33267	0.010845	0.82103	0.73938	0.95685	CCAAT enhancer binding protein alpha [Source:HGNC Symbol;Acc:HGNC:1833]	HGLibA_08955;HGLibA_08957
ENSG00000185774	KCNIP4	3	1.891	0.45943	0.0098341	0.81716	0.64593	0.95326	potassium voltage-gated channel interacting protein 4 [Source:HGNC Symbol;Acc:HGNC:30083]	HGLibA_24243;HGLibA_24244;HGLibA_24242
ENSG00000104671	DCTN6	3	2.6944	0.27706	0.0033546	0.81716	0.78174	0.95938	dynactin subunit 6 [Source:HGNC Symbol;Acc:HGNC:16964]	HGLibA_12558;HGLibA_12560;HGLibA_12559
ENSG00000130164	LDLR	3	1.4701	0.67469	0.023804	0.83016	0.49987	0.95326	low density lipoprotein receptor [Source:HGNC Symbol;Acc:HGNC:6547]	HGLibA_26239;HGLibA_26237;HGLibA_26238
ENSG00000113594	LIFR	2	1.7111	0.46534	0.013556	0.82103	0.64169	0.95326	LIF receptor alpha [Source:HGNC Symbol;Acc:HGNC:6597]	HGLibA_26476;HGLibA_26477
ENSG00000111445	RFC5	2	1.4989	0.42032	0.02215	0.83016	0.67425	0.95326	replication factor C subunit 5 [Source:HGNC Symbol;Acc:HGNC:9973]	HGLibA_40913;HGLibA_40914
ENSG00000163159	VPS72	3	1.2632	0.63045	0.042691	0.84121	0.5284	0.95326	vacuolar protein sorting 72 homolog [Source:HGNC Symbol;Acc:HGNC:11644]	HGLibA_53814;HGLibA_53816;HGLibA_53815
ENSG00000166856	GPR182	3	1.3597	0.67351	0.032673	0.83236	0.50062	0.95326	G protein-coupled receptor 182 [Source:HGNC Symbol;Acc:HGNC:13708]	HGLibA_19987;HGLibA_19986;HGLibA_19988
ENSG00000082516	GEMIN5	2	3.8294	0.10158	0.001011	0.78846	0.91909	0.98575	gem nuclear organelle associated protein 5 [Source:HGNC Symbol;Acc:HGNC:20043]	HGLibA_18918;HGLibA_18916
ENSG00000086102	NFX1	3	1.7927	0.55647	0.011488	0.82103	0.57789	0.95326	X-box binding 1 [Source:HGNC Symbol;Acc:HGNC:7803]"	HGLibA_31848;HGLibA_31849;HGLibA_31847
ENSG00000138674	SEC31A	3	1.5573	0.49484	0.019071	0.82834	0.62071	0.95326	COPII coat complex component [Source:HGNC Symbol;Acc:HGNC:17052]"	HGLibA_43241;HGLibA_43240;HGLibA_43242
ENSG00000205078	SYCE1L	2	1.601	0.48415	0.017462	0.82609	0.62828	0.95326	synaptonemal complex central element protein 1 like [Source:HGNC Symbol;Acc:HGNC:37236]	HGLibA_47917;HGLibA_47916
ENSG00000136636	KCTD3	2	1.9824	0.39385	0.0085934	0.81716	0.69369	0.95464	potassium channel tetramerization domain containing 3 [Source:HGNC Symbol;Acc:HGNC:21305]	HGLibA_24452;HGLibA_24453
ENSG00000134313	KIDINS220	2	1.8829	0.41795	0.009926	0.81716	0.67599	0.95326	kinase D interacting substrate 220 [Source:HGNC Symbol;Acc:HGNC:29508]	HGLibA_24847;HGLibA_24848
ENSG00000133805	AMPD3	3	1.4015	0.56767	0.029548	0.83236	0.57026	0.95326	adenosine monophosphate deaminase 3 [Source:HGNC Symbol;Acc:HGNC:470]	HGLibA_01828;HGLibA_01830;HGLibA_01829
hsa-mir-1322	hsa-mir-1322	1	1.394	0.32911	0.029824	0.83236	0.74207	0.95685	-	HGLibA_57762
ENSG00000177954	RPS27	1	1.573	0.29483	0.018473	0.82766	0.76813	0.95774	ribosomal protein S27 [Source:HGNC Symbol;Acc:HGNC:10416]	HGLibA_42121
ENSG00000143842	SOX13	2	1.4397	0.54986	0.026286	0.83016	0.58241	0.95326	SRY-box 13 [Source:HGNC Symbol;Acc:HGNC:11192]	HGLibA_46236;HGLibA_46238
ENSG00000135750	KCNK1	3	1.2163	0.49531	0.049033	0.86375	0.62038	0.95326	potassium two pore domain channel subfamily K member 1 [Source:HGNC Symbol;Acc:HGNC:6272]	HGLibA_24294;HGLibA_24295;HGLibA_24293
ENSG00000149564	ESAM	1	2.1526	0.20256	0.0071228	0.81716	0.83948	0.96916	endothelial cell adhesion molecule [Source:HGNC Symbol;Acc:HGNC:17474]	HGLibA_15555
ENSG00000115896	PLCL1	1	2.506	0.16089	0.0040899	0.81716	0.87218	0.97606	phospholipase C like 1 (inactive) [Source:HGNC Symbol;Acc:HGNC:9063]	HGLibA_37014
ENSG00000172167	MTBP	2	1.406	0.55195	0.029135	0.83236	0.58098	0.95326	MDM2 binding protein [Source:HGNC Symbol;Acc:HGNC:7417]	HGLibA_30322;HGLibA_30321
ENSG00000109063	MYH3	3	1.4266	0.58901	0.027388	0.83016	0.55586	0.95326	myosin heavy chain 3 [Source:HGNC Symbol;Acc:HGNC:7573]	HGLibA_30723;HGLibA_30725;HGLibA_30724
ENSG00000138448	ITGAV	1	1.4687	0.31506	0.023942	0.83016	0.75272	0.957	integrin subunit alpha V [Source:HGNC Symbol;Acc:HGNC:6150]	HGLibA_23763
ENSG00000128512	DOCK4	2	2.057	0.37148	0.007996	0.81716	0.71028	0.95575	dedicator of cytokinesis 4 [Source:HGNC Symbol;Acc:HGNC:19192]	HGLibA_13747;HGLibA_13746
ENSG00000204909	SPINK9	3	1.5214	0.48643	0.020633	0.83016	0.62667	0.95326	Kazal type 9 [Source:HGNC Symbol;Acc:HGNC:32951]"	HGLibA_46709;HGLibA_46710;HGLibA_46708
ENSG00000134962	KLB	2	1.9187	0.35039	0.0092827	0.81716	0.72604	0.95575	klotho beta [Source:HGNC Symbol;Acc:HGNC:15527]	HGLibA_25023;HGLibA_25025
hsa-mir-3610	hsa-mir-3610	3	1.4671	0.48999	0.024126	0.83016	0.62414	0.95326	-	HGLibA_59073;HGLibA_59074;HGLibA_59072
ENSG00000109705	NKX3-2	3	1.2376	0.69548	0.046138	0.85666	0.48675	0.95326	NK3 homeobox 2 [Source:HGNC Symbol;Acc:HGNC:951]	HGLibA_32051;HGLibA_32053;HGLibA_32052
ENSG00000049768	FOXP3	3	1.4106	0.62038	0.028675	0.83201	0.53501	0.95326	forkhead box P3 [Source:HGNC Symbol;Acc:HGNC:6106]	HGLibA_17935;HGLibA_17937;HGLibA_17936
ENSG00000184254	ALDH1A3	1	3.5576	0.081191	0.0015165	0.78846	0.93529	0.98779	aldehyde dehydrogenase 1 family member A3 [Source:HGNC Symbol;Acc:HGNC:409]	HGLibA_01546
ADSS	ADSS	3	1.3701	0.57242	0.031892	0.83236	0.56704	0.95326	-	HGLibA_01076;HGLibA_01075;HGLibA_01077
ENSG00000183578	TNFAIP8L3	3	2.2329	0.39532	0.0062038	0.81716	0.69261	0.95446	TNF alpha induced protein 8 like 3 [Source:HGNC Symbol;Acc:HGNC:20620]	HGLibA_50729;HGLibA_50731;HGLibA_50730
hsa-mir-4779	hsa-mir-4779	2	1.5068	0.50259	0.021874	0.83016	0.61526	0.95326	-	HGLibA_61224;HGLibA_61223
ENSG00000006756	ARSD	3	1.2875	0.58995	0.039199	0.83236	0.55522	0.95326	arylsulfatase D [Source:HGNC Symbol;Acc:HGNC:717]	HGLibA_03133;HGLibA_03134;HGLibA_03135
ENSG00000103356	EARS2	3	2.1838	0.39742	0.0067093	0.81716	0.69106	0.95446	mitochondrial [Source:HGNC Symbol;Acc:HGNC:29419]"	HGLibA_14344;HGLibA_14342;HGLibA_14343
ENSG00000164038	SLC9B2	3	1.4581	0.58473	0.025045	0.83016	0.55873	0.95326	solute carrier family 9 member B2 [Source:HGNC Symbol;Acc:HGNC:25143]	HGLibA_45435;HGLibA_45436;HGLibA_45437
ENSG00000182952	HMGN4	2	1.5722	0.49085	0.018565	0.82766	0.62354	0.95326	high mobility group nucleosomal binding domain 4 [Source:HGNC Symbol;Acc:HGNC:4989]	HGLibA_21726;HGLibA_21725
ENSG00000181031	RPH3AL	3	1.4145	0.56879	0.028399	0.83201	0.5695	0.95326	rabphilin 3A like (without C2 domains) [Source:HGNC Symbol;Acc:HGNC:10296]	HGLibA_41846;HGLibA_41848;HGLibA_41847
ENSG00000171496	OR1L8	3	1.7417	0.52306	0.012867	0.82103	0.60093	0.95326	olfactory receptor family 1 subfamily L member 8 [Source:HGNC Symbol;Acc:HGNC:15110]	HGLibA_33698;HGLibA_33697;HGLibA_33699
ENSG00000147274	RBMX	3	1.4957	0.61804	0.022288	0.83016	0.53655	0.95326	RNA binding motif protein X-linked [Source:HGNC Symbol;Acc:HGNC:9910]	HGLibA_40595;HGLibA_40596;HGLibA_40597
TCEB2	TCEB2	1	1.7519	0.26381	0.012683	0.82103	0.79193	0.96027	-	HGLibA_48736
ENSG00000084674	APOB	3	1.427	0.58559	0.027388	0.83016	0.55815	0.95326	apolipoprotein B [Source:HGNC Symbol;Acc:HGNC:603]	HGLibA_02464;HGLibA_02463;HGLibA_02465
ENSG00000100170	SLC5A1	3	1.2521	0.48242	0.044116	0.85031	0.62951	0.95326	solute carrier family 5 member 1 [Source:HGNC Symbol;Acc:HGNC:11036]	HGLibA_45254;HGLibA_45253;HGLibA_45252
hsa-mir-4300	hsa-mir-4300	4	1.4086	0.54658	0.028859	0.83201	0.58467	0.95326	-	HGLibA_59911;HGLibA_59912;HGLibA_59913;HGLibA_59910
hsa-mir-3919	hsa-mir-3919	4	1.2378	0.88914	0.046092	0.85666	0.37393	0.95326	-	HGLibA_59554;HGLibA_59551;HGLibA_59552;HGLibA_59553
ENSG00000182541	LIMK2	3	1.3616	0.72782	0.032627	0.83236	0.46672	0.95326	LIM domain kinase 2 [Source:HGNC Symbol;Acc:HGNC:6614]	HGLibA_26544;HGLibA_26542;HGLibA_26543
ENSG00000101146	RAE1	3	1.5362	0.46409	0.020128	0.83016	0.64259	0.95326	ribonucleic acid export 1 [Source:HGNC Symbol;Acc:HGNC:9828]	HGLibA_40116;HGLibA_40117;HGLibA_40118
ENSG00000171574	ZNF584	2	2.2264	0.29757	0.0062497	0.81716	0.76603	0.95774	zinc finger protein 584 [Source:HGNC Symbol;Acc:HGNC:27318]	HGLibA_56317;HGLibA_56316
ENSG00000205784	ARRDC5	3	1.2313	0.79138	0.047011	0.85738	0.42872	0.95326	arrestin domain containing 5 [Source:HGNC Symbol;Acc:HGNC:31407]	HGLibA_03125;HGLibA_03124;HGLibA_03126
ENSG00000182180	MRPS16	1	1.6696	0.27699	0.014705	0.82103	0.78179	0.95938	mitochondrial ribosomal protein S16 [Source:HGNC Symbol;Acc:HGNC:14048]	HGLibA_30044
ATP5B	ATP5B	3	1.8762	0.51767	0.009972	0.81716	0.60469	0.95326	-	HGLibA_03642;HGLibA_03640;HGLibA_03641
ENSG00000110107	PRPF19	2	1.305	0.54641	0.037498	0.83236	0.58478	0.95326	pre-mRNA processing factor 19 [Source:HGNC Symbol;Acc:HGNC:17896]	HGLibA_38714;HGLibA_38713
ENSG00000162461	SLC25A34	3	1.2727	0.63885	0.04145	0.84121	0.52292	0.95326	solute carrier family 25 member 34 [Source:HGNC Symbol;Acc:HGNC:27653]	HGLibA_44715;HGLibA_44714;HGLibA_44713
ENSG00000108510	MED13	2	1.9686	0.37229	0.0086393	0.81716	0.70967	0.95575	mediator complex subunit 13 [Source:HGNC Symbol;Acc:HGNC:22474]	HGLibA_28749;HGLibA_28748
ENSG00000150269	OR5M9	1	2.5548	0.16103	0.003998	0.81716	0.87207	0.97606	olfactory receptor family 5 subfamily M member 9 [Source:HGNC Symbol;Acc:HGNC:15294]	HGLibA_34343
ENSG00000180228	PRKRA	2	1.3365	0.58935	0.034879	0.83236	0.55563	0.95326	protein activator of interferon induced protein kinase EIF2AK2 [Source:HGNC Symbol;Acc:HGNC:9438]	HGLibA_38585;HGLibA_38583
ENSG00000136754	ABI1	2	1.5875	0.47462	0.018106	0.82766	0.63506	0.95326	abl interactor 1 [Source:HGNC Symbol;Acc:HGNC:11320]	HGLibA_00289;HGLibA_00290
ENSG00000148290	SURF1	3	1.3281	0.5949	0.035798	0.83236	0.55191	0.95326	cytochrome c oxidase assembly factor [Source:HGNC Symbol;Acc:HGNC:11474]"	HGLibA_47831;HGLibA_47833;HGLibA_47832
ENSG00000120075	HOXB5	3	1.2307	0.6845	0.047057	0.85738	0.49366	0.95326	homeobox B5 [Source:HGNC Symbol;Acc:HGNC:5116]	HGLibA_21940;HGLibA_21941;HGLibA_21942
ENSG00000136381	IREB2	1	2.7076	0.14551	0.0033546	0.81716	0.88431	0.97846	iron responsive element binding protein 2 [Source:HGNC Symbol;Acc:HGNC:6115]	HGLibA_23568
ENSG00000171960	PPIH	2	2.2516	0.26797	0.0060659	0.81716	0.78872	0.95957	peptidylprolyl isomerase H [Source:HGNC Symbol;Acc:HGNC:14651]	HGLibA_37937;HGLibA_37936
ENSG00000150316	CWC15	3	1.3206	0.62433	0.036395	0.83236	0.53241	0.95326	CWC15 spliceosome associated protein homolog [Source:HGNC Symbol;Acc:HGNC:26939]	HGLibA_11812;HGLibA_11811;HGLibA_11813
ENSG00000169891	REPS2	3	1.3361	0.73978	0.035017	0.83236	0.45943	0.95326	RALBP1 associated Eps domain containing 2 [Source:HGNC Symbol;Acc:HGNC:9963]	HGLibA_40851;HGLibA_40852;HGLibA_40850
ENSG00000178125	PPP1R42	2	1.809	0.40376	0.011167	0.82103	0.68639	0.95405	protein phosphatase 1 regulatory subunit 42 [Source:HGNC Symbol;Acc:HGNC:33732]	HGLibA_38120;HGLibA_38118
ENSG00000120697	ALG5	2	2.6467	0.21474	0.0034465	0.81716	0.82997	0.9664	dolichyl-phosphate beta-glucosyltransferase [Source:HGNC Symbol;Acc:HGNC:20266]"	HGLibA_01631;HGLibA_01630
ENSG00000157184	CPT2	2	1.56	0.38197	0.018933	0.82766	0.70248	0.95516	carnitine palmitoyltransferase 2 [Source:HGNC Symbol;Acc:HGNC:2330]	HGLibA_11026;HGLibA_11027
ENSG00000101782	RIOK3	2	1.7339	0.37791	0.013005	0.82103	0.7055	0.95516	RIO kinase 3 [Source:HGNC Symbol;Acc:HGNC:11451]	HGLibA_41313;HGLibA_41314
ENSG00000166762	CATSPER2	3	1.3275	0.64231	0.035844	0.83236	0.52067	0.95326	cation channel sperm associated 2 [Source:HGNC Symbol;Acc:HGNC:18810]	HGLibA_07357;HGLibA_07359;HGLibA_07358
ENSG00000086504	MRPL28	3	1.404	0.47324	0.029319	0.83236	0.63604	0.95326	mitochondrial ribosomal protein L28 [Source:HGNC Symbol;Acc:HGNC:14484]	HGLibA_29942;HGLibA_29943;HGLibA_29941
ENSG00000185467	KPNA7	3	1.3811	0.65863	0.030835	0.83236	0.51013	0.95326	karyopherin subunit alpha 7 [Source:HGNC Symbol;Acc:HGNC:21839]	HGLibA_25371;HGLibA_25373;HGLibA_25372
ENSG00000111581	NUP107	2	1.2371	0.58731	0.046184	0.85678	0.55699	0.95326	nucleoporin 107 [Source:HGNC Symbol;Acc:HGNC:29914]	HGLibA_33018;HGLibA_33017
ENSG00000168701	TMEM208	2	1.406	0.54173	0.029135	0.83236	0.588	0.95326	transmembrane protein 208 [Source:HGNC Symbol;Acc:HGNC:25015]	HGLibA_50202;HGLibA_50200
ENSG00000072506	HSD17B10	1	1.8895	0.24245	0.0098341	0.81716	0.80843	0.96293	hydroxysteroid 17-beta dehydrogenase 10 [Source:HGNC Symbol;Acc:HGNC:4800]	HGLibA_22173
ENSG00000101310	SEC23B	3	1.2327	0.74752	0.046689	0.85738	0.45475	0.95326	coat complex II component [Source:HGNC Symbol;Acc:HGNC:10702]"	HGLibA_43224;HGLibA_43222;HGLibA_43223
ENSG00000113303	BTNL8	3	1.2513	0.69569	0.044208	0.85057	0.48662	0.95326	butyrophilin like 8 [Source:HGNC Symbol;Acc:HGNC:26131]	HGLibA_04980;HGLibA_04981;HGLibA_04979
ENSG00000070882	OSBPL3	3	1.657	0.53025	0.015165	0.82103	0.59594	0.95326	oxysterol binding protein like 3 [Source:HGNC Symbol;Acc:HGNC:16370]	HGLibA_34655;HGLibA_34654;HGLibA_34656
ENSG00000158813	EDA	2	1.3314	0.4706	0.035292	0.83236	0.63793	0.95326	ectodysplasin A [Source:HGNC Symbol;Acc:HGNC:3157]	HGLibA_14430;HGLibA_14429
ENSG00000141564	RPTOR	3	1.4948	0.50131	0.022288	0.83016	0.61616	0.95326	regulatory associated protein of MTOR complex 1 [Source:HGNC Symbol;Acc:HGNC:30287]	HGLibA_42203;HGLibA_42204;HGLibA_42202
ENSG00000079689	SCGN	3	1.4301	0.59782	0.027021	0.83016	0.54996	0.95326	EF-hand calcium binding protein [Source:HGNC Symbol;Acc:HGNC:16941]"	HGLibA_42937;HGLibA_42939;HGLibA_42938
ENSG00000204308	RNF5	3	1.2998	0.58554	0.037958	0.83236	0.55819	0.95326	ring finger protein 5 [Source:HGNC Symbol;Acc:HGNC:10068]	HGLibA_41691;HGLibA_41692;HGLibA_41690
ENSG00000094880	CDC23	2	1.5078	0.42524	0.021874	0.83016	0.67066	0.95326	cell division cycle 23 [Source:HGNC Symbol;Acc:HGNC:1724]	HGLibA_08523;HGLibA_08525
ENSG00000134056	MRPS36	3	1.4698	0.61105	0.023804	0.83016	0.54117	0.95326	mitochondrial ribosomal protein S36 [Source:HGNC Symbol;Acc:HGNC:16631]	HGLibA_30102;HGLibA_30101;HGLibA_30100
hsa-mir-568	hsa-mir-568	4	1.329	0.70254	0.03566	0.83236	0.48234	0.95326	-	HGLibA_62233;HGLibA_62231;HGLibA_62232;HGLibA_62230
ENSG00000130559	CAMSAP1	1	2.351	0.17799	0.0051009	0.81716	0.85873	0.97287	calmodulin regulated spectrin associated protein 1 [Source:HGNC Symbol;Acc:HGNC:19946]	HGLibA_07105
ENSG00000187172	BAGE2	1	1.9684	0.23171	0.0086393	0.81716	0.81676	0.96372	BAGE family member 2 [Source:HGNC Symbol;Acc:HGNC:15723]	HGLibA_04095
ENSG00000108256	NUFIP2	2	1.7509	0.34661	0.012729	0.82103	0.72889	0.95622	nuclear FMR1 interacting protein 2 [Source:HGNC Symbol;Acc:HGNC:17634]	HGLibA_33004;HGLibA_33002
ENSG00000271303	SRXN1	3	1.2588	0.69121	0.043197	0.84121	0.48943	0.95326	sulfiredoxin 1 [Source:HGNC Symbol;Acc:HGNC:16132]	HGLibA_47095;HGLibA_47097;HGLibA_47096
ENSG00000165202	OR1Q1	3	1.2573	0.6809	0.043334	0.84121	0.49594	0.95326	olfactory receptor family 1 subfamily Q member 1 [Source:HGNC Symbol;Acc:HGNC:8223]	HGLibA_33710;HGLibA_33711;HGLibA_33709
ENSG00000149499	EML3	2	1.4214	0.45616	0.027802	0.83016	0.64828	0.95326	echinoderm microtubule associated protein like 3 [Source:HGNC Symbol;Acc:HGNC:26666]	HGLibA_15062;HGLibA_15061
C2orf44	C2orf44	2	1.7375	0.37036	0.012959	0.82103	0.71111	0.95575	-	HGLibA_06132;HGLibA_06130
ENSG00000103502	CDIPT	2	1.3531	0.38831	0.033087	0.83236	0.69779	0.95516	CDP-diacylglycerol--inositol 3-phosphatidyltransferase [Source:HGNC Symbol;Acc:HGNC:1769]	HGLibA_08717;HGLibA_08715
ENSG00000188729	OSTN	3	1.2413	0.67805	0.045632	0.85666	0.49774	0.95326	osteocrin [Source:HGNC Symbol;Acc:HGNC:29961]	HGLibA_34716;HGLibA_34717;HGLibA_34718
ENSG00000139651	ZNF740	3	1.5239	0.55825	0.020495	0.83016	0.57667	0.95326	zinc finger protein 740 [Source:HGNC Symbol;Acc:HGNC:27465]	HGLibA_56662;HGLibA_56663;HGLibA_56661
ENSG00000175390	EIF3F	3	2.1563	0.3129	0.0071228	0.81716	0.75435	0.95727	eukaryotic translation initiation factor 3 subunit F [Source:HGNC Symbol;Acc:HGNC:3275]	HGLibA_14779;HGLibA_14780;HGLibA_14781
hsa-mir-1243	hsa-mir-1243	4	1.2647	0.75768	0.042461	0.84121	0.44864	0.95326	-	HGLibA_57360;HGLibA_57359;HGLibA_57357;HGLibA_57358
ENSG00000155849	ELMO1	3	1.2393	0.64416	0.045954	0.85666	0.51947	0.95326	engulfment and cell motility 1 [Source:HGNC Symbol;Acc:HGNC:16286]	HGLibA_14930;HGLibA_14929;HGLibA_14931
ENSG00000198331	HYLS1	2	1.3822	0.52006	0.030743	0.83236	0.60302	0.95326	centriolar and ciliogenesis associated [Source:HGNC Symbol;Acc:HGNC:26558]"	HGLibA_22458;HGLibA_22457
hsa-mir-8084	hsa-mir-8084	3	1.4748	0.55941	0.023528	0.83016	0.57588	0.95326	-	HGLibA_64178;HGLibA_64177;HGLibA_64179
ENSG00000127954	STEAP4	3	1.3108	0.70976	0.037269	0.83236	0.47785	0.95326	STEAP4 metalloreductase [Source:HGNC Symbol;Acc:HGNC:21923]	HGLibA_47431;HGLibA_47432;HGLibA_47433
ENSG00000074935	TUBE1	1	4.1334	0.053526	0.00050549	0.6875	0.95731	0.9921	tubulin epsilon 1 [Source:HGNC Symbol;Acc:HGNC:20775]	HGLibA_52301
ENSG00000204511	MCCD1	1	1.4687	0.3142	0.023942	0.83016	0.75337	0.957	mitochondrial coiled-coil domain 1 [Source:HGNC Symbol;Acc:HGNC:20668]	HGLibA_28572
ENSG00000021776	AQR	2	1.7759	0.43031	0.01181	0.82103	0.66697	0.95326	aquarius intron-binding spliceosomal factor [Source:HGNC Symbol;Acc:HGNC:29513]	HGLibA_02617;HGLibA_02616
ENSG00000169385	RNASE2	3	1.5374	0.54393	0.020082	0.83016	0.58649	0.95326	ribonuclease A family member 2 [Source:HGNC Symbol;Acc:HGNC:10045]	HGLibA_41403;HGLibA_41404;HGLibA_41402
ENSG00000135341	MAP3K7	3	1.5395	0.59072	0.019806	0.83016	0.55471	0.95326	mitogen-activated protein kinase kinase kinase 7 [Source:HGNC Symbol;Acc:HGNC:6859]	HGLibA_28196;HGLibA_28195;HGLibA_28197
ENSG00000234776	C11orf94	3	1.5344	0.55663	0.020128	0.83016	0.57778	0.95326	chromosome 11 open reading frame 94 [Source:HGNC Symbol;Acc:HGNC:37213]	HGLibA_05217;HGLibA_05216;HGLibA_05218
ENSG00000167281	RBFOX3	2	1.5969	0.42307	0.0176	0.82766	0.67224	0.95326	RNA binding fox-1 homolog 3 [Source:HGNC Symbol;Acc:HGNC:27097]	HGLibA_40460;HGLibA_40461
ENSG00000125255	SLC10A2	2	1.8354	0.39475	0.010661	0.82103	0.69303	0.95456	solute carrier family 10 member 2 [Source:HGNC Symbol;Acc:HGNC:10906]	HGLibA_44332;HGLibA_44334
ENSG00000148824	MTG1	3	1.4351	0.52245	0.026653	0.83016	0.60136	0.95326	mitochondrial ribosome associated GTPase 1 [Source:HGNC Symbol;Acc:HGNC:32159]	HGLibA_30370;HGLibA_30371;HGLibA_30369
ENSG00000152253	SPC25	3	2.6146	0.26032	0.0034925	0.81716	0.79462	0.961	NDC80 kinetochore complex component [Source:HGNC Symbol;Acc:HGNC:24031]"	HGLibA_46556;HGLibA_46555;HGLibA_46554
WBSCR16	WBSCR16	3	1.9322	0.51197	0.0090988	0.81716	0.60867	0.95326	-	HGLibA_53989;HGLibA_53988;HGLibA_53990
ENSG00000127561	SYNGR3	3	1.4744	0.64845	0.023528	0.83016	0.51669	0.95326	synaptogyrin 3 [Source:HGNC Symbol;Acc:HGNC:11501]	HGLibA_47997;HGLibA_47996;HGLibA_47998
ENSG00000070047	PHRF1	3	1.363	0.69088	0.032535	0.83236	0.48964	0.95326	PHD and ring finger domains 1 [Source:HGNC Symbol;Acc:HGNC:24351]	HGLibA_36459;HGLibA_36458;HGLibA_36460
ENSG00000270757	HSPE1-MOB4	3	1.5834	0.60204	0.018198	0.82766	0.54715	0.95326	HSPE1-MOB4 readthrough [Source:HGNC Symbol;Acc:HGNC:49184]	HGLibA_22336;HGLibA_22338;HGLibA_22337
ENSG00000124479	NDP	3	1.6416	0.59298	0.01567	0.82103	0.55319	0.95326	norrin cystine knot growth factor [Source:HGNC Symbol;Acc:HGNC:7678]"	HGLibA_31409;HGLibA_31411;HGLibA_31410
ENSG00000050327	ARHGEF5	2	1.2704	0.4767	0.041864	0.84121	0.63357	0.95326	Rho guanine nucleotide exchange factor 5 [Source:HGNC Symbol;Acc:HGNC:13209]	HGLibA_02869;HGLibA_02870
ENSG00000163239	TDRD10	3	1.24	0.54086	0.045816	0.85666	0.5886	0.95326	tudor domain containing 10 [Source:HGNC Symbol;Acc:HGNC:25316]	HGLibA_48900;HGLibA_48899;HGLibA_48898
ENSG00000172724	CCL19	2	1.421	0.52444	0.027848	0.83016	0.59997	0.95326	C-C motif chemokine ligand 19 [Source:HGNC Symbol;Acc:HGNC:10617]	HGLibA_07984;HGLibA_07985
ENSG00000114867	EIF4G1	2	2.5454	0.22267	0.0040439	0.81716	0.82379	0.96457	eukaryotic translation initiation factor 4 gamma 1 [Source:HGNC Symbol;Acc:HGNC:3296]	HGLibA_14840;HGLibA_14841
ENSG00000254087	LYN	3	1.4865	0.67586	0.022839	0.83016	0.49913	0.95326	Src family tyrosine kinase [Source:HGNC Symbol;Acc:HGNC:6735]"	HGLibA_27708;HGLibA_27707;HGLibA_27706
ENSG00000157837	SPPL3	3	1.3786	0.7101	0.031019	0.83236	0.47764	0.95326	signal peptide peptidase like 3 [Source:HGNC Symbol;Acc:HGNC:30424]	HGLibA_46784;HGLibA_46785;HGLibA_46783
ENSG00000112130	RNF8	2	2.2874	0.32122	0.0056064	0.81716	0.74804	0.95697	ring finger protein 8 [Source:HGNC Symbol;Acc:HGNC:10071]	HGLibA_41701;HGLibA_41699
ENSG00000113552	GNPDA1	3	1.254	0.644	0.043886	0.84738	0.51957	0.95326	glucosamine-6-phosphate deaminase 1 [Source:HGNC Symbol;Acc:HGNC:4417]	HGLibA_19566;HGLibA_19565;HGLibA_19564
ENSG00000198019	FCGR1B	3	1.6686	0.5303	0.014751	0.82103	0.59591	0.95326	Fc fragment of IgG receptor Ib [Source:HGNC Symbol;Acc:HGNC:3614]	HGLibA_17187;HGLibA_17186;HGLibA_17185
ENSG00000159579	RSPRY1	3	1.4839	0.57434	0.023069	0.83016	0.56574	0.95326	ring finger and SPRY domain containing 1 [Source:HGNC Symbol;Acc:HGNC:29420]	HGLibA_42356;HGLibA_42355;HGLibA_42357
ENSG00000081059	TCF7	3	1.3717	0.58532	0.031662	0.83236	0.55834	0.95326	transcription factor 7 [Source:HGNC Symbol;Acc:HGNC:11639]	HGLibA_48791;HGLibA_48790;HGLibA_48792
ENSG00000074582	BCS1L	2	1.8849	0.36768	0.0098801	0.81716	0.71311	0.95575	ubiquinol-cytochrome c reductase complex chaperone [Source:HGNC Symbol;Acc:HGNC:1020]"	HGLibA_04372;HGLibA_04370
ENSG00000140694	PARN	3	1.3068	0.74735	0.037452	0.83236	0.45485	0.95326	poly(A)-specific ribonuclease [Source:HGNC Symbol;Acc:HGNC:8609]	HGLibA_35208;HGLibA_35209;HGLibA_35207
ENSG00000086475	SEPHS1	3	1.9152	0.42187	0.0093286	0.81716	0.67312	0.95326	selenophosphate synthetase 1 [Source:HGNC Symbol;Acc:HGNC:19685]	HGLibA_43412;HGLibA_43411;HGLibA_43413
FAM194A	FAM194A	3	1.5297	0.59624	0.020312	0.83016	0.55101	0.95326	-	HGLibA_16369;HGLibA_16368;HGLibA_16370
ENSG00000215045	GRID2IP	3	1.7423	0.49568	0.012867	0.82103	0.62012	0.95326	Grid2 interacting protein [Source:HGNC Symbol;Acc:HGNC:18464]	HGLibA_20277;HGLibA_20279;HGLibA_20278

ENSG00000132153	DHX30	3	1.398	0.70282	0.029732	0.83236	0.48217	0.95326	DExH-box helicase 30 [Source:HGNC Symbol;Acc:HGNC:16716]	HGLibA_13174;HGLibA_13176;HGLibA_13175
ENSG00000237763	AMY1A	3	1.486	0.57959	0.022839	0.83016	0.56219	0.95326	alpha 1A (salivary) [Source:HGNC Symbol;Acc:HGNC:474]"	HGLibA_01842;HGLibA_01841;HGLibA_01840
ENSG00000184634	MED12	2	1.4376	0.48727	0.026469	0.83016	0.62606	0.95326	mediator complex subunit 12 [Source:HGNC Symbol;Acc:HGNC:11957]	HGLibA_28741;HGLibA_28743
ENSG00000128731	HERC2	2	1.2391	0.60001	0.045954	0.85666	0.5485	0.95326	HECT and RLD domain containing E3 ubiquitin protein ligase 2 [Source:HGNC Symbol;Acc:HGNC:4868]	HGLibA_21155;HGLibA_21157
ENSG00000157625	TAB3	3	1.6205	0.56767	0.016451	0.82103	0.57026	0.95326	TGF-beta activated kinase 1 (MAP3K7) binding protein 3 [Source:HGNC Symbol;Acc:HGNC:30681]	HGLibA_48147;HGLibA_48148;HGLibA_48146
ENSG00000101057	MYBL2	2	1.2946	0.45782	0.038555	0.83236	0.64708	0.95326	MYB proto-oncogene like 2 [Source:HGNC Symbol;Acc:HGNC:7548]	HGLibA_30646;HGLibA_30645
ENSG00000227345	PARG	3	1.2146	0.62612	0.049262	0.86375	0.53124	0.95326	poly(ADP-ribose) glycohydrolase [Source:HGNC Symbol;Acc:HGNC:8605]	HGLibA_35193;HGLibA_35192;HGLibA_35194
ENSG00000287151	C2orf27A	1	1.2717	0.35343	0.04168	0.84121	0.72377	0.95575	-	HGLibA_06116
hsa-mir-3929	hsa-mir-3929	2	1.4969	0.41439	0.022196	0.83016	0.67859	0.95326	-	HGLibA_59592;HGLibA_59591
ENSG00000088205	DDX18	2	1.2217	0.60713	0.048251	0.85995	0.54377	0.95326	DEAD-box helicase 18 [Source:HGNC Symbol;Acc:HGNC:2741]	HGLibA_12661;HGLibA_12659
ENSG00000131503	ANKHD1	2	1.5173	0.51047	0.021047	0.83016	0.60972	0.95326	ankyrin repeat and KH domain containing 1 [Source:HGNC Symbol;Acc:HGNC:24714]	HGLibA_01965;HGLibA_01963
hsa-mir-6768	hsa-mir-6768	3	1.2165	0.56674	0.049033	0.86375	0.57089	0.95326	-	HGLibA_63235;HGLibA_63233;HGLibA_63232
ENSG00000178171	AMER3	2	1.4772	0.53919	0.023436	0.83016	0.58975	0.95326	APC membrane recruitment protein 3 [Source:HGNC Symbol;Acc:HGNC:26771]	HGLibA_01778;HGLibA_01779
ENSG00000244607	CCDC13	3	1.325	0.6314	0.036028	0.83236	0.52778	0.95326	coiled-coil domain containing 13 [Source:HGNC Symbol;Acc:HGNC:26358]	HGLibA_07590;HGLibA_07589;HGLibA_07588
ENSG00000125966	MMP24	2	1.8845	0.36394	0.009926	0.81716	0.7159	0.95575	matrix metallopeptidase 24 [Source:HGNC Symbol;Acc:HGNC:7172]	HGLibA_29520;HGLibA_29518
ENSG00000104998	IL27RA	2	1.2132	0.59395	0.04963	0.86375	0.55254	0.95326	interleukin 27 receptor subunit alpha [Source:HGNC Symbol;Acc:HGNC:17290]	HGLibA_23116;HGLibA_23114
ENSG00000100749	VRK1	2	2.1731	0.31178	0.0068471	0.81716	0.75521	0.95728	VRK serine/threonine kinase 1 [Source:HGNC Symbol;Acc:HGNC:12718]	HGLibA_53825;HGLibA_53824
ENSG00000215021	PHB2	1	1.5208	0.3045	0.020633	0.83016	0.76075	0.95744	prohibitin 2 [Source:HGNC Symbol;Acc:HGNC:30306]	HGLibA_36315
NBPF16	NBPF16	1	3.865	0.065153	0.00091908	0.78846	0.94805	0.9901	-	HGLibA_31227
ENSG00000149428	HYOU1	3	1.4323	0.68363	0.026883	0.83016	0.49421	0.95326	hypoxia up-regulated 1 [Source:HGNC Symbol;Acc:HGNC:16931]	HGLibA_22461;HGLibA_22460;HGLibA_22462
ENSG00000198015	MRPL42	1	1.2896	0.34986	0.038969	0.83236	0.72645	0.95576	mitochondrial ribosomal protein L42 [Source:HGNC Symbol;Acc:HGNC:14493]	HGLibA_29985
ENSG00000149231	CCDC82	1	1.6593	0.27907	0.015165	0.82103	0.78019	0.95938	coiled-coil domain containing 82 [Source:HGNC Symbol;Acc:HGNC:26282]	HGLibA_07882
ENSG00000184900	SUMO3	2	2.1168	0.36061	0.0075364	0.81716	0.71839	0.95575	small ubiquitin-like modifier 3 [Source:HGNC Symbol;Acc:HGNC:11124]	HGLibA_47783;HGLibA_47782
ENSG00000172977	KAT5	2	2.2439	0.29822	0.0060659	0.81716	0.76554	0.95774	lysine acetyltransferase 5 [Source:HGNC Symbol;Acc:HGNC:5275]	HGLibA_24056;HGLibA_24058
ENSG00000131738	KRT33B	2	1.3017	0.46783	0.037774	0.83236	0.63991	0.95326	keratin 33B [Source:HGNC Symbol;Acc:HGNC:6451]	HGLibA_25484;HGLibA_25482
ENSG00000205754	SLCO1B7	2	2.0395	0.31882	0.0080879	0.81716	0.74986	0.95697	solute carrier organic anion transporter family member 1B7 (putative) [Source:HGNC Symbol;Acc:HGNC:32934]	HGLibA_45455;HGLibA_45454
ENSG00000127481	UBR4	2	1.3874	0.56164	0.030192	0.83236	0.57436	0.95326	ubiquitin protein ligase E3 component n-recognin 4 [Source:HGNC Symbol;Acc:HGNC:30313]	HGLibA_52757;HGLibA_52756
ENSG00000158321	AUTS2	3	1.5794	0.62743	0.018336	0.82766	0.53038	0.95326	activator of transcription and developmental regulator [Source:HGNC Symbol;Acc:HGNC:14262]"	HGLibA_03886;HGLibA_03884;HGLibA_03885
ENSG00000198736	MSRB1	2	2.377	0.25277	0.005009	0.81716	0.80045	0.96142	methionine sulfoxide reductase B1 [Source:HGNC Symbol;Acc:HGNC:14133]	HGLibA_30241;HGLibA_30240
ENSG00000183978	COA3	2	1.8826	0.3318	0.009972	0.81716	0.74004	0.95685	cytochrome c oxidase assembly factor 3 [Source:HGNC Symbol;Acc:HGNC:24990]	HGLibA_10447;HGLibA_10446
ENSG00000110881	ASIC1	3	1.8203	0.50165	0.010845	0.82103	0.61591	0.95326	acid sensing ion channel subunit 1 [Source:HGNC Symbol;Acc:HGNC:100]	HGLibA_03300;HGLibA_03298;HGLibA_03299
ENSG00000167548	KMT2D	3	1.2187	0.69201	0.048665	0.86282	0.48893	0.95326	lysine methyltransferase 2D [Source:HGNC Symbol;Acc:HGNC:7133]	HGLibA_25331;HGLibA_25329;HGLibA_25330
ENSG00000136531	SCN2A	3	1.279	0.64603	0.040531	0.83741	0.51826	0.95326	sodium voltage-gated channel alpha subunit 2 [Source:HGNC Symbol;Acc:HGNC:10588]	HGLibA_42981;HGLibA_42980;HGLibA_42979
ENSG00000165288	BRWD3	2	1.4365	0.49568	0.026515	0.83016	0.62012	0.95326	bromodomain and WD repeat domain containing 3 [Source:HGNC Symbol;Acc:HGNC:17342]	HGLibA_04857;HGLibA_04856
ENSG00000125868	DSTN	3	1.2914	0.52688	0.038877	0.83236	0.59827	0.95326	actin depolymerizing factor [Source:HGNC Symbol;Acc:HGNC:15750]"	HGLibA_14046;HGLibA_14047;HGLibA_14045
ENSG00000155256	ZFYVE27	2	1.6546	0.40575	0.015257	0.82103	0.68493	0.95405	zinc finger FYVE-type containing 27 [Source:HGNC Symbol;Acc:HGNC:26559]	HGLibA_55287;HGLibA_55288
ENSG00000143001	TMEM61	1	2.3189	0.18119	0.0054225	0.81716	0.85622	0.97272	transmembrane protein 61 [Source:HGNC Symbol;Acc:HGNC:27296]	HGLibA_50469
ENSG00000116922	C1orf109	1	1.6621	0.27889	0.015119	0.82103	0.78033	0.95938	chromosome 1 open reading frame 109 [Source:HGNC Symbol;Acc:HGNC:26039]	HGLibA_05740
TCTE3	TCTE3	3	1.311	0.75285	0.037269	0.83236	0.45154	0.95326	-	HGLibA_48861;HGLibA_48859;HGLibA_48860
ENSG00000137055	PLAA	3	1.7511	0.57834	0.012729	0.82103	0.56304	0.95326	phospholipase A2 activating protein [Source:HGNC Symbol;Acc:HGNC:9043]	HGLibA_36930;HGLibA_36929;HGLibA_36931
ENSG00000139318	DUSP6	3	1.2891	0.74049	0.039061	0.83236	0.4559	0.95326	dual specificity phosphatase 6 [Source:HGNC Symbol;Acc:HGNC:3072]	HGLibA_14188;HGLibA_14186;HGLibA_14187
ENSG00000137275	RIPK1	3	1.3294	0.71877	0.035614	0.83236	0.47228	0.95326	receptor interacting serine/threonine kinase 1 [Source:HGNC Symbol;Acc:HGNC:10019]	HGLibA_41315;HGLibA_41317;HGLibA_41316
ENSG00000132970	WASF3	2	1.4358	0.49702	0.026653	0.83016	0.61917	0.95326	WAS protein family member 3 [Source:HGNC Symbol;Acc:HGNC:12734]	HGLibA_53960;HGLibA_53959
ENSG00000116266	STXBP3	2	1.3333	0.4056	0.035201	0.83236	0.68504	0.95405	syntaxin binding protein 3 [Source:HGNC Symbol;Acc:HGNC:11446]	HGLibA_47670;HGLibA_47669
ENSG00000030110	BAK1	3	1.3434	0.65538	0.034144	0.83236	0.51222	0.95326	BCL2 antagonist/killer 1 [Source:HGNC Symbol;Acc:HGNC:949]	HGLibA_04134;HGLibA_04133;HGLibA_04135
ENSG00000113013	HSPA9	3	1.6096	0.51431	0.016865	0.82103	0.60704	0.95326	heat shock protein family A (Hsp70) member 9 [Source:HGNC Symbol;Acc:HGNC:5244]	HGLibA_22299;HGLibA_22298;HGLibA_22297
ENSG00000144381	HSPD1	3	1.2837	0.63919	0.039842	0.83524	0.5227	0.95326	heat shock protein family D (Hsp60) member 1 [Source:HGNC Symbol;Acc:HGNC:5261]	HGLibA_22332;HGLibA_22331;HGLibA_22330
ENSG00000007392	LUC7L	1	1.3488	0.33722	0.033546	0.83236	0.73595	0.95685	LUC7 like [Source:HGNC Symbol;Acc:HGNC:6723]	HGLibA_27619
ENSG00000165819	METTL3	3	2.0095	0.46591	0.0083636	0.81716	0.64128	0.95326	methyltransferase like 3 [Source:HGNC Symbol;Acc:HGNC:17563]	HGLibA_29007;HGLibA_29005;HGLibA_29006
hsa-mir-5583-1	hsa-mir-5583-1	2	1.466	0.45685	0.024172	0.83016	0.64778	0.95326	-	HGLibA_62170;HGLibA_62168
hsa-mir-4468	hsa-mir-4468	3	1.8196	0.49373	0.010845	0.82103	0.6215	0.95326	-	HGLibA_60279;HGLibA_60280;HGLibA_60277
ENSG00000052850	ALX4	2	2.2251	0.29842	0.0062497	0.81716	0.76538	0.95774	ALX homeobox 4 [Source:HGNC Symbol;Acc:HGNC:450]	HGLibA_01739;HGLibA_01738
ENSG00000168970	JMJD7-PLA2G4B	2	1.2384	0.42169	0.046092	0.85666	0.67325	0.95326	JMJD7-PLA2G4B readthrough [Source:HGNC Symbol;Acc:HGNC:34449]	HGLibA_23963;HGLibA_23965
ENSG00000204941	PSG5	3	1.33	0.73368	0.035522	0.83236	0.46314	0.95326	pregnancy specific beta-1-glycoprotein 5 [Source:HGNC Symbol;Acc:HGNC:9522]	HGLibA_39049;HGLibA_39050;HGLibA_39048
ENSG00000168807	SNTB2	3	1.844	0.48474	0.010569	0.82103	0.62786	0.95326	syntrophin beta 2 [Source:HGNC Symbol;Acc:HGNC:11169]	HGLibA_46002;HGLibA_46003;HGLibA_46004
NPIPL3	NPIPL3	2	1.5438	0.47438	0.019484	0.83016	0.63523	0.95326	-	HGLibA_32477;HGLibA_32479
FAM57B	FAM57B	1	1.4279	0.32167	0.027205	0.83016	0.7477	0.95697	-	HGLibA_16648
ENSG00000123405	NFE2	2	1.4923	0.37752	0.022563	0.83016	0.70578	0.95516	erythroid 2 [Source:HGNC Symbol;Acc:HGNC:7780]"	HGLibA_31787;HGLibA_31788
ENSG00000135437	RDH5	3	1.2827	0.56968	0.040026	0.83524	0.56889	0.95326	retinol dehydrogenase 5 [Source:HGNC Symbol;Acc:HGNC:9940]	HGLibA_40749;HGLibA_40748;HGLibA_40750
ENSG00000176920	FUT2	3	1.2208	0.69268	0.048251	0.85995	0.48851	0.95326	fucosyltransferase 2 [Source:HGNC Symbol;Acc:HGNC:4013]	HGLibA_18188;HGLibA_18189;HGLibA_18187
ENSG00000186432	KPNA4	3	1.3973	0.63086	0.029778	0.83236	0.52813	0.95326	karyopherin subunit alpha 4 [Source:HGNC Symbol;Acc:HGNC:6397]	HGLibA_25363;HGLibA_25364;HGLibA_25362
hsa-mir-6833	hsa-mir-6833	3	1.2302	0.67533	0.047149	0.85738	0.49947	0.95326	-	HGLibA_63511;HGLibA_63508;HGLibA_63509
ENSG00000125046	SSUH2	3	1.2328	0.5762	0.046689	0.85738	0.56448	0.95326	ssu-2 homolog (C. elegans) [Source:HGNC Symbol;Acc:HGNC:24809]	HGLibA_47193;HGLibA_47191;HGLibA_47192
ENSG00000137077	CCL21	3	1.3272	0.66076	0.035844	0.83236	0.50876	0.95326	C-C motif chemokine ligand 21 [Source:HGNC Symbol;Acc:HGNC:10620]	HGLibA_07993;HGLibA_07994;HGLibA_07995
ENSG00000196715	VKORC1L1	3	1.4619	0.65657	0.024769	0.83016	0.51146	0.95326	vitamin K epoxide reductase complex subunit 1 like 1 [Source:HGNC Symbol;Acc:HGNC:21492]	HGLibA_53680;HGLibA_53681;HGLibA_53679
LOC554223	LOC554223	2	1.5776	0.49335	0.018336	0.82766	0.62177	0.95326	-	HGLibA_26954;HGLibA_26955
ENSG00000275832	ARHGAP23	3	1.2324	0.64448	0.046827	0.85738	0.51927	0.95326	Rho GTPase activating protein 23 [Source:HGNC Symbol;Acc:HGNC:29293]	HGLibA_02733;HGLibA_02731;HGLibA_02732
ENSG00000175198	PCCA	2	1.2729	0.55251	0.041312	0.84121	0.5806	0.95326	propionyl-CoA carboxylase subunit alpha [Source:HGNC Symbol;Acc:HGNC:8653]	HGLibA_35378;HGLibA_35380
ENSG00000196363	WDR5	2	1.3311	0.50799	0.03543	0.83236	0.61146	0.95326	WD repeat domain 5 [Source:HGNC Symbol;Acc:HGNC:12757]	HGLibA_54114;HGLibA_54115
ENSG00000133704	IPO8	2	1.5496	0.43956	0.019209	0.83016	0.66026	0.95326	importin 8 [Source:HGNC Symbol;Acc:HGNC:9853]	HGLibA_23475;HGLibA_23474
ENSG00000162897	FCAMR	3	1.2444	0.79081	0.045173	0.85627	0.42906	0.95326	Fc fragment of IgA and IgM receptor [Source:HGNC Symbol;Acc:HGNC:24692]	HGLibA_17163;HGLibA_17161;HGLibA_17162
ENSG00000161013	MGAT4B	3	1.3529	0.60981	0.033087	0.83236	0.54199	0.95326	3-mannosyl-glycoprotein 4-beta-N-acetylglucosaminyltransferase B [Source:HGNC Symbol;Acc:HGNC:7048]"	HGLibA_29143;HGLibA_29145;HGLibA_29144
ENSG00000156049	GNA14	3	1.4791	0.59959	0.023436	0.83016	0.54878	0.95326	G protein subunit alpha 14 [Source:HGNC Symbol;Acc:HGNC:4382]	HGLibA_19446;HGLibA_19445;HGLibA_19444
ENSG00000115233	PSMD14	2	2.6088	0.2613	0.0035384	0.81716	0.79386	0.96079	non-ATPase 14 [Source:HGNC Symbol;Acc:HGNC:16889]"	HGLibA_39163;HGLibA_39162
ENSG00000205832	C16orf96	1	1.5288	0.30152	0.020312	0.83016	0.76302	0.95769	chromosome 16 open reading frame 96 [Source:HGNC Symbol;Acc:HGNC:40031]	HGLibA_05511
ENSG00000233954	UQCRHL	2	1.2142	0.61204	0.049446	0.86375	0.54051	0.95326	ubiquinol-cytochrome c reductase hinge protein like [Source:HGNC Symbol;Acc:HGNC:51714]	HGLibA_53126;HGLibA_53125
ENSG00000186010	NDUFA13	2	1.674	0.42362	0.014659	0.82103	0.67185	0.95326	NADH:ubiquinone oxidoreductase subunit A13 [Source:HGNC Symbol;Acc:HGNC:17194]	HGLibA_31448;HGLibA_31449
ENSG00000178741	COX5A	1	1.2414	0.36091	0.045586	0.85666	0.71816	0.95575	cytochrome c oxidase subunit 5A [Source:HGNC Symbol;Acc:HGNC:2267]	HGLibA_10835
ENSG00000065243	PKN2	3	1.2447	0.58322	0.045173	0.85627	0.55974	0.95326	protein kinase N2 [Source:HGNC Symbol;Acc:HGNC:9406]	HGLibA_36840;HGLibA_36839;HGLibA_36841
hsa-mir-4719	hsa-mir-4719	1	1.3094	0.34706	0.037269	0.83236	0.72854	0.95617	-	HGLibA_60975
ENSG00000167196	FBXO22	3	1.8706	0.53627	0.010064	0.81716	0.59177	0.95326	F-box protein 22 [Source:HGNC Symbol;Acc:HGNC:13593]	HGLibA_17047;HGLibA_17048;HGLibA_17049
ENSG00000165732	DDX21	3	1.4419	0.46881	0.02624	0.83016	0.6392	0.95326	DExD-box helicase 21 [Source:HGNC Symbol;Acc:HGNC:2744]	HGLibA_12671;HGLibA_12673;HGLibA_12672
ENSG00000116560	SFPQ	1	3.1557	0.10744	0.002022	0.79661	0.91444	0.98413	splicing factor proline and glutamine rich [Source:HGNC Symbol;Acc:HGNC:10774]	HGLibA_43731
ENSG00000169031	COL4A3	3	1.3386	0.66638	0.034695	0.83236	0.50517	0.95326	collagen type IV alpha 3 chain [Source:HGNC Symbol;Acc:HGNC:2204]	HGLibA_10573;HGLibA_10572;HGLibA_10571
ENSG00000152620	NADK2	2	1.7889	0.43105	0.011672	0.82103	0.66643	0.95326	mitochondrial [Source:HGNC Symbol;Acc:HGNC:26404]"	HGLibA_31048;HGLibA_31047
ENSG00000158769	F11R	3	1.2378	0.53821	0.046092	0.85666	0.59043	0.95326	F11 receptor [Source:HGNC Symbol;Acc:HGNC:14685]	HGLibA_15848;HGLibA_15847;HGLibA_15846
ENSG00000054277	OPN3	3	1.8821	0.38684	0.009972	0.81716	0.69888	0.95516	opsin 3 [Source:HGNC Symbol;Acc:HGNC:14007]	HGLibA_33434;HGLibA_33435;HGLibA_33433
ENSG00000024048	UBR2	3	1.2304	0.69553	0.047103	0.85738	0.48673	0.95326	ubiquitin protein ligase E3 component n-recognin 2 [Source:HGNC Symbol;Acc:HGNC:21289]	HGLibA_52750;HGLibA_52751;HGLibA_52749
ENSG00000104299	INTS9	1	3.929	0.062194	0.00082717	0.78846	0.95041	0.99063	integrator complex subunit 9 [Source:HGNC Symbol;Acc:HGNC:25592]	HGLibA_23435
hsa-mir-3668	hsa-mir-3668	1	1.8321	0.25204	0.010661	0.82103	0.80101	0.96142	-	HGLibA_59222
ENSG00000048162	NOP16	2	2.2187	0.33161	0.0063416	0.81716	0.74018	0.95685	NOP16 nucleolar protein [Source:HGNC Symbol;Acc:HGNC:26934]	HGLibA_32308;HGLibA_32306
ENSG00000118200	CAMSAP2	2	1.4656	0.49905	0.024172	0.83016	0.61775	0.95326	calmodulin regulated spectrin associated protein family member 2 [Source:HGNC Symbol;Acc:HGNC:29188]	HGLibA_07110;HGLibA_07109
ENSG00000244045	TMEM199	1	2.5997	0.15032	0.0036303	0.81716	0.88051	0.97769	transmembrane protein 199 [Source:HGNC Symbol;Acc:HGNC:18085]	HGLibA_50164
ENSG00000150275	PCDH15	2	1.4518	0.49016	0.025504	0.83016	0.62402	0.95326	protocadherin related 15 [Source:HGNC Symbol;Acc:HGNC:14674]	HGLibA_35399;HGLibA_35401
ENSG00000151632	AKR1C2	3	1.23	0.61338	0.047149	0.85738	0.53962	0.95326	aldo-keto reductase family 1 member C2 [Source:HGNC Symbol;Acc:HGNC:385]	HGLibA_01484;HGLibA_01485;HGLibA_01483
ENSG00000100811	YY1	3	2.1471	0.42517	0.0071688	0.81716	0.67071	0.95326	YY1 transcription factor [Source:HGNC Symbol;Acc:HGNC:12856]	HGLibA_54758;HGLibA_54756;HGLibA_54757
ENSG00000109323	MANBA	3	1.4054	0.66265	0.029227	0.83236	0.50756	0.95326	mannosidase beta [Source:HGNC Symbol;Acc:HGNC:6831]	HGLibA_28086;HGLibA_28085;HGLibA_28084
LOC100505478	LOC100505478	2	2.1443	0.3234	0.0071688	0.81716	0.74639	0.95685	-	HGLibA_26846;HGLibA_26845
ENSG00000003509	NDUFAF7	2	3.7406	0.12217	0.0012408	0.78846	0.90276	0.98123	NADH:ubiquinone oxidoreductase complex assembly factor 7 [Source:HGNC Symbol;Acc:HGNC:28816]	HGLibA_31499;HGLibA_31500
ENSG00000116580	GON4L	3	1.3422	0.66386	0.034327	0.83236	0.50678	0.95326	gon-4 like [Source:HGNC Symbol;Acc:HGNC:25973]	HGLibA_19668;HGLibA_19670;HGLibA_19669
ENSG00000131480	AOC2	3	1.5398	0.54721	0.019714	0.83016	0.58423	0.95326	copper containing 2 [Source:HGNC Symbol;Acc:HGNC:549]"	HGLibA_02269;HGLibA_02271;HGLibA_02270
hsa-mir-5100	hsa-mir-5100	4	1.3694	0.83631	0.031984	0.83236	0.40298	0.95326	-	HGLibA_61550;HGLibA_61549;HGLibA_61551;HGLibA_61552
ENSG00000096872	IFT74	3	1.6695	0.51647	0.014705	0.82103	0.60553	0.95326	intraflagellar transport 74 [Source:HGNC Symbol;Acc:HGNC:21424]	HGLibA_22769;HGLibA_22770;HGLibA_22771
ENSG00000119203	CPSF3	1	3.6159	0.077902	0.0013786	0.78846	0.93791	0.98838	cleavage and polyadenylation specific factor 3 [Source:HGNC Symbol;Acc:HGNC:2326]	HGLibA_11000
WAPAL	WAPAL	2	1.5844	0.41988	0.018152	0.82766	0.67457	0.95326	-	HGLibA_53942;HGLibA_53941
ENSG00000130508	PXDN	3	1.3223	0.66574	0.036258	0.83236	0.50557	0.95326	peroxidasin [Source:HGNC Symbol;Acc:HGNC:14966]	HGLibA_39650;HGLibA_39648;HGLibA_39649
SRPR	SRPR	2	2.0443	0.36697	0.0080419	0.81716	0.71364	0.95575	-	HGLibA_47026;HGLibA_47027
ENSG00000204979	MS4A13	3	1.7632	0.54037	0.012178	0.82103	0.58894	0.95326	membrane spanning 4-domains A13 [Source:HGNC Symbol;Acc:HGNC:16674]	HGLibA_30136;HGLibA_30137;HGLibA_30135
ENSG00000186810	CXCR3	3	1.3029	0.59575	0.037636	0.83236	0.55134	0.95326	C-X-C motif chemokine receptor 3 [Source:HGNC Symbol;Acc:HGNC:4540]	HGLibA_11887;HGLibA_11888;HGLibA_11886
LOC440563	LOC440563	3	1.2656	0.65818	0.042323	0.84121	0.51043	0.95326	-	HGLibA_26948;HGLibA_26949;HGLibA_26947
ENSG00000100714	MTHFD1	2	1.3906	0.39162	0.030054	0.83236	0.69534	0.95506	cyclohydrolase and formyltetrahydrofolate synthetase 1 [Source:HGNC Symbol;Acc:HGNC:7432]"	HGLibA_30376;HGLibA_30377
ENSG00000144488	ESPNL	3	1.4124	0.52057	0.028537	0.83201	0.60267	0.95326	espin like [Source:HGNC Symbol;Acc:HGNC:27937]	HGLibA_15579;HGLibA_15577;HGLibA_15578
ENSG00000149923	PPP4C	2	1.2823	0.47355	0.040118	0.83524	0.63582	0.95326	protein phosphatase 4 catalytic subunit [Source:HGNC Symbol;Acc:HGNC:9319]	HGLibA_38200;HGLibA_38201
ENSG00000197745	SCGB1D4	1	1.4409	0.31934	0.026286	0.83016	0.74947	0.95697	secretoglobin family 1D member 4 [Source:HGNC Symbol;Acc:HGNC:31748]	HGLibA_42920
ENSG00000053371	AKR7A2	2	1.3091	0.40782	0.037314	0.83236	0.68341	0.9535	aldo-keto reductase family 7 member A2 [Source:HGNC Symbol;Acc:HGNC:389]	HGLibA_01498;HGLibA_01500
ENSG00000147536	GINS4	2	1.5583	0.48948	0.019025	0.828	0.6245	0.95326	GINS complex subunit 4 [Source:HGNC Symbol;Acc:HGNC:28226]	HGLibA_19111;HGLibA_19113
ENSG00000127125	PPCS	1	3.4956	0.084821	0.0015624	0.78846	0.9324	0.98697	phosphopantothenoylcysteine synthetase [Source:HGNC Symbol;Acc:HGNC:25686]	HGLibA_37862
hsa-mir-548al	hsa-mir-548al	1	1.974	0.22812	0.0086393	0.81716	0.81955	0.96415	-	HGLibA_61899
ENSG00000074657	ZNF532	3	1.6315	0.46438	0.015992	0.82103	0.64237	0.95326	zinc finger protein 532 [Source:HGNC Symbol;Acc:HGNC:30940]	HGLibA_56178;HGLibA_56179;HGLibA_56180
ENSG00000044574	HSPA5	3	1.2669	0.65079	0.04214	0.84121	0.51518	0.95326	heat shock protein family A (Hsp70) member 5 [Source:HGNC Symbol;Acc:HGNC:5238]	HGLibA_22290;HGLibA_22288;HGLibA_22289
ENSG00000125447	GGA3	1	1.319	0.34322	0.036579	0.83236	0.73143	0.95648	ARF binding protein 3 [Source:HGNC Symbol;Acc:HGNC:17079]"	HGLibA_18985
ENSG00000212935	KRTAP10-3	3	1.7417	0.51777	0.012867	0.82103	0.60462	0.95326	keratin associated protein 10-3 [Source:HGNC Symbol;Acc:HGNC:22968]	HGLibA_25606;HGLibA_25605;HGLibA_25607
ENSG00000136937	NCBP1	1	1.2396	0.35988	0.045954	0.85666	0.71893	0.95575	nuclear cap binding protein subunit 1 [Source:HGNC Symbol;Acc:HGNC:7658]	HGLibA_31282
ENSG00000249693	THEGL	3	1.2387	0.57576	0.046092	0.85666	0.56478	0.95326	theg spermatid protein like [Source:HGNC Symbol;Acc:HGNC:43771]	HGLibA_49338;HGLibA_49337;HGLibA_49336
QTRTD1	QTRTD1	3	1.5264	0.64067	0.020403	0.83016	0.52173	0.95326	-	HGLibA_39762;HGLibA_39763;HGLibA_39764
ENSG00000221983	UBA52	1	1.3509	0.33892	0.033316	0.83236	0.73467	0.95685	ubiquitin A-52 residue ribosomal protein fusion product 1 [Source:HGNC Symbol;Acc:HGNC:12458]	HGLibA_52526
ENSG00000260238	PMF1-BGLAP	3	1.293	0.72471	0.038739	0.83236	0.46863	0.95326	PMF1-BGLAP readthrough [Source:HGNC Symbol;Acc:HGNC:42953]	HGLibA_37306;HGLibA_37307;HGLibA_37305
ENSG00000099994	SUSD2	2	1.3051	0.59922	0.037498	0.83236	0.54903	0.95326	sushi domain containing 2 [Source:HGNC Symbol;Acc:HGNC:30667]	HGLibA_47846;HGLibA_47847
ENSG00000178075	GRAMD1C	2	1.5456	0.51541	0.019484	0.83016	0.60626	0.95326	GRAM domain containing 1C [Source:HGNC Symbol;Acc:HGNC:25252]	HGLibA_20199;HGLibA_20201
ENSG00000100104	SRRD	2	1.3086	0.54846	0.037314	0.83236	0.58337	0.95326	SRR1 domain containing [Source:HGNC Symbol;Acc:HGNC:33910]	HGLibA_47041;HGLibA_47043
ENSG00000138629	UBL7	2	1.4644	0.43926	0.024401	0.83016	0.66047	0.95326	ubiquitin like 7 [Source:HGNC Symbol;Acc:HGNC:28221]	HGLibA_52715;HGLibA_52713
hsa-mir-665	hsa-mir-665	2	1.6507	0.45882	0.01544	0.82103	0.64636	0.95326	-	HGLibA_63001;HGLibA_63003
ENSG00000049618	ARID1B	3	1.2451	0.6078	0.045035	0.8559	0.54332	0.95326	AT-rich interaction domain 1B [Source:HGNC Symbol;Acc:HGNC:18040]	HGLibA_02884;HGLibA_02885;HGLibA_02886
ENSG00000105419	MEIS3	2	1.2393	0.50953	0.045954	0.85666	0.61038	0.95326	Meis homeobox 3 [Source:HGNC Symbol;Acc:HGNC:29537]	HGLibA_28876;HGLibA_28877
ENSG00000090060	PAPOLA	3	1.2136	0.80215	0.049538	0.86375	0.42246	0.95326	poly(A) polymerase alpha [Source:HGNC Symbol;Acc:HGNC:14981]	HGLibA_35135;HGLibA_35136;HGLibA_35137
ENSG00000129007	CALML4	3	1.3166	0.67276	0.036671	0.83236	0.5011	0.95326	calmodulin like 4 [Source:HGNC Symbol;Acc:HGNC:18445]	HGLibA_07034;HGLibA_07033;HGLibA_07035
ENSG00000101882	NKAP	1	1.3076	0.34635	0.037406	0.83236	0.72908	0.95622	NFKB activating protein [Source:HGNC Symbol;Acc:HGNC:29873]	HGLibA_32000
FAM122C	FAM122C	2	1.6544	0.47939	0.015257	0.82103	0.63166	0.95326	-	HGLibA_16073;HGLibA_16072
ENSG00000116983	HPCAL4	1	1.3738	0.33297	0.031432	0.83236	0.73915	0.95685	hippocalcin like 4 [Source:HGNC Symbol;Acc:HGNC:18212]	HGLibA_22021
ENSG00000184697	CLDN6	3	1.3374	0.7202	0.034833	0.83236	0.4714	0.95326	claudin 6 [Source:HGNC Symbol;Acc:HGNC:2048]	HGLibA_09903;HGLibA_09904;HGLibA_09905
ENSG00000137821	LRRC49	1	1.219	0.36443	0.048527	0.86204	0.71554	0.95575	leucine rich repeat containing 49 [Source:HGNC Symbol;Acc:HGNC:25965]	HGLibA_27378
ENSG00000214160	ALG3	3	1.3009	0.74569	0.037866	0.83236	0.45585	0.95326	3- mannosyltransferase [Source:HGNC Symbol;Acc:HGNC:23056]"	HGLibA_01629;HGLibA_01628;HGLibA_01627
ENSG00000163029	SMC6	1	1.6239	0.28697	0.016268	0.82103	0.77413	0.95837	structural maintenance of chromosomes 6 [Source:HGNC Symbol;Acc:HGNC:20466]	HGLibA_45660
hsa-mir-1268a	hsa-mir-1268a	1	1.8362	0.25052	0.010661	0.82103	0.80218	0.96142	-	HGLibA_57497
ENSG00000179044	EXOC3L1	2	1.6021	0.40601	0.017325	0.82609	0.68474	0.95405	exocyst complex component 3 like 1 [Source:HGNC Symbol;Acc:HGNC:27540]	HGLibA_15739;HGLibA_15740
KIAA0226	KIAA0226	2	1.538	0.48419	0.020036	0.83016	0.62825	0.95326	-	HGLibA_24605;HGLibA_24604
ENSG00000164007	CLDN19	2	1.4878	0.45244	0.022793	0.83016	0.65095	0.95326	claudin 19 [Source:HGNC Symbol;Acc:HGNC:2040]	HGLibA_09875;HGLibA_09874
ENSG00000103342	GSPT1	1	4.3028	0.047174	0.00036763	0.66667	0.96237	0.99375	G1 to S phase transition 1 [Source:HGNC Symbol;Acc:HGNC:4621]	HGLibA_20440
ENSG00000152332	UHMK1	2	1.263	0.40855	0.042737	0.84121	0.68287	0.95337	U2AF homology motif kinase 1 [Source:HGNC Symbol;Acc:HGNC:19683]	HGLibA_52943;HGLibA_52941
ENSG00000060749	QSER1	3	1.243	0.76856	0.045173	0.85627	0.44215	0.95326	glutamine and serine rich 1 [Source:HGNC Symbol;Acc:HGNC:26154]	HGLibA_39752;HGLibA_39751;HGLibA_39750
ENSG00000177302	TOP3A	3	1.3117	0.47051	0.037131	0.83236	0.63799	0.95326	DNA topoisomerase III alpha [Source:HGNC Symbol;Acc:HGNC:11992]	HGLibA_51001;HGLibA_50999;HGLibA_51000
ENSG00000174891	RSRC1	1	2.0812	0.2129	0.0077662	0.81716	0.8314	0.96676	arginine and serine rich coiled-coil 1 [Source:HGNC Symbol;Acc:HGNC:24152]	HGLibA_42359
ENSG00000157036	EXOG	3	1.2313	0.70705	0.047011	0.85738	0.47953	0.95326	exo/endonuclease G [Source:HGNC Symbol;Acc:HGNC:3347]	HGLibA_15766;HGLibA_15765;HGLibA_15767
ENSG00000172795	DCP2	2	1.4302	0.40885	0.027021	0.83016	0.68265	0.95335	decapping mRNA 2 [Source:HGNC Symbol;Acc:HGNC:24452]	HGLibA_12524;HGLibA_12523
ENSG00000144713	RPL32	1	1.2334	0.36286	0.046643	0.85738	0.71671	0.95575	ribosomal protein L32 [Source:HGNC Symbol;Acc:HGNC:10336]	HGLibA_41940
ENSG00000140525	FANCI	3	1.5512	0.46489	0.019209	0.83016	0.64201	0.95326	FA complementation group I [Source:HGNC Symbol;Acc:HGNC:25568]	HGLibA_16853;HGLibA_16855;HGLibA_16854
ENSG00000158458	NRG2	2	1.3338	0.58844	0.035155	0.83236	0.55624	0.95326	neuregulin 2 [Source:HGNC Symbol;Acc:HGNC:7998]	HGLibA_32679;HGLibA_32680
ENSG00000117593	DARS2	3	1.378	0.56727	0.031065	0.83236	0.57053	0.95326	mitochondrial [Source:HGNC Symbol;Acc:HGNC:25538]"	HGLibA_12342;HGLibA_12344;HGLibA_12343
ENSG00000175267	VWA3A	3	1.3754	0.73317	0.031295	0.83236	0.46345	0.95326	von Willebrand factor A domain containing 3A [Source:HGNC Symbol;Acc:HGNC:27088]	HGLibA_53901;HGLibA_53902;HGLibA_53903
ENSG00000185024	BRF1	1	2.1728	0.20049	0.0068471	0.81716	0.8411	0.96927	RNA polymerase III transcription initiation factor subunit [Source:HGNC Symbol;Acc:HGNC:11551]"	HGLibA_04797
ENSG00000089220	PEBP1	2	1.2843	0.49092	0.039658	0.83524	0.62348	0.95326	phosphatidylethanolamine binding protein 1 [Source:HGNC Symbol;Acc:HGNC:8630]	HGLibA_36012;HGLibA_36013
hsa-mir-1296	hsa-mir-1296	3	1.2625	0.71357	0.042737	0.84121	0.47549	0.95326	-	HGLibA_57663;HGLibA_57665;HGLibA_57664
ENSG00000167139	TBC1D21	2	1.3185	0.48313	0.036625	0.83236	0.629	0.95326	TBC1 domain family member 21 [Source:HGNC Symbol;Acc:HGNC:28536]	HGLibA_48499;HGLibA_48501
ENSG00000167130	DOLPP1	2	1.3781	0.38404	0.031065	0.83236	0.70095	0.95516	dolichyldiphosphatase 1 [Source:HGNC Symbol;Acc:HGNC:29565]	HGLibA_13791;HGLibA_13790
ENSG00000161217	PCYT1A	3	1.6125	0.52731	0.016773	0.82103	0.59798	0.95326	alpha [Source:HGNC Symbol;Acc:HGNC:8754]"	HGLibA_35700;HGLibA_35701;HGLibA_35699
ENSG00000162851	TFB2M	2	1.287	0.56172	0.039245	0.83236	0.57431	0.95326	mitochondrial [Source:HGNC Symbol;Acc:HGNC:18559]"	HGLibA_49141;HGLibA_49143
ENSG00000135870	RC3H1	1	2.3489	0.17888	0.0051009	0.81716	0.85803	0.97247	ring finger and CCCH-type domains 1 [Source:HGNC Symbol;Acc:HGNC:29434]	HGLibA_40663
ENSG00000137807	KIF23	2	1.2711	0.45607	0.041772	0.84121	0.64834	0.95326	kinesin family member 23 [Source:HGNC Symbol;Acc:HGNC:6392]	HGLibA_24906;HGLibA_24908
ENSG00000196092	PAX5	3	1.419	0.5601	0.028032	0.83016	0.57541	0.95326	paired box 5 [Source:HGNC Symbol;Acc:HGNC:8619]	HGLibA_35308;HGLibA_35307;HGLibA_35306
MINOS1	MINOS1	1	2.5813	0.15421	0.0038142	0.81716	0.87745	0.97683	-	HGLibA_29278
ENSG00000266338	NBPF15	1	3.6974	0.073505	0.0013327	0.78846	0.9414	0.98891	NBPF member 15 [Source:HGNC Symbol;Acc:HGNC:28791]	HGLibA_31224
hsa-mir-518d	hsa-mir-518d	4	1.2934	0.7132	0.038693	0.83236	0.47573	0.95326	-	HGLibA_61667;HGLibA_61669;HGLibA_61668;HGLibA_61666
FAM127A	FAM127A	1	2.448	0.16877	0.0044116	0.81716	0.86598	0.97464	-	HGLibA_16087
ENSG00000035681	NSMAF	3	1.2958	0.63475	0.038417	0.83236	0.52559	0.95326	neutral sphingomyelinase activation associated factor [Source:HGNC Symbol;Acc:HGNC:8017]	HGLibA_32764;HGLibA_32762;HGLibA_32763
NAT6	NAT6	2	1.2217	0.5529	0.048251	0.85995	0.58033	0.95326	-	HGLibA_31179;HGLibA_31180
FAM96B	FAM96B	3	1.458	0.52623	0.025045	0.83016	0.59873	0.95326	-	HGLibA_16805;HGLibA_16807;HGLibA_16806
ENSG00000127980	PEX1	3	1.3174	0.58809	0.036671	0.83236	0.55647	0.95326	peroxisomal biogenesis factor 1 [Source:HGNC Symbol;Acc:HGNC:8850]	HGLibA_36079;HGLibA_36077;HGLibA_36078
ENSG00000173207	CKS1B	3	1.6661	0.58437	0.014843	0.82103	0.55897	0.95326	CDC28 protein kinase regulatory subunit 1B [Source:HGNC Symbol;Acc:HGNC:19083]	HGLibA_09786;HGLibA_09787;HGLibA_09788
ENSG00000005175	RPAP3	3	1.4431	0.56585	0.026148	0.83016	0.57149	0.95326	RNA polymerase II associated protein 3 [Source:HGNC Symbol;Acc:HGNC:26151]	HGLibA_41820;HGLibA_41819;HGLibA_41821
ENSG00000257949	TEN1	2	1.3433	0.47272	0.034144	0.83236	0.63642	0.95326	CST complex subunit [Source:HGNC Symbol;Acc:HGNC:37242]"	HGLibA_48990;HGLibA_48988
ENSG00000141569	TRIM65	3	1.7892	0.54271	0.011672	0.82103	0.58733	0.95326	tripartite motif containing 65 [Source:HGNC Symbol;Acc:HGNC:27316]	HGLibA_51585;HGLibA_51586;HGLibA_51584
ENSG00000173928	SWSAP1	2	1.7419	0.45622	0.012867	0.82103	0.64823	0.95326	SWIM-type zinc finger 7 associated protein 1 [Source:HGNC Symbol;Acc:HGNC:26638]	HGLibA_47902;HGLibA_47901
ENSG00000113312	TTC1	3	1.6867	0.42667	0.014292	0.82103	0.66962	0.95326	tetratricopeptide repeat domain 1 [Source:HGNC Symbol;Acc:HGNC:12391]	HGLibA_52046;HGLibA_52044;HGLibA_52045
ENSG00000080603	SRCAP	3	1.4284	0.54742	0.027159	0.83016	0.58409	0.95326	Snf2 related CREBBP activator protein [Source:HGNC Symbol;Acc:HGNC:16974]	HGLibA_46931;HGLibA_46932;HGLibA_46930
ENSG00000158864	NDUFS2	2	3.9526	0.10052	0.00082717	0.78846	0.91993	0.98575	NADH:ubiquinone oxidoreductase core subunit S2 [Source:HGNC Symbol;Acc:HGNC:7708]	HGLibA_31548;HGLibA_31547

DDX26B	DDX26B	3	1.4034	0.63445	0.029319	0.83236	0.52579	0.95326	-	HGLibA_12684;HGLibA_12683;HGLibA_12685
C7orf43	C7orf43	3	1.7788	0.55046	0.01181	0.82103	0.582	0.95326	-	HGLibA_06546;HGLibA_06544;HGLibA_06545
ENSG00000257529	RPL36A-HNRNPH2	2	1.3213	0.4809	0.036395	0.83236	0.63059	0.95326	RPL36A-HNRNPH2 readthrough [Source:HGNC Symbol;Acc:HGNC:48349]	HGLibA_41959;HGLibA_41958
ENSG00000160193	WDR4	2	1.9286	0.35484	0.0090988	0.81716	0.72271	0.95575	WD repeat domain 4 [Source:HGNC Symbol;Acc:HGNC:12756]	HGLibA_54086;HGLibA_54085
ENSG00000037637	FBXO42	1	2.5474	0.15639	0.003998	0.81716	0.87572	0.97658	F-box protein 42 [Source:HGNC Symbol;Acc:HGNC:29249]	HGLibA_17100
ENSG00000146410	MTFR2	1	1.4563	0.31681	0.025183	0.83016	0.75139	0.957	mitochondrial fission regulator 2 [Source:HGNC Symbol;Acc:HGNC:21115]	HGLibA_30368
ENSG00000163755	HPS3	2	1.6127	0.45816	0.016773	0.82103	0.64684	0.95326	biogenesis of lysosomal organelles complex 2 subunit 1 [Source:HGNC Symbol;Acc:HGNC:15597]"	HGLibA_22048;HGLibA_22049
ENSG00000132664	POLR3F	3	1.5922	0.56994	0.017876	0.82766	0.56872	0.95326	RNA polymerase III subunit F [Source:HGNC Symbol;Acc:HGNC:15763]	HGLibA_37623;HGLibA_37624;HGLibA_37625
ENSG00000096746	HNRNPH3	2	1.3836	0.41092	0.030467	0.83236	0.68113	0.95334	heterogeneous nuclear ribonucleoprotein H3 [Source:HGNC Symbol;Acc:HGNC:5043]	HGLibA_21827;HGLibA_21829
ENSG00000153187	HNRNPU	3	1.3005	0.66739	0.037912	0.83236	0.50452	0.95326	heterogeneous nuclear ribonucleoprotein U [Source:HGNC Symbol;Acc:HGNC:5048]	HGLibA_21846;HGLibA_21845;HGLibA_21847
ENSG00000131116	ZNF428	3	1.8013	0.55782	0.011305	0.82103	0.57697	0.95326	zinc finger protein 428 [Source:HGNC Symbol;Acc:HGNC:20804]	HGLibA_55970;HGLibA_55968;HGLibA_55969
ENSG00000196427	NBPF4	2	1.4831	0.45506	0.023161	0.83016	0.64907	0.95326	NBPF member 4 [Source:HGNC Symbol;Acc:HGNC:26550]	HGLibA_31237;HGLibA_31236
ENSG00000196459	TRAPPC2	1	2.9568	0.12307	0.0024355	0.81716	0.90206	0.98115	trafficking protein particle complex 2 [Source:HGNC Symbol;Acc:HGNC:23068]	HGLibA_51311
ENSG00000134001	EIF2S1	1	3.242	0.10123	0.0018841	0.78846	0.91936	0.98575	eukaryotic translation initiation factor 2 subunit alpha [Source:HGNC Symbol;Acc:HGNC:3265]	HGLibA_14753
ENSG00000114854	TNNC1	3	1.681	0.54527	0.014521	0.82103	0.58557	0.95326	slow skeletal and cardiac type [Source:HGNC Symbol;Acc:HGNC:11943]"	HGLibA_50866;HGLibA_50864;HGLibA_50865
ENSG00000149806	FAU	3	2.1119	0.43135	0.0075364	0.81716	0.66621	0.95326	ubiquitin like and ribosomal protein S30 fusion [Source:HGNC Symbol;Acc:HGNC:3597]"	HGLibA_16925;HGLibA_16924;HGLibA_16926
ENSG00000125637	PSD4	2	1.6108	0.41643	0.016865	0.82103	0.6771	0.95326	pleckstrin and Sec7 domain containing 4 [Source:HGNC Symbol;Acc:HGNC:19096]	HGLibA_39023;HGLibA_39021
ENSG00000197170	PSMD12	2	1.6203	0.42584	0.016451	0.82103	0.67022	0.95326	non-ATPase 12 [Source:HGNC Symbol;Acc:HGNC:9557]"	HGLibA_39156;HGLibA_39157
ENSG00000197172	MAGEA6	2	1.504	0.41929	0.021966	0.83016	0.67501	0.95326	MAGE family member A6 [Source:HGNC Symbol;Acc:HGNC:6804]	HGLibA_27918;HGLibA_27917
ENSG00000204572	KRTAP5-10	2	1.3944	0.55642	0.029824	0.83236	0.57792	0.95326	keratin associated protein 5-10 [Source:HGNC Symbol;Acc:HGNC:23605]	HGLibA_25785;HGLibA_25784
ENSG00000166710	B2M	3	2.4033	0.30236	0.0048251	0.81716	0.76238	0.95768	beta-2-microglobulin [Source:HGNC Symbol;Acc:HGNC:914]	HGLibA_03944;HGLibA_03946;HGLibA_03945
ENSG00000131747	TOP2A	2	1.7381	0.44134	0.012959	0.82103	0.65897	0.95326	DNA topoisomerase II alpha [Source:HGNC Symbol;Acc:HGNC:11989]	HGLibA_50993;HGLibA_50994
ENSG00000182903	ZNF721	1	2.485	0.16716	0.0041358	0.81716	0.86724	0.97464	zinc finger protein 721 [Source:HGNC Symbol;Acc:HGNC:29425]	HGLibA_56629
ENSG00000100344	PNPLA3	3	1.5199	0.55177	0.020679	0.83016	0.58111	0.95326	patatin like phospholipase domain containing 3 [Source:HGNC Symbol;Acc:HGNC:18590]	HGLibA_37411;HGLibA_37410;HGLibA_37412
ENSG00000170606	HSPA4	2	1.701	0.40469	0.013924	0.82103	0.6857	0.95405	heat shock protein family A (Hsp70) member 4 [Source:HGNC Symbol;Acc:HGNC:5237]	HGLibA_22284;HGLibA_22282
ENSG00000203730	TEDDM1	3	2.4681	0.29981	0.0042277	0.81716	0.76432	0.95774	transmembrane epididymal protein 1 [Source:HGNC Symbol;Acc:HGNC:30233]	HGLibA_48958;HGLibA_48959;HGLibA_48960
ENSG00000116748	AMPD1	2	1.2877	0.59441	0.039199	0.83236	0.55224	0.95326	adenosine monophosphate deaminase 1 [Source:HGNC Symbol;Acc:HGNC:468]	HGLibA_01822;HGLibA_01824
ENSG00000156261	CCT8	2	2.2554	0.26465	0.0060199	0.81716	0.79128	0.96013	chaperonin containing TCP1 subunit 8 [Source:HGNC Symbol;Acc:HGNC:1623]	HGLibA_08217;HGLibA_08216
ENSG00000068354	TBC1D25	2	1.2301	0.49827	0.047149	0.85738	0.61829	0.95326	TBC1 domain family member 25 [Source:HGNC Symbol;Acc:HGNC:8092]	HGLibA_48515;HGLibA_48516
ENSG00000175164	ABO	2	1.5879	0.4948	0.01806	0.82766	0.62074	0.95326	alpha 1-3-N-acetylgalactosaminyltransferase and alpha 1-3-galactosyltransferase [Source:HGNC Symbol;Acc:HGNC:79]"	HGLibA_00316;HGLibA_00317
ENSG00000065526	SPEN	2	1.8855	0.32064	0.0098801	0.81716	0.74849	0.95697	spen family transcriptional repressor [Source:HGNC Symbol;Acc:HGNC:17575]	HGLibA_46622;HGLibA_46620
ENSG00000016490	CLCA1	3	1.3015	0.71184	0.037774	0.83236	0.47656	0.95326	chloride channel accessory 1 [Source:HGNC Symbol;Acc:HGNC:2015]	HGLibA_09805;HGLibA_09804;HGLibA_09806
ENSG00000175110	MRPS22	1	3.3587	0.093361	0.0017462	0.78846	0.92562	0.98586	mitochondrial ribosomal protein S22 [Source:HGNC Symbol;Acc:HGNC:14508]	HGLibA_30064
ENSG00000186832	KRT16	3	1.2788	0.78334	0.040531	0.83741	0.43343	0.95326	keratin 16 [Source:HGNC Symbol;Acc:HGNC:6423]	HGLibA_25433;HGLibA_25431;HGLibA_25432
ENSG00000165527	ARF6	2	1.349	0.53883	0.0335	0.83236	0.59001	0.95326	ADP ribosylation factor 6 [Source:HGNC Symbol;Acc:HGNC:659]	HGLibA_02658;HGLibA_02657
C6orf10	C6orf10	3	1.2573	0.64559	0.043334	0.84121	0.51855	0.95326	-	HGLibA_06430;HGLibA_06432;HGLibA_06431
ENSG00000100883	SRP54	2	2.2593	0.32414	0.005928	0.81716	0.74583	0.95685	signal recognition particle 54 [Source:HGNC Symbol;Acc:HGNC:11301]	HGLibA_47006;HGLibA_47007
hsa-mir-708	hsa-mir-708	3	1.3176	0.57024	0.036671	0.83236	0.56852	0.95326	-	HGLibA_63779;HGLibA_63780;HGLibA_63781
ENSG00000015676	NUDCD3	2	2.4265	0.29276	0.0046873	0.81716	0.76971	0.9579	NudC domain containing 3 [Source:HGNC Symbol;Acc:HGNC:22208]	HGLibA_32929;HGLibA_32927
ENSG00000092010	PSME1	3	1.325	0.55201	0.035982	0.83236	0.58094	0.95326	proteasome activator subunit 1 [Source:HGNC Symbol;Acc:HGNC:9568]	HGLibA_39191;HGLibA_39190;HGLibA_39189
ENSG00000143882	ATP6V1C2	3	2.4124	0.33382	0.0047792	0.81716	0.73852	0.95685	ATPase H+ transporting V1 subunit C2 [Source:HGNC Symbol;Acc:HGNC:18264]	HGLibA_03741;HGLibA_03743;HGLibA_03742
PTPLB	PTPLB	3	1.2653	0.58324	0.042369	0.84121	0.55973	0.95326	-	HGLibA_39423;HGLibA_39424;HGLibA_39425
HIST1H2AL	HIST1H2AL	3	1.6106	0.62625	0.016865	0.82103	0.53115	0.95326	-	HGLibA_21406;HGLibA_21404;HGLibA_21405
HIST1H2AI	HIST1H2AI	2	1.506	0.44989	0.021874	0.83016	0.65279	0.95326	-	HGLibA_21396;HGLibA_21395
ENSG00000008018	PSMB1	2	1.7301	0.34807	0.013189	0.82103	0.72779	0.95601	proteasome subunit beta 1 [Source:HGNC Symbol;Acc:HGNC:9537]	HGLibA_39093;HGLibA_39094
ENSG00000144161	ZC3H8	2	2.147	0.32314	0.0071688	0.81716	0.74659	0.95692	zinc finger CCCH-type containing 8 [Source:HGNC Symbol;Acc:HGNC:30941]	HGLibA_54988;HGLibA_54987
ENSG00000151503	NCAPD3	1	4.3973	0.043932	0.00036763	0.66667	0.96496	0.99417	non-SMC condensin II complex subunit D3 [Source:HGNC Symbol;Acc:HGNC:28952]	HGLibA_31267
ENSG00000151092	NGLY1	3	1.6241	0.47701	0.016268	0.82103	0.63335	0.95326	N-glycanase 1 [Source:HGNC Symbol;Acc:HGNC:17646]	HGLibA_31882;HGLibA_31881;HGLibA_31880
ENSG00000093009	CDC45	2	1.3075	0.43359	0.037406	0.83236	0.66458	0.95326	cell division cycle 45 [Source:HGNC Symbol;Acc:HGNC:1739]	HGLibA_08587;HGLibA_08586
ENSG00000176108	CHMP6	1	1.4953	0.30952	0.022288	0.83016	0.75692	0.95728	charged multivesicular body protein 6 [Source:HGNC Symbol;Acc:HGNC:25675]	HGLibA_09498
ENSG00000110906	KCTD10	2	1.9333	0.33505	0.0090988	0.81716	0.73759	0.95685	potassium channel tetramerization domain containing 10 [Source:HGNC Symbol;Acc:HGNC:23236]	HGLibA_24414;HGLibA_24415
ENSG00000100258	LMF2	2	1.6193	0.47098	0.016451	0.82103	0.63766	0.95326	lipase maturation factor 2 [Source:HGNC Symbol;Acc:HGNC:25096]	HGLibA_26687;HGLibA_26686
ENSG00000160783	PMF1	2	1.5629	0.50822	0.018887	0.82766	0.6113	0.95326	polyamine modulated factor 1 [Source:HGNC Symbol;Acc:HGNC:9112]	HGLibA_37303;HGLibA_37302
ENSG00000232838	PET117	2	1.6817	0.44076	0.014475	0.82103	0.65939	0.95326	PET117 homolog [Source:HGNC Symbol;Acc:HGNC:40045]	HGLibA_36076;HGLibA_36075
ENSG00000140521	POLG	2	1.5044	0.43493	0.02192	0.83016	0.66362	0.95326	catalytic subunit [Source:HGNC Symbol;Acc:HGNC:9179]"	HGLibA_37523;HGLibA_37522
ENSG00000151151	IPMK	3	1.4527	0.57316	0.025321	0.83016	0.56654	0.95326	inositol polyphosphate multikinase [Source:HGNC Symbol;Acc:HGNC:20739]	HGLibA_23458;HGLibA_23456;HGLibA_23457
hsa-mir-1277	hsa-mir-1277	2	1.7231	0.3876	0.013235	0.82103	0.69831	0.95516	-	HGLibA_57565;HGLibA_57563
ENSG00000087088	BAX	3	1.2885	0.6186	0.039153	0.83236	0.53618	0.95326	apoptosis regulator [Source:HGNC Symbol;Acc:HGNC:959]"	HGLibA_04183;HGLibA_04181;HGLibA_04182
hsa-mir-5591	hsa-mir-5591	2	1.3413	0.46177	0.034419	0.83236	0.64425	0.95326	-	HGLibA_62204;HGLibA_62205
ENSG00000187733	AMY1C	3	1.2871	0.52456	0.039245	0.83236	0.59989	0.95326	alpha 1C (salivary) [Source:HGNC Symbol;Acc:HGNC:476]"	HGLibA_01848;HGLibA_01847;HGLibA_01846
ENSG00000115539	PDCL3	2	1.2929	0.47875	0.038739	0.83236	0.63212	0.95326	phosducin like 3 [Source:HGNC Symbol;Acc:HGNC:28860]	HGLibA_35754;HGLibA_35753
ENSG00000146143	PRIM2	2	1.4554	0.54273	0.025229	0.83016	0.58732	0.95326	DNA primase subunit 2 [Source:HGNC Symbol;Acc:HGNC:9370]	HGLibA_38486;HGLibA_38485
ENSG00000178096	BOLA1	3	2.0701	0.38389	0.007904	0.81716	0.70106	0.95516	bolA family member 1 [Source:HGNC Symbol;Acc:HGNC:24263]	HGLibA_04679;HGLibA_04681;HGLibA_04680
ENSG00000125449	ARMC7	2	1.9855	0.37891	0.0085474	0.81716	0.70475	0.95516	armadillo repeat containing 7 [Source:HGNC Symbol;Acc:HGNC:26168]	HGLibA_03030;HGLibA_03029
ENSG00000174527	MYO1H	3	1.4741	0.52835	0.023528	0.83016	0.59726	0.95326	myosin IH [Source:HGNC Symbol;Acc:HGNC:13879]	HGLibA_30841;HGLibA_30842;HGLibA_30840
ENSG00000170860	LSM3	2	3.9274	0.10763	0.00091908	0.78846	0.91429	0.98406	U6 small nuclear RNA and mRNA degradation associated [Source:HGNC Symbol;Acc:HGNC:17874]"	HGLibA_27540;HGLibA_27539
ENSG00000113758	DBN1	3	1.5458	0.53775	0.019484	0.83016	0.59075	0.95326	drebrin 1 [Source:HGNC Symbol;Acc:HGNC:2695]	HGLibA_12389;HGLibA_12388;HGLibA_12387
ENSG00000177669	MBOAT4	3	1.5861	0.61587	0.018152	0.82766	0.53798	0.95326	membrane bound O-acyltransferase domain containing 4 [Source:HGNC Symbol;Acc:HGNC:32311]	HGLibA_28522;HGLibA_28524;HGLibA_28523
ENSG00000169306	IL1RAPL1	3	1.2349	0.68972	0.046505	0.85738	0.49037	0.95326	interleukin 1 receptor accessory protein like 1 [Source:HGNC Symbol;Acc:HGNC:5996]	HGLibA_23054;HGLibA_23055;HGLibA_23056
ENSG00000174839	DENND6A	3	1.3157	0.66341	0.036671	0.83236	0.50707	0.95326	DENN domain containing 6A [Source:HGNC Symbol;Acc:HGNC:26635]	HGLibA_12965;HGLibA_12964;HGLibA_12966
ENSG00000109846	CRYAB	2	1.5155	0.50062	0.021414	0.83016	0.61664	0.95326	crystallin alpha B [Source:HGNC Symbol;Acc:HGNC:2389]	HGLibA_11250;HGLibA_11248
ENSG00000181192	DHTKD1	3	1.5367	0.62005	0.020128	0.83016	0.53523	0.95326	dehydrogenase E1 and transketolase domain containing 1 [Source:HGNC Symbol;Acc:HGNC:23537]	HGLibA_13162;HGLibA_13163;HGLibA_13164
ENSG00000177879	AP3S1	3	1.3265	0.60145	0.03589	0.83236	0.54754	0.95326	adaptor related protein complex 3 subunit sigma 1 [Source:HGNC Symbol;Acc:HGNC:2013]	HGLibA_02336;HGLibA_02334;HGLibA_02335
ENSG00000181449	SOX2	2	1.4592	0.54629	0.024999	0.83016	0.58487	0.95326	SRY-box 2 [Source:HGNC Symbol;Acc:HGNC:11195]	HGLibA_46252;HGLibA_46253
ENSG00000223611	SUPT20HL2	2	1.4448	0.40307	0.025918	0.83016	0.6869	0.95405	SUPT20H like 2 [Source:HGNC Symbol;Acc:HGNC:31797]	HGLibA_47810;HGLibA_47812
ENSG00000165060	FXN	3	1.3723	0.57272	0.03157	0.83236	0.56683	0.95326	frataxin [Source:HGNC Symbol;Acc:HGNC:3951]	HGLibA_18214;HGLibA_18215;HGLibA_18216
ENSG00000196497	IPO4	3	1.2208	0.62305	0.048251	0.85995	0.53325	0.95326	importin 4 [Source:HGNC Symbol;Acc:HGNC:19426]	HGLibA_23465;HGLibA_23467;HGLibA_23466
hsa-mir-4784	hsa-mir-4784	2	1.2801	0.60317	0.040256	0.83524	0.5464	0.95326	-	HGLibA_61243;HGLibA_61245
ENSG00000179915	NRXN1	3	1.3582	0.65512	0.032765	0.83236	0.51239	0.95326	neurexin 1 [Source:HGNC Symbol;Acc:HGNC:8008]	HGLibA_32734;HGLibA_32733;HGLibA_32732
ENSG00000141314	RHBDL3	1	4.4716	0.041521	0.00027572	0.66667	0.96688	0.99417	rhomboid like 3 [Source:HGNC Symbol;Acc:HGNC:16502]	HGLibA_41143
hsa-mir-1184-3	hsa-mir-1184-3	2	1.4232	0.45714	0.027618	0.83016	0.64757	0.95326	-	HGLibA_57236;HGLibA_57238
ENSG00000004866	ST7	3	1.2685	0.72823	0.042048	0.84121	0.46647	0.95326	suppression of tumorigenicity 7 [Source:HGNC Symbol;Acc:HGNC:11351]	HGLibA_47283;HGLibA_47282;HGLibA_47281
ENSG00000103091	WDR59	2	1.6343	0.47421	0.015992	0.82103	0.63535	0.95326	WD repeat domain 59 [Source:HGNC Symbol;Acc:HGNC:25706]	HGLibA_54129;HGLibA_54130
ENSG00000179059	ZFP42	3	1.2584	0.58537	0.043242	0.84121	0.5583	0.95326	ZFP42 zinc finger protein [Source:HGNC Symbol;Acc:HGNC:30949]	HGLibA_55220;HGLibA_55218;HGLibA_55219
ENSG00000132612	VPS4A	3	1.2874	0.77009	0.039245	0.83236	0.44125	0.95326	vacuolar protein sorting 4 homolog A [Source:HGNC Symbol;Acc:HGNC:13488]	HGLibA_53797;HGLibA_53796;HGLibA_53798
ENSG00000182795	C1orf116	2	1.2808	0.46404	0.040164	0.83524	0.64262	0.95326	chromosome 1 open reading frame 116 [Source:HGNC Symbol;Acc:HGNC:28667]	HGLibA_05757;HGLibA_05756
ENSG00000126261	UBA2	1	2.4685	0.17043	0.0042277	0.81716	0.86467	0.97452	ubiquitin like modifier activating enzyme 2 [Source:HGNC Symbol;Acc:HGNC:30661]	HGLibA_52517
ENSG00000153822	KCNJ16	3	1.3769	0.64216	0.031111	0.83236	0.52077	0.95326	potassium voltage-gated channel subfamily J member 16 [Source:HGNC Symbol;Acc:HGNC:6262]	HGLibA_24266;HGLibA_24267;HGLibA_24268
ENSG00000167178	ISLR2	2	1.4932	0.5285	0.022471	0.83016	0.59715	0.95326	immunoglobulin superfamily containing leucine rich repeat 2 [Source:HGNC Symbol;Acc:HGNC:29286]	HGLibA_23672;HGLibA_23673
ENSG00000156925	ZIC3	3	1.5026	0.56269	0.022058	0.83016	0.57365	0.95326	Zic family member 3 [Source:HGNC Symbol;Acc:HGNC:12874]	HGLibA_55327;HGLibA_55326;HGLibA_55328
ENSG00000069248	NUP133	3	1.4365	0.44313	0.026515	0.83016	0.65767	0.95326	nucleoporin 133 [Source:HGNC Symbol;Acc:HGNC:18016]	HGLibA_33021;HGLibA_33022;HGLibA_33020
ENSG00000188493	C19orf54	3	1.4886	0.55293	0.022747	0.83016	0.58031	0.95326	chromosome 19 open reading frame 54 [Source:HGNC Symbol;Acc:HGNC:24758]	HGLibA_05680;HGLibA_05681;HGLibA_05682
ENSG00000163918	RFC4	2	1.2876	0.59221	0.039199	0.83236	0.55371	0.95326	replication factor C subunit 4 [Source:HGNC Symbol;Acc:HGNC:9972]	HGLibA_40912;HGLibA_40911
ENSG00000249581	CLRN2	3	1.2397	0.7078	0.045908	0.85666	0.47907	0.95326	clarin 2 [Source:HGNC Symbol;Acc:HGNC:33939]	HGLibA_10126;HGLibA_10125;HGLibA_10124
ENSG00000101199	ARFGAP1	3	1.41	0.52932	0.028675	0.83201	0.59658	0.95326	ADP ribosylation factor GTPase activating protein 1 [Source:HGNC Symbol;Acc:HGNC:15852]	HGLibA_02661;HGLibA_02662;HGLibA_02660
ENSG00000100029	PES1	2	1.252	0.42016	0.044162	0.85044	0.67437	0.95326	pescadillo ribosomal biogenesis factor 1 [Source:HGNC Symbol;Acc:HGNC:8848]	HGLibA_36067;HGLibA_36065
ENSG00000100028	SNRPD3	2	1.7826	0.339	0.011764	0.82103	0.73461	0.95685	small nuclear ribonucleoprotein D3 polypeptide [Source:HGNC Symbol;Acc:HGNC:11160]	HGLibA_45982;HGLibA_45983
ENSG00000001167	NFYA	1	2.1926	0.19876	0.0066633	0.81716	0.84245	0.96927	nuclear transcription factor Y subunit alpha [Source:HGNC Symbol;Acc:HGNC:7804]	HGLibA_31853
ENSG00000105486	LIG1	2	1.2684	0.41058	0.04214	0.84121	0.68138	0.95334	DNA ligase 1 [Source:HGNC Symbol;Acc:HGNC:6598]	HGLibA_26480;HGLibA_26479
ENSG00000125107	CNOT1	2	1.288	0.50321	0.039153	0.83236	0.61482	0.95326	CCR4-NOT transcription complex subunit 1 [Source:HGNC Symbol;Acc:HGNC:7877]	HGLibA_10325;HGLibA_10327
ENSG00000134371	CDC73	3	1.6652	0.48676	0.014889	0.82103	0.62643	0.95326	cell division cycle 73 [Source:HGNC Symbol;Acc:HGNC:16783]	HGLibA_08598;HGLibA_08599;HGLibA_08600
ENSG00000134597	RBMX2	1	1.351	0.33743	0.033316	0.83236	0.73579	0.95685	RNA binding motif protein X-linked 2 [Source:HGNC Symbol;Acc:HGNC:24282]	HGLibA_40600
hsa-mir-5087	hsa-mir-5087	2	3.3645	0.15587	0.0017462	0.78846	0.87614	0.97658	-	HGLibA_61496;HGLibA_61495
hsa-mir-548f-3	hsa-mir-548f-3	3	1.6466	0.53529	0.015578	0.82103	0.59245	0.95326	-	HGLibA_61981;HGLibA_61980;HGLibA_61979
hsa-mir-324	hsa-mir-324	4	1.2895	0.63651	0.039061	0.83236	0.52444	0.95326	-	HGLibA_58963;HGLibA_58965;HGLibA_58964;HGLibA_58962
ENSG00000142937	RPS8	2	1.2265	0.42649	0.047654	0.85844	0.66975	0.95326	ribosomal protein S8 [Source:HGNC Symbol;Acc:HGNC:10441]	HGLibA_42191;HGLibA_42190
ENSG00000124486	USP9X	3	1.3386	0.70236	0.034695	0.83236	0.48246	0.95326	ubiquitin specific peptidase 9 X-linked [Source:HGNC Symbol;Acc:HGNC:12632]	HGLibA_53416;HGLibA_53415;HGLibA_53417
ENSG00000165702	GFI1B	3	1.2866	0.72397	0.039245	0.83236	0.46908	0.95326	growth factor independent 1B transcriptional repressor [Source:HGNC Symbol;Acc:HGNC:4238]	HGLibA_18944;HGLibA_18945;HGLibA_18943
ENSG00000131791	PRKAB2	3	1.2188	0.71409	0.048573	0.86215	0.47517	0.95326	protein kinase AMP-activated non-catalytic subunit beta 2 [Source:HGNC Symbol;Acc:HGNC:9379]	HGLibA_38501;HGLibA_38500;HGLibA_38499
ENSG00000121579	NAA50	2	3.3178	0.16483	0.0017922	0.78846	0.86908	0.97535	NatE catalytic subunit [Source:HGNC Symbol;Acc:HGNC:29533]"	HGLibA_31000;HGLibA_30999
ENSG00000186212	SOWAHB	3	1.6226	0.55306	0.016314	0.82103	0.58022	0.95326	sosondowah ankyrin repeat domain family member B [Source:HGNC Symbol;Acc:HGNC:32958]	HGLibA_46215;HGLibA_46217;HGLibA_46216
ENSG00000110075	PPP6R3	2	1.8074	0.42336	0.011213	0.82103	0.67203	0.95326	protein phosphatase 6 regulatory subunit 3 [Source:HGNC Symbol;Acc:HGNC:1173]	HGLibA_38226;HGLibA_38228
ENSG00000053524	MCF2L2	2	2.0937	0.3305	0.0076743	0.81716	0.74103	0.95685	MCF.2 cell line derived transforming sequence-like 2 [Source:HGNC Symbol;Acc:HGNC:30319]	HGLibA_28584;HGLibA_28582
ENSG00000167394	ZNF668	3	1.3508	0.68121	0.033316	0.83236	0.49574	0.95326	zinc finger protein 668 [Source:HGNC Symbol;Acc:HGNC:25821]	HGLibA_56495;HGLibA_56493;HGLibA_56494
ENSG00000172638	EFEMP2	2	1.5415	0.40357	0.019668	0.83016	0.68653	0.95405	EGF containing fibulin extracellular matrix protein 2 [Source:HGNC Symbol;Acc:HGNC:3219]	HGLibA_14565;HGLibA_14563
ENSG00000112282	MED23	2	3.1589	0.18422	0.002022	0.79661	0.85384	0.97162	mediator complex subunit 23 [Source:HGNC Symbol;Acc:HGNC:2372]	HGLibA_28782;HGLibA_28781
ENSG00000163739	CXCL1	3	1.3174	0.62023	0.036671	0.83236	0.53511	0.95326	C-X-C motif chemokine ligand 1 [Source:HGNC Symbol;Acc:HGNC:4602]	HGLibA_11842;HGLibA_11843;HGLibA_11841
ENSG00000112796	ENPP5	2	1.2689	0.53222	0.042048	0.84121	0.59457	0.95326	ectonucleotide pyrophosphatase/phosphodiesterase 5 (putative) [Source:HGNC Symbol;Acc:HGNC:13717]	HGLibA_15179;HGLibA_15180
ENSG00000108344	PSMD3	2	1.5661	0.43261	0.018841	0.82766	0.6653	0.95326	non-ATPase 3 [Source:HGNC Symbol;Acc:HGNC:9560]"	HGLibA_39168;HGLibA_39169
hsa-mir-3152	hsa-mir-3152	4	1.3724	0.58969	0.031524	0.83236	0.5554	0.95326	-	HGLibA_58668;HGLibA_58666;HGLibA_58669;HGLibA_58667
ENSG00000196277	GRM7	2	2.0791	0.33445	0.0077662	0.81716	0.73804	0.95685	glutamate metabotropic receptor 7 [Source:HGNC Symbol;Acc:HGNC:4599]	HGLibA_20358;HGLibA_20359
ENSG00000185264	TEX33	1	1.8026	0.25637	0.011259	0.82103	0.79767	0.96142	testis expressed 33 [Source:HGNC Symbol;Acc:HGNC:28568]	HGLibA_49096
ENSG00000160654	CD3G	3	1.4352	0.64315	0.026653	0.83016	0.52012	0.95326	CD3g molecule [Source:HGNC Symbol;Acc:HGNC:1675]	HGLibA_08377;HGLibA_08378;HGLibA_08379
ENSG00000150048	CLEC1A	3	1.5345	0.53187	0.020128	0.83016	0.59481	0.95326	C-type lectin domain family 1 member A [Source:HGNC Symbol;Acc:HGNC:24355]	HGLibA_09956;HGLibA_09954;HGLibA_09955
ENSG00000100767	PAPLN	1	1.9901	0.226	0.0084095	0.81716	0.8212	0.96439	proteoglycan like sulfated glycoprotein [Source:HGNC Symbol;Acc:HGNC:19262]"	HGLibA_35132
ENSG00000120314	WDR55	2	2.0135	0.33197	0.0083176	0.81716	0.73991	0.95685	WD repeat domain 55 [Source:HGNC Symbol;Acc:HGNC:25971]	HGLibA_54127;HGLibA_54128
ENSG00000197635	DPP4	2	1.7891	0.44367	0.011672	0.82103	0.65728	0.95326	dipeptidyl peptidase 4 [Source:HGNC Symbol;Acc:HGNC:3009]	HGLibA_13873;HGLibA_13871
ENSG00000176182	MYPOP	3	1.9143	0.50734	0.0093286	0.81716	0.61191	0.95326	partner of profilin [Source:HGNC Symbol;Acc:HGNC:20178]"	HGLibA_30912;HGLibA_30914;HGLibA_30913
ENSG00000205744	DENND1C	2	1.2708	0.59048	0.041818	0.84121	0.55487	0.95326	DENN domain containing 1C [Source:HGNC Symbol;Acc:HGNC:26225]	HGLibA_12935;HGLibA_12934
ENSG00000115942	ORC2	3	1.617	0.63225	0.016543	0.82103	0.52722	0.95326	origin recognition complex subunit 2 [Source:HGNC Symbol;Acc:HGNC:8488]	HGLibA_34603;HGLibA_34604;HGLibA_34605
ENSG00000115053	NCL	1	3.4295	0.088852	0.0016543	0.78846	0.9292	0.98661	nucleolin [Source:HGNC Symbol;Acc:HGNC:7667]	HGLibA_31326
ENSG00000133193	FAM104A	2	1.2382	0.59561	0.046092	0.85666	0.55143	0.95326	family with sequence similarity 104 member A [Source:HGNC Symbol;Acc:HGNC:25918]	HGLibA_15988;HGLibA_15987
ENSG00000244005	NFS1	1	1.8235	0.25184	0.010845	0.82103	0.80116	0.96142	cysteine desulfurase [Source:HGNC Symbol;Acc:HGNC:15910]"	HGLibA_31843
hsa-mir-433	hsa-mir-433	3	1.3741	0.59267	0.031432	0.83236	0.5534	0.95326	-	HGLibA_60042;HGLibA_60045;HGLibA_60043
hsa-mir-556	hsa-mir-556	2	1.2689	0.53202	0.042048	0.84121	0.59471	0.95326	-	HGLibA_62139;HGLibA_62140
ENSG00000153885	KCTD15	2	1.2789	0.55885	0.040531	0.83741	0.57626	0.95326	potassium channel tetramerization domain containing 15 [Source:HGNC Symbol;Acc:HGNC:23297]	HGLibA_24428;HGLibA_24429
hsa-mir-93	hsa-mir-93	1	1.4332	0.32203	0.026837	0.83016	0.74743	0.95695	-	HGLibA_64303

Table S2 Positive candidate genes in 2nd screen

Gene	Gene Name	sgRNA	group|beta	group|z	group|p-valu	group|fd	group|wald-p-valu	group|wald-fd	Description	sgRNA ID
ENSG00000111358	GTF2H3	1	1.2167	0.36654	0.040867	0.87097	0.71396	0.9521	general transcription factor IIH subunit 3 [Source:HGNC Symbol;Acc:HGNC:4657]	HGLibA_20556
ENSG00000167741	GGT6	2	2.0258	0.3356	0.0050969	0.79902	0.73717	0.95358	gamma-glutamyltransferase 6 [Source:HGNC Symbol;Acc:HGNC:26891]	HGLibA_19017;HGLibA_19015
ENSG00000188342	GTF2F2	3	1.3789	0.54334	0.024153	0.84371	0.5869	0.95153	general transcription factor IIF subunit 2 [Source:HGNC Symbol;Acc:HGNC:4653]	HGLibA_20541;HGLibA_20540;HGLibA_20542
ENSG00000162594	IL23R	2	1.3324	0.50773	0.027734	0.84371	0.61164	0.95153	interleukin 23 receptor [Source:HGNC Symbol;Acc:HGNC:19100]	HGLibA_23100;HGLibA_23099
ENSG00000179950	PUF60	2	1.4377	0.55219	0.020296	0.84371	0.58082	0.95153	poly(U) binding splicing factor 60 [Source:HGNC Symbol;Acc:HGNC:17042]	HGLibA_39578;HGLibA_39576
ENSG00000174428	GTF2IRD2B	2	1.3369	0.51998	0.027413	0.84371	0.60307	0.95153	GTF2I repeat domain containing 2B [Source:HGNC Symbol;Acc:HGNC:33125]	HGLibA_20573;HGLibA_20574
ENSG00000073464	CLCN4	3	1.2154	0.67225	0.04151	0.87558	0.50143	0.95153	chloride voltage-gated channel 4 [Source:HGNC Symbol;Acc:HGNC:2022]	HGLibA_09829;HGLibA_09830;HGLibA_09828
ENSG00000101972	STAG2	2	1.781	0.33067	0.0083571	0.83871	0.7409	0.95441	stromal antigen 2 [Source:HGNC Symbol;Acc:HGNC:11355]	HGLibA_47325;HGLibA_47323
ENSG00000174437	ATP2A2	2	1.5421	0.36988	0.014648	0.84371	0.71148	0.95153	ATPase sarcoplasmic/endoplasmic reticulum Ca2+ transporting 2 [Source:HGNC Symbol;Acc:HGNC:812]	HGLibA_03608;HGLibA_03607
ENSG00000085978	ATG16L1	3	1.1728	0.70825	0.047571	0.87787	0.47879	0.95153	autophagy related 16 like 1 [Source:HGNC Symbol;Acc:HGNC:21498]	HGLibA_03472;HGLibA_03473;HGLibA_03474
ENSG00000185133	INPP5J	2	1.8871	0.39517	0.0065203	0.79902	0.69272	0.95153	inositol polyphosphate-5-phosphatase J [Source:HGNC Symbol;Acc:HGNC:8956]	HGLibA_23358;HGLibA_23357
ENSG00000108384	RAD51C	3	1.2904	0.61619	0.031637	0.84371	0.53777	0.95153	RAD51 paralog C [Source:HGNC Symbol;Acc:HGNC:9820]	HGLibA_40089;HGLibA_40090;HGLibA_40091
ENSG00000091140	DLD	1	2.0039	0.22367	0.0053265	0.79902	0.82301	0.96422	dihydrolipoamide dehydrogenase [Source:HGNC Symbol;Acc:HGNC:2898]	HGLibA_13316
ENSG00000177045	SIX5	2	1.5773	0.50778	0.013546	0.84371	0.61161	0.95153	SIX homeobox 5 [Source:HGNC Symbol;Acc:HGNC:10891]	HGLibA_44256;HGLibA_44254
ENSG00000137804	NUSAP1	2	1.258	0.61024	0.035403	0.85525	0.5417	0.95153	nucleolar and spindle associated protein 1 [Source:HGNC Symbol;Acc:HGNC:18538]	HGLibA_33097;HGLibA_33095
ENSG00000196943	NOP9	2	1.2598	0.41007	0.034806	0.85011	0.68176	0.95153	NOP9 nucleolar protein [Source:HGNC Symbol;Acc:HGNC:19826]	HGLibA_32319;HGLibA_32320
ENSG00000164220	F2RL2	1	2.2654	0.18838	0.0032602	0.784	0.85058	0.96918	coagulation factor II thrombin receptor like 2 [Source:HGNC Symbol;Acc:HGNC:3539]	HGLibA_15869
ENSG00000114503	NCBP2	3	1.1923	0.61999	0.045137	0.87787	0.53526	0.95153	nuclear cap binding protein subunit 2 [Source:HGNC Symbol;Acc:HGNC:7659]	HGLibA_31283;HGLibA_31285;HGLibA_31284
ENSG00000169764	UGP2	2	1.3101	0.4096	0.030076	0.84371	0.6821	0.95153	UDP-glucose pyrophosphorylase 2 [Source:HGNC Symbol;Acc:HGNC:12527]	HGLibA_52874;HGLibA_52873
ENSG00000144559	TAMM41	2	1.3809	0.52812	0.023969	0.84371	0.59742	0.95153	TAM41 mitochondrial translocator assembly and maintenance homolog [Source:HGNC Symbol;Acc:HGNC:25187]	HGLibA_48288;HGLibA_48287
ENSG00000147509	RGS20	2	2.6878	0.23367	0.0014694	0.7619	0.81524	0.96285	regulator of G protein signaling 20 [Source:HGNC Symbol;Acc:HGNC:14600]	HGLibA_41081;HGLibA_41082
ENSG00000169592	INO80E	3	1.346	0.45517	0.026449	0.84371	0.64898	0.95153	INO80 complex subunit E [Source:HGNC Symbol;Acc:HGNC:26905]	HGLibA_23330;HGLibA_23331;HGLibA_23332
ENSG00000169594	BNC1	3	1.3017	0.71921	0.030811	0.84371	0.47201	0.95153	basonuclin 1 [Source:HGNC Symbol;Acc:HGNC:1081]	HGLibA_04645;HGLibA_04643;HGLibA_04644
LOC100130705	LOC100130705	1	1.555	0.29711	0.014143	0.84371	0.76639	0.95707	-	HGLibA_26795
ENSG00000106636	YKT6	3	1.4235	0.45502	0.021352	0.84371	0.6491	0.95153	YKT6 v-SNARE homolog [Source:HGNC Symbol;Acc:HGNC:16959]	HGLibA_54694;HGLibA_54693;HGLibA_54695
ENSG00000137100	DCTN3	2	1.3161	0.43961	0.029571	0.84371	0.66022	0.95153	dynactin subunit 3 [Source:HGNC Symbol;Acc:HGNC:2713]	HGLibA_12551;HGLibA_12549
ENSG00000153107	ANAPC1	2	2.4883	0.25517	0.0023418	0.76812	0.7986	0.95997	anaphase promoting complex subunit 1 [Source:HGNC Symbol;Acc:HGNC:19988]	HGLibA_01861;HGLibA_01862
ENSG00000133313	CNDP2	2	1.2058	0.49671	0.042841	0.87558	0.61939	0.95153	carnosine dipeptidase 2 [Source:HGNC Symbol;Acc:HGNC:24437]	HGLibA_10255;HGLibA_10253
ENSG00000122728	TAF1L	2	1.4029	0.54435	0.022454	0.84371	0.5862	0.95153	TATA-box binding protein associated factor 1 like [Source:HGNC Symbol;Acc:HGNC:18056]	HGLibA_48225;HGLibA_48224
hsa-mir-99a	hsa-mir-99a	1	3.515	0.083602	0.00041326	0.5625	0.93337	0.9856	-	HGLibA_64379
ENSG00000104826	LHB	3	1.1734	0.71273	0.047525	0.87787	0.47602	0.95153	luteinizing hormone beta polypeptide [Source:HGNC Symbol;Acc:HGNC:6584]	HGLibA_26420;HGLibA_26419;HGLibA_26421
ENSG00000103479	RBL2	3	1.3152	0.67723	0.029617	0.84371	0.49826	0.95153	RB transcriptional corepressor like 2 [Source:HGNC Symbol;Acc:HGNC:9894]	HGLibA_40470;HGLibA_40471;HGLibA_40469
ENSG00000204007	GLT6D1	2	1.5564	0.49323	0.014143	0.84371	0.62185	0.95153	glycosyltransferase 6 domain containing 1 [Source:HGNC Symbol;Acc:HGNC:23671]	HGLibA_19333;HGLibA_19335
hsa-mir-4419a	hsa-mir-4419a	1	1.1773	0.37401	0.046836	0.87787	0.7084	0.95153	-	HGLibA_60059
ENSG00000116161	CACYBP	1	1.8813	0.24204	0.006704	0.79902	0.80875	0.96114	calcyclin binding protein [Source:HGNC Symbol;Acc:HGNC:30423]	HGLibA_06958
ENSG00000164434	FABP7	3	1.2141	0.6878	0.041648	0.87558	0.49158	0.95153	fatty acid binding protein 7 [Source:HGNC Symbol;Acc:HGNC:3562]	HGLibA_15929;HGLibA_15927;HGLibA_15928
ENSG00000170162	VGLL2	1	1.4196	0.32294	0.021444	0.84371	0.74674	0.95475	vestigial like family member 2 [Source:HGNC Symbol;Acc:HGNC:20232]	HGLibA_53637
ENSG00000104613	INTS10	3	1.2176	0.61722	0.040683	0.87097	0.53709	0.95153	integrator complex subunit 10 [Source:HGNC Symbol;Acc:HGNC:25548]	HGLibA_23408;HGLibA_23410;HGLibA_23409
ENSG00000027644	INSRR	2	1.1662	0.58171	0.048397	0.87787	0.56076	0.95153	insulin receptor related receptor [Source:HGNC Symbol;Acc:HGNC:6093]	HGLibA_23403;HGLibA_23402
ENSG00000131400	NAPSA	2	1.5582	0.45853	0.014097	0.84371	0.64657	0.95153	napsin A aspartic peptidase [Source:HGNC Symbol;Acc:HGNC:13395]	HGLibA_31139;HGLibA_31140
ENSG00000198758	EPS8L3	2	1.836	0.35392	0.0074387	0.79902	0.7234	0.95266	EPS8 like 3 [Source:HGNC Symbol;Acc:HGNC:21297]	HGLibA_15382;HGLibA_15383
ENSG00000150760	DOCK1	3	1.2205	0.71957	0.040362	0.87097	0.47179	0.95153	dedicator of cytokinesis 1 [Source:HGNC Symbol;Acc:HGNC:2987]	HGLibA_13732;HGLibA_13730;HGLibA_13731
ENSG00000182993	C12orf60	3	1.4784	0.54641	0.017908	0.84371	0.58478	0.95153	chromosome 12 open reading frame 60 [Source:HGNC Symbol;Acc:HGNC:28726]	HGLibA_05273;HGLibA_05274;HGLibA_05272
ENSG00000197958	RPL12	1	1.3278	0.34224	0.028285	0.84371	0.73217	0.95275	ribosomal protein L12 [Source:HGNC Symbol;Acc:HGNC:10302]	HGLibA_41866
ENSG00000139428	MMAB	3	1.3119	0.73537	0.029893	0.84371	0.46212	0.95153	metabolism of cobalamin associated B [Source:HGNC Symbol;Acc:HGNC:19331]	HGLibA_29453;HGLibA_29454;HGLibA_29452
hsa-mir-548ai	hsa-mir-548ai	3	1.8914	0.4301	0.0065203	0.79902	0.66713	0.95153	-	HGLibA_61884;HGLibA_61883;HGLibA_61886

hsa-mir-548ao	hsa-mir-548ao	3	1.2786	0.72406	0.032739	0.84371	0.46903	0.95153	-	HGLibA_61907;HGLibA_61909;HGLibA_61910
ENSG00000137203	TFAP2A	2	1.245	0.61463	0.037377	0.85956	0.5388	0.95153	transcription factor AP-2 alpha [Source:HGNC Symbol;Acc:HGNC:11742]	HGLibA_49122;HGLibA_49120
ENSG00000060688	SNRNP40	2	1.3296	0.39995	0.028148	0.84371	0.68919	0.95153	small nuclear ribonucleoprotein U5 subunit 40 [Source:HGNC Symbol;Acc:HGNC:30857]	HGLibA_45952;HGLibA_45954
hsa-mir-7856	hsa-mir-7856	4	1.2093	0.73327	0.04229	0.87558	0.4634	0.95153	-	HGLibA_64012;HGLibA_64015;HGLibA_64014;HGLibA_64013
ENSG00000010256	UQCRC1	3	1.3327	0.7473	0.027734	0.84371	0.45488	0.95153	ubiquinol-cytochrome c reductase core protein 1 [Source:HGNC Symbol;Acc:HGNC:12585]	HGLibA_53113;HGLibA_53112;HGLibA_53114
ENSG00000182504	CEP97	2	1.2301	0.62498	0.038892	0.86735	0.53199	0.95153	centrosomal protein 97 [Source:HGNC Symbol;Acc:HGNC:26244]	HGLibA_09183;HGLibA_09181
ENSG00000113595	TRIM23	2	1.4973	0.51392	0.01676	0.84371	0.60731	0.95153	tripartite motif containing 23 [Source:HGNC Symbol;Acc:HGNC:660]	HGLibA_51434;HGLibA_51435
ENSG00000151917	BEND6	1	1.5994	0.28928	0.012857	0.84371	0.77237	0.95762	BEN domain containing 6 [Source:HGNC Symbol;Acc:HGNC:20871]	HGLibA_04412
ENSG00000180828	BHLHE22	3	1.3496	0.67296	0.026127	0.84371	0.50097	0.95153	basic helix-loop-helix family member e22 [Source:HGNC Symbol;Acc:HGNC:11963]	HGLibA_04476;HGLibA_04475;HGLibA_04477
ENSG00000163605	PPP4R2	2	2.293	0.30889	0.0030765	0.784	0.7541	0.95596	protein phosphatase 4 regulatory subunit 2 [Source:HGNC Symbol;Acc:HGNC:18296]	HGLibA_38207;HGLibA_38205
ENSG00000145740	SLC30A5	1	3.7426	0.071111	0.00041326	0.5625	0.94331	0.98744	solute carrier family 30 member 5 [Source:HGNC Symbol;Acc:HGNC:19089]	HGLibA_44911
hsa-mir-3908	hsa-mir-3908	1	1.4667	0.3145	0.018229	0.84371	0.75314	0.95583	-	HGLibA_59498
hsa-mir-1322	hsa-mir-1322	1	1.5771	0.2938	0.013546	0.84371	0.76891	0.95707	-	HGLibA_57762
ENSG00000160862	AZGP1	2	1.223	0.62376	0.039811	0.87097	0.53279	0.95153	zinc-binding [Source:HGNC Symbol;Acc:HGNC:910]"	HGLibA_03931;HGLibA_03930
ENSG00000142552	RCN3	1	1.5335	0.30066	0.014969	0.84371	0.76368	0.95644	reticulocalbin 3 [Source:HGNC Symbol;Acc:HGNC:21145]	HGLibA_40707
ENSG00000155897	ADCY8	3	1.4467	0.6359	0.019469	0.84371	0.52484	0.95153	adenylate cyclase 8 [Source:HGNC Symbol;Acc:HGNC:239]	HGLibA_00928;HGLibA_00930;HGLibA_00929
ENSG00000011275	RNF216	3	1.1742	0.72744	0.047479	0.87787	0.46696	0.95153	ring finger protein 216 [Source:HGNC Symbol;Acc:HGNC:21698]	HGLibA_41632;HGLibA_41630;HGLibA_41631
ENSG00000106244	PDAP1	1	1.7163	0.2691	0.0096887	0.84371	0.78785	0.95945	PDGFA associated protein 1 [Source:HGNC Symbol;Acc:HGNC:14634]	HGLibA_35709
ENSG00000106799	TGFBR1	3	1.2506	0.78739	0.036734	0.85944	0.43105	0.95153	transforming growth factor beta receptor 1 [Source:HGNC Symbol;Acc:HGNC:11772]	HGLibA_49223;HGLibA_49224;HGLibA_49222
ENSG00000134962	KLB	2	1.5752	0.45518	0.013592	0.84371	0.64898	0.95153	klotho beta [Source:HGNC Symbol;Acc:HGNC:15527]	HGLibA_25024;HGLibA_25023
ENSG00000139233	LLPH	3	1.2813	0.61685	0.032188	0.84371	0.53733	0.95153	long-term synaptic facilitation factor [Source:HGNC Symbol;Acc:HGNC:28229]"	HGLibA_26655;HGLibA_26654;HGLibA_26653
ENSG00000167123	CERCAM	3	1.6002	0.43839	0.012811	0.84371	0.6611	0.95153	cerebral endothelial cell adhesion molecule [Source:HGNC Symbol;Acc:HGNC:23723]	HGLibA_09191;HGLibA_09192;HGLibA_09190
ENSG00000089818	NECAP1	2	1.436	0.52711	0.020479	0.84371	0.59812	0.95153	NECAP endocytosis associated 1 [Source:HGNC Symbol;Acc:HGNC:24539]	HGLibA_31594;HGLibA_31592
ENSG00000120137	PANK3	2	1.9214	0.42345	0.0062908	0.79902	0.67197	0.95153	pantothenate kinase 3 [Source:HGNC Symbol;Acc:HGNC:19365]	HGLibA_35103;HGLibA_35102
ENSG00000272391	POM121C	3	1.2131	0.5942	0.041648	0.87558	0.55238	0.95153	POM121 transmembrane nucleoporin C [Source:HGNC Symbol;Acc:HGNC:34005]	HGLibA_37644;HGLibA_37646;HGLibA_37645
ENSG00000112343	TRIM38	2	1.3979	0.55306	0.022683	0.84371	0.58022	0.95153	tripartite motif containing 38 [Source:HGNC Symbol;Acc:HGNC:10059]	HGLibA_51479;HGLibA_51480
ENSG00000172315	TP53RK	2	2.1243	0.29752	0.0040408	0.784	0.76607	0.95707	TP53 regulating kinase [Source:HGNC Symbol;Acc:HGNC:16197]	HGLibA_51074;HGLibA_51076
ENSG00000187522	HSPA14	1	1.3527	0.33719	0.025898	0.84371	0.73597	0.95358	heat shock protein family A (Hsp70) member 14 [Source:HGNC Symbol;Acc:HGNC:29526]	HGLibA_22268
hsa-mir-4258	hsa-mir-4258	2	1.2802	0.46244	0.032372	0.84371	0.64377	0.95153	-	HGLibA_59738;HGLibA_59740
ENSG00000100330	MTMR3	3	1.4804	0.59632	0.017724	0.84371	0.55096	0.95153	myotubularin related protein 3 [Source:HGNC Symbol;Acc:HGNC:7451]	HGLibA_30426;HGLibA_30428;HGLibA_30427
ENSG00000100319	ZMAT5	3	1.2426	0.74083	0.037836	0.86555	0.4588	0.95153	zinc finger matrin-type 5 [Source:HGNC Symbol;Acc:HGNC:28046]	HGLibA_55377;HGLibA_55379;HGLibA_55378
FAM208A	FAM208A	1	2.2937	0.18645	0.0030765	0.784	0.85209	0.96994	-	HGLibA_16433
hsa-mir-4433b	hsa-mir-4433b	4	1.1599	0.86925	0.049316	0.87787	0.38471	0.95153	-	HGLibA_60121;HGLibA_60122;HGLibA_60123;HGLibA_60124
ENSG00000198700	IPO9	2	1.3003	0.58069	0.030949	0.84371	0.56145	0.95153	importin 9 [Source:HGNC Symbol;Acc:HGNC:19425]	HGLibA_23479;HGLibA_23477
ENSG00000228716	DHFR	2	1.2738	0.48758	0.032877	0.84371	0.62585	0.95153	dihydrofolate reductase [Source:HGNC Symbol;Acc:HGNC:2861]	HGLibA_13104;HGLibA_13102
ENSG00000181374	CCL13	3	1.3779	0.58899	0.024245	0.84371	0.55587	0.95153	C-C motif chemokine ligand 13 [Source:HGNC Symbol;Acc:HGNC:10611]	HGLibA_07966;HGLibA_07968;HGLibA_07967
ENSG00000125484	GTF3C4	1	1.1851	0.37247	0.04578	0.87787	0.70954	0.95153	general transcription factor IIIC subunit 4 [Source:HGNC Symbol;Acc:HGNC:4667]	HGLibA_20588
ENSG00000166200	COPS2	1	1.194	0.37019	0.044724	0.87787	0.71124	0.95153	COP9 signalosome subunit 2 [Source:HGNC Symbol;Acc:HGNC:30747]	HGLibA_10714
hsa-mir-32	hsa-mir-32	2	1.1595	0.64885	0.049362	0.87787	0.51644	0.95153	-	HGLibA_58905;HGLibA_58904
ENSG00000168234	TTC39C	2	1.5363	0.51862	0.014831	0.84371	0.60403	0.95153	tetratricopeptide repeat domain 39C [Source:HGNC Symbol;Acc:HGNC:26595]	HGLibA_52137;HGLibA_52139
ENSG00000179299	NSUN7	2	1.1785	0.41429	0.046515	0.87787	0.67866	0.95153	NOP2/Sun RNA methyltransferase family member 7 [Source:HGNC Symbol;Acc:HGNC:25857]	HGLibA_32797;HGLibA_32796
ENSG00000239306	RBM14	1	1.8403	0.24992	0.0073928	0.79902	0.80265	0.96046	RNA binding motif protein 14 [Source:HGNC Symbol;Acc:HGNC:14219]	HGLibA_40484
ENSG00000092067	CEBPE	2	1.3319	0.58224	0.027872	0.84371	0.56041	0.95153	CCAAT enhancer binding protein epsilon [Source:HGNC Symbol;Acc:HGNC:1836]	HGLibA_08966;HGLibA_08965
ENSG00000110768	GTF2H1	2	1.5368	0.50973	0.01474	0.84371	0.61024	0.95153	general transcription factor IIH subunit 1 [Source:HGNC Symbol;Acc:HGNC:4655]	HGLibA_20545;HGLibA_20544
ENSG00000003249	DBNDD1	3	1.2538	0.59384	0.036275	0.8559	0.55262	0.95153	dysbindin domain containing 1 [Source:HGNC Symbol;Acc:HGNC:28455]	HGLibA_12392;HGLibA_12390;HGLibA_12391
ENSG00000188761	BCL2L15	2	1.2439	0.48298	0.037561	0.86287	0.62911	0.95153	BCL2 like 15 [Source:HGNC Symbol;Acc:HGNC:33624]	HGLibA_04321;HGLibA_04319
ENSG00000150269	OR5M9	2	2.8279	0.17556	0.0012857	0.725	0.86064	0.9721	olfactory receptor family 5 subfamily M member 9 [Source:HGNC Symbol;Acc:HGNC:15294]	HGLibA_34344;HGLibA_34343
ENSG00000180198	RCC1	2	2.2363	0.32106	0.003352	0.784	0.74816	0.95525	regulator of chromosome condensation 1 [Source:HGNC Symbol;Acc:HGNC:1913]	HGLibA_40682;HGLibA_40684
ENSG00000197016	ZNF470	3	1.2421	0.6936	0.037836	0.86555	0.48793	0.95153	zinc finger protein 470 [Source:HGNC Symbol;Acc:HGNC:22220]	HGLibA_56054;HGLibA_56053;HGLibA_56052
ENSG00000160753	RUSC1	3	1.2953	0.6072	0.03127	0.84371	0.54372	0.95153	RUN and SH3 domain containing 1 [Source:HGNC Symbol;Acc:HGNC:17153]	HGLibA_42466;HGLibA_42468;HGLibA_42467
ENSG00000141510	TP53	3	1.833	0.54093	0.0074846	0.79902	0.58856	0.95153	tumor protein p53 [Source:HGNC Symbol;Acc:HGNC:11998]	HGLibA_51048;HGLibA_51047;HGLibA_51049
ENSG00000168564	CDKN2AIP	2	1.6173	0.49484	0.01249	0.84371	0.62071	0.95153	CDKN2A interacting protein [Source:HGNC Symbol;Acc:HGNC:24325]	HGLibA_08833;HGLibA_08832
ENSG00000135241	PNPLA8	3	1.3012	0.73326	0.030811	0.84371	0.4634	0.95153	patatin like phospholipase domain containing 8 [Source:HGNC Symbol;Acc:HGNC:28900]	HGLibA_37426;HGLibA_37425;HGLibA_37427
ENSG00000125534	PPDPF	2	2.5786	0.247	0.0019745	0.76812	0.80491	0.96046	pancreatic progenitor cell differentiation and proliferation factor [Source:HGNC Symbol;Acc:HGNC:16142]	HGLibA_37865;HGLibA_37864
ENSG00000118113	MMP8	3	1.8318	0.4525	0.0074846	0.79902	0.65091	0.95153	matrix metallopeptidase 8 [Source:HGNC Symbol;Acc:HGNC:7175]	HGLibA_29540;HGLibA_29541;HGLibA_29539
ENSG00000009724	MASP2	3	1.4197	0.64244	0.021444	0.84371	0.52059	0.95153	mannan binding lectin serine peptidase 2 [Source:HGNC Symbol;Acc:HGNC:6902]	HGLibA_28397;HGLibA_28396;HGLibA_28398
ENSG00000167881	SRP68	3	1.9924	0.41645	0.0054642	0.79902	0.67708	0.95153	signal recognition particle 68 [Source:HGNC Symbol;Acc:HGNC:11302]	HGLibA_47010;HGLibA_47008;HGLibA_47009
ENSG00000154764	WNT7A	2	1.4281	0.51152	0.020939	0.84371	0.60899	0.95153	Wnt family member 7A [Source:HGNC Symbol;Acc:HGNC:12786]	HGLibA_54383;HGLibA_54382
ENSG00000175216	CKAP5	3	1.3707	0.61489	0.024612	0.84371	0.53863	0.95153	cytoskeleton associated protein 5 [Source:HGNC Symbol;Acc:HGNC:28959]	HGLibA_09763;HGLibA_09762;HGLibA_09764
ENSG00000109181	UGT2B10	3	1.2876	0.75918	0.031775	0.84371	0.44774	0.95153	UDP glucuronosyltransferase family 2 member B10 [Source:HGNC Symbol;Acc:HGNC:12544]	HGLibA_52911;HGLibA_52913;HGLibA_52912
ENSG00000134186	PRPF38B	2	3.6578	0.12087	0.00041326	0.5625	0.90379	0.98065	pre-mRNA processing factor 38B [Source:HGNC Symbol;Acc:HGNC:25512]	HGLibA_38725;HGLibA_38724
ENSG00000109919	MTCH2	3	1.159	0.78827	0.049408	0.87787	0.43054	0.95153	mitochondrial carrier 2 [Source:HGNC Symbol;Acc:HGNC:17587]	HGLibA_30328;HGLibA_30327;HGLibA_30329
ENSG00000141570	CBX8	2	2.001	0.33432	0.0053724	0.79902	0.73814	0.95387	chromobox 8 [Source:HGNC Symbol;Acc:HGNC:15962]	HGLibA_07465;HGLibA_07467
ENSG00000139631	CSAD	3	1.2771	0.59617	0.032785	0.84371	0.55106	0.95153	cysteine sulfinic acid decarboxylase [Source:HGNC Symbol;Acc:HGNC:18966]	HGLibA_11303;HGLibA_11304;HGLibA_11302
ENSG00000172534	HCFC1	2	1.5093	0.3754	0.015796	0.84371	0.70736	0.95153	host cell factor C1 [Source:HGNC Symbol;Acc:HGNC:4839]	HGLibA_20959;HGLibA_20957
ENSG00000196860	TOMM20L	3	1.4616	0.55169	0.01878	0.84371	0.58116	0.95153	translocase of outer mitochondrial membrane 20 like [Source:HGNC Symbol;Acc:HGNC:33752]	HGLibA_50958;HGLibA_50957;HGLibA_50959
ENSG00000138698	RAP1GDS1	3	1.9252	0.38368	0.0061989	0.79902	0.70122	0.95153	Rap1 GTPase-GDP dissociation stimulator 1 [Source:HGNC Symbol;Acc:HGNC:9859]	HGLibA_40233;HGLibA_40232;HGLibA_40234
ENSG00000145982	FARS2	1	1.2164	0.36733	0.041188	0.87512	0.71338	0.95202	mitochondrial [Source:HGNC Symbol;Acc:HGNC:21062]"	HGLibA_16879
WDR16	WDR16	3	1.2637	0.63773	0.034393	0.85011	0.52365	0.95153	-	HGLibA_54033;HGLibA_54035;HGLibA_54034
C10orf32	C10orf32	2	1.4482	0.53503	0.019423	0.84371	0.59263	0.95153	-	HGLibA_05067;HGLibA_05068
ENSG00000164163	ABCE1	2	1.7258	0.38808	0.0093673	0.84371	0.69796	0.95153	ATP binding cassette subfamily E member 1 [Source:HGNC Symbol;Acc:HGNC:69]	HGLibA_00202;HGLibA_00203
ENSG00000124784	RIOK1	3	1.6926	0.47824	0.010194	0.84371	0.63248	0.95153	RIO kinase 1 [Source:HGNC Symbol;Acc:HGNC:18656]	HGLibA_41307;HGLibA_41308;HGLibA_41306
ENSG00000149489	ROM1	3	1.2946	0.62088	0.03127	0.84371	0.53468	0.95153	retinal outer segment membrane protein 1 [Source:HGNC Symbol;Acc:HGNC:10254]	HGLibA_41755;HGLibA_41753;HGLibA_41754
hsa-mir-4509-3	hsa-mir-4509-3	3	1.2418	0.63624	0.037928	0.86674	0.52462	0.95153	-	HGLibA_60471;HGLibA_60470;HGLibA_60472
ENSG00000175602	CCDC85B	2	1.5144	0.37087	0.015612	0.84371	0.71073	0.95153	coiled-coil domain containing 85B [Source:HGNC Symbol;Acc:HGNC:24926]	HGLibA_07895;HGLibA_07896
ENSG00000176102	CSTF3	1	2.5982	0.15372	0.0017908	0.76812	0.87783	0.9752	cleavage stimulation factor subunit 3 [Source:HGNC Symbol;Acc:HGNC:2485]	HGLibA_11491
ENSG00000103502	CDIPT	2	2.0718	0.28296	0.0045459	0.78571	0.7772	0.95833	CDP-diacylglycerol--inositol 3-phosphatidyltransferase [Source:HGNC Symbol;Acc:HGNC:1769]	HGLibA_08717;HGLibA_08715
hsa-mir-329-1	hsa-mir-329-1	3	1.2466	0.52242	0.037148	0.85956	0.60138	0.95153	-	HGLibA_58979;HGLibA_58981;HGLibA_58978
ENSG00000136810	TXN	3	1.2579	0.5762	0.035449	0.85525	0.56448	0.95153	thioredoxin [Source:HGNC Symbol;Acc:HGNC:12435]	HGLibA_52399;HGLibA_52398;HGLibA_52400
ENSG00000178776	C5orf46	3	1.2208	0.71418	0.040316	0.87097	0.47511	0.95153	chromosome 5 open reading frame 46 [Source:HGNC Symbol;Acc:HGNC:33768]	HGLibA_06389;HGLibA_06390;HGLibA_06388
C16orf59	C16orf59	2	1.17	0.42963	0.047755	0.87787	0.66746	0.95153	-	HGLibA_05464;HGLibA_05466
ENSG00000120705	ETF1	2	1.9667	0.3468	0.0056479	0.79902	0.72874	0.95266	eukaryotic translation termination factor 1 [Source:HGNC Symbol;Acc:HGNC:3477]	HGLibA_15617;HGLibA_15616
ENSG00000214026	MRPL23	3	1.8723	0.45222	0.0067958	0.79902	0.65111	0.95153	mitochondrial ribosomal protein L23 [Source:HGNC Symbol;Acc:HGNC:10322]	HGLibA_29934;HGLibA_29933;HGLibA_29932
ENSG00000128274	A4GALT	3	1.1565	0.75994	0.049867	0.87787	0.44729	0.95153	4-galactosyltransferase (P blood group) [Source:HGNC Symbol;Acc:HGNC:18149]"	HGLibA_00017;HGLibA_00018;HGLibA_00016
ENSG00000196152	ZNF79	3	1.3346	0.72065	0.027551	0.84371	0.47113	0.95153	zinc finger protein 79 [Source:HGNC Symbol;Acc:HGNC:13153]	HGLibA_56760;HGLibA_56762;HGLibA_56761
ENSG00000164091	WDR82	2	1.4975	0.44469	0.01676	0.84371	0.65654	0.95153	WD repeat domain 82 [Source:HGNC Symbol;Acc:HGNC:28826]	HGLibA_54191;HGLibA_54190
ENSG00000112852	PCDHB2	3	1.1744	0.79316	0.047433	0.87787	0.42768	0.95153	protocadherin beta 2 [Source:HGNC Symbol;Acc:HGNC:8687]	HGLibA_35494;HGLibA_35493;HGLibA_35492
ENSG00000166295	ANAPC16	2	1.6701	0.46561	0.010837	0.84371	0.6415	0.95153	anaphase promoting complex subunit 16 [Source:HGNC Symbol;Acc:HGNC:26976]	HGLibA_01876;HGLibA_01878
ENSG00000228049	POLR2J2	2	1.4306	0.49264	0.020847	0.84371	0.62227	0.95153	RNA polymerase II subunit J2 [Source:HGNC Symbol;Acc:HGNC:23208]	HGLibA_37594;HGLibA_37593
ENSG00000146352	CLVS2	2	1.3837	0.70268	0.023877	0.84371	0.48225	0.95153	clavesin 2 [Source:HGNC Symbol;Acc:HGNC:23046]	HGLibA_10171;HGLibA_10170;HGLibA_10169
ENSG00000140265	ZSCAN29	2	3.0408	0.18156	0.00087244	0.6129	0.85593	0.97087	zinc finger and SCAN domain containing 29 [Source:HGNC Symbol;Acc:HGNC:26673]	HGLibA_57013;HGLibA_57012
ENSG00000140264	SERF2	2	1.6448	0.48138	0.011388	0.84371	0.63025	0.95153	small EDRK-rich factor 2 [Source:HGNC Symbol;Acc:HGNC:10757]	HGLibA_43476;HGLibA_43475
ENSG00000162545	CAMK2N1	3	1.3866	0.70638	0.023648	0.84371	0.47995	0.95153	calcium/calmodulin dependent protein kinase II inhibitor 1 [Source:HGNC Symbol;Acc:HGNC:24190]	HGLibA_07079;HGLibA_07080;HGLibA_07078
hsa-mir-548s	hsa-mir-548s	3	2.7573	0.27314	0.0013775	0.73171	0.78475	0.95886	-	HGLibA_62063;HGLibA_62064;HGLibA_62062
ENSG00000173530	TNFRSF10D	2	3.0349	0.19804	0.00087244	0.6129	0.84301	0.96734	TNF receptor superfamily member 10d [Source:HGNC Symbol;Acc:HGNC:11907]	HGLibA_50741;HGLibA_50743
ENSG00000162039	MEIOB	3	1.2736	0.6594	0.032969	0.84371	0.50964	0.95153	meiosis specific with OB domains [Source:HGNC Symbol;Acc:HGNC:28569]	HGLibA_28869;HGLibA_28868;HGLibA_28867
ENSG00000140015	KCNH5	2	1.5577	0.3831	0.014097	0.84371	0.70164	0.95153	potassium voltage-gated channel subfamily H member 5 [Source:HGNC Symbol;Acc:HGNC:6254]	HGLibA_24223;HGLibA_24222
ENSG00000060069	CTDP1	2	3.2779	0.16544	0.00059693	0.59091	0.86859	0.9741	CTD phosphatase subunit 1 [Source:HGNC Symbol;Acc:HGNC:2498]	HGLibA_11606;HGLibA_11604
ENSG00000162521	RBBP4	3	1.2249	0.4908	0.039489	0.87097	0.62357	0.95153	chromatin remodeling factor [Source:HGNC Symbol;Acc:HGNC:9887]"	HGLibA_40427;HGLibA_40429;HGLibA_40428
HIST2H3C	HIST2H3C	2	1.5027	0.41507	0.016255	0.84371	0.67809	0.95153	-	HGLibA_21543;HGLibA_21542
VARS	VARS	3	1.5018	0.4909	0.016347	0.84371	0.6235	0.95153	-	HGLibA_53525;HGLibA_53523;HGLibA_53524
ENSG00000163531	NFASC	3	1.3676	0.68237	0.024979	0.84371	0.495	0.95153	neurofascin [Source:HGNC Symbol;Acc:HGNC:29866]	HGLibA_31767;HGLibA_31768;HGLibA_31766
ENSG00000127561	SYNGR3	3	1.4012	0.67941	0.0225	0.84371	0.49688	0.95153	synaptogyrin 3 [Source:HGNC Symbol;Acc:HGNC:11501]	HGLibA_47997;HGLibA_47996;HGLibA_47998
ENSG00000162959	MEMO1	2	1.7545	0.44541	0.0088622	0.84371	0.65603	0.95153	mediator of cell motility 1 [Source:HGNC Symbol;Acc:HGNC:14014]	HGLibA_28882;HGLibA_28883
ENSG00000025423	HSD17B6	2	1.3444	0.58068	0.026724	0.84371	0.56145	0.95153	hydroxysteroid 17-beta dehydrogenase 6 [Source:HGNC Symbol;Acc:HGNC:23316]	HGLibA_22196;HGLibA_22197;HGLibA_22195
ENSG00000122958	VPS26A	2	1.2457	0.57139	0.037331	0.85956	0.56774	0.95153	retromer complex component A [Source:HGNC Symbol;Acc:HGNC:12711]"	HGLibA_53751;HGLibA_53753
ENSG00000111700	SLCO1B3	1	1.4848	0.31683	0.017311	0.84371	0.75137	0.95562	solute carrier organic anion transporter family member 1B3 [Source:HGNC Symbol;Acc:HGNC:10961]	HGLibA_45452
ENSG00000214113	LYRM4	2	1.1578	0.64708	0.049545	0.87787	0.51758	0.95153	LYR motif containing 4 [Source:HGNC Symbol;Acc:HGNC:21365]	HGLibA_27752;HGLibA_27753
ITFG3	ITFG3	2	1.2505	0.4122	0.036734	0.85944	0.68019	0.95153	-	HGLibA_23713;HGLibA_23712
hsa-mir-548o-2	hsa-mir-548o-2	4	1.1561	0.74181	0.049867	0.87787	0.4582	0.95153	-	HGLibA_62054;HGLibA_62051;HGLibA_62052;HGLibA_62053
ENSG00000128965	CHAC1	3	1.222	0.62943	0.039994	0.87097	0.52907	0.95153	ChaC glutathione specific gamma-glutamylcyclotransferase 1 [Source:HGNC Symbol;Acc:HGNC:28680]	HGLibA_09336;HGLibA_09335;HGLibA_09334
ENSG00000036565	SLC18A1	3	1.837	0.55354	0.0074387	0.79902	0.57989	0.95153	solute carrier family 18 member A1 [Source:HGNC Symbol;Acc:HGNC:10934]	HGLibA_44486;HGLibA_44487;HGLibA_44485
ENSG00000154127	UBASH3B	3	1.4246	0.58932	0.021306	0.84371	0.55565	0.95153	ubiquitin associated and SH3 domain containing B [Source:HGNC Symbol;Acc:HGNC:29884]	HGLibA_52562;HGLibA_52561;HGLibA_52560
ENSG00000011376	LARS2	1	3.0806	0.11302	0.00087244	0.6129	0.91001	0.98129	mitochondrial [Source:HGNC Symbol;Acc:HGNC:17095]"	HGLibA_26050
ENSG00000124193	SRSF6	3	1.4792	0.49896	0.017908	0.84371	0.61781	0.95153	serine and arginine rich splicing factor 6 [Source:HGNC Symbol;Acc:HGNC:10788]	HGLibA_47084;HGLibA_47083;HGLibA_47085
ENSG00000269190	FBXO17	3	1.2758	0.73879	0.032877	0.84371	0.46003	0.95153	F-box protein 17 [Source:HGNC Symbol;Acc:HGNC:18754]	HGLibA_17037;HGLibA_17035;HGLibA_17036
ENSG00000213672	NCKIPSD	3	1.368	0.7349	0.024933	0.84371	0.4624	0.95153	NCK interacting protein with SH3 domain [Source:HGNC Symbol;Acc:HGNC:15486]	HGLibA_31323;HGLibA_31324;HGLibA_31322
ENSG00000124279	FASTKD3	2	1.4659	0.5423	0.018505	0.84371	0.58761	0.95153	FAST kinase domains 3 [Source:HGNC Symbol;Acc:HGNC:28758]	HGLibA_16906;HGLibA_16908
ENSG00000189090	FAM25G	3	1.3252	0.72319	0.028515	0.84371	0.46956	0.95153	family with sequence similarity 25 member G [Source:HGNC Symbol;Acc:HGNC:23590]	HGLibA_16557;HGLibA_16558;HGLibA_16559
ENSG00000277161	PIGW	1	2.6809	0.14402	0.0015153	0.76744	0.88549	0.97613	phosphatidylinositol glycan anchor biosynthesis class W [Source:HGNC Symbol;Acc:HGNC:23213]	HGLibA_36598
RGAG1	RGAG1	2	1.1813	0.61552	0.046193	0.87787	0.53821	0.95153	-	HGLibA_40992;HGLibA_40991
ENSG00000136383	ALPK3	1	1.403	0.32653	0.022454	0.84371	0.74402	0.95475	alpha kinase 3 [Source:HGNC Symbol;Acc:HGNC:17574]	HGLibA_01705
ENSG00000101057	MYBL2	3	2.1067	0.40614	0.0041785	0.784	0.68464	0.95153	MYB proto-oncogene like 2 [Source:HGNC Symbol;Acc:HGNC:7548]	HGLibA_30646;HGLibA_30645;HGLibA_30647
hsa-mir-635	hsa-mir-635	4	1.4017	0.76074	0.0225	0.84371	0.44681	0.95153	-	HGLibA_62778;HGLibA_62779;HGLibA_62781;HGLibA_62780
ENSG00000112096	SOD2	2	1.6249	0.36619	0.01226	0.84371	0.71422	0.95225	superoxide dismutase 2 [Source:HGNC Symbol;Acc:HGNC:11180]	HGLibA_46147;HGLibA_46146
ENSG00000130638	ATXN10	3	1.3196	0.6496	0.029387	0.84371	0.51595	0.95153	ataxin 10 [Source:HGNC Symbol;Acc:HGNC:10549]	HGLibA_03831;HGLibA_03830;HGLibA_03832
ENSG00000139053	PDE6H	2	1.655	0.43934	0.011158	0.84371	0.66041	0.95153	phosphodiesterase 6H [Source:HGNC Symbol;Acc:HGNC:8790]	HGLibA_35819;HGLibA_35821
ENSG00000197646	PDCD1LG2	3	1.3102	0.49951	0.030076	0.84371	0.61742	0.95153	programmed cell death 1 ligand 2 [Source:HGNC Symbol;Acc:HGNC:18731]	HGLibA_35724;HGLibA_35723;HGLibA_35725
MKI67IP	MKI67IP	2	4.3577	0.078203	0.00027551	0.5625	0.93767	0.98633	-	HGLibA_29328;HGLibA_29327
ENSG00000153989	NUS1	2	2.1967	0.33175	0.0037193	0.784	0.74008	0.95438	dehydrodolichyl diphosphate synthase subunit [Source:HGNC Symbol;Acc:HGNC:21042]"	HGLibA_33093;HGLibA_33092
ENSG00000189409	MMP23B	3	1.2498	0.58913	0.03678	0.85944	0.55577	0.95153	matrix metallopeptidase 23B [Source:HGNC Symbol;Acc:HGNC:7171]	HGLibA_29515;HGLibA_29516;HGLibA_29517
CCDC94	CCDC94	3	1.2227	0.7749	0.039949	0.87097	0.4384	0.95153	-	HGLibA_07934;HGLibA_07935;HGLibA_07933
hsa-mir-4426	hsa-mir-4426	3	1.7705	0.47596	0.0084948	0.84371	0.6341	0.95153	-	HGLibA_60090;HGLibA_60091;HGLibA_60092
ENSG00000165512	ZNF22	3	1.3585	0.73665	0.025484	0.84371	0.46134	0.95153	zinc finger protein 22 [Source:HGNC Symbol;Acc:HGNC:13012]	HGLibA_55605;HGLibA_55606;HGLibA_55607
ENSG00000089123	TASP1	3	1.1657	0.57808	0.048443	0.87787	0.56321	0.95153	taspase 1 [Source:HGNC Symbol;Acc:HGNC:15859]	HGLibA_48434;HGLibA_48435;HGLibA_48436
ENSG00000064309	CDON	3	1.2455	0.74265	0.037331	0.85956	0.45769	0.95153	oncogene regulated [Source:HGNC Symbol;Acc:HGNC:17104]"	HGLibA_08858;HGLibA_08857;HGLibA_08856
ENSG00000151229	SLC2A13	2	1.6047	0.38867	0.012673	0.84371	0.69752	0.95153	solute carrier family 2 member 13 [Source:HGNC Symbol;Acc:HGNC:15956]	HGLibA_44863;HGLibA_44865
ENSG00000115524	SF3B1	2	2.2854	0.264	0.0031224	0.784	0.79178	0.9595	splicing factor 3b subunit 1 [Source:HGNC Symbol;Acc:HGNC:10768]	HGLibA_43701;HGLibA_43699
IL9R_X	IL9R_X	3	1.4622	0.5996	0.018689	0.84371	0.54878	0.95153	-	HGLibA_23203;HGLibA_23202;HGLibA_23201
ENSG00000149231	CCDC82	2	1.1927	0.5194	0.045091	0.87787	0.60348	0.95153	coiled-coil domain containing 82 [Source:HGNC Symbol;Acc:HGNC:26282]	HGLibA_07883;HGLibA_07882
ENSG00000186479	RGS7BP	3	1.2204	0.68981	0.040408	0.87097	0.49032	0.95153	regulator of G protein signaling 7 binding protein [Source:HGNC Symbol;Acc:HGNC:23271]	HGLibA_41105;HGLibA_41106;HGLibA_41107
ENSG00000110583	NAA40	3	1.5232	0.6061	0.015428	0.84371	0.54445	0.95153	NatD catalytic subunit [Source:HGNC Symbol;Acc:HGNC:25845]"	HGLibA_30996;HGLibA_30997;HGLibA_30998
ENSG00000105696	TMEM59L	3	1.2012	0.73724	0.043438	0.87616	0.46098	0.95153	transmembrane protein 59 like [Source:HGNC Symbol;Acc:HGNC:13237]	HGLibA_50463;HGLibA_50462;HGLibA_50461
TOMM70A	TOMM70A	2	1.6168	0.46766	0.01249	0.84371	0.64003	0.95153	-	HGLibA_50981;HGLibA_50982
ENSG00000198736	MSRB1	2	1.1872	0.63767	0.045642	0.87787	0.52369	0.95153	methionine sulfoxide reductase B1 [Source:HGNC Symbol;Acc:HGNC:14133]	HGLibA_30241;HGLibA_30240
ENSG00000253710	ALG11	2	1.2093	0.50693	0.04229	0.87558	0.61221	0.95153	2-mannosyltransferase [Source:HGNC Symbol;Acc:HGNC:32456]"	HGLibA_01611;HGLibA_01609
ENSG00000132432	SEC61G	1	1.3909	0.33042	0.023189	0.84371	0.74108	0.95441	Sec61 translocon gamma subunit [Source:HGNC Symbol;Acc:HGNC:18277]	HGLibA_43256
ENSG00000186281	GPAT2	3	1.1604	0.6568	0.04927	0.87787	0.51131	0.95153	mitochondrial [Source:HGNC Symbol;Acc:HGNC:27168]"	HGLibA_19733;HGLibA_19732;HGLibA_19731
ENSG00000089195	TRMT6	2	1.2184	0.4476	0.040637	0.87097	0.65444	0.95153	tRNA methyltransferase 6 [Source:HGNC Symbol;Acc:HGNC:20900]	HGLibA_51695;HGLibA_51696
ENSG00000101981	F9	3	1.4931	0.4885	0.016852	0.84371	0.6252	0.95153	coagulation factor IX [Source:HGNC Symbol;Acc:HGNC:3551]	HGLibA_15896;HGLibA_15894;HGLibA_15895
ENSG00000073711	PPP2R3A	3	2.4281	0.30801	0.0023877	0.76812	0.75808	0.95596	protein phosphatase 2 regulatory subunit B''alpha [Source:HGNC Symbol;Acc:HGNC:9307]	HGLibA_38154;HGLibA_38156;HGLibA_38155
ENSG00000131966	ACTR10	2	2.2972	0.31735	0.0030765	0.784	0.75098	0.95562	actin related protein 10 homolog [Source:HGNC Symbol;Acc:HGNC:17372]	HGLibA_00647;HGLibA_00646
ENSG00000170631	ZNF16	1	1.3706	0.33275	0.024658	0.84371	0.73932	0.95438	zinc finger protein 16 [Source:HGNC Symbol;Acc:HGNC:12947]	HGLibA_55509
ENSG00000214530	STARD10	3	1.2093	0.73201	0.04229	0.87558	0.46416	0.95153	StAR related lipid transfer domain containing 10 [Source:HGNC Symbol;Acc:HGNC:10666]	HGLibA_47350;HGLibA_47352;HGLibA_47351
hsa-mir-6087	hsa-mir-6087	1	1.8626	0.24686	0.0071173	0.79902	0.80501	0.96046	-	HGLibA_62609
ENSG00000155926	SLA	2	1.2179	0.49379	0.040637	0.87097	0.62146	0.95153	Src like adaptor [Source:HGNC Symbol;Acc:HGNC:10902]	HGLibA_44300;HGLibA_44301
ENSG00000116922	C1orf109	2	1.1933	0.52321	0.044954	0.87787	0.60083	0.95153	chromosome 1 open reading frame 109 [Source:HGNC Symbol;Acc:HGNC:26039]	HGLibA_05742;HGLibA_05740
ENSG00000176890	TYMS	2	1.5004	0.41784	0.016485	0.84371	0.67606	0.95153	thymidylate synthetase [Source:HGNC Symbol;Acc:HGNC:12441]	HGLibA_52462;HGLibA_52461
ENSG00000182348	ZNF804B	2	1.865	0.4205	0.0071173	0.79902	0.67412	0.95153	zinc finger protein 804B [Source:HGNC Symbol;Acc:HGNC:21958]	HGLibA_56790;HGLibA_56791
AGAP7	AGAP7	3	1.2424	0.73696	0.037836	0.86555	0.46115	0.95153	-	HGLibA_01156;HGLibA_01158;HGLibA_01157
ENSG00000130940	CASZ1	3	1.4857	0.66562	0.017311	0.84371	0.50565	0.95153	castor zinc finger 1 [Source:HGNC Symbol;Acc:HGNC:26002]	HGLibA_07349;HGLibA_07350;HGLibA_07348
hsa-mir-4472-2	hsa-mir-4472-2	1	4.5602	0.038786	0.00013775	0.5	0.96906	0.99326	-	HGLibA_60299
AGAP8	AGAP8	3	1.3775	0.65894	0.024291	0.84371	0.50993	0.95153	-	HGLibA_01160;HGLibA_01159;HGLibA_01161
ENSG00000126858	RHOT1	2	2.3389	0.30509	0.002801	0.784	0.7603	0.95605	ras homolog family member T1 [Source:HGNC Symbol;Acc:HGNC:21168]	HGLibA_41201;HGLibA_41202
ENSG00000188312	CENPP	3	2.3234	0.37964	0.0030306	0.784	0.70421	0.95153	centromere protein P [Source:HGNC Symbol;Acc:HGNC:32933]	HGLibA_09081;HGLibA_09080;HGLibA_09079
LOC100129924	LOC100129924	3	1.697	0.5438	0.010056	0.84371	0.58658	0.95153	-	HGLibA_26772;HGLibA_26771;HGLibA_26770
ENSG00000119616	FCF1	3	1.5462	0.5864	0.014464	0.84371	0.5576	0.95153	rRNA-processing protein [Source:HGNC Symbol;Acc:HGNC:20220]"	HGLibA_17178;HGLibA_17177;HGLibA_17176
ENSG00000179922	ZNF784	2	1.3	0.48288	0.030949	0.84371	0.62918	0.95153	zinc finger protein 784 [Source:HGNC Symbol;Acc:HGNC:33111]	HGLibA_56745;HGLibA_56747
ENSG00000214253	FIS1	2	1.4011	0.52826	0.0225	0.84371	0.59732	0.95153	mitochondrial 1 [Source:HGNC Symbol;Acc:HGNC:21689]"	HGLibA_17536;HGLibA_17538
ENSG00000198826	ARHGAP11A	2	1.1967	0.59363	0.044403	0.87787	0.55276	0.95153	Rho GTPase activating protein 11A [Source:HGNC Symbol;Acc:HGNC:15783]	HGLibA_02701;HGLibA_02702
ENSG00000033100	CHPF2	3	1.2545	0.6845	0.036229	0.8559	0.49366	0.95153	chondroitin polymerizing factor 2 [Source:HGNC Symbol;Acc:HGNC:29270]	HGLibA_09527;HGLibA_09526;HGLibA_09525
ENSG00000134343	ANO3	3	1.2375	0.59377	0.038204	0.86735	0.55267	0.95153	anoctamin 3 [Source:HGNC Symbol;Acc:HGNC:14004]	HGLibA_02176;HGLibA_02178;HGLibA_02177
ENSG00000177432	NAP1L5	2	1.427	0.44873	0.021122	0.84371	0.65362	0.95153	nucleosome assembly protein 1 like 5 [Source:HGNC Symbol;Acc:HGNC:19968]	HGLibA_31124;HGLibA_31123
ENSG00000189169	KRTAP10-12	2	1.2622	0.529	0.034438	0.85011	0.5968	0.95153	keratin associated protein 10-12 [Source:HGNC Symbol;Acc:HGNC:20533]	HGLibA_25599;HGLibA_25600
ENSG00000011260	UTP18	2	1.5849	0.43434	0.013316	0.84371	0.66404	0.95153	small subunit processome component [Source:HGNC Symbol;Acc:HGNC:24274]"	HGLibA_53443;HGLibA_53444
ENSG00000172426	RSPH9	2	2.1335	0.31699	0.0040408	0.784	0.75125	0.95562	radial spoke head 9 homolog [Source:HGNC Symbol;Acc:HGNC:21057]	HGLibA_42342;HGLibA_42340
hsa-mir-6835	hsa-mir-6835	2	1.4292	0.50161	0.020939	0.84371	0.61594	0.95153	-	HGLibA_63517;HGLibA_63516
HMHA1	HMHA1	1	1.5041	0.30834	0.016117	0.84371	0.75782	0.95596	-	HGLibA_21738
C19orf57	C19orf57	3	1.3206	0.69059	0.029204	0.84371	0.48982	0.95153	-	HGLibA_05684;HGLibA_05685;HGLibA_05683
ENSG00000077380	DYNC1I2	2	1.2577	0.60905	0.035495	0.85525	0.54249	0.95153	dynein cytoplasmic 1 intermediate chain 2 [Source:HGNC Symbol;Acc:HGNC:2964]	HGLibA_14237;HGLibA_14238
ENSG00000134759	ELP2	3	1.4686	0.66697	0.018138	0.84371	0.50479	0.95153	elongator acetyltransferase complex subunit 2 [Source:HGNC Symbol;Acc:HGNC:18248]	HGLibA_14977;HGLibA_14978;HGLibA_14979
ENSG00000140829	DHX38	2	1.8046	0.37844	0.0079438	0.82775	0.7051	0.95153	DEAH-box helicase 38 [Source:HGNC Symbol;Acc:HGNC:17211]	HGLibA_13195;HGLibA_13196
ENSG00000111605	CPSF6	2	1.4699	0.48241	0.018138	0.84371	0.62951	0.95153	cleavage and polyadenylation specific factor 6 [Source:HGNC Symbol;Acc:HGNC:13871]	HGLibA_11011;HGLibA_11013
ENSG00000133704	IPO8	3	1.2206	0.55939	0.040362	0.87097	0.5759	0.95153	importin 8 [Source:HGNC Symbol;Acc:HGNC:9853]	HGLibA_23475;HGLibA_23474;HGLibA_23476
ENSG00000117020	AKT3	3	1.292	0.57078	0.031546	0.84371	0.56815	0.95153	AKT serine/threonine kinase 3 [Source:HGNC Symbol;Acc:HGNC:393]	HGLibA_01514;HGLibA_01515;HGLibA_01513

hsa-mir-4483	hsa-mir-4483	3	1.5111	0.57098	0.015704	0.84371	0.56801	0.95153	-	HGLibA_60350;HGLibA_60351;HGLibA_60352
hsa-mir-4509-1	hsa-mir-4509-1	3	1.1777	0.67157	0.04679	0.87787	0.50186	0.95153	-	HGLibA_60463;HGLibA_60464;HGLibA_60462
ENSG00000179262	RAD23A	2	1.2395	0.50219	0.03802	0.86702	0.61553	0.95153	nucleotide excision repair protein [Source:HGNC Symbol;Acc:HGNC:9812]"	HGLibA_40070;HGLibA_40068
ENSG00000185105	MYADML2	2	1.5367	0.37872	0.014786	0.84371	0.7049	0.95153	myeloid associated differentiation marker like 2 [Source:HGNC Symbol;Acc:HGNC:34548]	HGLibA_30635;HGLibA_30633
ENSG00000109171	SLAIN2	3	1.583	0.56216	0.013362	0.84371	0.57401	0.95153	SLAIN motif family member 2 [Source:HGNC Symbol;Acc:HGNC:29282]	HGLibA_44309;HGLibA_44308;HGLibA_44310
ENSG00000047365	ARAP2	3	1.3288	0.70188	0.028194	0.84371	0.48276	0.95153	ankyrin repeat and PH domain 2 [Source:HGNC Symbol;Acc:HGNC:16924]"	HGLibA_02628;HGLibA_02627;HGLibA_02629
ENSG00000188725	SMIM15	2	2.6584	0.25056	0.001653	0.76812	0.80216	0.96046	small integral membrane protein 15 [Source:HGNC Symbol;Acc:HGNC:33861]	HGLibA_45733;HGLibA_45734
ENSG00000138768	USO1	2	1.4428	0.51136	0.019837	0.84371	0.6091	0.95153	USO1 vesicle transport factor [Source:HGNC Symbol;Acc:HGNC:30904]	HGLibA_53188;HGLibA_53189
ENSG00000106328	FSCN3	3	1.2177	0.76186	0.040637	0.87097	0.44614	0.95153	fascin actin-bundling protein 3 [Source:HGNC Symbol;Acc:HGNC:3961]	HGLibA_18073;HGLibA_18075;HGLibA_18074
ENSG00000146576	C7orf26	2	1.3559	0.51328	0.025668	0.84371	0.60776	0.95153	chromosome 7 open reading frame 26 [Source:HGNC Symbol;Acc:HGNC:21702]	HGLibA_06528;HGLibA_06527
ENSG00000113273	ARSB	2	1.4326	0.54371	0.020663	0.84371	0.58664	0.95153	arylsulfatase B [Source:HGNC Symbol;Acc:HGNC:714]	HGLibA_03131;HGLibA_03132
ENSG00000130176	CNN1	3	1.8429	0.46095	0.0073469	0.79902	0.64484	0.95153	calponin 1 [Source:HGNC Symbol;Acc:HGNC:2155]	HGLibA_10306;HGLibA_10305;HGLibA_10304
ENSG00000100209	HSCB	2	1.5938	0.47849	0.013087	0.84371	0.6323	0.95153	HscB mitochondrial iron-sulfur cluster cochaperone [Source:HGNC Symbol;Acc:HGNC:28913]	HGLibA_22156;HGLibA_22158
ENSG00000174842	GLMN	2	1.1889	0.61546	0.045505	0.87787	0.53825	0.95153	FKBP associated protein [Source:HGNC Symbol;Acc:HGNC:14373]"	HGLibA_19279;HGLibA_19280
ENSG00000174840	PDE12	2	1.1601	0.41316	0.049316	0.87787	0.67949	0.95153	phosphodiesterase 12 [Source:HGNC Symbol;Acc:HGNC:25386]	HGLibA_35766;HGLibA_35767
ENSG00000134115	CNTN6	2	1.2912	0.47329	0.031637	0.84371	0.63601	0.95153	contactin 6 [Source:HGNC Symbol;Acc:HGNC:2176]	HGLibA_10417;HGLibA_10416
ENSG00000178741	COX5A	2	1.3113	0.40736	0.029938	0.84371	0.68375	0.95153	cytochrome c oxidase subunit 5A [Source:HGNC Symbol;Acc:HGNC:2267]	HGLibA_10836;HGLibA_10835
ENSG00000167770	OTUB1	3	1.3986	0.45755	0.022683	0.84371	0.64727	0.95153	ubiquitin aldehyde binding 1 [Source:HGNC Symbol;Acc:HGNC:23077]"	HGLibA_34752;HGLibA_34754;HGLibA_34753
ENSG00000175711	B3GNTL1	2	1.3905	0.56011	0.023234	0.84371	0.5754	0.95153	3-N-acetylglucosaminyltransferase like 1 [Source:HGNC Symbol;Acc:HGNC:21727]"	HGLibA_04007;HGLibA_04008
hsa-mir-1302-5	hsa-mir-1302-5	2	1.6487	0.39829	0.011204	0.84371	0.69041	0.95153	-	HGLibA_57707;HGLibA_57709
ENSG00000141698	NT5C3B	2	1.8277	0.37052	0.0074846	0.79902	0.711	0.95153	cytosolic IIIB [Source:HGNC Symbol;Acc:HGNC:28300]"	HGLibA_32817;HGLibA_32818
ENSG00000066923	STAG3	2	1.6911	0.46829	0.010194	0.84371	0.63958	0.95153	stromal antigen 3 [Source:HGNC Symbol;Acc:HGNC:11356]	HGLibA_47327;HGLibA_47328
ENSG00000138376	BARD1	2	1.2638	0.41075	0.034393	0.85011	0.68125	0.95153	BRCA1 associated RING domain 1 [Source:HGNC Symbol;Acc:HGNC:952]	HGLibA_04156;HGLibA_04154
ENSG00000227268	KLLN	2	1.2055	0.43424	0.042887	0.87558	0.66411	0.95153	p53 regulated DNA replication inhibitor [Source:HGNC Symbol;Acc:HGNC:37212]"	HGLibA_25280;HGLibA_25279
ENSG00000158716	DUSP23	3	1.1774	0.79073	0.046836	0.87787	0.4291	0.95153	dual specificity phosphatase 23 [Source:HGNC Symbol;Acc:HGNC:21480]	HGLibA_14165;HGLibA_14167;HGLibA_14166
ENSG00000055044	NOP58	2	1.944	0.32268	0.0060152	0.79902	0.74693	0.9549	NOP58 ribonucleoprotein [Source:HGNC Symbol;Acc:HGNC:29926]	HGLibA_32316;HGLibA_32315
ENSG00000127080	IPPK	2	2.1508	0.26874	0.0039489	0.784	0.78813	0.95947	inositol-pentakisphosphate 2-kinase [Source:HGNC Symbol;Acc:HGNC:14645]	HGLibA_23485;HGLibA_23484
ENSG00000105514	RAB3D	3	1.8634	0.53181	0.0071173	0.79902	0.59486	0.95153	member RAS oncogene family [Source:HGNC Symbol;Acc:HGNC:9779]"	HGLibA_39917;HGLibA_39916;HGLibA_39915
ENSG00000250486	FAM218A	2	1.2141	0.62858	0.041648	0.87558	0.52963	0.95153	family with sequence similarity 218 member A [Source:HGNC Symbol;Acc:HGNC:26466]	HGLibA_16495;HGLibA_16494
ENSG00000139131	YARS2	3	1.5522	0.53834	0.014189	0.84371	0.59034	0.95153	tyrosyl-tRNA synthetase 2 [Source:HGNC Symbol;Acc:HGNC:24249]	HGLibA_54637;HGLibA_54638;HGLibA_54636
ENSG00000169255	B3GALNT1	3	1.5409	0.58858	0.014694	0.84371	0.55614	0.95153	3-N-acetylgalactosaminyltransferase 1 (globoside blood group) [Source:HGNC Symbol;Acc:HGNC:918]"	HGLibA_03947;HGLibA_03949;HGLibA_03948
ENSG00000169258	GPRIN1	2	1.1929	0.45658	0.045	0.87787	0.64797	0.95153	G protein regulated inducer of neurite outgrowth 1 [Source:HGNC Symbol;Acc:HGNC:24835]	HGLibA_20140;HGLibA_20139
ENSG00000135297	MTO1	2	4.0185	0.099533	0.00032143	0.5625	0.92071	0.98352	mitochondrial tRNA translation optimization 1 [Source:HGNC Symbol;Acc:HGNC:19261]	HGLibA_30450;HGLibA_30452
ENSG00000142892	PIGK	2	1.4233	0.54242	0.021352	0.84371	0.58753	0.95153	phosphatidylinositol glycan anchor biosynthesis class K [Source:HGNC Symbol;Acc:HGNC:8965]	HGLibA_36562;HGLibA_36561
ENSG00000137434	C6orf52	2	1.1919	0.48678	0.045183	0.87787	0.62641	0.95153	chromosome 6 open reading frame 52 [Source:HGNC Symbol;Acc:HGNC:20881]	HGLibA_06494;HGLibA_06495
ENSG00000132017	DCAF15	3	1.5046	0.55863	0.016071	0.84371	0.57642	0.95153	DDB1 and CUL4 associated factor 15 [Source:HGNC Symbol;Acc:HGNC:25095]	HGLibA_12431;HGLibA_12430;HGLibA_12429
ENSG00000070371	CLTCL1	2	1.2993	0.59705	0.03104	0.84371	0.55047	0.95153	clathrin heavy chain like 1 [Source:HGNC Symbol;Acc:HGNC:2093]	HGLibA_10152;HGLibA_10153
ENSG00000126787	DLGAP5	3	1.5244	0.44241	0.015337	0.84371	0.65819	0.95153	DLG associated protein 5 [Source:HGNC Symbol;Acc:HGNC:16864]	HGLibA_13350;HGLibA_13351;HGLibA_13352
hsa-mir-563	hsa-mir-563	4	1.1726	0.85622	0.047617	0.87787	0.39188	0.95153	-	HGLibA_62217;HGLibA_62214;HGLibA_62216;HGLibA_62215
ENSG00000179134	SAMD4B	2	3.1458	0.15562	0.00082652	0.6129	0.87633	0.97491	sterile alpha motif domain containing 4B [Source:HGNC Symbol;Acc:HGNC:25492]	HGLibA_42686;HGLibA_42687
ENSG00000119919	NKX2-3	2	1.1902	0.61539	0.045367	0.87787	0.5383	0.95153	NK2 homeobox 3 [Source:HGNC Symbol;Acc:HGNC:7836]	HGLibA_32037;HGLibA_32038
hsa-mir-3180-4	hsa-mir-3180-4	3	1.3002	0.57545	0.030949	0.84371	0.56499	0.95153	-	HGLibA_58812;HGLibA_58814;HGLibA_58813
ENSG00000084072	PPIE	2	1.7382	0.451	0.0090458	0.84371	0.65199	0.95153	peptidylprolyl isomerase E [Source:HGNC Symbol;Acc:HGNC:9258]	HGLibA_37927;HGLibA_37928
ENSG00000181827	RFX7	2	1.1578	0.62753	0.049545	0.87787	0.53031	0.95153	regulatory factor X7 [Source:HGNC Symbol;Acc:HGNC:25777]	HGLibA_40981;HGLibA_40980
ENSG00000164252	AGGF1	1	1.4945	0.30949	0.016852	0.84371	0.75695	0.95596	angiogenic factor with G-patch and FHA domains 1 [Source:HGNC Symbol;Acc:HGNC:24684]	HGLibA_01190
ENSG00000111775	COX6A1	1	2.2023	0.19687	0.0036734	0.784	0.84393	0.96768	cytochrome c oxidase subunit 6A1 [Source:HGNC Symbol;Acc:HGNC:2277]	HGLibA_10843
ENSG00000159147	DONSON	1	1.504	0.30875	0.016117	0.84371	0.75751	0.95596	downstream neighbor of SON [Source:HGNC Symbol;Acc:HGNC:2993]	HGLibA_13796
ENSG00000161203	AP2M1	1	1.8118	0.25524	0.0076224	0.80193	0.79854	0.95997	adaptor related protein complex 2 subunit mu 1 [Source:HGNC Symbol;Acc:HGNC:564]	HGLibA_02313
C22orf43	C22orf43	2	1.3023	0.59053	0.030719	0.84371	0.55484	0.95153	-	HGLibA_06081;HGLibA_06080
ENSG00000139343	SNRPF	3	1.8464	0.47438	0.0073469	0.79902	0.63523	0.95153	small nuclear ribonucleoprotein polypeptide F [Source:HGNC Symbol;Acc:HGNC:11162]	HGLibA_45989;HGLibA_45988;HGLibA_45987
ENSG00000169752	NRG4	2	1.1639	0.63263	0.048627	0.87787	0.52698	0.95153	neuregulin 4 [Source:HGNC Symbol;Acc:HGNC:29862]	HGLibA_32685;HGLibA_32684
ENSG00000032219	ARID4A	3	1.2529	0.76586	0.036275	0.8559	0.44376	0.95153	AT-rich interaction domain 4A [Source:HGNC Symbol;Acc:HGNC:9885]	HGLibA_02899;HGLibA_02900;HGLibA_02901
ENSG00000197766	CFD	2	1.3218	0.53898	0.028882	0.84371	0.5899	0.95153	complement factor D [Source:HGNC Symbol;Acc:HGNC:2771]	HGLibA_09255;HGLibA_09253
ENSG00000261371	PECAM1	2	1.3104	0.58725	0.030076	0.84371	0.55704	0.95153	platelet and endothelial cell adhesion molecule 1 [Source:HGNC Symbol;Acc:HGNC:8823]	HGLibA_36019;HGLibA_36018
H2AFY	H2AFY	1	1.4848	0.31683	0.017311	0.84371	0.75137	0.95562	-	HGLibA_20764
ENSG00000155313	USP25	3	1.449	0.55833	0.019423	0.84371	0.57662	0.95153	ubiquitin specific peptidase 25 [Source:HGNC Symbol;Acc:HGNC:12624]	HGLibA_53311;HGLibA_53312;HGLibA_53310
ENSG00000163666	HESX1	3	1.498	0.47034	0.01676	0.84371	0.63811	0.95153	HESX homeobox 1 [Source:HGNC Symbol;Acc:HGNC:4877]	HGLibA_21198;HGLibA_21199;HGLibA_21197
ENSG00000119203	CPSF3	3	1.2837	0.5049	0.032051	0.84371	0.61363	0.95153	cleavage and polyadenylation specific factor 3 [Source:HGNC Symbol;Acc:HGNC:2326]	HGLibA_11001;HGLibA_11000;HGLibA_10999
ENSG00000091157	WDR7	2	1.5412	0.39906	0.014694	0.84371	0.68985	0.95153	WD repeat domain 7 [Source:HGNC Symbol;Acc:HGNC:13490]	HGLibA_54161;HGLibA_54159
ENSG00000165678	GHITM	2	1.3025	0.60037	0.030719	0.84371	0.54826	0.95153	growth hormone inducible transmembrane protein [Source:HGNC Symbol;Acc:HGNC:17281]	HGLibA_19037;HGLibA_19038
ENSG00000188234	AGAP4	3	1.3719	0.68282	0.024612	0.84371	0.49472	0.95153	ankyrin repeat and PH domain 4 [Source:HGNC Symbol;Acc:HGNC:23459]"	HGLibA_01149;HGLibA_01148;HGLibA_01147
ENSG00000133800	LYVE1	2	1.3296	0.56937	0.028148	0.84371	0.56911	0.95153	lymphatic vessel endothelial hyaluronan receptor 1 [Source:HGNC Symbol;Acc:HGNC:14687]	HGLibA_27778;HGLibA_27779
ENSG00000168427	KLHL30	3	1.2386	0.77318	0.03802	0.86702	0.43942	0.95153	kelch like family member 30 [Source:HGNC Symbol;Acc:HGNC:24770]	HGLibA_25181;HGLibA_25180;HGLibA_25179
ENSG00000100714	MTHFD1	3	1.3832	0.55465	0.023877	0.84371	0.57914	0.95153	cyclohydrolase and formyltetrahydrofolate synthetase 1 [Source:HGNC Symbol;Acc:HGNC:7432]"	HGLibA_30375;HGLibA_30376;HGLibA_30377
FAM19A2	FAM19A2	3	1.3466	0.60519	0.026403	0.84371	0.54505	0.95153	-	HGLibA_16400;HGLibA_16398;HGLibA_16399
hsa-mir-3197	hsa-mir-3197	3	1.3192	0.65094	0.029387	0.84371	0.51509	0.95153	-	HGLibA_58887;HGLibA_58884;HGLibA_58885
ENSG00000158201	ABHD3	3	1.402	0.58844	0.0225	0.84371	0.55623	0.95153	abhydrolase domain containing 3 [Source:HGNC Symbol;Acc:HGNC:18718]	HGLibA_00276;HGLibA_00274;HGLibA_00275
ENSG00000185163	DDX51	3	1.3543	0.68067	0.025806	0.84371	0.49608	0.95153	DEAD-box helicase 51 [Source:HGNC Symbol;Acc:HGNC:20082]	HGLibA_12735;HGLibA_12736;HGLibA_12734
hsa-mir-6128	hsa-mir-6128	4	1.2534	0.72235	0.036275	0.8559	0.47008	0.95153	-	HGLibA_62658;HGLibA_62659;HGLibA_62661;HGLibA_62660
hsa-mir-5695	hsa-mir-5695	4	1.5274	0.52006	0.015107	0.84371	0.60302	0.95153	-	HGLibA_62314;HGLibA_62312;HGLibA_62313;HGLibA_62315
ENSG00000100749	VRK1	2	1.4153	0.50278	0.021765	0.84371	0.61512	0.95153	VRK serine/threonine kinase 1 [Source:HGNC Symbol;Acc:HGNC:12718]	HGLibA_53823;HGLibA_53824
ENSG00000123999	INHA	3	1.1919	0.61851	0.045183	0.87787	0.53624	0.95153	inhibin subunit alpha [Source:HGNC Symbol;Acc:HGNC:6065]	HGLibA_23297;HGLibA_23298;HGLibA_23299
ENSG00000144792	ZNF660	2	1.1624	0.55024	0.048948	0.87787	0.58216	0.95153	zinc finger protein 660 [Source:HGNC Symbol;Acc:HGNC:26720]	HGLibA_56477;HGLibA_56475
ENSG00000074657	ZNF532	2	2.0525	0.33309	0.0047295	0.79902	0.73906	0.95431	zinc finger protein 532 [Source:HGNC Symbol;Acc:HGNC:30940]	HGLibA_56178;HGLibA_56179
ENSG00000221983	UBA52	2	1.4772	0.38595	0.017908	0.84371	0.69954	0.95153	ubiquitin A-52 residue ribosomal protein fusion product 1 [Source:HGNC Symbol;Acc:HGNC:12458]	HGLibA_52526;HGLibA_52525
ENSG00000172602	RND1	1	1.1816	0.3725	0.046193	0.87787	0.70952	0.95153	Rho family GTPase 1 [Source:HGNC Symbol;Acc:HGNC:18314]	HGLibA_41445
ENSG00000114391	RPL24	1	1.4088	0.32714	0.022087	0.84371	0.74356	0.95475	ribosomal protein L24 [Source:HGNC Symbol;Acc:HGNC:10325]	HGLibA_41911
ENSG00000066185	ZMYND12	3	1.2183	0.81073	0.040637	0.87097	0.41752	0.95153	zinc finger MYND-type containing 12 [Source:HGNC Symbol;Acc:HGNC:21192]	HGLibA_55418;HGLibA_55417;HGLibA_55416
ENSG00000006118	TMEM132A	2	1.6483	0.41207	0.011204	0.84371	0.68029	0.95153	transmembrane protein 132A [Source:HGNC Symbol;Acc:HGNC:31092]	HGLibA_49930;HGLibA_49931
ENSG00000197641	SERPINB13	2	1.8451	0.35149	0.0073469	0.79902	0.72522	0.95266	serpin family B member 13 [Source:HGNC Symbol;Acc:HGNC:8944]	HGLibA_43545;HGLibA_43543
ENSG00000197728	RPS26	2	2.4091	0.23871	0.0024796	0.77143	0.81133	0.96179	ribosomal protein S26 [Source:HGNC Symbol;Acc:HGNC:10414]	HGLibA_42119;HGLibA_42120
ENSG00000151883	PARP8	2	1.3808	0.49184	0.023969	0.84371	0.62283	0.95153	poly(ADP-ribose) polymerase family member 8 [Source:HGNC Symbol;Acc:HGNC:26124]	HGLibA_35244;HGLibA_35245
ENSG00000129197	RPAIN	2	2.5437	0.2223	0.0020663	0.76812	0.82408	0.96435	RPA interacting protein [Source:HGNC Symbol;Acc:HGNC:28641]	HGLibA_41810;HGLibA_41812
ENSG00000177370	TIMM22	3	1.1837	0.48611	0.045872	0.87787	0.62689	0.95153	translocase of inner mitochondrial membrane 22 [Source:HGNC Symbol;Acc:HGNC:17317]	HGLibA_49512;HGLibA_49511;HGLibA_49510
ENSG00000146872	TLK2	3	1.4834	0.56688	0.017449	0.84371	0.5708	0.95153	tousled like kinase 2 [Source:HGNC Symbol;Acc:HGNC:11842]	HGLibA_49624;HGLibA_49625;HGLibA_49626
ENSG00000146731	CCT6A	2	1.4007	0.4883	0.022546	0.84371	0.62534	0.95153	chaperonin containing TCP1 subunit 6A [Source:HGNC Symbol;Acc:HGNC:1620]	HGLibA_08208;HGLibA_08207
ENSG00000120370	GORAB	3	1.3588	0.54809	0.025484	0.84371	0.58363	0.95153	RAB6 interacting [Source:HGNC Symbol;Acc:HGNC:25676]"	HGLibA_19675;HGLibA_19676;HGLibA_19674
ENSG00000197172	MAGEA6	1	2.4191	0.1718	0.0024336	0.76812	0.86359	0.9723	MAGE family member A6 [Source:HGNC Symbol;Acc:HGNC:6804]	HGLibA_27917
hsa-mir-548n	hsa-mir-548n	4	1.4345	0.50677	0.020571	0.84371	0.61232	0.95153	-	HGLibA_62046;HGLibA_62044;HGLibA_62043;HGLibA_62045
ENSG00000101883	RHOXF1	3	1.1858	0.75698	0.045688	0.87787	0.44906	0.95153	Rhox homeobox family member 1 [Source:HGNC Symbol;Acc:HGNC:29993]	HGLibA_41215;HGLibA_41214;HGLibA_41213
ENSG00000197532	OR6Y1	2	1.242	0.50459	0.037836	0.86555	0.61384	0.95153	olfactory receptor family 6 subfamily Y member 1 [Source:HGNC Symbol;Acc:HGNC:14823]	HGLibA_34454;HGLibA_34453
ENSG00000110367	DDX6	3	1.1916	0.62769	0.045183	0.87787	0.5302	0.95153	DEAD-box helicase 6 [Source:HGNC Symbol;Acc:HGNC:2747]	HGLibA_12759;HGLibA_12760;HGLibA_12758
ENSG00000110851	PRDM4	3	1.3338	0.70802	0.027688	0.84371	0.47893	0.95153	PR/SET domain 4 [Source:HGNC Symbol;Acc:HGNC:9348]	HGLibA_38389;HGLibA_38388;HGLibA_38390
ENSG00000106278	PTPRZ1	1	1.293	0.34999	0.031454	0.84371	0.72635	0.95266	receptor type Z1 [Source:HGNC Symbol;Acc:HGNC:9685]"	HGLibA_39543
ENSG00000109184	DCUN1D4	1	1.4822	0.31199	0.017541	0.84371	0.75505	0.95596	defective in cullin neddylation 1 domain containing 4 [Source:HGNC Symbol;Acc:HGNC:28998]	HGLibA_12574
ENSG00000133103	COG6	3	1.7271	0.50987	0.0093673	0.84371	0.61014	0.95153	component of oligomeric golgi complex 6 [Source:HGNC Symbol;Acc:HGNC:18621]	HGLibA_10484;HGLibA_10486;HGLibA_10485
ENSG00000118526	TCF21	2	1.3826	0.54805	0.023877	0.84371	0.58366	0.95153	transcription factor 21 [Source:HGNC Symbol;Acc:HGNC:11632]	HGLibA_48773;HGLibA_48772
ENSG00000076201	PTPN23	3	1.2753	0.57815	0.032877	0.84371	0.56317	0.95153	non-receptor type 23 [Source:HGNC Symbol;Acc:HGNC:14406]"	HGLibA_39463;HGLibA_39462;HGLibA_39464
ENSG00000116874	WARS2	3	1.9449	0.46023	0.0059234	0.79902	0.64535	0.95153	mitochondrial [Source:HGNC Symbol;Acc:HGNC:12730]"	HGLibA_53947;HGLibA_53948;HGLibA_53946
ENSG00000256806	C17orf100	1	1.4736	0.31274	0.018	0.84371	0.75448	0.95596	chromosome 17 open reading frame 100 [Source:HGNC Symbol;Acc:HGNC:34494]	HGLibA_05517
hsa-mir-8087	hsa-mir-8087	2	1.4562	0.38664	0.01901	0.84371	0.69902	0.95153	-	HGLibA_64189;HGLibA_64192
ENSG00000163754	GYG1	3	1.2674	0.71781	0.033658	0.84936	0.47287	0.95153	glycogenin 1 [Source:HGNC Symbol;Acc:HGNC:4699]	HGLibA_20687;HGLibA_20688;HGLibA_20689
ENSG00000152689	RASGRP3	3	1.3039	0.72481	0.030535	0.84371	0.46857	0.95153	RAS guanyl releasing protein 3 [Source:HGNC Symbol;Acc:HGNC:14545]	HGLibA_40349;HGLibA_40350;HGLibA_40351
ENSG00000100462	PRMT5	1	1.6015	0.28989	0.012811	0.84371	0.7719	0.95759	protein arginine methyltransferase 5 [Source:HGNC Symbol;Acc:HGNC:10894]	HGLibA_38628
ENSG00000198937	CCDC167	2	1.2082	0.52268	0.042336	0.87558	0.6012	0.95153	coiled-coil domain containing 167 [Source:HGNC Symbol;Acc:HGNC:21239]	HGLibA_07675;HGLibA_07676
ENSG00000215784	FAM72D	3	1.1749	0.67335	0.047295	0.87787	0.50073	0.95153	family with sequence similarity 72 member D [Source:HGNC Symbol;Acc:HGNC:33593]	HGLibA_16714;HGLibA_16713;HGLibA_16712
ENSG00000099817	POLR2E	3	1.1926	0.73306	0.045091	0.87787	0.46352	0.95153	RNA polymerase II subunit E [Source:HGNC Symbol;Acc:HGNC:9192]	HGLibA_37575;HGLibA_37576;HGLibA_37577
ENSG00000112742	TTK	3	1.2112	0.60476	0.042015	0.87558	0.54534	0.95153	TTK protein kinase [Source:HGNC Symbol;Acc:HGNC:12401]	HGLibA_52181;HGLibA_52179;HGLibA_52180
ENSG00000114021	NIT2	3	1.3409	0.61654	0.027	0.84371	0.53754	0.95153	nitrilase family member 2 [Source:HGNC Symbol;Acc:HGNC:29878]	HGLibA_31987;HGLibA_31986;HGLibA_31985
ENSG00000136715	SAP130	3	1.3299	0.61986	0.028102	0.84371	0.53535	0.95153	Sin3A associated protein 130 [Source:HGNC Symbol;Acc:HGNC:29813]	HGLibA_42712;HGLibA_42714;HGLibA_42713
hsa-mir-3669	hsa-mir-3669	1	4.5822	0.038137	0.00013775	0.5	0.96958	0.99326	-	HGLibA_59225
ENSG00000285077	ARHGAP11B	1	1.706	0.27682	0.0098723	0.84371	0.78191	0.95872	Rho GTPase activating protein 11B [Source:HGNC Symbol;Acc:HGNC:15782]	HGLibA_02706
ENSG00000100567	PSMA3	1	2.8776	0.1284	0.0011479	0.67568	0.89783	0.9795	proteasome subunit alpha 3 [Source:HGNC Symbol;Acc:HGNC:9532]	HGLibA_39077
LOC100130880	LOC100130880	2	1.201	0.58448	0.043484	0.87616	0.55889	0.95153	-	HGLibA_26799;HGLibA_26798
ENSG00000169375	SIN3A	2	1.4764	0.50622	0.017908	0.84371	0.6127	0.95153	SIN3 transcription regulator family member A [Source:HGNC Symbol;Acc:HGNC:19353]	HGLibA_44182;HGLibA_44183
ENSG00000225968	ELFN1	2	1.4059	0.53923	0.02227	0.84371	0.58973	0.95153	extracellular leucine rich repeat and fibronectin type III domain containing 1 [Source:HGNC Symbol;Acc:HGNC:33154]	HGLibA_14906;HGLibA_14905
ENSG00000105072	C19orf44	3	1.3618	0.53719	0.025439	0.84371	0.59114	0.95153	chromosome 19 open reading frame 44 [Source:HGNC Symbol;Acc:HGNC:26141]	HGLibA_05664;HGLibA_05662;HGLibA_05663
ENSG00000113810	SMC4	2	3.5652	0.13306	0.00041326	0.5625	0.89415	0.97853	structural maintenance of chromosomes 4 [Source:HGNC Symbol;Acc:HGNC:14013]	HGLibA_45652;HGLibA_45653
ENSG00000005175	RPAP3	3	1.3287	0.67172	0.028194	0.84371	0.50176	0.95153	RNA polymerase II associated protein 3 [Source:HGNC Symbol;Acc:HGNC:26151]	HGLibA_41820;HGLibA_41819;HGLibA_41821
ENSG00000175203	DCTN2	3	1.1902	0.51179	0.045367	0.87787	0.6088	0.95153	dynactin subunit 2 [Source:HGNC Symbol;Acc:HGNC:2712]	HGLibA_12548;HGLibA_12547;HGLibA_12546
ENSG00000039650	PNKP	3	1.2729	0.59221	0.033015	0.8439	0.55371	0.95153	polynucleotide kinase 3'-phosphatase [Source:HGNC Symbol;Acc:HGNC:9154]	HGLibA_37352;HGLibA_37351;HGLibA_37350
ENSG00000184293	CLECL1	1	1.3819	0.33059	0.023923	0.84371	0.74096	0.95441	C-type lectin like 1 [Source:HGNC Symbol;Acc:HGNC:24462]	HGLibA_10013
ENSG00000145675	PIK3R1	2	1.1659	0.52239	0.048397	0.87787	0.6014	0.95153	phosphoinositide-3-kinase regulatory subunit 1 [Source:HGNC Symbol;Acc:HGNC:8979]	HGLibA_36648;HGLibA_36649
ENSG00000133858	ZFC3H1	3	1.2592	0.57609	0.034989	0.8514	0.56456	0.95153	zinc finger C3H1-type containing [Source:HGNC Symbol;Acc:HGNC:28328]	HGLibA_55169;HGLibA_55167;HGLibA_55168
ENSG00000130810	PPAN	3	1.6403	0.53841	0.011571	0.84371	0.59029	0.95153	peter pan homolog [Source:HGNC Symbol;Acc:HGNC:9227]	HGLibA_37810;HGLibA_37809;HGLibA_37811
ENSG00000161652	IZUMO2	2	1.2477	0.46859	0.037056	0.85956	0.63937	0.95153	IZUMO family member 2 [Source:HGNC Symbol;Acc:HGNC:28518]	HGLibA_23896;HGLibA_23895
ENSG00000172742	OR4D9	2	1.842	0.37423	0.0073928	0.79902	0.70824	0.95153	olfactory receptor family 4 subfamily D member 9 [Source:HGNC Symbol;Acc:HGNC:15178]	HGLibA_33984;HGLibA_33982
ENSG00000125772	GPCPD1	2	1.4388	0.41067	0.020158	0.84371	0.68132	0.95153	glycerophosphocholine phosphodiesterase 1 [Source:HGNC Symbol;Acc:HGNC:26957]	HGLibA_19779;HGLibA_19780
ENSG00000164902	PHAX	2	1.6713	0.36047	0.010837	0.84371	0.7185	0.95266	phosphorylated adaptor for RNA export [Source:HGNC Symbol;Acc:HGNC:10241]	HGLibA_36309;HGLibA_36308
SPDYE2L	SPDYE2L	3	1.9628	0.44114	0.0056479	0.79902	0.65911	0.95153	-	HGLibA_46589;HGLibA_46587;HGLibA_46588
ENSG00000068394	GPKOW	2	1.6428	0.40293	0.011479	0.84371	0.687	0.95153	G-patch domain and KOW motifs [Source:HGNC Symbol;Acc:HGNC:30677]	HGLibA_19813;HGLibA_19814
FDX1L	FDX1L	1	1.5246	0.30477	0.015337	0.84371	0.76054	0.95605	-	HGLibA_17261
ENSG00000160193	WDR4	2	2.1693	0.31381	0.003903	0.784	0.75366	0.95596	WD repeat domain 4 [Source:HGNC Symbol;Acc:HGNC:12756]	HGLibA_54086;HGLibA_54084
KIAA0226L	KIAA0226L	1	1.5994	0.29038	0.012857	0.84371	0.77152	0.95755	-	HGLibA_24608
ENSG00000176571	CNBD1	1	1.8415	0.24861	0.0073928	0.79902	0.80366	0.96046	cyclic nucleotide binding domain containing 1 [Source:HGNC Symbol;Acc:HGNC:26663]	HGLibA_10242
ENSG00000139644	TMBIM6	2	1.282	0.52208	0.032097	0.84371	0.60162	0.95153	transmembrane BAX inhibitor motif containing 6 [Source:HGNC Symbol;Acc:HGNC:11723]	HGLibA_49746;HGLibA_49744
ENSG00000185052	SLC24A3	2	1.8211	0.37978	0.0075305	0.8	0.70411	0.95153	solute carrier family 24 member 3 [Source:HGNC Symbol;Acc:HGNC:10977]	HGLibA_44621;HGLibA_44622
ENSG00000130427	EPO	3	1.4483	0.69014	0.019423	0.84371	0.49011	0.95153	erythropoietin [Source:HGNC Symbol;Acc:HGNC:3415]	HGLibA_15351;HGLibA_15350;HGLibA_15349
ENSG00000137513	NARS2	2	1.6271	0.46932	0.012214	0.84371	0.63884	0.95153	mitochondrial [Source:HGNC Symbol;Acc:HGNC:26274]"	HGLibA_31158;HGLibA_31157
ENSG00000121749	TBC1D15	3	1.223	0.59344	0.039811	0.87097	0.55289	0.95153	TBC1 domain family member 15 [Source:HGNC Symbol;Acc:HGNC:25694]	HGLibA_48481;HGLibA_48483;HGLibA_48482
ENSG00000067182	TNFRSF1A	2	1.245	0.6204	0.037377	0.85956	0.535	0.95153	TNF receptor superfamily member 1A [Source:HGNC Symbol;Acc:HGNC:11916]	HGLibA_50773;HGLibA_50772
ENSG00000198598	TMED2	1	1.4107	0.32568	0.022087	0.84371	0.74467	0.95475	transmembrane p24 trafficking protein 2 [Source:HGNC Symbol;Acc:HGNC:16996]	HGLibA_49804
ENSG00000231500	RPS18	2	3.3515	0.16159	0.0005051	0.57895	0.87163	0.9745	ribosomal protein S18 [Source:HGNC Symbol;Acc:HGNC:10401]	HGLibA_42092;HGLibA_42093
ENSG00000131188	PRR7	1	1.3641	0.33452	0.025255	0.84371	0.73798	0.95387	synaptic [Source:HGNC Symbol;Acc:HGNC:28130]"	HGLibA_38852
ENSG00000180739	S1PR5	3	1.2328	0.68672	0.038571	0.86735	0.49226	0.95153	sphingosine-1-phosphate receptor 5 [Source:HGNC Symbol;Acc:HGNC:14299]	HGLibA_42605;HGLibA_42604;HGLibA_42606
ENSG00000196427	NBPF4	2	1.8954	0.38628	0.0064744	0.79902	0.69929	0.95153	NBPF member 4 [Source:HGNC Symbol;Acc:HGNC:26550]	HGLibA_31236;HGLibA_31237
ENSG00000125356	NDUFA1	2	1.2677	0.5395	0.033658	0.84936	0.58954	0.95153	NADH:ubiquinone oxidoreductase subunit A1 [Source:HGNC Symbol;Acc:HGNC:7683]	HGLibA_31438;HGLibA_31436
ENSG00000171858	RPS21	2	1.5557	0.49637	0.014143	0.84371	0.61963	0.95153	ribosomal protein S21 [Source:HGNC Symbol;Acc:HGNC:10409]	HGLibA_42108;HGLibA_42107
ENSG00000196459	TRAPPC2	2	2.2783	0.29602	0.0031224	0.784	0.76722	0.95707	trafficking protein particle complex 2 [Source:HGNC Symbol;Acc:HGNC:23068]	HGLibA_51312;HGLibA_51311
ENSG00000105726	ATP13A1	3	1.6757	0.49222	0.010561	0.84371	0.62257	0.95153	ATPase 13A1 [Source:HGNC Symbol;Acc:HGNC:24215]	HGLibA_03566;HGLibA_03565;HGLibA_03567
ENSG00000225921	NOL7	3	2.1123	0.4331	0.0041326	0.784	0.66494	0.95153	nucleolar protein 7 [Source:HGNC Symbol;Acc:HGNC:21040]	HGLibA_32274;HGLibA_32275;HGLibA_32273
ENSG00000075461	CACNG4	2	1.161	0.65312	0.049086	0.87787	0.51368	0.95153	calcium voltage-gated channel auxiliary subunit gamma 4 [Source:HGNC Symbol;Acc:HGNC:1408]	HGLibA_06938;HGLibA_06939
ENSG00000182195	LDOC1	3	1.4587	0.59912	0.018872	0.84371	0.54909	0.95153	regulator of NFKB signaling [Source:HGNC Symbol;Acc:HGNC:6548]"	HGLibA_26257;HGLibA_26256;HGLibA_26255
ENSG00000126549	STATH	3	1.3193	0.75494	0.029387	0.84371	0.45028	0.95153	statherin [Source:HGNC Symbol;Acc:HGNC:11369]	HGLibA_47402;HGLibA_47403;HGLibA_47401
ENSG00000119681	LTBP2	2	1.1567	0.52673	0.049821	0.87787	0.59838	0.95153	latent transforming growth factor beta binding protein 2 [Source:HGNC Symbol;Acc:HGNC:6715]	HGLibA_27593;HGLibA_27592
ENSG00000167447	SMG8	2	1.9752	0.37573	0.005602	0.79902	0.70712	0.95153	nonsense mediated mRNA decay factor [Source:HGNC Symbol;Acc:HGNC:25551]"	HGLibA_45711;HGLibA_45709
ENSG00000167113	COQ4	1	1.3549	0.33567	0.02576	0.84371	0.73712	0.95358	coenzyme Q4 [Source:HGNC Symbol;Acc:HGNC:19693]	HGLibA_10755
HIST4H4	HIST4H4	3	1.1899	0.7581	0.045413	0.87787	0.44839	0.95153	-	HGLibA_21563;HGLibA_21564;HGLibA_21565
ENSG00000205022	PABPN1L	3	1.3789	0.58993	0.024153	0.84371	0.55524	0.95153	cytoplasmic [Source:HGNC Symbol;Acc:HGNC:37237]"	HGLibA_34946;HGLibA_34945;HGLibA_34944
ENSG00000087157	PGS1	1	1.3309	0.34283	0.027964	0.84371	0.73172	0.95266	phosphatidylglycerophosphate synthase 1 [Source:HGNC Symbol;Acc:HGNC:30029]	HGLibA_36294
ENSG00000089289	IGBP1	2	1.3301	0.4908	0.028102	0.84371	0.62357	0.95153	immunoglobulin binding protein 1 [Source:HGNC Symbol;Acc:HGNC:5461]	HGLibA_22781;HGLibA_22783
ENSG00000168906	MAT2A	3	1.6732	0.5529	0.010699	0.84371	0.58033	0.95153	methionine adenosyltransferase 2A [Source:HGNC Symbol;Acc:HGNC:6904]	HGLibA_28419;HGLibA_28417;HGLibA_28418
ENSG00000185608	MRPL40	3	1.3334	0.55605	0.027734	0.84371	0.57818	0.95153	mitochondrial ribosomal protein L40 [Source:HGNC Symbol;Acc:HGNC:14491]	HGLibA_29978;HGLibA_29979;HGLibA_29977
ENSG00000112592	TBP	2	1.1638	0.55478	0.048673	0.87787	0.57905	0.95153	TATA-box binding protein [Source:HGNC Symbol;Acc:HGNC:11588]	HGLibA_48624;HGLibA_48623
ENSG00000181381	DDX60L	3	1.56	0.55699	0.014097	0.84371	0.57753	0.95153	DExD/H-box 60 like [Source:HGNC Symbol;Acc:HGNC:26429]	HGLibA_12764;HGLibA_12766;HGLibA_12765
ENSG00000086548	CEACAM6	1	2.4895	0.16347	0.0022959	0.76812	0.87015	0.9741	carcinoembryonic antigen related cell adhesion molecule 6 [Source:HGNC Symbol;Acc:HGNC:1818]	HGLibA_08948
ENSG00000165525	NEMF	2	1.3541	0.48346	0.025806	0.84371	0.62877	0.95153	nuclear export mediator factor [Source:HGNC Symbol;Acc:HGNC:10663]	HGLibA_31689;HGLibA_31688
ENSG00000160563	MED27	2	1.2278	0.48173	0.039122	0.8685	0.63	0.95153	mediator complex subunit 27 [Source:HGNC Symbol;Acc:HGNC:2377]	HGLibA_28794;HGLibA_28793
ENSG00000120436	GPR31	2	2.0344	0.35641	0.0049591	0.79902	0.72153	0.95266	G protein-coupled receptor 31 [Source:HGNC Symbol;Acc:HGNC:4486]	HGLibA_20016;HGLibA_20017
ENSG00000153064	BANK1	3	1.276	0.72075	0.032877	0.84371	0.47106	0.95153	B cell scaffold protein with ankyrin repeats 1 [Source:HGNC Symbol;Acc:HGNC:18233]	HGLibA_04145;HGLibA_04146;HGLibA_04147
ENSG00000163202	LCE3D	1	1.7826	0.25935	0.0083571	0.83871	0.79536	0.95961	late cornified envelope 3D [Source:HGNC Symbol;Acc:HGNC:16615]	HGLibA_26138
hsa-mir-1912	hsa-mir-1912	2	2.0912	0.30461	0.0042704	0.784	0.76067	0.95605	-	HGLibA_58053;HGLibA_58054
FAM86C1	FAM86C1	3	1.5397	0.63113	0.014694	0.84371	0.52795	0.95153	-	HGLibA_16779;HGLibA_16778;HGLibA_16780
hsa-mir-4293	hsa-mir-4293	4	1.3712	0.85498	0.024612	0.84371	0.39256	0.95153	-	HGLibA_59883;HGLibA_59882;HGLibA_59884;HGLibA_59885
ENSG00000198554	WDHD1	1	1.5701	0.29559	0.013821	0.84371	0.76754	0.95707	WD repeat and HMG-box DNA binding protein 1 [Source:HGNC Symbol;Acc:HGNC:23170]	HGLibA_54015
ENSG00000001167	NFYA	1	1.9657	0.22996	0.0056479	0.79902	0.81812	0.96298	nuclear transcription factor Y subunit alpha [Source:HGNC Symbol;Acc:HGNC:7804]	HGLibA_31853
ENSG00000100883	SRP54	2	1.2074	0.51675	0.042474	0.87558	0.60533	0.95153	signal recognition particle 54 [Source:HGNC Symbol;Acc:HGNC:11301]	HGLibA_47006;HGLibA_47007
ENSG00000176714	CCDC121	2	1.1985	0.59843	0.044219	0.87787	0.54955	0.95153	coiled-coil domain containing 121 [Source:HGNC Symbol;Acc:HGNC:25833]	HGLibA_07568;HGLibA_07569
ENSG00000136826	KLF4	3	1.4484	0.57661	0.019423	0.84371	0.5642	0.95153	Kruppel like factor 4 [Source:HGNC Symbol;Acc:HGNC:6348]	HGLibA_25073;HGLibA_25072;HGLibA_25071
ENSG00000221947	XKR9	2	1.3909	0.4548	0.023189	0.84371	0.64925	0.95153	XK related 9 [Source:HGNC Symbol;Acc:HGNC:20937]	HGLibA_54536;HGLibA_54534
ENSG00000106701	FSD1L	3	1.3207	0.75757	0.029204	0.84371	0.44871	0.95153	fibronectin type III and SPRY domain containing 1 like [Source:HGNC Symbol;Acc:HGNC:13753]	HGLibA_18080;HGLibA_18081;HGLibA_18079
ENSG00000018869	ZNF582	3	1.3044	0.64726	0.030535	0.84371	0.51747	0.95153	zinc finger protein 582 [Source:HGNC Symbol;Acc:HGNC:26421]	HGLibA_56310;HGLibA_56312;HGLibA_56311
ENSG00000145632	PLK2	2	1.5472	0.47341	0.014464	0.84371	0.63592	0.95153	polo like kinase 2 [Source:HGNC Symbol;Acc:HGNC:19699]	HGLibA_37192;HGLibA_37191
ENSG00000076604	TRAF4	2	1.1659	0.41199	0.048397	0.87787	0.68034	0.95153	TNF receptor associated factor 4 [Source:HGNC Symbol;Acc:HGNC:12034]	HGLibA_51257;HGLibA_51259
ENSG00000115541	HSPE1	3	1.9981	0.43884	0.0054183	0.79902	0.66078	0.95153	heat shock protein family E (Hsp10) member 1 [Source:HGNC Symbol;Acc:HGNC:5269]	HGLibA_22335;HGLibA_22333;HGLibA_22334

ENSG00000249115	HAUS5	3	1.4086	0.53362	0.022178	0.84371	0.5936	0.95153	HAUS augmin like complex subunit 5 [Source:HGNC Symbol;Acc:HGNC:29130]	HGLibA_20887;HGLibA_20886;HGLibA_20885
ENSG00000177613	CSTF2T	2	1.3277	0.41642	0.028285	0.84371	0.6771	0.95153	cleavage stimulation factor subunit 2 tau variant [Source:HGNC Symbol;Acc:HGNC:17086]	HGLibA_11489;HGLibA_11490
ENSG00000187416	LHFPL3	2	1.1604	0.42127	0.04927	0.87787	0.67356	0.95153	LHFPL tetraspan subfamily member 3 [Source:HGNC Symbol;Acc:HGNC:6589]	HGLibA_26436;HGLibA_26435
ENSG00000134333	LDHA	2	1.5177	0.3793	0.015566	0.84371	0.70446	0.95153	lactate dehydrogenase A [Source:HGNC Symbol;Acc:HGNC:6535]	HGLibA_26221;HGLibA_26220
ENSG00000176845	METRNL	2	1.1592	0.60271	0.049408	0.87787	0.5467	0.95153	glial cell differentiation regulator [Source:HGNC Symbol;Acc:HGNC:27584]"	HGLibA_28940;HGLibA_28941
ENSG00000204822	MRPL53	2	2.0952	0.28598	0.0042244	0.784	0.77489	0.95782	mitochondrial ribosomal protein L53 [Source:HGNC Symbol;Acc:HGNC:16684]	HGLibA_30017;HGLibA_30016
ENSG00000007202	KIAA0100	2	1.3471	0.40333	0.026357	0.84371	0.68671	0.95153	KIAA0100 [Source:HGNC Symbol;Acc:HGNC:28960]	HGLibA_24587;HGLibA_24588
ENSG00000151151	IPMK	3	1.4785	0.49115	0.017908	0.84371	0.62332	0.95153	inositol polyphosphate multikinase [Source:HGNC Symbol;Acc:HGNC:20739]	HGLibA_23458;HGLibA_23456;HGLibA_23457
ENSG00000179115	FARSA	2	1.9911	0.31698	0.0055101	0.79902	0.75126	0.95562	phenylalanyl-tRNA synthetase subunit alpha [Source:HGNC Symbol;Acc:HGNC:3592]	HGLibA_16883;HGLibA_16884
ENSG00000105372	RPS19	1	1.3219	0.34413	0.028882	0.84371	0.73075	0.95266	ribosomal protein S19 [Source:HGNC Symbol;Acc:HGNC:10402]	HGLibA_42095
ENSG00000008324	SS18L2	2	2.1208	0.29179	0.0040867	0.784	0.77045	0.95707	SS18 like 2 [Source:HGNC Symbol;Acc:HGNC:15593]	HGLibA_47107;HGLibA_47108
ENSG00000135486	HNRNPA1	3	2.0855	0.41806	0.0044081	0.784	0.67591	0.95153	heterogeneous nuclear ribonucleoprotein A1 [Source:HGNC Symbol;Acc:HGNC:5031]	HGLibA_21791;HGLibA_21793;HGLibA_21792
ENSG00000178096	BOLA1	2	1.7362	0.39884	0.0090917	0.84371	0.69001	0.95153	bolA family member 1 [Source:HGNC Symbol;Acc:HGNC:24263]	HGLibA_04680;HGLibA_04679
hsa-mir-9-3	hsa-mir-9-3	2	1.2636	0.56376	0.034393	0.85011	0.57292	0.95153	-	HGLibA_64270;HGLibA_64269
ENSG00000133055	MYBPH	3	1.7194	0.51267	0.0095968	0.84371	0.60819	0.95153	myosin binding protein H [Source:HGNC Symbol;Acc:HGNC:7552]	HGLibA_30657;HGLibA_30659;HGLibA_30658
ENSG00000079482	OPHN1	3	1.5636	0.56425	0.014005	0.84371	0.57259	0.95153	oligophrenin 1 [Source:HGNC Symbol;Acc:HGNC:8148]	HGLibA_33417;HGLibA_33415;HGLibA_33416
ENSG00000198380	GFPT1	2	1.5887	0.43041	0.013178	0.84371	0.6669	0.95153	glutamine--fructose-6-phosphate transaminase 1 [Source:HGNC Symbol;Acc:HGNC:4241]	HGLibA_18959;HGLibA_18958
ENSG00000166211	SPIC	3	1.1626	0.65233	0.048948	0.87787	0.51419	0.95153	Spi-C transcription factor [Source:HGNC Symbol;Acc:HGNC:29549]	HGLibA_46657;HGLibA_46659;HGLibA_46658
ENSG00000165060	FXN	2	1.4615	0.50579	0.01878	0.84371	0.61301	0.95153	frataxin [Source:HGNC Symbol;Acc:HGNC:3951]	HGLibA_18216;HGLibA_18215
ENSG00000139726	DENR	2	1.9056	0.4045	0.0062908	0.79902	0.68584	0.95153	density regulated re-initiation and release factor [Source:HGNC Symbol;Acc:HGNC:2769]	HGLibA_12970;HGLibA_12971
ENSG00000171487	NLRP5	3	1.2612	0.77501	0.03453	0.85011	0.43833	0.95153	NLR family pyrin domain containing 5 [Source:HGNC Symbol;Acc:HGNC:21269]	HGLibA_32123;HGLibA_32125;HGLibA_32124
ENSG00000169436	COL22A1	2	1.7234	0.45943	0.0094591	0.84371	0.64592	0.95153	collagen type XXII alpha 1 chain [Source:HGNC Symbol;Acc:HGNC:22989]	HGLibA_10541;HGLibA_10542
ENSG00000187479	C11orf96	3	1.2266	0.76115	0.03926	0.87067	0.44657	0.95153	chromosome 11 open reading frame 96 [Source:HGNC Symbol;Acc:HGNC:38675]	HGLibA_05221;HGLibA_05220;HGLibA_05219
ENSG00000183751	TBL3	1	1.3155	0.34414	0.029617	0.84371	0.73074	0.95266	transducin beta like 3 [Source:HGNC Symbol;Acc:HGNC:11587]	HGLibA_48620
ENSG00000089157	RPLP0	3	1.358	0.70733	0.02553	0.84371	0.47936	0.95153	ribosomal protein lateral stalk subunit P0 [Source:HGNC Symbol;Acc:HGNC:10371]	HGLibA_42009;HGLibA_42008;HGLibA_42007
ENSG00000259571	BLID	3	1.1771	0.63551	0.046928	0.87787	0.52509	0.95153	cell death inducer [Source:HGNC Symbol;Acc:HGNC:33495]"	HGLibA_04541;HGLibA_04542;HGLibA_04543
ENSG00000124380	SNRNP27	3	1.3513	0.52337	0.02599	0.84371	0.60072	0.95153	small nuclear ribonucleoprotein U4/U6.U5 subunit 27 [Source:HGNC Symbol;Acc:HGNC:30240]	HGLibA_45947;HGLibA_45948;HGLibA_45946
ENSG00000164985	PSIP1	2	1.8846	0.33532	0.0066122	0.79902	0.73738	0.95358	PC4 and SFRS1 interacting protein 1 [Source:HGNC Symbol;Acc:HGNC:9527]	HGLibA_39060;HGLibA_39062
ENSG00000183111	ARHGEF37	2	1.6783	0.44074	0.010561	0.84371	0.6594	0.95153	Rho guanine nucleotide exchange factor 37 [Source:HGNC Symbol;Acc:HGNC:34430]	HGLibA_02854;HGLibA_02855
ENSG00000166402	TUB	2	1.2044	0.43266	0.043071	0.87558	0.66526	0.95153	tubby bipartite transcription factor [Source:HGNC Symbol;Acc:HGNC:12406]	HGLibA_52240;HGLibA_52239
ENSG00000182795	C1orf116	2	1.4479	0.44276	0.019469	0.84371	0.65794	0.95153	chromosome 1 open reading frame 116 [Source:HGNC Symbol;Acc:HGNC:28667]	HGLibA_05755;HGLibA_05756
ENSG00000126267	COX6B1	2	1.2479	0.52388	0.03701	0.85956	0.60036	0.95153	cytochrome c oxidase subunit 6B1 [Source:HGNC Symbol;Acc:HGNC:2280]	HGLibA_10849;HGLibA_10847
ENSG00000155097	ATP6V1C1	3	1.3817	0.59885	0.023923	0.84371	0.54927	0.95153	ATPase H+ transporting V1 subunit C1 [Source:HGNC Symbol;Acc:HGNC:856]	HGLibA_03739;HGLibA_03738;HGLibA_03740
ENSG00000145912	NHP2	3	1.2317	0.6045	0.038617	0.86735	0.54551	0.95153	NHP2 ribonucleoprotein [Source:HGNC Symbol;Acc:HGNC:14377]	HGLibA_31907;HGLibA_31908;HGLibA_31909
ENSG00000185813	PCYT2	2	1.4137	0.54527	0.021903	0.84371	0.58557	0.95153	ethanolamine [Source:HGNC Symbol;Acc:HGNC:8756]"	HGLibA_35707;HGLibA_35706
ENSG00000256188	TAS2R30	2	1.7618	0.41718	0.0086785	0.84371	0.67655	0.95153	taste 2 receptor member 30 [Source:HGNC Symbol;Acc:HGNC:19112]	HGLibA_48386;HGLibA_48388
hsa-mir-548f-4	hsa-mir-548f-4	3	1.3073	0.5918	0.030214	0.84371	0.55399	0.95153	-	HGLibA_61985;HGLibA_61982;HGLibA_61984
ZCCHC6	ZCCHC6	2	1.5024	0.45921	0.016301	0.84371	0.64608	0.95153	-	HGLibA_55043;HGLibA_55041
ENSG00000156304	SCAF4	2	1.4995	0.43866	0.01653	0.84371	0.66091	0.95153	SR-related CTD associated factor 4 [Source:HGNC Symbol;Acc:HGNC:19304]	HGLibA_42826;HGLibA_42827
ENSG00000130595	TNNT3	2	1.1598	0.52467	0.049316	0.87787	0.59981	0.95153	fast skeletal type [Source:HGNC Symbol;Acc:HGNC:11950]"	HGLibA_50890;HGLibA_50889
ENSG00000160949	TONSL	2	1.4241	0.55848	0.021352	0.84371	0.57651	0.95153	DNA repair protein [Source:HGNC Symbol;Acc:HGNC:7801]"	HGLibA_50985;HGLibA_50986
ENSG00000175305	CCNE2	3	1.2173	0.61085	0.040821	0.87097	0.5413	0.95153	cyclin E2 [Source:HGNC Symbol;Acc:HGNC:1590]	HGLibA_08086;HGLibA_08088;HGLibA_08087
ENSG00000143257	NR1I3	3	1.2771	0.73449	0.032785	0.84371	0.46265	0.95153	nuclear receptor subfamily 1 group I member 3 [Source:HGNC Symbol;Acc:HGNC:7969]	HGLibA_32591;HGLibA_32592;HGLibA_32593
ENSG00000101773	RBBP8	1	3.7445	0.067962	0.00041326	0.5625	0.94582	0.98802	endonuclease [Source:HGNC Symbol;Acc:HGNC:9891]"	HGLibA_40439
ENSG00000059588	TARBP1	3	1.3724	0.64658	0.024566	0.84371	0.5179	0.95153	TAR (HIV-1) RNA binding protein 1 [Source:HGNC Symbol;Acc:HGNC:11568]	HGLibA_48329;HGLibA_48331;HGLibA_48330
ENSG00000182117	NOP10	1	1.774	0.25993	0.0084489	0.84371	0.79491	0.95986	NOP10 ribonucleoprotein [Source:HGNC Symbol;Acc:HGNC:14378]	HGLibA_32301
ENSG00000109805	NCAPG	1	3.3218	0.094409	0.00059693	0.59091	0.92478	0.98395	non-SMC condensin I complex subunit G [Source:HGNC Symbol;Acc:HGNC:24304]	HGLibA_31270
ENSG00000114302	PRKAR2A	3	1.1868	0.66276	0.045642	0.87787	0.50748	0.95153	protein kinase cAMP-dependent type II regulatory subunit alpha [Source:HGNC Symbol;Acc:HGNC:9391]	HGLibA_38526;HGLibA_38528;HGLibA_38527
ENSG00000071575	TRIB2	3	1.1562	0.60033	0.049867	0.87787	0.54828	0.95153	tribbles pseudokinase 2 [Source:HGNC Symbol;Acc:HGNC:30809]	HGLibA_51392;HGLibA_51394;HGLibA_51393
hsa-mir-4479	hsa-mir-4479	2	1.6883	0.40091	0.010332	0.84371	0.68848	0.95153	-	HGLibA_60329;HGLibA_60331
ENSG00000089505	CMTM1	3	1.5398	0.64041	0.014694	0.84371	0.52191	0.95153	CKLF like MARVEL transmembrane domain containing 1 [Source:HGNC Symbol;Acc:HGNC:19172]	HGLibA_10210;HGLibA_10208;HGLibA_10209
ENSG00000185271	KLHL33	2	1.5381	0.36351	0.014694	0.84371	0.71622	0.95243	kelch like family member 33 [Source:HGNC Symbol;Acc:HGNC:31952]	HGLibA_25188;HGLibA_25189
ENSG00000153930	ANKFN1	2	1.3218	0.59399	0.028882	0.84371	0.55252	0.95153	ankyrin repeat and fibronectin type III domain containing 1 [Source:HGNC Symbol;Acc:HGNC:26766]	HGLibA_01955;HGLibA_01954
ENSG00000173113	TRMT112	2	2.2555	0.25736	0.0033061	0.784	0.7969	0.95974	tRNA methyltransferase subunit 11-2 [Source:HGNC Symbol;Acc:HGNC:26940]	HGLibA_51673;HGLibA_51671
HIST2H3A	HIST2H3A	3	1.696	0.44899	0.010056	0.84371	0.65344	0.95153	-	HGLibA_21539;HGLibA_21540;HGLibA_21541
ENSG00000204882	GPR20	3	1.1788	0.77058	0.046515	0.87787	0.44096	0.95153	G protein-coupled receptor 20 [Source:HGNC Symbol;Acc:HGNC:4475]	HGLibA_19996;HGLibA_19995;HGLibA_19997
ENSG00000198203	SULT1C2	3	1.3578	0.56655	0.02553	0.84371	0.57102	0.95153	sulfotransferase family 1C member 2 [Source:HGNC Symbol;Acc:HGNC:11456]	HGLibA_47747;HGLibA_47745;HGLibA_47746
ENSG00000124562	SNRPC	1	2.4779	0.1642	0.0023418	0.76812	0.86958	0.9741	small nuclear ribonucleoprotein polypeptide C [Source:HGNC Symbol;Acc:HGNC:11157]	HGLibA_45973
ENSG00000092208	GEMIN2	2	2.042	0.31285	0.0048673	0.79902	0.7544	0.95596	gem nuclear organelle associated protein 2 [Source:HGNC Symbol;Acc:HGNC:10884]	HGLibA_18910;HGLibA_18912
ENSG00000149016	TUT1	2	1.6201	0.48187	0.012398	0.84371	0.6299	0.95153	U6 snRNA-specific [Source:HGNC Symbol;Acc:HGNC:26184]"	HGLibA_52353;HGLibA_52354
ENSG00000163631	ALB	3	1.6068	0.50276	0.012673	0.84371	0.61513	0.95153	albumin [Source:HGNC Symbol;Acc:HGNC:399]	HGLibA_01530;HGLibA_01529;HGLibA_01528
ENSG00000107872	FBXL15	2	1.3039	0.59354	0.030535	0.84371	0.55282	0.95153	F-box and leucine rich repeat protein 15 [Source:HGNC Symbol;Acc:HGNC:28155]	HGLibA_16981;HGLibA_16982
ENSG00000156052	GNAQ	3	1.2311	0.5252	0.038755	0.86735	0.59945	0.95153	G protein subunit alpha q [Source:HGNC Symbol;Acc:HGNC:4390]	HGLibA_19466;HGLibA_19467;HGLibA_19465
ENSG00000105640	RPL18A	3	2.1278	0.38718	0.0040408	0.784	0.69862	0.95153	ribosomal protein L18a [Source:HGNC Symbol;Acc:HGNC:10311]	HGLibA_41889;HGLibA_41888;HGLibA_41890
hsa-mir-342	hsa-mir-342	4	1.5198	0.62175	0.01552	0.84371	0.53411	0.95153	-	HGLibA_59025;HGLibA_59023;HGLibA_59024;HGLibA_59022
ENSG00000244067	GSTA2	1	1.5562	0.29639	0.014143	0.84371	0.76693	0.95707	glutathione S-transferase alpha 2 [Source:HGNC Symbol;Acc:HGNC:4627]	HGLibA_20453
ENSG00000129521	EGLN3	3	1.4704	0.5085	0.018138	0.84371	0.6111	0.95153	egl-9 family hypoxia inducible factor 3 [Source:HGNC Symbol;Acc:HGNC:14661]	HGLibA_14646;HGLibA_14645;HGLibA_14644
ENSG00000136710	CCDC115	1	1.9403	0.2361	0.0060152	0.79902	0.81335	0.96246	coiled-coil domain containing 115 [Source:HGNC Symbol;Acc:HGNC:28178]	HGLibA_07554
ENSG00000177186	OR2M7	2	1.1588	0.49626	0.049408	0.87787	0.61971	0.95153	olfactory receptor family 2 subfamily M member 7 [Source:HGNC Symbol;Acc:HGNC:19594]	HGLibA_33846;HGLibA_33845
ENSG00000116237	ICMT	2	1.1671	0.52382	0.048306	0.87787	0.6004	0.95153	isoprenylcysteine carboxyl methyltransferase [Source:HGNC Symbol;Acc:HGNC:5350]	HGLibA_22511;HGLibA_22513
FAM208B	FAM208B	3	1.257	0.59881	0.035724	0.8559	0.5493	0.95153	-	HGLibA_16436;HGLibA_16435;HGLibA_16434
ENSG00000110536	PTPMT1	3	1.7655	0.50746	0.0086326	0.84371	0.61183	0.95153	mitochondrial 1 [Source:HGNC Symbol;Acc:HGNC:26965]"	HGLibA_39427;HGLibA_39428;HGLibA_39426
ENSG00000131778	CHD1L	3	1.2702	0.6726	0.033245	0.84579	0.5012	0.95153	chromodomain helicase DNA binding protein 1 like [Source:HGNC Symbol;Acc:HGNC:1916]	HGLibA_09385;HGLibA_09386;HGLibA_09387
ENSG00000136271	DDX56	2	2.4608	0.2409	0.0023418	0.76812	0.80963	0.96114	DEAD-box helicase 56 [Source:HGNC Symbol;Acc:HGNC:18193]	HGLibA_12750;HGLibA_12749
ENSG00000186583	SPATC1	2	1.4118	0.51439	0.021995	0.84371	0.60698	0.95153	spermatogenesis and centriole associated 1 [Source:HGNC Symbol;Acc:HGNC:30510]	HGLibA_46536;HGLibA_46538
ENSG00000101951	PAGE4	2	1.3425	0.42247	0.026816	0.84371	0.67268	0.95153	PAGE family member 4 [Source:HGNC Symbol;Acc:HGNC:4108]	HGLibA_35012;HGLibA_35013
ENSG00000077721	UBE2A	3	1.2709	0.66456	0.033153	0.84543	0.50633	0.95153	ubiquitin conjugating enzyme E2 A [Source:HGNC Symbol;Acc:HGNC:12472]	HGLibA_52574;HGLibA_52573;HGLibA_52572
ENSG00000005022	SLC25A5	3	1.4286	0.6099	0.020939	0.84371	0.54193	0.95153	solute carrier family 25 member 5 [Source:HGNC Symbol;Acc:HGNC:10991]	HGLibA_44763;HGLibA_44762;HGLibA_44761
ENSG00000089060	SLC8B1	3	1.293	0.67926	0.031454	0.84371	0.49697	0.95153	solute carrier family 8 member B1 [Source:HGNC Symbol;Acc:HGNC:26175]	HGLibA_45396;HGLibA_45397;HGLibA_45398
T	T	2	1.5846	0.40318	0.013316	0.84371	0.68682	0.95153	-	HGLibA_48121;HGLibA_48120
ENSG00000163870	TPRA1	3	1.2164	0.57372	0.041188	0.87512	0.56616	0.95153	transmembrane protein adipocyte associated 1 [Source:HGNC Symbol;Acc:HGNC:30413]	HGLibA_51178;HGLibA_51177;HGLibA_51176
ENSG00000165449	SLC16A9	2	1.5805	0.49537	0.013408	0.84371	0.62034	0.95153	solute carrier family 16 member 9 [Source:HGNC Symbol;Acc:HGNC:23520]	HGLibA_44457;HGLibA_44455
hsa-mir-148a	hsa-mir-148a	2	1.2323	0.5085	0.038617	0.86735	0.61111	0.95153	-	HGLibA_57881;HGLibA_57879
ENSG00000128591	FLNC	2	1.5027	0.49445	0.016255	0.84371	0.62099	0.95153	filamin C [Source:HGNC Symbol;Acc:HGNC:3756]	HGLibA_17644;HGLibA_17645

Table S2 Negative candidate genes in 1st screen

Gene	Gene Name	sgRNA	group|beta	group|z	group|p-value	group|fdr	group|wald-p-value	group|wald-fdr	Description	sgRNA ID
ENSG00000124201	ZNFX1	1	-3.0392	-2.2738	0.026607	0.83016	0.022976	0.92867	zinc finger NFX1-type containing 1 [Source:HGNC Symbol;Acc:HGNC:29271]	HGLibA_56919
ENSG00000189058	APOD	2	-3.8963	-4.6077	0.007904	0.81716	4.07E-06	0.0022714	apolipoprotein D [Source:HGNC Symbol;Acc:HGNC:612]	HGLibA_02515;HGLibA_02516
ENSG00000147687	TATDN1	2	-3.1791	-3.9224	0.022471	0.83016	8.77E-05	0.023845	TatD DNase domain containing 1 [Source:HGNC Symbol;Acc:HGNC:24220]	HGLibA_48440;HGLibA_48441
ENSG00000058600	POLR3E	2	-4.6772	-3.4793	0.00073526	0.78846	0.0005028	0.084165	RNA polymerase III subunit E [Source:HGNC Symbol;Acc:HGNC:30347]	HGLibA_37620;HGLibA_37621
ENSG00000213585	VDAC1	2	-2.259	-2.3636	0.046827	0.85738	0.0181	0.82056	voltage dependent anion channel 1 [Source:HGNC Symbol;Acc:HGNC:12669]	HGLibA_53599;HGLibA_53598
ENSG00000213859	KCTD11	3	-4.0117	-3.1023	0.0062497	0.81716	0.00192	0.21887	potassium channel tetramerization domain containing 11 [Source:HGNC Symbol;Acc:HGNC:21302]	HGLibA_24418;HGLibA_24416;HGLibA_24417
ENSG00000164654	MIOS	2	-3.0729	-1.5372	0.025872	0.83016	0.12425	0.95326	meiosis regulator for oocyte development [Source:HGNC Symbol;Acc:HGNC:21905]	HGLibA_29284;HGLibA_29286
ENSG00000145782	ATG12	2	-3.3489	-4.2339	0.01829	0.82766	2.30E-05	0.0081943	autophagy related 12 [Source:HGNC Symbol;Acc:HGNC:588]	HGLibA_03465;HGLibA_03463
ENSG00000160886	LY6K	3	-4.2333	-6.0794	0.0036763	0.81716	1.21E-09	8.45E-06	lymphocyte antigen 6 family member K [Source:HGNC Symbol;Acc:HGNC:24225]	HGLibA_27678;HGLibA_27676;HGLibA_27677
ENSG00000091140	DLD	1	-3.8509	-2.8201	0.0084095	0.81716	0.0048014	0.38413	dihydrolipoamide dehydrogenase [Source:HGNC Symbol;Acc:HGNC:2898]	HGLibA_13316
ENSG00000187398	LUZP2	1	-3.5534	-2.622	0.014246	0.82103	0.0087425	0.54759	leucine zipper protein 2 [Source:HGNC Symbol;Acc:HGNC:23206]	HGLibA_27640
ENSG00000147689	FAM83A	3	-2.5462	-1.3998	0.038096	0.83236	0.16158	0.95326	family with sequence similarity 83 member A [Source:HGNC Symbol;Acc:HGNC:28210]	HGLibA_16740;HGLibA_16739;HGLibA_16741
TARS	TARS	2	-2.5214	-2.575	0.038647	0.83236	0.010023	0.58686	-	HGLibA_48345;HGLibA_48344
hsa-mir-20a	hsa-mir-20a	1	-4.4003	-3.1928	0.0022517	0.81667	0.001409	0.17412	-	HGLibA_58202
ENSG00000082482	KCNK2	1	-2.9606	-2.2378	0.028078	0.83016	0.025235	0.95326	potassium two pore domain channel subfamily K member 2 [Source:HGNC Symbol;Acc:HGNC:6277]	HGLibA_24319
ENSG00000176490	DIRAS1	3	-2.814	-1.6132	0.032397	0.83236	0.10671	0.95326	DIRAS family GTPase 1 [Source:HGNC Symbol;Acc:HGNC:19127]	HGLibA_13255;HGLibA_13256;HGLibA_13257
ENSG00000138326	RPS24	2	-2.8979	-2.0482	0.029824	0.83236	0.040537	0.95326	ribosomal protein S24 [Source:HGNC Symbol;Acc:HGNC:10411]	HGLibA_42114;HGLibA_42113
ENSG00000124541	RRP36	2	-4.1067	-5.0544	0.0051468	0.81716	4.32E-07	0.00042039	ribosomal RNA processing 36 [Source:HGNC Symbol;Acc:HGNC:21374]	HGLibA_42279;HGLibA_42277
ENSG00000178358	OR2D3	3	-3.9062	-3.1833	0.0077662	0.81716	0.001456	0.17701	olfactory receptor family 2 subfamily D member 3 [Source:HGNC Symbol;Acc:HGNC:15146]	HGLibA_33785;HGLibA_33786;HGLibA_33784
ENSG00000197497	ZNF665	2	-3.6861	-4.0309	0.011029	0.82103	5.56E-05	0.016771	zinc finger protein 665 [Source:HGNC Symbol;Acc:HGNC:25885]	HGLibA_56488;HGLibA_56489
ENSG00000100393	EP300	3	-3.4494	-4.2601	0.016268	0.82103	2.04E-05	0.0075376	E1A binding protein p300 [Source:HGNC Symbol;Acc:HGNC:3373]	HGLibA_15229;HGLibA_15231;HGLibA_15230
ENSG00000122958	VPS26A	2	-2.9018	-3.0808	0.029824	0.83236	0.0020647	0.2304	retromer complex component A [Source:HGNC Symbol;Acc:HGNC:12711]"	HGLibA_53751;HGLibA_53753
ENSG00000147509	RGS20	1	-3.1637	-2.3676	0.022793	0.83016	0.017904	0.81508	regulator of G protein signaling 20 [Source:HGNC Symbol;Acc:HGNC:14600]	HGLibA_41081
ENSG00000197971	MBP	3	-3.1628	-1.3632	0.023115	0.83016	0.17282	0.95326	myelin basic protein [Source:HGNC Symbol;Acc:HGNC:6925]	HGLibA_28530;HGLibA_28528;HGLibA_28529
ENSG00000172650	AGAP5	3	-3.006	-3.5038	0.027251	0.83016	0.0004587	0.077982	ankyrin repeat and PH domain 5 [Source:HGNC Symbol;Acc:HGNC:23467]"	HGLibA_01151;HGLibA_01150;HGLibA_01152
ENSG00000162129	CLPB	3	-3.0607	-1.2808	0.026148	0.83016	0.20025	0.95326	mitochondrial AAA ATPase chaperonin [Source:HGNC Symbol;Acc:HGNC:30664]"	HGLibA_10098;HGLibA_10099;HGLibA_10097
ENSG00000174720	LARP7	3	-2.3639	-0.73684	0.043151	0.84121	0.46122	0.95326	La ribonucleoprotein domain family member 7 [Source:HGNC Symbol;Acc:HGNC:24912]	HGLibA_26044;HGLibA_26042;HGLibA_26043
ENSG00000174606	ANGEL2	2	-3.3206	-0.96608	0.018887	0.82766	0.33401	0.95326	angel homolog 2 [Source:HGNC Symbol;Acc:HGNC:30534]	HGLibA_01897;HGLibA_01898
ENSG00000164758	MED30	1	-3.0469	-2.2927	0.026332	0.83016	0.021866	0.89948	mediator complex subunit 30 [Source:HGNC Symbol;Acc:HGNC:23032]	HGLibA_28803
ENSG00000109184	DCUN1D4	1	-2.866	-2.1781	0.030513	0.83236	0.029399	0.95326	defective in cullin neddylation 1 domain containing 4 [Source:HGNC Symbol;Acc:HGNC:28998]	HGLibA_12574
ENSG00000064218	DMRT3	3	-2.9231	-1.4154	0.028905	0.83201	0.15694	0.95326	doublesex and mab-3 related transcription factor 3 [Source:HGNC Symbol;Acc:HGNC:13909]	HGLibA_13423;HGLibA_13422;HGLibA_13424
ENSG00000088986	DYNLL1	1	-4.4314	-3.2141	0.0021598	0.79661	0.0013083	0.16457	dynein light chain LC8-type 1 [Source:HGNC Symbol;Acc:HGNC:15476]	HGLibA_14252
MTERFD2	MTERFD2	3	-4.068	-5.4831	0.0056523	0.81716	4.18E-08	7.00E-05	-	HGLibA_30343;HGLibA_30344;HGLibA_30342
hsa-mir-4419a	hsa-mir-4419a	1	-4.6423	-3.3592	0.00082717	0.78846	0.00078167	0.11812	-	HGLibA_60059
ENSG00000114388	NPRL2	2	-2.6847	-2.6333	0.035063	0.83236	0.008455	0.5418	GATOR1 complex subunit [Source:HGNC Symbol;Acc:HGNC:24969]"	HGLibA_32516;HGLibA_32518
ENSG00000128829	EIF2AK4	2	-2.5602	-2.5833	0.037728	0.83236	0.0097856	0.58501	eukaryotic translation initiation factor 2 alpha kinase 4 [Source:HGNC Symbol;Acc:HGNC:19687]	HGLibA_14733;HGLibA_14731
ENSG00000067334	DNTTIP2	2	-2.2332	-2.5164	0.047332	0.85762	0.011856	0.64019	deoxynucleotidyltransferase terminal interacting protein 2 [Source:HGNC Symbol;Acc:HGNC:24013]	HGLibA_13722;HGLibA_13723
ENSG00000089009	RPL6	2	-3.4521	-4.3155	0.016268	0.82103	1.59E-05	0.0060784	ribosomal protein L6 [Source:HGNC Symbol;Acc:HGNC:10362]	HGLibA_41990;HGLibA_41991
hsa-mir-4771-2	hsa-mir-4771-2	2	-2.4037	-0.58	0.042232	0.84121	0.56191	0.95326	-	HGLibA_61183;HGLibA_61185
ENSG00000198796	ALPK2	3	-2.8516	-3.332	0.031157	0.83236	0.00086231	0.12679	alpha kinase 2 [Source:HGNC Symbol;Acc:HGNC:20565]	HGLibA_01703;HGLibA_01704;HGLibA_01702
ENSG00000146670	CDCA5	3	-3.4742	-2.3229	0.01613	0.82103	0.020185	0.86275	cell division cycle associated 5 [Source:HGNC Symbol;Acc:HGNC:14626]	HGLibA_08610;HGLibA_08611;HGLibA_08612
hsa-mir-548ak	hsa-mir-548ak	1	-3.7133	-2.728	0.010477	0.82103	0.0063711	0.45014	-	HGLibA_61894
hsa-mir-548au	hsa-mir-548au	2	-3.6763	-2.586	0.011397	0.82103	0.0097111	0.58216	-	HGLibA_61928;HGLibA_61929
ENSG00000271567	PPIAL4E	2	-2.905	-1.5006	0.029732	0.83236	0.13345	0.95326	peptidylprolyl isomerase A like 4E [Source:HGNC Symbol;Acc:HGNC:33997]	HGLibA_37909;HGLibA_37910
ENSG00000137440	FGFBP1	2	-4.4799	-2.9432	0.0017462	0.78846	0.0032488	0.30473	fibroblast growth factor binding protein 1 [Source:HGNC Symbol;Acc:HGNC:19695]	HGLibA_17427;HGLibA_17426
ENSG00000110719	TCIRG1	3	-2.7756	-2.1517	0.033133	0.83236	0.031418	0.95326	ATPase H+ transporting V0 subunit a3 [Source:HGNC Symbol;Acc:HGNC:11647]"	HGLibA_48812;HGLibA_48811;HGLibA_48813
ENSG00000198835	GJC2	1	-3.2346	-2.4134	0.020679	0.83016	0.015806	0.76097	gap junction protein gamma 2 [Source:HGNC Symbol;Acc:HGNC:17494]	HGLibA_19182
ENSG00000182504	CEP97	2	-2.5255	-3.3808	0.038509	0.83236	0.00072285	0.11236	centrosomal protein 97 [Source:HGNC Symbol;Acc:HGNC:26244]	HGLibA_09181;HGLibA_09183
ENSG00000117091	CD48	2	-3.4481	-3.3164	0.016451	0.82103	0.00091177	0.1314	CD48 molecule [Source:HGNC Symbol;Acc:HGNC:1683]	HGLibA_08400;HGLibA_08399
ENSG00000234284	ZNF879	3	-3.8985	-3.4815	0.007904	0.81716	0.00049854	0.084098	zinc finger protein 879 [Source:HGNC Symbol;Acc:HGNC:37273]	HGLibA_56889;HGLibA_56890;HGLibA_56891
hsa-mir-6813	hsa-mir-6813	3	-2.3913	-1.4066	0.042691	0.84121	0.15955	0.95326	-	HGLibA_63428;HGLibA_63431;HGLibA_63430
ENSG00000079215	SLC1A3	1	-4.1497	-3.0218	0.0044116	0.81716	0.0025126	0.26036	solute carrier family 1 member 3 [Source:HGNC Symbol;Acc:HGNC:10941]	HGLibA_44514
ENSG00000111669	TPI1	1	-3.9387	-2.8791	0.0073066	0.81716	0.0039886	0.34306	triosephosphate isomerase 1 [Source:HGNC Symbol;Acc:HGNC:12009]	HGLibA_51135
H2AFZ	H2AFZ	2	-2.3333	-2.8208	0.04407	0.85018	0.0047899	0.38413	-	HGLibA_20769;HGLibA_20770
ENSG00000103429	BFAR	1	-3.8135	-2.795	0.008961	0.81716	0.0051901	0.39909	bifunctional apoptosis regulator [Source:HGNC Symbol;Acc:HGNC:17613]	HGLibA_04453
ENSG00000108107	RPL28	2	-2.9944	-3.0663	0.027618	0.83016	0.0021675	0.23763	ribosomal protein L28 [Source:HGNC Symbol;Acc:HGNC:10330]	HGLibA_41924;HGLibA_41925
ENSG00000164902	PHAX	2	-2.9294	-3.7979	0.028767	0.83201	0.0001459	0.035674	phosphorylated adaptor for RNA export [Source:HGNC Symbol;Acc:HGNC:10241]	HGLibA_36309;HGLibA_36308
ENSG00000052795	FNIP2	2	-3.5506	-2.0021	0.014567	0.82103	0.04527	0.95326	folliculin interacting protein 2 [Source:HGNC Symbol;Acc:HGNC:29280]	HGLibA_17767;HGLibA_17769
ENSG00000148606	POLR3A	2	-3.1735	-2.5808	0.022563	0.83016	0.0098567	0.58617	RNA polymerase III subunit A [Source:HGNC Symbol;Acc:HGNC:30074]	HGLibA_37609;HGLibA_37610
ENSG00000081154	PCNP	2	-4.1287	-5.1853	0.0047332	0.81716	2.16E-07	0.00027612	PEST proteolytic signal containing nuclear protein [Source:HGNC Symbol;Acc:HGNC:30023]	HGLibA_35636;HGLibA_35637
ENSG00000164011	ZNF691	2	-3.1981	-4.0942	0.022058	0.83016	4.24E-05	0.013762	zinc finger protein 691 [Source:HGNC Symbol;Acc:HGNC:28028]	HGLibA_56552;HGLibA_56551
ENSG00000197713	RPE	3	-3.3786	-3.2596	0.017462	0.82609	0.0011157	0.15366	ribulose-5-phosphate-3-epimerase [Source:HGNC Symbol;Acc:HGNC:10293]	HGLibA_41824;HGLibA_41822;HGLibA_41823
C3orf17	C3orf17	1	-3.0874	-2.3186	0.025321	0.83016	0.020418	0.86275	-	HGLibA_06212
ENSG00000096080	MRPS18A	3	-2.7449	-1.0777	0.033914	0.83236	0.28115	0.95326	mitochondrial ribosomal protein S18A [Source:HGNC Symbol;Acc:HGNC:14515]	HGLibA_30049;HGLibA_30050;HGLibA_30051
ENSG00000134061	CD180	2	-2.9368	-0.33336	0.028675	0.83201	0.73886	0.95685	CD180 molecule [Source:HGNC Symbol;Acc:HGNC:6726]	HGLibA_08257;HGLibA_08259
ENSG00000153147	SMARCA5	3	-2.9811	-5.0008	0.027756	0.83016	5.71E-07	0.00048353	member 5 [Source:HGNC Symbol;Acc:HGNC:11101]"	HGLibA_45610;HGLibA_45612;HGLibA_45611
ENSG00000174780	SRP72	2	-2.6622	-2.8572	0.035338	0.83236	0.0042735	0.35763	signal recognition particle 72 [Source:HGNC Symbol;Acc:HGNC:11303]	HGLibA_47011;HGLibA_47012
ENSG00000159593	NAE1	1	-4.0037	-2.9229	0.0062497	0.81716	0.0034677	0.31369	NEDD8 activating enzyme E1 subunit 1 [Source:HGNC Symbol;Acc:HGNC:621]	HGLibA_31058
ENSG00000124608	AARS2	2	-4.5763	-5.0578	0.0011948	0.78846	4.24E-07	0.00042039	mitochondrial [Source:HGNC Symbol;Acc:HGNC:21022]"	HGLibA_00072;HGLibA_00070
ENSG00000111641	NOP2	2	-3.382	-1.3534	0.017416	0.82609	0.17592	0.95326	NOP2 nucleolar protein [Source:HGNC Symbol;Acc:HGNC:7867]	HGLibA_32310;HGLibA_32309
ENSG00000103569	AQP9	1	-3.4761	-2.571	0.015854	0.82103	0.01014	0.58686	aquaporin 9 [Source:HGNC Symbol;Acc:HGNC:643]	HGLibA_02611
ENSG00000197321	SVIL	3	-3.1738	-1.2339	0.022563	0.83016	0.21726	0.95326	supervillin [Source:HGNC Symbol;Acc:HGNC:11480]	HGLibA_47886;HGLibA_47887;HGLibA_47885
ENSG00000211456	SACM1L	1	-3.9554	-2.8903	0.0070769	0.81716	0.0038489	0.33435	SAC1 like phosphatidylinositide phosphatase [Source:HGNC Symbol;Acc:HGNC:17059]	HGLibA_42626
hsa-mir-1299	hsa-mir-1299	2	-2.9228	-2.2843	0.028905	0.83201	0.022355	0.91098	-	HGLibA_57676;HGLibA_57674
ENSG00000169981	ZNF35	2	-2.7326	-2.8148	0.034006	0.83236	0.0048804	0.38797	zinc finger protein 35 [Source:HGNC Symbol;Acc:HGNC:13099]	HGLibA_55854;HGLibA_55855
ENSG00000137876	RSL24D1	2	-3.7398	-2.8897	0.010064	0.81716	0.0038566	0.33435	ribosomal L24 domain containing 1 [Source:HGNC Symbol;Acc:HGNC:18479]	HGLibA_42321;HGLibA_42320
ENSG00000187522	HSPA14	2	-2.8427	-1.0181	0.031249	0.83236	0.30865	0.95326	heat shock protein family A (Hsp70) member 14 [Source:HGNC Symbol;Acc:HGNC:29526]	HGLibA_22268;HGLibA_22269
ENSG00000261308	FIGNL2	3	-2.4377	-1.7691	0.041634	0.84121	0.076881	0.95326	fidgetin like 2 [Source:HGNC Symbol;Acc:HGNC:13287]	HGLibA_17524;HGLibA_17526;HGLibA_17525
EFTUD1	EFTUD1	2	-3.4221	-3.919	0.016773	0.82103	8.89E-05	0.02389	-	HGLibA_14614;HGLibA_14615
ENSG00000198700	IPO9	2	-3.3306	-3.6243	0.018795	0.82766	0.00028976	0.056	importin 9 [Source:HGNC Symbol;Acc:HGNC:19425]	HGLibA_23479;HGLibA_23477
ENSG00000147586	MRPS28	2	-3.4018	-2.3736	0.017095	0.82119	0.017617	0.80879	mitochondrial ribosomal protein S28 [Source:HGNC Symbol;Acc:HGNC:14513]	HGLibA_30084;HGLibA_30083
ENSG00000163728	TTC14	2	-3.4355	-3.7099	0.016589	0.82103	0.00020737	0.046047	tetratricopeptide repeat domain 14 [Source:HGNC Symbol;Acc:HGNC:24697]	HGLibA_52055;HGLibA_52053
ENSG00000066813	ACSM2B	3	-2.9953	-4.8186	0.027526	0.83016	1.45E-06	0.0010303	acyl-CoA synthetase medium chain family member 2B [Source:HGNC Symbol;Acc:HGNC:30931]	HGLibA_00574;HGLibA_00576;HGLibA_00575
ENSG00000116455	WDR77	1	-2.866	-2.1781	0.030513	0.83236	0.029399	0.95326	WD repeat domain 77 [Source:HGNC Symbol;Acc:HGNC:29652]	HGLibA_54181
ENSG00000158104	HPD	2	-2.3825	-2.482	0.042921	0.84121	0.013063	0.67521	4-hydroxyphenylpyruvate dioxygenase [Source:HGNC Symbol;Acc:HGNC:5147]	HGLibA_22025;HGLibA_22024
ENSG00000173726	TOMM20	1	-3.5534	-2.622	0.014246	0.82103	0.0087425	0.54759	translocase of outer mitochondrial membrane 20 [Source:HGNC Symbol;Acc:HGNC:20947]	HGLibA_50954
CCDC84	CCDC84	2	-3.0792	-3.1241	0.02578	0.83016	0.0017837	0.20757	-	HGLibA_07888;HGLibA_07890
ENSG00000087128	TMPRSS11E	2	-2.2608	-2.2433	0.046781	0.85738	0.024876	0.95326	transmembrane serine protease 11E [Source:HGNC Symbol;Acc:HGNC:24465]	HGLibA_50621;HGLibA_50620
ENSG00000169446	MMGT1	2	-3.1799	-4.0982	0.022425	0.83016	4.16E-05	0.013731	membrane magnesium transporter 1 [Source:HGNC Symbol;Acc:HGNC:28100]	HGLibA_29475;HGLibA_29473
ENSG00000184277	TM2D3	3	-2.4706	-0.7144	0.040256	0.83524	0.47498	0.95326	TM2 domain containing 3 [Source:HGNC Symbol;Acc:HGNC:24128]	HGLibA_49687;HGLibA_49688;HGLibA_49689
ENSG00000204007	GLT6D1	3	-3.228	-1.3311	0.021231	0.83016	0.18315	0.95326	glycosyltransferase 6 domain containing 1 [Source:HGNC Symbol;Acc:HGNC:23671]	HGLibA_19335;HGLibA_19333;HGLibA_19334
ENSG00000108518	PFN1	1	-2.4386	-1.9148	0.040945	0.83741	0.055519	0.95326	profilin 1 [Source:HGNC Symbol;Acc:HGNC:8881]	HGLibA_36174
hsa-mir-4671	hsa-mir-4671	3	-3.8227	-3.6796	0.008915	0.81716	0.00023357	0.04983	-	HGLibA_60802;HGLibA_60801;HGLibA_60800
ENSG00000141552	ANAPC11	3	-3.1577	-4.6164	0.023299	0.83016	3.91E-06	0.0022714	anaphase promoting complex subunit 11 [Source:HGNC Symbol;Acc:HGNC:14452]	HGLibA_01869;HGLibA_01867;HGLibA_01868
ENSG00000011590	ZBTB32	2	-2.5195	-2.576	0.038693	0.83236	0.0099954	0.58686	zinc finger and BTB domain containing 32 [Source:HGNC Symbol;Acc:HGNC:16763]	HGLibA_54858;HGLibA_54860
ENSG00000183431	SF3A3	2	-3.1961	-3.6623	0.022058	0.83016	0.00024995	0.051802	splicing factor 3a subunit 3 [Source:HGNC Symbol;Acc:HGNC:10767]	HGLibA_43698;HGLibA_43697
ENSG00000186105	LRRC70	1	-3.5779	-2.6381	0.013602	0.82103	0.0083361	0.53828	leucine rich repeat containing 70 [Source:HGNC Symbol;Acc:HGNC:35155]	HGLibA_27417
ENSG00000138095	LRPPRC	2	-3.3936	-4.0891	0.017233	0.82599	4.33E-05	0.013858	leucine rich pentatricopeptide repeat containing [Source:HGNC Symbol;Acc:HGNC:15714]	HGLibA_27239;HGLibA_27240
ENSG00000131143	COX4I1	2	-2.5906	-2.746	0.036947	0.83236	0.006032	0.43754	cytochrome c oxidase subunit 4I1 [Source:HGNC Symbol;Acc:HGNC:2265]	HGLibA_10831;HGLibA_10829

ENSG00000136522	MRPL47	3	-3.1308	-3.8478	0.023896	0.83016	0.00011917	0.030508	mitochondrial ribosomal protein L47 [Source:HGNC Symbol;Acc:HGNC:16652]	HGLibA_29999;HGLibA_29998;HGLibA_30000
ENSG00000256892	MTRNR2L7	2	-2.5896	-2.9818	0.036947	0.83236	0.0028654	0.27961	MT-RNR2 like 7 [Source:HGNC Symbol;Acc:HGNC:37164]	HGLibA_30488;HGLibA_30489
ENSG00000166974	MAPRE2	3	-2.4445	-1.6768	0.040807	0.83741	0.093591	0.95326	microtubule associated protein RP/EB family member 2 [Source:HGNC Symbol;Acc:HGNC:6891]	HGLibA_28317;HGLibA_28316;HGLibA_28315
ENSG00000055950	MRPL43	2	-3.4665	-4.4131	0.016176	0.82103	1.02E-05	0.0041059	mitochondrial ribosomal protein L43 [Source:HGNC Symbol;Acc:HGNC:14517]	HGLibA_29988;HGLibA_29986
ENSG00000092295	TGM1	3	-3.9683	-5.328	0.0070309	0.81716	9.93E-08	0.00014402	transglutaminase 1 [Source:HGNC Symbol;Acc:HGNC:11777]	HGLibA_49254;HGLibA_49252;HGLibA_49253
ENSG00000120694	HSPH1	2	-2.4821	-0.40517	0.040072	0.83524	0.68535	0.95405	heat shock protein family H (Hsp110) member 1 [Source:HGNC Symbol;Acc:HGNC:16969]	HGLibA_22344;HGLibA_22343
ENSG00000084070	SMAP2	2	-3.3306	-3.6243	0.018795	0.82766	0.00028976	0.056	small ArfGAP2 [Source:HGNC Symbol;Acc:HGNC:25082]	HGLibA_45598;HGLibA_45600
ENSG00000121892	PDS5A	2	-2.9282	-3.5464	0.028859	0.83201	0.0003905	0.069653	PDS5 cohesin associated factor A [Source:HGNC Symbol;Acc:HGNC:29088]	HGLibA_35942;HGLibA_35944
ENSG00000142657	PGD	3	-2.3461	-3.6871	0.043794	0.84738	0.00022682	0.048869	phosphogluconate dehydrogenase [Source:HGNC Symbol;Acc:HGNC:8891]	HGLibA_36232;HGLibA_36230;HGLibA_36231
ENSG00000115310	RTN4	3	-2.7285	-3.945	0.034006	0.83236	7.98E-05	0.022045	reticulon 4 [Source:HGNC Symbol;Acc:HGNC:14085]	HGLibA_42408;HGLibA_42406;HGLibA_42407
ENSG00000087365	SF3B2	1	-4.9086	-3.5436	0.00027572	0.66667	0.00039478	0.069843	splicing factor 3b subunit 2 [Source:HGNC Symbol;Acc:HGNC:10769]	HGLibA_43705
ENSG00000131351	HAUS8	3	-3.8752	-3.5858	0.0081338	0.81716	0.00033609	0.061459	HAUS augmin like complex subunit 8 [Source:HGNC Symbol;Acc:HGNC:30532]	HGLibA_20896;HGLibA_20895;HGLibA_20894
ENSG00000133606	MKRN1	3	-3.0305	-0.53638	0.026837	0.83016	0.59169	0.95326	makorin ring finger protein 1 [Source:HGNC Symbol;Acc:HGNC:7112]	HGLibA_29348;HGLibA_29347;HGLibA_29349
ENSG00000172534	HCFC1	2	-2.7758	-3.623	0.033087	0.83236	0.00029118	0.056	host cell factor C1 [Source:HGNC Symbol;Acc:HGNC:4839]	HGLibA_20957;HGLibA_20959
ENSG00000145331	TRMT10A	3	-2.7919	-0.71973	0.032857	0.83236	0.47169	0.95326	tRNA methyltransferase 10A [Source:HGNC Symbol;Acc:HGNC:28403]	HGLibA_51661;HGLibA_51660;HGLibA_51659
ENSG00000124785	NRN1	3	-2.3839	-3.5731	0.042921	0.84121	0.00035282	0.063452	neuritin 1 [Source:HGNC Symbol;Acc:HGNC:17972]	HGLibA_32709;HGLibA_32710;HGLibA_32708
ENSG00000109618	SEPSECS	2	-3.087	-1.3282	0.025504	0.83016	0.18411	0.95326	Sep (O-phosphoserine) tRNA:Sec (selenocysteine) tRNA synthase [Source:HGNC Symbol;Acc:HGNC:30605]	HGLibA_43424;HGLibA_43423
hsa-mir-8064	hsa-mir-8064	3	-2.215	-0.9418	0.0477	0.85856	0.34629	0.95326	-	HGLibA_64095;HGLibA_64096;HGLibA_64094
ENSG00000149480	MTA2	3	-2.9529	-3.7037	0.028445	0.83201	0.00021246	0.046234	metastasis associated 1 family member 2 [Source:HGNC Symbol;Acc:HGNC:7411]	HGLibA_30313;HGLibA_30312;HGLibA_30314
ENSG00000113845	TIMMDC1	1	-3.0874	-2.3186	0.025321	0.83016	0.020418	0.86275	translocase of inner mitochondrial membrane domain containing 1 [Source:HGNC Symbol;Acc:HGNC:1321]	HGLibA_49531
ENSG00000104081	BMF	3	-2.2747	-2.6214	0.045908	0.85666	0.0087571	0.54759	Bcl2 modifying factor [Source:HGNC Symbol;Acc:HGNC:24132]	HGLibA_04583;HGLibA_04584;HGLibA_04585
ENSG00000105100	DHX33	1	-3.6018	-2.654	0.013143	0.82103	0.0079553	0.52301	DEAH-box helicase 33 [Source:HGNC Symbol;Acc:HGNC:16718]	HGLibA_13182
ENSG00000006451	RALA	2	-3.4908	-2.2359	0.01567	0.82103	0.025368	0.95326	RAS like proto-oncogene A [Source:HGNC Symbol;Acc:HGNC:9839]	HGLibA_40147;HGLibA_40148
ENSG00000106772	PRUNE2	3	-2.9842	-3.3753	0.027756	0.83016	0.00073728	0.11299	prune homolog 2 [Source:HGNC Symbol;Acc:HGNC:25209]	HGLibA_38990;HGLibA_38989;HGLibA_38988
ENSG00000090316	MAEA	2	-3.0641	-0.30456	0.026102	0.83016	0.7607	0.95744	macrophage erythroblast attacher [Source:HGNC Symbol;Acc:HGNC:13731]	HGLibA_27861;HGLibA_27860
ENSG00000100353	EIF3D	3	-2.1957	-0.79355	0.048068	0.85995	0.42746	0.95326	eukaryotic translation initiation factor 3 subunit D [Source:HGNC Symbol;Acc:HGNC:3278]	HGLibA_14774;HGLibA_14775;HGLibA_14773
ENSG00000001084	GCLC	1	-2.5122	-1.9593	0.038969	0.83236	0.050081	0.95326	glutamate-cysteine ligase catalytic subunit [Source:HGNC Symbol;Acc:HGNC:4311]	HGLibA_18794
ENSG00000164091	WDR82	1	-2.6447	-2.0403	0.03566	0.83236	0.04132	0.95326	WD repeat domain 82 [Source:HGNC Symbol;Acc:HGNC:28826]	HGLibA_54190
ENSG00000144034	TPRKB	1	-3.5779	-2.6381	0.013602	0.82103	0.0083361	0.53828	TP53RK binding protein [Source:HGNC Symbol;Acc:HGNC:24259]	HGLibA_51187
ENSG00000099783	HNRNPM	2	-3.8536	-2.5796	0.0084095	0.81716	0.0098917	0.58652	heterogeneous nuclear ribonucleoprotein M [Source:HGNC Symbol;Acc:HGNC:5046]	HGLibA_21839;HGLibA_21840
ENSG00000198083	KRTAP9-9	2	-2.1771	-2.4125	0.049262	0.86375	0.015845	0.76114	keratin associated protein 9-9 [Source:HGNC Symbol;Acc:HGNC:16773]	HGLibA_25851;HGLibA_25852
ENSG00000131100	ATP6V1E1	2	-2.1515	-2.3079	0.049676	0.86375	0.021005	0.87904	ATPase H+ transporting V1 subunit E1 [Source:HGNC Symbol;Acc:HGNC:857]	HGLibA_03749;HGLibA_03748
ENSG00000103197	TSC2	3	-3.0353	-1.2452	0.026791	0.83016	0.21306	0.95326	TSC complex subunit 2 [Source:HGNC Symbol;Acc:HGNC:12363]	HGLibA_51814;HGLibA_51813;HGLibA_51815
ENSG00000160799	CCDC12	3	-2.6092	-1.6721	0.036763	0.83236	0.0945	0.95326	coiled-coil domain containing 12 [Source:HGNC Symbol;Acc:HGNC:28332]	HGLibA_07562;HGLibA_07563;HGLibA_07561
ENSG00000214929	SPATA31D1	3	-4.0636	-5.689	0.0056983	0.81716	1.28E-08	3.97E-05	SPATA31 subfamily D member 1 [Source:HGNC Symbol;Acc:HGNC:37283]	HGLibA_46494;HGLibA_46495;HGLibA_46496
hsa-mir-4285	hsa-mir-4285	3	-2.6879	-2.3905	0.035063	0.83236	0.016824	0.78733	-	HGLibA_59848;HGLibA_59847;HGLibA_59849
ENSG00000127483	HP1BP3	2	-2.9715	-1.388	0.027986	0.83016	0.16514	0.95326	heterochromatin protein 1 binding protein 3 [Source:HGNC Symbol;Acc:HGNC:24973]	HGLibA_22013;HGLibA_22012
ENSG00000006695	COX10	1	-3.2346	-2.4134	0.020679	0.83016	0.015806	0.76097	cytochrome c oxidase assembly factor heme A:farnesyltransferase COX10 [Source:HGNC Symbol;Acc:HGNC:2260]	HGLibA_10803
ENSG00000060069	CTDP1	1	-3.6479	-2.6845	0.011994	0.82103	0.0072628	0.49331	CTD phosphatase subunit 1 [Source:HGNC Symbol;Acc:HGNC:2498]	HGLibA_11604
ENSG00000100628	ASB2	2	-4.0513	-4.1517	0.0057442	0.81716	3.30E-05	0.011048	ankyrin repeat and SOCS box containing 2 [Source:HGNC Symbol;Acc:HGNC:16012]	HGLibA_03234;HGLibA_03233
hsa-mir-3118-2	hsa-mir-3118-2	1	-3.692	-2.7138	0.010707	0.82103	0.0066507	0.46238	-	HGLibA_58515
hsa-mir-3118-3	hsa-mir-3118-3	1	-3.1998	-2.3909	0.021782	0.83016	0.016809	0.78733	-	HGLibA_58516
ENSG00000128159	TUBGCP6	2	-2.5225	-2.8145	0.038601	0.83236	0.004885	0.38797	tubulin gamma complex associated protein 6 [Source:HGNC Symbol;Acc:HGNC:18127]	HGLibA_52322;HGLibA_52321
ENSG00000104147	OIP5	2	-2.4126	-3.2728	0.042048	0.84121	0.0010649	0.14855	Opa interacting protein 5 [Source:HGNC Symbol;Acc:HGNC:20300]	HGLibA_33332;HGLibA_33333
ENSG00000187701	OR2T27	2	-3.8753	-3.4407	0.0081338	0.81716	0.00058031	0.094239	olfactory receptor family 2 subfamily T member 27 [Source:HGNC Symbol;Acc:HGNC:31252]	HGLibA_33866;HGLibA_33867
ENSG00000149418	ST14	3	-2.3165	-0.74863	0.044621	0.85175	0.45408	0.95326	suppression of tumorigenicity 14 [Source:HGNC Symbol;Acc:HGNC:11344]	HGLibA_47226;HGLibA_47224;HGLibA_47225
NARG2	NARG2	3	-3.4317	-2.3779	0.016635	0.82103	0.017409	0.80266	-	HGLibA_31149;HGLibA_31150;HGLibA_31148
ENSG00000179862	CITED4	1	-3.0047	-2.2658	0.027251	0.83016	0.023464	0.92867	Cbp/p300 interacting transactivator with Glu/Asp rich carboxy-terminal domain 4 [Source:HGNC Symbol;Acc:HGNC:18696]	HGLibA_09747
ENSG00000150361	KLHL1	2	-3.3262	-0.34433	0.018841	0.82766	0.7306	0.95633	kelch like family member 1 [Source:HGNC Symbol;Acc:HGNC:6352]	HGLibA_25119;HGLibA_25120
ENSG00000188800	TMCO2	2	-2.9664	-1.6639	0.028032	0.83016	0.096138	0.95326	transmembrane and coiled-coil domains 2 [Source:HGNC Symbol;Acc:HGNC:23312]	HGLibA_49784;HGLibA_49785
ENSG00000196557	CACNA1H	3	-3.9942	-4.5715	0.0066173	0.81716	4.84E-06	0.0022949	calcium voltage-gated channel subunit alpha1 H [Source:HGNC Symbol;Acc:HGNC:1395]	HGLibA_06896;HGLibA_06895;HGLibA_06897
ENSG00000065518	NDUFB4	3	-3.426	-3.5887	0.016727	0.82103	0.00033228	0.061277	NADH:ubiquinone oxidoreductase subunit B4 [Source:HGNC Symbol;Acc:HGNC:7699]	HGLibA_31518;HGLibA_31517;HGLibA_31519
ENSG00000131876	SNRPA1	3	-2.8833	-3.8142	0.030054	0.83236	0.0001366	0.033779	small nuclear ribonucleoprotein polypeptide A' [Source:HGNC Symbol;Acc:HGNC:11152]	HGLibA_45966;HGLibA_45964;HGLibA_45965
ENSG00000132780	NASP	3	-3.5622	-3.8255	0.0142	0.82103	0.00013052	0.032647	nuclear autoantigenic sperm protein [Source:HGNC Symbol;Acc:HGNC:7644]	HGLibA_31161;HGLibA_31160;HGLibA_31162
ENSG00000172232	AZU1	2	-3.7524	-4.6016	0.009972	0.81716	4.19E-06	0.0022807	azurocidin 1 [Source:HGNC Symbol;Acc:HGNC:913]	HGLibA_03941;HGLibA_03942
ENSG00000108298	RPL19	1	-3.8691	-2.8323	0.0081338	0.81716	0.0046219	0.37669	ribosomal protein L19 [Source:HGNC Symbol;Acc:HGNC:10312]	HGLibA_41892
ENSG00000162669	HFM1	2	-2.5862	-2.9331	0.036993	0.83236	0.0033559	0.30813	ATP dependent DNA helicase homolog [Source:HGNC Symbol;Acc:HGNC:20193]"	HGLibA_21230;HGLibA_21231
ENSG00000148187	MRRF	3	-3.3014	-5.4108	0.018979	0.82766	6.28E-08	9.75E-05	mitochondrial ribosome recycling factor [Source:HGNC Symbol;Acc:HGNC:7234]	HGLibA_30114;HGLibA_30115;HGLibA_30116
ENSG00000186265	BTLA	2	-2.4623	-2.2298	0.040301	0.83524	0.025761	0.95326	B and T lymphocyte associated [Source:HGNC Symbol;Acc:HGNC:21087]	HGLibA_04957;HGLibA_04955
ENSG00000161542	PRPSAP1	2	-2.4043	-3.1022	0.042186	0.84121	0.0019211	0.21887	phosphoribosyl pyrophosphate synthetase associated protein 1 [Source:HGNC Symbol;Acc:HGNC:9466]	HGLibA_38764;HGLibA_38763;HGLibA_38765
ENSG00000145555	MYO10	3	-3.2044	-2.9151	0.021782	0.83016	0.0035557	0.31712	myosin X [Source:HGNC Symbol;Acc:HGNC:7593]	HGLibA_30802;HGLibA_30803;HGLibA_30801
ENSG00000242028	HYPK	2	-3.3306	-3.6243	0.018795	0.82766	0.00028976	0.056	huntingtin interacting protein K [Source:HGNC Symbol;Acc:HGNC:18418]	HGLibA_22463;HGLibA_22464
ENSG00000176853	FAM91A1	3	-2.248	-2.7528	0.046919	0.85738	0.0059361	0.43434	family with sequence similarity 91 member A1 [Source:HGNC Symbol;Acc:HGNC:26306]	HGLibA_16795;HGLibA_16793;HGLibA_16794
ENSG00000132646	PCNA	1	-2.2729	-1.8159	0.046092	0.85666	0.069387	0.95326	proliferating cell nuclear antigen [Source:HGNC Symbol;Acc:HGNC:8729]	HGLibA_35633
ENSG00000115816	CEBPZ	3	-3.3828	-4.8412	0.017416	0.82609	1.29E-06	0.00096856	CCAAT enhancer binding protein zeta [Source:HGNC Symbol;Acc:HGNC:24218]	HGLibA_08971;HGLibA_08972;HGLibA_08970
ENSG00000108592	FTSJ3	2	-3.6726	-2.73	0.011397	0.82103	0.0063329	0.45014	FtsJ RNA methyltransferase homolog 3 [Source:HGNC Symbol;Acc:HGNC:17136]	HGLibA_18140;HGLibA_18139
ENSG00000177535	OR2B11	2	-3.2004	-3.6662	0.021782	0.83016	0.00024616	0.051507	olfactory receptor family 2 subfamily B member 11 [Source:HGNC Symbol;Acc:HGNC:31249]	HGLibA_33764;HGLibA_33763
hsa-mir-297	hsa-mir-297	1	-2.5806	-2.001	0.037131	0.83236	0.045392	0.95326	-	HGLibA_58399
ENSG00000196800	SPINK14	3	-3.1599	-5.1035	0.023299	0.83016	3.33E-07	0.00040143	Kazal type 14 (putative) [Source:HGNC Symbol;Acc:HGNC:33825]"	HGLibA_46688;HGLibA_46687;HGLibA_46689
ENSG00000164327	RICTOR	2	-2.7496	-2.2213	0.033868	0.83236	0.026333	0.95326	RPTOR independent companion of MTOR complex 2 [Source:HGNC Symbol;Acc:HGNC:28611]	HGLibA_41241;HGLibA_41242
ENSG00000159063	ALG8	2	-3.0475	-0.42415	0.026332	0.83016	0.67146	0.95326	3-glucosyltransferase [Source:HGNC Symbol;Acc:HGNC:23161]"	HGLibA_01637;HGLibA_01636
hsa-mir-548t	hsa-mir-548t	4	-2.5191	-1.8629	0.038693	0.83236	0.062474	0.95326	-	HGLibA_62065;HGLibA_62068;HGLibA_62066;HGLibA_62067
ENSG00000124587	PEX6	3	-2.7186	-2.6439	0.03419	0.83236	0.0081949	0.53552	peroxisomal biogenesis factor 6 [Source:HGNC Symbol;Acc:HGNC:8859]	HGLibA_36122;HGLibA_36123;HGLibA_36124
ENSG00000277791	PSMB3	2	-3.1899	-2.0986	0.022288	0.83016	0.035849	0.95326	proteasome subunit beta 3 [Source:HGNC Symbol;Acc:HGNC:9540]	HGLibA_39105;HGLibA_39107
ENSG00000189403	HMGB1	3	-3.1145	-1.5641	0.024815	0.83016	0.11779	0.95326	high mobility group box 1 [Source:HGNC Symbol;Acc:HGNC:4983]	HGLibA_21690;HGLibA_21691;HGLibA_21689
ENSG00000166200	COPS2	3	-2.4912	-1.8747	0.039704	0.83524	0.060832	0.95326	COP9 signalosome subunit 2 [Source:HGNC Symbol;Acc:HGNC:30747]	HGLibA_10712;HGLibA_10713;HGLibA_10714
ENSG00000101546	RBFA	2	-2.7996	-2.9366	0.032673	0.83236	0.0033183	0.30813	ribosome binding factor A [Source:HGNC Symbol;Acc:HGNC:26120]	HGLibA_40451;HGLibA_40453
ENSG00000078369	GNB1	3	-2.5456	-0.71829	0.038142	0.83236	0.47258	0.95326	G protein subunit beta 1 [Source:HGNC Symbol;Acc:HGNC:4396]	HGLibA_19484;HGLibA_19483;HGLibA_19485
IARS	IARS	1	-2.866	-2.1781	0.030513	0.83236	0.029399	0.95326	-	HGLibA_22472
ENSG00000116120	FARSB	1	-2.4386	-1.9148	0.040945	0.83741	0.055519	0.95326	phenylalanyl-tRNA synthetase subunit beta [Source:HGNC Symbol;Acc:HGNC:17800]	HGLibA_16887
ENSG00000183396	TMEM89	2	-3.2303	-3.3764	0.021185	0.83016	0.00073441	0.11299	transmembrane protein 89 [Source:HGNC Symbol;Acc:HGNC:32372]	HGLibA_50544;HGLibA_50542
ENSG00000153107	ANAPC1	1	-2.9606	-2.2378	0.028078	0.83016	0.025235	0.95326	anaphase promoting complex subunit 1 [Source:HGNC Symbol;Acc:HGNC:19988]	HGLibA_01862
ENSG00000179046	TRIML2	2	-2.712	-0.38958	0.03419	0.83236	0.69684	0.95516	tripartite motif family like 2 [Source:HGNC Symbol;Acc:HGNC:26378]	HGLibA_51624;HGLibA_51623
ENSG00000185666	SYN3	3	-4.11	-5.973	0.005009	0.81716	2.33E-09	8.45E-06	synapsin III [Source:HGNC Symbol;Acc:HGNC:11496]	HGLibA_47960;HGLibA_47962;HGLibA_47961
ENSG00000114127	XRN1	3	-3.0915	-1.3561	0.025229	0.83016	0.17508	0.95326	5'-3' exoribonuclease 1 [Source:HGNC Symbol;Acc:HGNC:30654]	HGLibA_54604;HGLibA_54603;HGLibA_54605
ENSG00000115524	SF3B1	3	-3.0398	-3.5806	0.026607	0.83016	0.00034275	0.062155	splicing factor 3b subunit 1 [Source:HGNC Symbol;Acc:HGNC:10768]	HGLibA_43701;HGLibA_43699
ENSG00000186474	KLK12	3	-3.1804	-1.8461	0.022425	0.83016	0.064879	0.95326	kallikrein related peptidase 12 [Source:HGNC Symbol;Acc:HGNC:6360]	HGLibA_25240;HGLibA_25241;HGLibA_25239
ENSG00000074695	LMAN1	3	-3.7514	-2.3484	0.009972	0.81716	0.018855	0.84318	mannose binding 1 [Source:HGNC Symbol;Acc:HGNC:6631]"	HGLibA_26658;HGLibA_26657;HGLibA_26656
ENSG00000067955	CBFB	2	-2.2755	-2.3653	0.045908	0.85666	0.018014	0.8184	core-binding factor subunit beta [Source:HGNC Symbol;Acc:HGNC:1539]	HGLibA_07390;HGLibA_07391
ENSG00000141252	VPS53	3	-3.0912	-3.5933	0.025229	0.83016	0.00032647	0.06072	GARP complex subunit [Source:HGNC Symbol;Acc:HGNC:25608]"	HGLibA_53810;HGLibA_53809;HGLibA_53808
ENSG00000171311	EXOSC1	2	-3.2849	-4.2197	0.019622	0.83016	2.45E-05	0.0084562	exosome component 1 [Source:HGNC Symbol;Acc:HGNC:17286]	HGLibA_15768;HGLibA_15770
ENSG00000170855	TRIAP1	1	-3.5534	-2.622	0.014246	0.82103	0.0087425	0.54759	TP53 regulated inhibitor of apoptosis 1 [Source:HGNC Symbol;Acc:HGNC:26937]	HGLibA_51388
ENSG00000284733	OR4F29	3	-2.8939	-2.6147	0.029916	0.83236	0.0089312	0.55381	olfactory receptor family 4 subfamily F member 29 [Source:HGNC Symbol;Acc:HGNC:31275]	HGLibA_34000;HGLibA_34001;HGLibA_34002
ENSG00000253710	ALG11	2	-3.666	-4.5761	0.01181	0.82103	4.74E-06	0.0022949	2-mannosyltransferase [Source:HGNC Symbol;Acc:HGNC:32456]"	HGLibA_01609;HGLibA_01611
ENSG00000132432	SEC61G	2	-2.3646	-1.7691	0.043151	0.84121	0.076879	0.95326	Sec61 translocon gamma subunit [Source:HGNC Symbol;Acc:HGNC:18277]	HGLibA_43257;HGLibA_43256
ENSG00000241233	KRTAP5-8	2	-2.2439	-1.911	0.047011	0.85738	0.056008	0.95326	keratin associated protein 5-8 [Source:HGNC Symbol;Acc:HGNC:23603]	HGLibA_25810;HGLibA_25809
ENSG00000163909	HEYL	3	-2.3087	-1.9954	0.044805	0.85377	0.045995	0.95326	hes related family bHLH transcription factor with YRPW motif-like [Source:HGNC Symbol;Acc:HGNC:4882]	HGLibA_21222;HGLibA_21223;HGLibA_21221
ENSG00000174282	ZBTB4	2	-2.7472	-3.506	0.033868	0.83236	0.00045498	0.077959	zinc finger and BTB domain containing 4 [Source:HGNC Symbol;Acc:HGNC:23847]	HGLibA_54877;HGLibA_54878
ENSG00000115133	CCDC88C	1	-3.449	-2.5532	0.016268	0.82103	0.010675	0.60337	coiled-coil domain containing 88C [Source:HGNC Symbol;Acc:HGNC:19967]	HGLibA_07912
ENSG00000139718	SETD1B	2	-3.5418	-3.422	0.014705	0.82103	0.00062158	0.099728	SET domain containing 1B [Source:HGNC Symbol;Acc:HGNC:29187]	HGLibA_43639;HGLibA_43640
ENSG00000187688	TRPV2	1	-3.1263	-2.3435	0.023988	0.83016	0.019102	0.84318	transient receptor potential cation channel subfamily V member 2 [Source:HGNC Symbol;Acc:HGNC:18082]	HGLibA_51784
hsa-mir-4448	hsa-mir-4448	2	-3.6534	-4.2829	0.011948	0.82103	1.84E-05	0.0069212	-	HGLibA_60200;HGLibA_60198
ENSG00000186226	LCE1E	3	-3.9867	-4.8416	0.0067552	0.81716	1.29E-06	0.00096856	late cornified envelope 1E [Source:HGNC Symbol;Acc:HGNC:29466]	HGLibA_26112;HGLibA_26111;HGLibA_26113
ENSG00000165280	VCP	1	-2.358	-1.8527	0.04361	0.84581	0.06392	0.95326	valosin containing protein [Source:HGNC Symbol;Acc:HGNC:12666]	HGLibA_53576
ENSG00000183763	TRAIP	2	-2.3403	-2.4709	0.043886	0.84738	0.013478	0.68498	TRAF interacting protein [Source:HGNC Symbol;Acc:HGNC:30764]	HGLibA_51274;HGLibA_51273
ENSG00000152601	MBNL1	2	-2.4973	-1.9351	0.039474	0.83398	0.052973	0.95326	muscleblind like splicing regulator 1 [Source:HGNC Symbol;Acc:HGNC:6923]	HGLibA_28508;HGLibA_28509
ENSG00000178691	SUZ12	1	-3.5283	-2.6054	0.014843	0.82103	0.0091769	0.56412	polycomb repressive complex 2 subunit [Source:HGNC Symbol;Acc:HGNC:17101]"	HGLibA_47872
hsa-mir-6087	hsa-mir-6087	1	-2.1783	-1.7604	0.048849	0.86282	0.078347	0.95326	-	HGLibA_62609
PAPL	PAPL	3	-3.3789	-3.4504	0.017462	0.82609	0.00055973	0.092275	-	HGLibA_35129;HGLibA_35131;HGLibA_35130
ENSG00000176896	TCEANC	2	-2.8774	-0.84471	0.030238	0.83236	0.39827	0.95326	transcription elongation factor A N-terminal and central domain containing [Source:HGNC Symbol;Acc:HGNC:28277]	HGLibA_48728;HGLibA_48727
ENSG00000159377	PSMB4	3	-2.3	-0.77452	0.045035	0.8559	0.43862	0.95326	proteasome subunit beta 4 [Source:HGNC Symbol;Acc:HGNC:9541]	HGLibA_39109;HGLibA_39110;HGLibA_39108
ENSG00000091844	RGS17	3	-3.1848	-5.0941	0.022425	0.83016	3.50E-07	0.00040143	regulator of G protein signaling 17 [Source:HGNC Symbol;Acc:HGNC:14088]	HGLibA_41071;HGLibA_41070;HGLibA_41069
ENSG00000100387	RBX1	1	-3.0469	-2.2927	0.026332	0.83016	0.021866	0.89948	ring-box 1 [Source:HGNC Symbol;Acc:HGNC:9928]	HGLibA_40660
C17orf96	C17orf96	3	-3.6283	-4.3739	0.012683	0.82103	1.22E-05	0.0048281	-	HGLibA_05593;HGLibA_05595;HGLibA_05594
hsa-mir-4472-2	hsa-mir-4472-2	1	-2.7048	-2.0775	0.034282	0.83236	0.03776	0.95326	-	HGLibA_60297
ENSG00000204264	PSMB8	2	-3.849	-2.5869	0.0086393	0.81716	0.0096835	0.58211	proteasome subunit beta 8 [Source:HGNC Symbol;Acc:HGNC:9545]	HGLibA_39122;HGLibA_39121
ENSG00000115760	BIRC6	3	-3.5251	-5.4883	0.015119	0.82103	4.06E-08	7.00E-05	baculoviral IAP repeat containing 6 [Source:HGNC Symbol;Acc:HGNC:13516]	HGLibA_04525;HGLibA_04523;HGLibA_04524
ENSG00000158526	TSR2	2	-2.5937	-3.3496	0.036901	0.83236	0.00080926	0.12062	ribosome maturation factor [Source:HGNC Symbol;Acc:HGNC:25455]"	HGLibA_51998;HGLibA_51997
ENSG00000126858	RHOT1	3	-3.9112	-5.526	0.0077202	0.81716	3.28E-08	6.64E-05	ras homolog family member T1 [Source:HGNC Symbol;Acc:HGNC:21168]	HGLibA_41201;HGLibA_41203;HGLibA_41202
ENSG00000048544	MRPS10	2	-3.0968	-2.4915	0.025229	0.83016	0.012722	0.66547	mitochondrial ribosomal protein S10 [Source:HGNC Symbol;Acc:HGNC:14502]	HGLibA_30030;HGLibA_30028
hsa-mir-1181	hsa-mir-1181	4	-2.6386	-2.0606	0.036166	0.83236	0.03934	0.95326	-	HGLibA_57218;HGLibA_57217;HGLibA_57216;HGLibA_57219
ENSG00000170469	SPATA24	3	-2.1866	-1.9712	0.048435	0.86182	0.0487	0.95326	spermatogenesis associated 24 [Source:HGNC Symbol;Acc:HGNC:27322]	HGLibA_46457;HGLibA_46456;HGLibA_46455
ENSG00000101191	DIDO1	1	-4.5287	-3.2809	0.0015165	0.78846	0.0010347	0.1462	death inducer-obliterator 1 [Source:HGNC Symbol;Acc:HGNC:2680]	HGLibA_13229
LPHN3	LPHN3	2	-3.8585	-2.3132	0.0082717	0.81716	0.020709	0.87118	-	HGLibA_27091;HGLibA_27092
ENSG00000276043	UHRF1	3	-2.2331	-0.61436	0.047332	0.85762	0.53897	0.95326	ubiquitin like with PHD and ring finger domains 1 [Source:HGNC Symbol;Acc:HGNC:12556]	HGLibA_52944;HGLibA_52945;HGLibA_52946
ENSG00000254647	INS	2	-4.0835	-1.3828	0.0054225	0.81716	0.16673	0.95326	insulin [Source:HGNC Symbol;Acc:HGNC:6081]	HGLibA_23366;HGLibA_23368
ENSG00000170312	CDK1	1	-3.3007	-2.4563	0.018979	0.82766	0.014039	0.70229	cyclin dependent kinase 1 [Source:HGNC Symbol;Acc:HGNC:1722]	HGLibA_08718
ENSG00000124571	XPO5	2	-2.8803	-3.6433	0.030054	0.83236	0.00026918	0.054602	exportin 5 [Source:HGNC Symbol;Acc:HGNC:17675]	HGLibA_54568;HGLibA_54567
ENSG00000185728	YTHDF3	1	-2.5015	-1.9381	0.039474	0.83398	0.052609	0.95326	YTH N6-methyladenosine RNA binding protein 3 [Source:HGNC Symbol;Acc:HGNC:26465]	HGLibA_54736
ZNF259	ZNF259	3	-3.3653	-4.2461	0.017692	0.82766	2.18E-05	0.0078902	-	HGLibA_55684;HGLibA_55683;HGLibA_55685
ENSG00000275066	SYNRG	3	-2.3035	-2.6411	0.044851	0.85389	0.0082633	0.53723	synergin gamma [Source:HGNC Symbol;Acc:HGNC:557]	HGLibA_48029;HGLibA_48031;HGLibA_48030
ENSG00000174332	GLIS1	3	-2.1997	-2.875	0.048022	0.85995	0.0040407	0.34504	GLIS family zinc finger 1 [Source:HGNC Symbol;Acc:HGNC:29525]	HGLibA_19270;HGLibA_19272;HGLibA_19271
ENSG00000115568	ZNF142	3	-2.683	-0.84908	0.035109	0.83236	0.39584	0.95326	zinc finger protein 142 [Source:HGNC Symbol;Acc:HGNC:12927]	HGLibA_55488;HGLibA_55490;HGLibA_55489
ENSG00000172426	RSPH9	2	-3.1908	-2.3291	0.022288	0.83016	0.019852	0.85808	radial spoke head 9 homolog [Source:HGNC Symbol;Acc:HGNC:21057]	HGLibA_42342;HGLibA_42340
hsa-mir-6835	hsa-mir-6835	1	-4.0193	-2.9335	0.0061578	0.81716	0.0033521	0.30813	-	HGLibA_63517
ENSG00000204246	OR13C3	2	-3.8416	-4.7119	0.0086393	0.81716	2.45E-06	0.0015711	olfactory receptor family 13 subfamily C member 3 [Source:HGNC Symbol;Acc:HGNC:14704]	HGLibA_33598;HGLibA_33600
ENSG00000204160	ZDHHC18	2	-4.2235	-5.2807	0.0037682	0.81716	1.29E-07	0.00017502	zinc finger DHHC-type containing 18 [Source:HGNC Symbol;Acc:HGNC:20712]	HGLibA_55090;HGLibA_55091
ENSG00000067225	PKM	3	-3.4567	-2.1214	0.016268	0.82103	0.033885	0.95326	pyruvate kinase M1/2 [Source:HGNC Symbol;Acc:HGNC:9021]	HGLibA_36831;HGLibA_36832;HGLibA_36830
ENSG00000123213	NLN	1	-3.3322	-2.4768	0.018519	0.82766	0.013258	0.67724	neurolysin [Source:HGNC Symbol;Acc:HGNC:16058]	HGLibA_32085
ENSG00000128604	IRF5	2	-4.9572	-6.0027	0.00018382	0.66667	1.94E-09	8.45E-06	interferon regulatory factor 5 [Source:HGNC Symbol;Acc:HGNC:6120]	HGLibA_23592;HGLibA_23591
ENSG00000250479	CHCHD10	3	-2.7226	-3.1921	0.034052	0.83236	0.0014124	0.17412	coiled-coil-helix-coiled-coil-helix domain containing 10 [Source:HGNC Symbol;Acc:HGNC:15559]	HGLibA_09363;HGLibA_09362;HGLibA_09361
hsa-mir-4487	hsa-mir-4487	4	-3.4084	-5.5711	0.017095	0.82119	2.53E-08	6.12E-05	-	HGLibA_60368;HGLibA_60367;HGLibA_60365;HGLibA_60366
ENSG00000125691	RPL23	1	-2.2729	-1.8159	0.046092	0.85666	0.069387	0.95326	ribosomal protein L23 [Source:HGNC Symbol;Acc:HGNC:10316]	HGLibA_41903
hsa-mir-3134	hsa-mir-3134	3	-3.2586	-3.0379	0.020358	0.83016	0.0023822	0.253	-	HGLibA_58592;HGLibA_58591;HGLibA_58589
ENSG00000148019	CEP78	3	-2.3221	-2.2365	0.044529	0.85175	0.025318	0.95326	centrosomal protein 78 [Source:HGNC Symbol;Acc:HGNC:25740]	HGLibA_09168;HGLibA_09166;HGLibA_09167
ENSG00000198841	KTI12	3	-3.4873	-2.689	0.01567	0.82103	0.0071666	0.49042	KTI12 chromatin associated homolog [Source:HGNC Symbol;Acc:HGNC:25160]	HGLibA_25868;HGLibA_25869;HGLibA_25870
hsa-mir-4509-1	hsa-mir-4509-1	3	-2.1423	-2.1205	0.049814	0.86375	0.033964	0.95326	-	HGLibA_60463;HGLibA_60464;HGLibA_60462
ENSG00000110063	DCPS	1	-2.2729	-1.8159	0.046092	0.85666	0.069387	0.95326	scavenger [Source:HGNC Symbol;Acc:HGNC:29812]"	HGLibA_12526
ENSG00000123684	LPGAT1	3	-2.656	-0.65375	0.035522	0.83236	0.51327	0.95326	lysophosphatidylglycerol acyltransferase 1 [Source:HGNC Symbol;Acc:HGNC:28985]	HGLibA_27083;HGLibA_27082;HGLibA_27084
ENSG00000249481	SPATS1	2	-3.1281	-0.43763	0.023942	0.83016	0.66165	0.95326	spermatogenesis associated serine rich 1 [Source:HGNC Symbol;Acc:HGNC:22957]	HGLibA_46544;HGLibA_46542
ENSG00000146049	KAAG1	1	-2.3592	-1.8672	0.043288	0.84121	0.061879	0.95326	kidney associated antigen 1 [Source:HGNC Symbol;Acc:HGNC:21031]	HGLibA_24015
ENSG00000084774	CAD	1	-2.8152	-2.1462	0.031984	0.83236	0.031859	0.95326	and dihydroorotase [Source:HGNC Symbol;Acc:HGNC:1424]"	HGLibA_06961
ENSG00000133135	RNF128	3	-2.4635	-2.6925	0.040256	0.83524	0.0070917	0.48682	E3 ubiquitin protein ligase [Source:HGNC Symbol;Acc:HGNC:21153]"	HGLibA_41500;HGLibA_41499;HGLibA_41498
ENSG00000163626	COX18	2	-2.721	-2.9119	0.034052	0.83236	0.0035927	0.31781	cytochrome c oxidase assembly factor COX18 [Source:HGNC Symbol;Acc:HGNC:26801]	HGLibA_10820;HGLibA_10821
ENSG00000137968	SLC44A5	2	-2.6526	-2.8105	0.035522	0.83236	0.0049457	0.39065	solute carrier family 44 member 5 [Source:HGNC Symbol;Acc:HGNC:28524]	HGLibA_45170;HGLibA_45168
ENSG00000119421	NDUFA8	1	-3.4761	-2.571	0.015854	0.82103	0.01014	0.58686	NADH:ubiquinone oxidoreductase subunit A8 [Source:HGNC Symbol;Acc:HGNC:7692]	HGLibA_31473
ENSG00000071626	DAZAP1	1	-2.6447	-2.0403	0.03566	0.83236	0.04132	0.95326	DAZ associated protein 1 [Source:HGNC Symbol;Acc:HGNC:2683]	HGLibA_12365
ENSG00000146576	C7orf26	1	-2.1972	-1.7607	0.048068	0.85995	0.078295	0.95326	chromosome 7 open reading frame 26 [Source:HGNC Symbol;Acc:HGNC:21702]	HGLibA_06527
ENSG00000006007	GDE1	2	-3.9444	-3.4512	0.0073066	0.81716	0.00055802	0.092275	glycerophosphodiester phosphodiesterase 1 [Source:HGNC Symbol;Acc:HGNC:29644]	HGLibA_18848;HGLibA_18849
ENSG00000068308	OTUD5	2	-3.857	-3.8398	0.0083176	0.81716	0.00012314	0.03116	OTU deubiquitinase 5 [Source:HGNC Symbol;Acc:HGNC:25402]	HGLibA_34769;HGLibA_34767
ENSG00000075624	ACTB	3	-2.221	-2.7035	0.047654	0.85844	0.0068613	0.47551	actin beta [Source:HGNC Symbol;Acc:HGNC:132]	HGLibA_00602;HGLibA_00601;HGLibA_00603
ENSG00000168286	THAP11	3	-3.0346	-0.42444	0.026791	0.83016	0.67125	0.95326	THAP domain containing 11 [Source:HGNC Symbol;Acc:HGNC:23194]	HGLibA_49291;HGLibA_49292;HGLibA_49293
hsa-mir-1302-5	hsa-mir-1302-5	2	-2.56	-3.3461	0.037728	0.83236	0.00081947	0.12131	-	HGLibA_57707;HGLibA_57709
hsa-mir-544a	hsa-mir-544a	4	-3.5178	-6.0051	0.015165	0.82103	1.91E-09	8.45E-06	-	HGLibA_61826;HGLibA_61824;HGLibA_61825;HGLibA_61823
ENSG00000147162	OGT	2	-2.6266	-0.83821	0.036487	0.83236	0.40191	0.95326	O-linked N-acetylglucosamine (GlcNAc) transferase [Source:HGNC Symbol;Acc:HGNC:8127]	HGLibA_33330;HGLibA_33329
ENSG00000127337	YEATS4	2	-2.4167	-1.8983	0.041956	0.84121	0.057659	0.95326	YEATS domain containing 4 [Source:HGNC Symbol;Acc:HGNC:24859]	HGLibA_54657;HGLibA_54659
ENSG00000055044	NOP58	2	-2.5581	-2.982	0.03782	0.83236	0.0028641	0.27961	NOP58 ribonucleoprotein [Source:HGNC Symbol;Acc:HGNC:29926]	HGLibA_32316;HGLibA_32315
ENSG00000204669	C9orf57	3	-2.7233	-0.59318	0.034052	0.83236	0.55306	0.95326	chromosome 9 open reading frame 57 [Source:HGNC Symbol;Acc:HGNC:27037]	HGLibA_06742;HGLibA_06744;HGLibA_06743
ENSG00000100567	PSMA3	2	-3.5182	-3.9879	0.015165	0.82103	6.67E-05	0.019341	proteasome subunit alpha 3 [Source:HGNC Symbol;Acc:HGNC:9532]	HGLibA_39077;HGLibA_39076
ENSG00000157895	C12orf43	2	-2.4771	-0.87513	0.040118	0.83524	0.3815	0.95326	chromosome 12 open reading frame 43 [Source:HGNC Symbol;Acc:HGNC:25719]	HGLibA_05244;HGLibA_05243
ENSG00000100697	DICER1	2	-2.5186	-1.9528	0.038693	0.83236	0.050848	0.95326	ribonuclease III [Source:HGNC Symbol;Acc:HGNC:17098]"	HGLibA_13225;HGLibA_13227
ENSG00000182473	EXOC7	2	-3.6527	-1.8886	0.011948	0.82103	0.058949	0.95326	exocyst complex component 7 [Source:HGNC Symbol;Acc:HGNC:23214]	HGLibA_15759;HGLibA_15761
ATPBD4	ATPBD4	1	-3.1637	-2.3676	0.022793	0.83016	0.017904	0.81508	-	HGLibA_03804
ENSG00000142676	RPL11	2	-2.7169	-3.1911	0.03419	0.83236	0.0014172	0.17412	ribosomal protein L11 [Source:HGNC Symbol;Acc:HGNC:10301]	HGLibA_41861;HGLibA_41863
ENSG00000105619	TFPT	2	-2.7237	-3.388	0.034052	0.83236	0.00070411	0.11023	TCF3 fusion partner [Source:HGNC Symbol;Acc:HGNC:13630]	HGLibA_49190;HGLibA_49191
ENSG00000176387	HSD11B2	1	-3.449	-2.5532	0.016268	0.82103	0.010675	0.60337	hydroxysteroid 11-beta dehydrogenase 2 [Source:HGNC Symbol;Acc:HGNC:5209]	HGLibA_22167
ENSG00000136997	MYC	1	-3.1263	-2.3435	0.023988	0.83016	0.019102	0.84318	bHLH transcription factor [Source:HGNC Symbol;Acc:HGNC:7553]"	HGLibA_30665
ENSG00000175416	CLTB	2	-2.7345	-2.8735	0.034006	0.83236	0.0040591	0.34504	clathrin light chain B [Source:HGNC Symbol;Acc:HGNC:2091]	HGLibA_10146;HGLibA_10147
ENSG00000104341	LAPTM4B	3	-2.6021	-3.0136	0.036809	0.83236	0.0025816	0.26593	lysosomal protein transmembrane 4 beta [Source:HGNC Symbol;Acc:HGNC:13646]	HGLibA_26020;HGLibA_26018;HGLibA_26019
ENSG00000214114	MYCBP	1	-4.2026	-3.0578	0.003952	0.81716	0.0022299	0.24089	MYC binding protein [Source:HGNC Symbol;Acc:HGNC:7554]	HGLibA_30667
ENSG00000168615	ADAM9	3	-3.0367	-1.3857	0.026607	0.83016	0.16584	0.95326	ADAM metallopeptidase domain 9 [Source:HGNC Symbol;Acc:HGNC:216]	HGLibA_00786;HGLibA_00785;HGLibA_00784
ENSG00000147123	NDUFB11	1	-4.3682	-3.1708	0.0025275	0.81716	0.0015203	0.18177	NADH:ubiquinone oxidoreductase subunit B11 [Source:HGNC Symbol;Acc:HGNC:20372]	HGLibA_31510
ENSG00000127837	AAMP	2	-2.9758	-3.8508	0.027848	0.83016	0.00011775	0.030504	angio associated migratory cell protein [Source:HGNC Symbol;Acc:HGNC:18]	HGLibA_00056;HGLibA_00057
ENSG00000119913	TECTB	2	-2.1475	-2.1978	0.049768	0.86375	0.027967	0.95326	tectorin beta [Source:HGNC Symbol;Acc:HGNC:11721]	HGLibA_48955;HGLibA_48956
ENSG00000130741	EIF2S3	1	-2.5806	-2.001	0.037131	0.83236	0.045392	0.95326	eukaryotic translation initiation factor 2 subunit gamma [Source:HGNC Symbol;Acc:HGNC:3267]	HGLibA_14758

ENSG00000182118	FAM89A	2	-2.2371	-2.802	0.04724	0.85738	0.0050789	0.39472	family with sequence similarity 89 member A [Source:HGNC Symbol;Acc:HGNC:25057]	HGLibA_16781;HGLibA_16782
ENSG00000141385	AFG3L2	3	-3.0549	-2.6146	0.026332	0.83016	0.0089328	0.55381	AFG3 like matrix AAA peptidase subunit 2 [Source:HGNC Symbol;Acc:HGNC:315]	HGLibA_01119;HGLibA_01117;HGLibA_01118
hsa-mir-3687	hsa-mir-3687	1	-3.1998	-2.3909	0.021782	0.83016	0.016809	0.78733	-	HGLibA_59302
ENSG00000225362	CT62	2	-3.4469	-1.4677	0.016497	0.82103	0.14218	0.95326	cancer/testis antigen 62 [Source:HGNC Symbol;Acc:HGNC:27286]	HGLibA_11555;HGLibA_11556
ENSG00000214253	FIS1	2	-3.1208	-1.1381	0.024723	0.83016	0.25506	0.95326	mitochondrial 1 [Source:HGNC Symbol;Acc:HGNC:21689]"	HGLibA_17536;HGLibA_17538
ENSG00000174744	BRMS1	2	-2.3187	-0.45126	0.044529	0.85175	0.6518	0.95326	transcriptional repressor and anoikis regulator [Source:HGNC Symbol;Acc:HGNC:17262]"	HGLibA_04831;HGLibA_04830
ENSG00000077514	POLD3	2	-2.2744	-1.0175	0.045908	0.85666	0.30891	0.95326	accessory subunit [Source:HGNC Symbol;Acc:HGNC:20932]"	HGLibA_37498;HGLibA_37499
ENSG00000198258	UBL5	1	-3.3007	-2.4563	0.018979	0.82766	0.014039	0.70229	ubiquitin like 5 [Source:HGNC Symbol;Acc:HGNC:13736]	HGLibA_52712
ENSG00000206127	GOLGA8O	2	-2.3659	-1.3812	0.043151	0.84121	0.16723	0.95326	golgin A8 family member O [Source:HGNC Symbol;Acc:HGNC:44406]	HGLibA_19646;HGLibA_19647
ENSG00000074800	ENO1	2	-2.881	-3.7738	0.030054	0.83236	0.00016076	0.038582	enolase 1 [Source:HGNC Symbol;Acc:HGNC:3350]	HGLibA_15141;HGLibA_15140
hsa-mir-548ae-2	hsa-mir-548ae-2	2	-2.4865	-2.8457	0.03998	0.83524	0.004432	0.36692	-	HGLibA_61871;HGLibA_61872
ENSG00000075188	NUP37	1	-3.8135	-2.795	0.008961	0.81716	0.0051901	0.39909	nucleoporin 37 [Source:HGNC Symbol;Acc:HGNC:29929]	HGLibA_33051
ENSG00000198758	EPS8L3	2	-2.5916	-2.9553	0.036947	0.83236	0.0031241	0.29818	EPS8 like 3 [Source:HGNC Symbol;Acc:HGNC:21297]	HGLibA_15382;HGLibA_15383
ENSG00000131269	ABCB7	3	-3.4195	-2.9128	0.017003	0.82119	0.0035825	0.31781	ATP binding cassette subfamily B member 7 [Source:HGNC Symbol;Acc:HGNC:48]	HGLibA_00148;HGLibA_00150;HGLibA_00149
ENSG00000091157	WDR7	2	-4.0531	-3.5376	0.0057442	0.81716	0.00040383	0.070746	WD repeat domain 7 [Source:HGNC Symbol;Acc:HGNC:13490]	HGLibA_54161;HGLibA_54159
ENSG00000165416	SUGT1	2	-3.0381	-1.3801	0.026607	0.83016	0.014723	0.17799	MIS12 kinetochore complex assembly cochaperone [Source:HGNC Symbol;Acc:HGNC:16987]"	HGLibA_47722;HGLibA_47723
ENSG00000186051	TAL2	2	-2.1751	-2.3456	0.049262	0.86375	0.018994	0.84318	TAL bHLH transcription factor 2 [Source:HGNC Symbol;Acc:HGNC:11557]	HGLibA_48283;HGLibA_48282
ENSG00000144771	LRTM1	1	-2.3592	-1.8672	0.043288	0.84121	0.061879	0.95326	leucine rich repeats and transmembrane domains 1 [Source:HGNC Symbol;Acc:HGNC:25023]	HGLibA_27499
ENSG00000175283	DOLK	3	-3.1368	-1.3492	0.023804	0.83016	0.17729	0.95326	dolichol kinase [Source:HGNC Symbol;Acc:HGNC:23406]	HGLibA_13787;HGLibA_13789;HGLibA_13788
ENSG00000106459	NRF1	3	-3.2854	-4.056	0.019622	0.83016	4.99E-05	0.015303	nuclear respiratory factor 1 [Source:HGNC Symbol;Acc:HGNC:7996]	HGLibA_32673;HGLibA_32672;HGLibA_32674
ENSG00000119138	KLF9	2	-2.6096	-3.4144	0.036763	0.83236	0.0006392	0.10079	Kruppel like factor 9 [Source:HGNC Symbol;Acc:HGNC:1123]	HGLibA_25087;HGLibA_25088
ENSG00000089597	GANAB	2	-2.3621	-2.9779	0.043151	0.84121	0.0029019	0.28191	glucosidase II alpha subunit [Source:HGNC Symbol;Acc:HGNC:4138]	HGLibA_18605;HGLibA_18606
ENSG00000176225	RTTN	3	-3.6193	-3.1897	0.012959	0.82103	0.0014242	0.17412	rotatin [Source:HGNC Symbol;Acc:HGNC:18654]	HGLibA_42433;HGLibA_42435;HGLibA_42434
ENSG00000157765	SLC34A2	3	-2.6045	-0.50456	0.036809	0.83236	0.61387	0.95326	solute carrier family 34 member 2 [Source:HGNC Symbol;Acc:HGNC:11020]	HGLibA_44940;HGLibA_44941;HGLibA_44942
ENSG00000149295	DRD2	2	-2.9953	-3.4435	0.027526	0.83016	0.0005742	0.093948	dopamine receptor D2 [Source:HGNC Symbol;Acc:HGNC:3023]	HGLibA_13961;HGLibA_13963
ENSG00000149294	NCAM1	2	-4.101	-0.39816	0.0052387	0.81716	0.69051	0.95446	neural cell adhesion molecule 1 [Source:HGNC Symbol;Acc:HGNC:7656]	HGLibA_31255;HGLibA_31253
ENSG00000184029	DSCR4	2	-2.7273	-2.8381	0.034006	0.83236	0.0045382	0.3716	Down syndrome critical region 4 [Source:HGNC Symbol;Acc:HGNC:3045]	HGLibA_14010;HGLibA_14011
ENSG00000164934	DCAF13	2	-3.3593	-3.0104	0.018152	0.82766	0.0026099	0.26653	DDB1 and CUL4 associated factor 13 [Source:HGNC Symbol;Acc:HGNC:24535]	HGLibA_12428;HGLibA_12427
ENSG00000108055	SMC3	2	-2.7726	-2.9681	0.033179	0.83236	0.0029965	0.28981	structural maintenance of chromosomes 3 [Source:HGNC Symbol;Acc:HGNC:2468]	HGLibA_45650;HGLibA_45649
TMEM55B	TMEM55B	1	-3.9554	-2.8903	0.0070769	0.81716	0.0038489	0.33435	-	HGLibA_50446
ENSG00000204120	GIGYF2	3	-2.9327	-4.5166	0.028721	0.83201	6.28E-06	0.0027903	GRB10 interacting GYF protein 2 [Source:HGNC Symbol;Acc:HGNC:11960]	HGLibA_19068;HGLibA_19067;HGLibA_19066
ENSG00000147133	TAF1	2	-3.025	-3.6694	0.026975	0.83016	0.00024315	0.051371	TATA-box binding protein associated factor 1 [Source:HGNC Symbol;Acc:HGNC:11535]	HGLibA_48194;HGLibA_48196
ENSG00000165338	HECTD2	3	-2.2544	-0.79248	0.046867	0.85738	0.42808	0.95326	HECT domain E3 ubiquitin protein ligase 2 [Source:HGNC Symbol;Acc:HGNC:26736]	HGLibA_21097;HGLibA_21096;HGLibA_21095
ENSG00000198743	SLC5A3	3	-2.8478	-3.9712	0.031157	0.83236	7.15E-05	0.020209	solute carrier family 5 member 3 [Source:HGNC Symbol;Acc:HGNC:11038]	HGLibA_45268;HGLibA_45269;HGLibA_45267
ENSG00000229833	PET100	1	-2.7615	-2.1127	0.033362	0.83236	0.034628	0.95326	PET100 homolog [Source:HGNC Symbol;Acc:HGNC:40038]	HGLibA_36069
ENSG00000171443	ZNF524	2	-3.4213	-3.7286	0.016773	0.82103	0.00019255	0.043648	zinc finger protein 524 [Source:HGNC Symbol;Acc:HGNC:28322]	HGLibA_56161;HGLibA_56162
ENSG00000106868	SUSD1	3	-4.5083	-6.0453	0.0017003	0.78846	1.49E-09	8.45E-06	sushi domain containing 1 [Source:HGNC Symbol;Acc:HGNC:25413]	HGLibA_47843;HGLibA_47845;HGLibA_47844
ENSG00000186141	POLR3C	2	-2.9753	-2.2589	0.027894	0.83016	0.023888	0.93831	RNA polymerase III subunit C [Source:HGNC Symbol;Acc:HGNC:30076]	HGLibA_37614;HGLibA_37615;HGLibA_37616
ENSG00000090263	MRPS33	3	-3.7818	-3.2062	0.0095124	0.81716	0.0013452	0.16823	mitochondrial ribosomal protein S33 [Source:HGNC Symbol;Acc:HGNC:16634]	HGLibA_30091;HGLibA_30093;HGLibA_30092
ENSG00000001631	KRIT1	3	-2.2615	-0.84412	0.046735	0.85738	0.3986	0.95326	ankyrin repeat containing [Source:HGNC Symbol;Acc:HGNC:1573]"	HGLibA_25407;HGLibA_25409;HGLibA_25408
ENSG00000130787	HIP1R	2	-3.7258	-3.0569	0.010386	0.82103	0.002236	0.24089	huntingtin interacting protein 1 related [Source:HGNC Symbol;Acc:HGNC:18415]	HGLibA_21335;HGLibA_21337
C8orf59	C8orf59	3	-3.8406	-3.3269	0.0086393	0.81716	0.00087814	0.12825	-	HGLibA_06640;HGLibA_06642;HGLibA_06641
ENSG00000128346	C22orf23	3	-2.1761	-0.76651	0.049262	0.86375	0.44337	0.95326	chromosome 22 open reading frame 23 [Source:HGNC Symbol;Acc:HGNC:18589]	HGLibA_06057;HGLibA_06055;HGLibA_06056
ENSG00000170298	LGALS9B	2	-3.1352	-0.85548	0.02385	0.83016	0.39229	0.95326	galectin 9B [Source:HGNC Symbol;Acc:HGNC:24842]	HGLibA_26383;HGLibA_26385
hsa-mir-4775	hsa-mir-4775	2	-3.1757	-3.1504	0.022563	0.83016	0.0016307	0.19181	-	HGLibA_61205;HGLibA_61204
ENSG00000137337	MDC1	2	-3.7384	-2.2134	0.010064	0.81716	0.02687	0.95326	mediator of DNA damage checkpoint 1 [Source:HGNC Symbol;Acc:HGNC:21163]	HGLibA_28666;HGLibA_28668
ENSG00000137842	TMEM62	2	-3.8134	-4.8156	0.0090988	0.81716	1.47E-06	0.0010303	transmembrane protein 62 [Source:HGNC Symbol;Acc:HGNC:26269]	HGLibA_50472;HGLibA_50471
ENSG00000101189	MRGBP	2	-2.3718	-3.2171	0.043013	0.84121	0.001295	0.16457	MRG domain binding protein [Source:HGNC Symbol;Acc:HGNC:15866]	HGLibA_29835;HGLibA_29834
ENSG00000151883	PARP8	2	-3.0976	-0.9873	0.025229	0.83016	0.32349	0.95326	poly(ADP-ribose) polymerase family member 8 [Source:HGNC Symbol;Acc:HGNC:26124]	HGLibA_35244;HGLibA_35245
ENSG00000161980	POLR3K	3	-3.1598	-5.0335	0.023299	0.83016	4.82E-07	0.00043665	RNA polymerase III subunit K [Source:HGNC Symbol;Acc:HGNC:14121]	HGLibA_37635;HGLibA_37636;HGLibA_37637
ENSG00000062822	POLD1	2	-2.7478	-3.6086	0.033868	0.83236	0.00030786	0.058256	catalytic subunit [Source:HGNC Symbol;Acc:HGNC:9175]"	HGLibA_37493;HGLibA_37492
ENSG00000162734	PEA15	1	-3.4761	-2.571	0.015854	0.82103	0.01014	0.58686	proliferation and apoptosis adaptor protein 15 [Source:HGNC Symbol;Acc:HGNC:8822]	HGLibA_36002
ENSG00000103811	CTSH	3	-3.1169	-1.4963	0.024815	0.83016	0.13457	0.95326	cathepsin H [Source:HGNC Symbol;Acc:HGNC:2535]	HGLibA_11709;HGLibA_11711;HGLibA_11710
ENSG00000156239	N6AMT1	1	-3.7133	-2.728	0.010477	0.82103	0.0063711	0.45014	N-6 adenine-specific DNA methyltransferase 1 [Source:HGNC Symbol;Acc:HGNC:16021]	HGLibA_30965
ENSG00000148396	SEC16A	3	-2.2107	-2.2133	0.047792	0.8595	0.026878	0.95326	endoplasmic reticulum export factor [Source:HGNC Symbol;Acc:HGNC:29006]"	HGLibA_43209;HGLibA_43207;HGLibA_43208
ENSG00000109536	FRG1	3	-2.4725	-0.64614	0.040256	0.83524	0.51819	0.95326	FSHD region gene 1 [Source:HGNC Symbol;Acc:HGNC:3954]	HGLibA_17994;HGLibA_17992;HGLibA_17993
ENSG00000120370	GORAB	1	-2.3592	-1.8672	0.043288	0.84121	0.061879	0.95326	RAB6 interacting [Source:HGNC Symbol;Acc:HGNC:25676]"	HGLibA_19674
ENSG00000159307	SCUBE1	1	-4.0037	-2.9229	0.0062497	0.81716	0.0034677	0.31369	CUB domain and EGF like domain containing 1 [Source:HGNC Symbol;Acc:HGNC:13441]"	HGLibA_43069
ENSG00000143420	ENSA	3	-3.6162	-3.7051	0.013026	0.82103	0.00021133	0.046234	endosulfine alpha [Source:HGNC Symbol;Acc:HGNC:3360]	HGLibA_15189;HGLibA_15188;HGLibA_15187
ENSG00000100823	APEX1	3	-4.1746	-4.9985	0.0042737	0.81716	5.78E-07	0.00048353	apurinic/apyrimidinic endodeoxyribonuclease 1 [Source:HGNC Symbol;Acc:HGNC:587]	HGLibA_02408;HGLibA_02406;HGLibA_02407
ENSG00000151304	SRFBP1	2	-3.2456	-2.8569	0.020541	0.83016	0.0042779	0.35763	serum response factor binding protein 1 [Source:HGNC Symbol;Acc:HGNC:26333]	HGLibA_46963;HGLibA_46965
ENSG00000176915	ANKLE2	3	-2.2494	-2.4947	0.046873	0.85738	0.012606	0.66274	ankyrin repeat and LEM domain containing 2 [Source:HGNC Symbol;Acc:HGNC:29101]	HGLibA_01975;HGLibA_01977;HGLibA_01976
ENSG00000176919	C8G	3	-3.3206	-2.4838	0.018887	0.82766	0.012998	0.67521	complement C8 gamma chain [Source:HGNC Symbol;Acc:HGNC:1354]	HGLibA_06600;HGLibA_06598;HGLibA_06599
ENSG00000113231	PDE8B	2	-2.6368	-0.64068	0.036212	0.83236	0.52173	0.95326	phosphodiesterase 8B [Source:HGNC Symbol;Acc:HGNC:8794]	HGLibA_35833;HGLibA_35831
ENSG00000187918	OR51I2	2	-3.8293	-3.8865	0.008915	0.81716	0.00010171	0.026667	olfactory receptor family 51 subfamily I member 2 [Source:HGNC Symbol;Acc:HGNC:15201]	HGLibA_34117;HGLibA_34118
hsa-mir-412	hsa-mir-412	3	-2.1532	-2.6499	0.049676	0.86375	0.0080521	0.52778	-	HGLibA_59686;HGLibA_59688;HGLibA_59689
ENSG00000136888	ATP6V1G1	1	-3.6702	-2.6994	0.011397	0.82103	0.0069475	0.47843	ATPase H+ transporting V1 subunit G1 [Source:HGNC Symbol;Acc:HGNC:864]	HGLibA_03758
ENSG00000126461	SCAF1	2	-3.7824	-2.8221	0.0095124	0.81716	0.0047706	0.38413	SR-related CTD associated factor 1 [Source:HGNC Symbol;Acc:HGNC:30403]	HGLibA_42822;HGLibA_42821
ENSG00000133107	TRPC4	3	-3.0018	-2.4694	0.02748	0.83016	0.013535	0.68498	transient receptor potential cation channel subfamily C member 4 [Source:HGNC Symbol;Acc:HGNC:12336]	HGLibA_51735;HGLibA_51733;HGLibA_51734
ENSG00000137868	STRA6	3	-2.7864	-2.127	0.032857	0.83236	0.033422	0.95326	stimulated by retinoic acid 6 [Source:HGNC Symbol;Acc:HGNC:30650]	HGLibA_47568;HGLibA_47566;HGLibA_47567
ENSG00000138592	USP8	2	-2.2371	-2.802	0.04724	0.85738	0.0050789	0.39472	ubiquitin specific peptidase 8 [Source:HGNC Symbol;Acc:HGNC:12631]	HGLibA_53414;HGLibA_53413
ENSG00000180834	MAP6D1	3	-3.0059	-1.3554	0.027251	0.83016	0.17529	0.95326	MAP6 domain containing 1 [Source:HGNC Symbol;Acc:HGNC:25753]	HGLibA_28229;HGLibA_28228;HGLibA_28230
ENSG00000159111	MRPL10	1	-3.6251	-2.6694	0.012683	0.82103	0.0075982	0.50564	mitochondrial ribosomal protein L10 [Source:HGNC Symbol;Acc:HGNC:14055]	HGLibA_29892
ENSG00000028310	BRD9	3	-2.5314	-2.0168	0.038371	0.83236	0.043721	0.95326	bromodomain containing 9 [Source:HGNC Symbol;Acc:HGNC:25818]	HGLibA_04789;HGLibA_04788;HGLibA_04787
hsa-mir-4703	hsa-mir-4703	1	-2.1783	-1.7604	0.048849	0.86282	0.078347	0.95326	-	HGLibA_60915
ENSG00000120054	CPN1	3	-3.0906	-0.58384	0.025275	0.83016	0.55933	0.95326	carboxypeptidase N subunit 1 [Source:HGNC Symbol;Acc:HGNC:2312]	HGLibA_10948;HGLibA_10947;HGLibA_10946
ENSG00000165125	TRPV6	3	-3.1693	-1.2779	0.022701	0.83016	0.20129	0.95326	transient receptor potential cation channel subfamily V member 6 [Source:HGNC Symbol;Acc:HGNC:14006]	HGLibA_51796;HGLibA_51798;HGLibA_51797
ENSG00000164815	ORC5	1	-3.421	-2.5348	0.016773	0.82103	0.01125	0.62292	origin recognition complex subunit 5 [Source:HGNC Symbol;Acc:HGNC:8491]	HGLibA_34612
ENSG00000111247	RAD51AP1	2	-2.4127	-2.5051	0.042048	0.84121	0.012243	0.65298	RAD51 associated protein 1 [Source:HGNC Symbol;Acc:HGNC:16956]	HGLibA_40082;HGLibA_40080
ENSG00000059377	TBXAS1	2	-2.6233	-2.8094	0.036533	0.83236	0.004964	0.39065	thromboxane A synthase 1 [Source:HGNC Symbol;Acc:HGNC:11609]	HGLibA_48684;HGLibA_48683
ENSG00000122862	SRGN	2	-3.4369	-1.7963	0.016543	0.82103	0.072444	0.95326	serglycin [Source:HGNC Symbol;Acc:HGNC:9361]	HGLibA_46986;HGLibA_46984
ENSG00000244617	ASPRV1	3	-2.1502	-2.589	0.049676	0.86375	0.0096266	0.58121	aspartic peptidase retroviral like 1 [Source:HGNC Symbol;Acc:HGNC:26321]	HGLibA_03362;HGLibA_03363;HGLibA_03361
ENSG00000182149	IST1	2	-2.3209	-2.2685	0.044529	0.85175	0.023299	0.92867	ESCRT-III associated factor [Source:HGNC Symbol;Acc:HGNC:28977]"	HGLibA_23687;HGLibA_23689
ENSG00000168813	ZNF507	2	-2.7925	-1.2787	0.032811	0.83236	0.201	0.95326	zinc finger protein 507 [Source:HGNC Symbol;Acc:HGNC:23783]	HGLibA_56123;HGLibA_56121
ENSG00000168906	MAT2A	2	-2.9451	-2.3217	0.028583	0.83201	0.020249	0.86275	methionine adenosyltransferase 2A [Source:HGNC Symbol;Acc:HGNC:6904]	HGLibA_28417;HGLibA_28419;HGLibA_28418
ENSG00000204316	MRPL38	3	-2.7615	-1.1581	0.033592	0.83236	0.24682	0.95326	mitochondrial ribosomal protein L38 [Source:HGNC Symbol;Acc:HGNC:14033]	HGLibA_29970;HGLibA_29968;HGLibA_29969
ENSG00000113838	TBCCD1	2	-3.1784	-3.7729	0.022471	0.83016	0.00016134	0.038582	TBCC domain containing 1 [Source:HGNC Symbol;Acc:HGNC:25546]	HGLibA_48587;HGLibA_48586
ENSG00000167105	TMEM92	3	-2.3481	-1.4132	0.043748	0.84738	0.1576	0.95326	transmembrane protein 92 [Source:HGNC Symbol;Acc:HGNC:26579]	HGLibA_50562;HGLibA_50561;HGLibA_50560
hsa-mir-548ac	hsa-mir-548ac	4	-2.7303	-4.3573	0.034006	0.83236	1.32E-05	0.0051168	-	HGLibA_61861;HGLibA_61860;HGLibA_61862;HGLibA_61859
ENSG00000183793	NPIPA5	3	-2.2387	-1.5348	0.04724	0.85738	0.12482	0.95326	nuclear pore complex interacting protein family member A5 [Source:HGNC Symbol;Acc:HGNC:41980]	HGLibA_32472;HGLibA_32471;HGLibA_32473
ENSG00000177119	ANO6	3	-2.5956	-0.57062	0.036855	0.83236	0.56826	0.95326	anoctamin 6 [Source:HGNC Symbol;Acc:HGNC:25240]	HGLibA_02187;HGLibA_02185;HGLibA_02186
ENSG00000172324	OR5A2	2	-2.7606	-0.63625	0.033638	0.83236	0.52461	0.95326	olfactory receptor family 5 subfamily A member 2 [Source:HGNC Symbol;Acc:HGNC:15249]	HGLibA_34231;HGLibA_34233
ENSG00000183891	TTC32	3	-3.7771	-3.3535	0.0095584	0.81716	0.0007979	0.11975	tetratricopeptide repeat domain 32 [Source:HGNC Symbol;Acc:HGNC:32954]	HGLibA_52115;HGLibA_52114;HGLibA_52113
ENSG00000171634	BPTF	2	-3.5311	-3.9735	0.014797	0.82103	7.08E-05	0.020209	bromodomain PHD finger transcription factor [Source:HGNC Symbol;Acc:HGNC:3581]	HGLibA_04741;HGLibA_04740
ENSG00000107862	GBF1	2	-2.2447	-2.3235	0.047011	0.85738	0.020154	0.86275	golgi brefeldin A resistant guanine nucleotide exchange factor 1 [Source:HGNC Symbol;Acc:HGNC:4181]	HGLibA_18723;HGLibA_18722
ENSG00000132704	FCRL2	1	-3.9045	-2.8561	0.0078121	0.81716	0.0042894	0.35763	Fc receptor like 2 [Source:HGNC Symbol;Acc:HGNC:14875]	HGLibA_17228
ENSG00000179271	GADD45GIP1	2	-3.0943	-1.5224	0.025229	0.83016	0.1279	0.95326	GADD45G interacting protein 1 [Source:HGNC Symbol;Acc:HGNC:29996]	HGLibA_18424;HGLibA_18426
ENSG00000062582	MRPS24	2	-2.1877	-2.6173	0.048389	0.8617	0.0088628	0.55262	mitochondrial ribosomal protein S24 [Source:HGNC Symbol;Acc:HGNC:14510]	HGLibA_30072;HGLibA_30070
ENSG00000148136	OR13C4	3	-2.5888	-0.81904	0.036947	0.83236	0.41276	0.95326	olfactory receptor family 13 subfamily C member 4 [Source:HGNC Symbol;Acc:HGNC:14722]	HGLibA_33602;HGLibA_33601;HGLibA_33603
ENSG00000138411	HECW2	1	-2.2194	-1.7712	0.047654	0.85844	0.07653	0.95326	C2 and WW domain containing E3 ubiquitin protein ligase 2 [Source:HGNC Symbol;Acc:HGNC:29853]"	HGLibA_21105
ENSG00000091483	FH	3	-3.1712	-4.774	0.022701	0.83016	1.81E-06	0.0012282	fumarate hydratase [Source:HGNC Symbol;Acc:HGNC:3700]	HGLibA_17472;HGLibA_17470;HGLibA_17471
ENSG00000253598	SLC10A5	1	-4.1222	-3.0031	0.0047792	0.81716	0.0026722	0.27046	solute carrier family 10 member 5 [Source:HGNC Symbol;Acc:HGNC:22981]	HGLibA_44341
ENSG00000039650	PNKP	2	-3.123	-4.028	0.024631	0.83016	5.63E-05	0.016771	polynucleotide kinase 3'-phosphatase [Source:HGNC Symbol;Acc:HGNC:9154]	HGLibA_37352;HGLibA_37351
ENSG00000203963	C1orf141	3	-2.933	-4.4678	0.028721	0.83201	7.90E-06	0.0033726	chromosome 1 open reading frame 141 [Source:HGNC Symbol;Acc:HGNC:32044]	HGLibA_05771;HGLibA_05772;HGLibA_05770
ENSG00000138107	ACTR1A	2	-2.7606	-3.2235	0.033638	0.83236	0.0012663	0.16457	ARP1 actin related protein 1 homolog A [Source:HGNC Symbol;Acc:HGNC:167]	HGLibA_00649;HGLibA_00650
ENSG00000126934	MAP2K2	3	-2.5703	-2.5723	0.037636	0.83236	0.010101	0.58686	mitogen-activated protein kinase kinase 2 [Source:HGNC Symbol;Acc:HGNC:6842]	HGLibA_28141;HGLibA_28143;HGLibA_28142
ENSG00000130449	ZSWIM6	2	-2.3184	-0.98531	0.044575	0.85175	0.32447	0.95326	zinc finger SWIM-type containing 6 [Source:HGNC Symbol;Acc:HGNC:29316]	HGLibA_57052;HGLibA_57053
ENSG00000168389	MFSD2A	2	-2.5844	-2.9215	0.037085	0.83236	0.0034835	0.31369	major facilitator superfamily domain containing 2A [Source:HGNC Symbol;Acc:HGNC:25897]	HGLibA_29094;HGLibA_29093
ENSG00000132485	ZRANB2	2	-2.6726	-2.7379	0.035201	0.83236	0.006184	0.44412	zinc finger RANBP2-type containing 2 [Source:HGNC Symbol;Acc:HGNC:13058]	HGLibA_56975;HGLibA_56974
ENSG00000084463	WBP11	3	-3.1456	-4.6626	0.023528	0.83016	3.12E-06	0.0019412	WW domain binding protein 11 [Source:HGNC Symbol;Acc:HGNC:16461]	HGLibA_53971;HGLibA_53970;HGLibA_53972
ENSG00000103512	NOMO1	3	-2.1516	-2.7747	0.049676	0.86375	0.0055249	0.41745	NODAL modulator 1 [Source:HGNC Symbol;Acc:HGNC:30060]	HGLibA_32288;HGLibA_32289;HGLibA_32290
ENSG00000129910	CDH15	3	-2.91	-3.5359	0.029548	0.83236	0.00040638	0.070746	cadherin 15 [Source:HGNC Symbol;Acc:HGNC:1754]	HGLibA_08644;HGLibA_08643;HGLibA_08645
ENSG00000128059	PPAT	2	-3.4847	-1.7517	0.015762	0.82103	0.079822	0.95326	phosphoribosyl pyrophosphate amidotransferase [Source:HGNC Symbol;Acc:HGNC:9238]	HGLibA_37852;HGLibA_37851
ENSG00000150991	UBC	3	-2.29	-2.5191	0.04531	0.85666	0.011765	0.64019	ubiquitin C [Source:HGNC Symbol;Acc:HGNC:12468]	HGLibA_52566;HGLibA_52567;HGLibA_52568
ENSG00000164002	EXO5	2	-2.3206	-1.4041	0.044529	0.85175	0.16029	0.95326	exonuclease 5 [Source:HGNC Symbol;Acc:HGNC:26115]	HGLibA_15728;HGLibA_15727
ENSG00000169340	PDILT	2	-3.9905	-4.7475	0.0066173	0.81716	2.06E-06	0.0013581	testis expressed [Source:HGNC Symbol;Acc:HGNC:27338]"	HGLibA_35891;HGLibA_35893
ENSG00000105072	C19orf44	3	-2.192	-2.7386	0.048251	0.85995	0.0061708	0.44412	chromosome 19 open reading frame 44 [Source:HGNC Symbol;Acc:HGNC:26141]	HGLibA_05662;HGLibA_05664;HGLibA_05663
SPDYE2L	SPDYE2L	3	-2.2283	-1.4735	0.04747	0.85797	0.14063	0.95326	-	HGLibA_46587;HGLibA_46589;HGLibA_46588
ENSG00000218823	PAPOLB	2	-2.2073	-1.399	0.047884	0.85995	0.1618	0.95326	poly(A) polymerase beta [Source:HGNC Symbol;Acc:HGNC:15970]	HGLibA_35139;HGLibA_35140
ENSG00000173085	COQ2	2	-2.327	-2.7537	0.044299	0.85159	0.0058925	0.43434	polyprenyltransferase [Source:HGNC Symbol;Acc:HGNC:25223]"	HGLibA_10750;HGLibA_10749
ENSG00000127463	EMC1	2	-3.2291	-3.0655	0.021231	0.83016	0.0021731	0.23763	ER membrane protein complex subunit 1 [Source:HGNC Symbol;Acc:HGNC:28957]	HGLibA_15002;HGLibA_15001
ENSG00000170214	ADRA1B	2	-2.8601	-0.34689	0.030743	0.83236	0.72867	0.95622	adrenoceptor alpha 1B [Source:HGNC Symbol;Acc:HGNC:278]	HGLibA_01039;HGLibA_01040
ENSG00000150672	DLG2	1	-2.4386	-1.9148	0.040945	0.83741	0.055519	0.95326	discs large MAGUK scaffold protein 2 [Source:HGNC Symbol;Acc:HGNC:2901]	HGLibA_13328
ENSG00000111432	FZD10	3	-2.4875	-2.2419	0.039934	0.83524	0.02497	0.95326	frizzled class receptor 10 [Source:HGNC Symbol;Acc:HGNC:4039]	HGLibA_18262;HGLibA_18263;HGLibA_18264
ENSG00000132600	PRMT7	3	-2.8907	-3.0378	0.029962	0.83236	0.0023834	0.253	protein arginine methyltransferase 7 [Source:HGNC Symbol;Acc:HGNC:25557]	HGLibA_38636;HGLibA_38634;HGLibA_38635
hsa-mir-4679-2	hsa-mir-4679-2	1	-2.8152	-2.1462	0.031984	0.83236	0.031859	0.95326	-	HGLibA_60832
ENSG00000170788	DYDC1	1	-3.9554	-2.8903	0.0070769	0.81716	0.0038489	0.33435	DPY30 domain containing 1 [Source:HGNC Symbol;Acc:HGNC:23460]	HGLibA_14219
ENSG00000203757	OR6K3	3	-2.1474	-2.9365	0.049768	0.86375	0.003319	0.30813	olfactory receptor family 6 subfamily K member 3 [Source:HGNC Symbol;Acc:HGNC:15030]	HGLibA_34421;HGLibA_34420;HGLibA_34422
ENSG00000182902	SLC25A18	3	-2.8988	-0.82193	0.029824	0.83236	0.41112	0.95326	solute carrier family 25 member 18 [Source:HGNC Symbol;Acc:HGNC:10988]	HGLibA_44661;HGLibA_44659;HGLibA_44660
ENSG00000163882	POLR2H	2	-2.8458	-2.7494	0.031203	0.83236	0.0059713	0.43604	RNA polymerase II subunit H [Source:HGNC Symbol;Acc:HGNC:9195]	HGLibA_37584;HGLibA_37586
ENSG00000225921	NOL7	1	-2.5806	-2.001	0.037131	0.83236	0.045392	0.95326	nucleolar protein 7 [Source:HGNC Symbol;Acc:HGNC:21040]	HGLibA_32273
ENSG00000157152	SYN2	2	-4.007	-4.8448	0.0062497	0.81716	1.27E-06	0.00096856	synapsin II [Source:HGNC Symbol;Acc:HGNC:11495]	HGLibA_47957;HGLibA_47959
ENSG00000114857	NKTR	1	-4.1361	-3.0125	0.0045954	0.81716	0.0025907	0.26593	natural killer cell triggering receptor [Source:HGNC Symbol;Acc:HGNC:7833]	HGLibA_32025
ENSG00000168827	GFM1	2	-2.7118	-2.0233	0.03419	0.83236	0.043038	0.95326	G elongation factor mitochondrial 1 [Source:HGNC Symbol;Acc:HGNC:13780]	HGLibA_18948;HGLibA_18946
ENSG00000168152	THAP9	2	-3.5071	-4.4539	0.015349	0.82103	8.43E-06	0.0035293	THAP domain containing 9 [Source:HGNC Symbol;Acc:HGNC:23192]	HGLibA_49315;HGLibA_49317
ENSG00000204370	SDHD	2	-3.4907	-3.7406	0.01567	0.82103	0.0001836	0.042056	succinate dehydrogenase complex subunit D [Source:HGNC Symbol;Acc:HGNC:10683]	HGLibA_43149;HGLibA_43148
hsa-mir-140	hsa-mir-140	3	-2.5085	-2.992	0.039428	0.83398	0.0027716	0.2754	-	HGLibA_57824;HGLibA_57823;HGLibA_57825
ENSG00000155561	NUP205	2	-3.659	-1.3417	0.011902	0.82103	0.17969	0.95326	nucleoporin 205 [Source:HGNC Symbol;Acc:HGNC:18658]	HGLibA_33036;HGLibA_33035
ENSG00000205022	PABPN1L	2	-3.2953	-2.7677	0.01953	0.83016	0.0056458	0.42365	cytoplasmic [Source:HGNC Symbol;Acc:HGNC:37237]"	HGLibA_34946;HGLibA_34944
ENSG00000082781	ITGB5	1	-4.4812	-3.2483	0.0017462	0.78846	0.0011608	0.15593	integrin subunit beta 5 [Source:HGNC Symbol;Acc:HGNC:6160]	HGLibA_23789
ENSG00000125630	POLR1B	3	-3.1108	-1.8375	0.024953	0.83016	0.066129	0.95326	RNA polymerase I subunit B [Source:HGNC Symbol;Acc:HGNC:20454]	HGLibA_37551;HGLibA_37553;HGLibA_37552
ENSG00000172086	KRCC1	2	-2.2283	-1.0519	0.047424	0.85786	0.29284	0.95326	lysine rich coiled-coil 1 [Source:HGNC Symbol;Acc:HGNC:28039]	HGLibA_25399;HGLibA_25398
ENSG00000197822	OCLN	2	-4.5551	-5.5218	0.0014705	0.78846	3.36E-08	6.64E-05	occludin [Source:HGNC Symbol;Acc:HGNC:8104]	HGLibA_33253;HGLibA_33255
ENSG00000111011	RSRC2	2	-2.8447	-3.0277	0.031249	0.83236	0.0024644	0.25907	arginine and serine rich coiled-coil 2 [Source:HGNC Symbol;Acc:HGNC:30559]	HGLibA_42363;HGLibA_42361
ENSG00000162923	WDR26	3	-2.8119	-3.6003	0.032443	0.83236	0.00031788	0.059633	WD repeat domain 26 [Source:HGNC Symbol;Acc:HGNC:21208]	HGLibA_54055;HGLibA_54054;HGLibA_54056
ENSG00000173653	RCE1	3	-2.5699	-0.72651	0.037636	0.83236	0.46753	0.95326	Ras converting CAAX endopeptidase 1 [Source:HGNC Symbol;Acc:HGNC:13721]	HGLibA_40691;HGLibA_40693;HGLibA_40692
ENSG00000100347	SAMM50	2	-3.2094	-2.5175	0.021598	0.83016	0.01182	0.64019	SAMM50 sorting and assembly machinery component [Source:HGNC Symbol;Acc:HGNC:24276]	HGLibA_42706;HGLibA_42707
ENSG00000086548	CEACAM6	1	-3.887	-2.8443	0.00795	0.81716	0.0044514	0.36692	carcinoembryonic antigen related cell adhesion molecule 6 [Source:HGNC Symbol;Acc:HGNC:1818]	HGLibA_08948
ENSG00000175166	PSMD2	1	-3.7943	-2.7821	0.0092367	0.81716	0.0054008	0.4095	non-ATPase 2 [Source:HGNC Symbol;Acc:HGNC:9559]"	HGLibA_39165
ENSG00000155858	LSM11	2	-3.5714	-4.5417	0.013924	0.82103	5.58E-06	0.0025843	U7 small nuclear RNA associated [Source:HGNC Symbol;Acc:HGNC:30860]"	HGLibA_27524;HGLibA_27523
ENSG00000160563	MED27	1	-2.3592	-1.8672	0.043288	0.84121	0.061879	0.95326	mediator complex subunit 27 [Source:HGNC Symbol;Acc:HGNC:2377]	HGLibA_28794
hsa-mir-4679-1	hsa-mir-4679-1	1	-2.4386	-1.9148	0.040945	0.83741	0.055519	0.95326	-	HGLibA_60831
ENSG00000146151	HMGCLL1	3	-2.1426	-0.79594	0.049814	0.86375	0.42606	0.95326	3-hydroxymethyl-3-methylglutaryl-CoA lyase like 1 [Source:HGNC Symbol;Acc:HGNC:21359]	HGLibA_21706;HGLibA_21704;HGLibA_21705
ENSG00000149313	AASDHPPT	2	-3.0132	-1.1684	0.027113	0.83016	0.24264	0.95326	aminoadipate-semialdehyde dehydrogenase-phosphopantetheinyl transferase [Source:HGNC Symbol;Acc:HGNC:14235]	HGLibA_00079;HGLibA_00080
SPHAR	SPHAR	1	-2.5122	-1.9593	0.038969	0.83236	0.050081	0.95326	-	HGLibA_46641
ENSG00000258366	RTEL1	3	-3.4703	-2.3207	0.016176	0.82103	0.020301	0.86275	regulator of telomere elongation helicase 1 [Source:HGNC Symbol;Acc:HGNC:15888]	HGLibA_42380;HGLibA_42379;HGLibA_42381
ENSG00000169495	HTRA4	2	-4.1194	-4.6108	0.0048711	0.81716	4.01E-06	0.0022714	HtrA serine peptidase 4 [Source:HGNC Symbol;Acc:HGNC:26909]	HGLibA_22415;HGLibA_22417
ENSG00000168538	TRAPPC11	2	-3.1611	-1.4785	0.023207	0.83016	0.13928	0.95326	trafficking protein particle complex 11 [Source:HGNC Symbol;Acc:HGNC:25751]	HGLibA_51304;HGLibA_51302
ENSG00000255529	POLR2M	3	-3.9358	-6.1126	0.0073986	0.81716	9.80E-10	8.45E-06	RNA polymerase II subunit M [Source:HGNC Symbol;Acc:HGNC:14862]	HGLibA_37606;HGLibA_37605;HGLibA_37607
ENSG00000205765	C5orf51	3	-2.8007	-0.64069	0.032627	0.83236	0.52173	0.95326	chromosome 5 open reading frame 51 [Source:HGNC Symbol;Acc:HGNC:27750]	HGLibA_06401;HGLibA_06402;HGLibA_06400
ENSG00000105499	PLA2G4C	3	-2.6839	-4.5714	0.035063	0.83236	4.85E-06	0.0022949	phospholipase A2 group IVC [Source:HGNC Symbol;Acc:HGNC:9037]	HGLibA_36905;HGLibA_36907;HGLibA_36906
ENSG00000173145	NOC3L	1	-2.5806	-2.001	0.037131	0.83236	0.045392	0.95326	NOC3 like DNA replication regulator [Source:HGNC Symbol;Acc:HGNC:24034]	HGLibA_32237
ENSG00000221944	TIGD1	3	-2.4603	-2.9617	0.040301	0.83524	0.0030594	0.29328	tigger transposable element derived 1 [Source:HGNC Symbol;Acc:HGNC:14523]	HGLibA_49463;HGLibA_49462;HGLibA_49464
HIST1H2AG	HIST1H2AG	3	-2.2409	-2.675	0.047103	0.85738	0.0074738	0.50352	-	HGLibA_21389;HGLibA_21390;HGLibA_21391
ENSG00000138115	CYP2C8	2	-2.4194	-1.256	0.04191	0.84121	0.20913	0.95326	cytochrome P450 family 2 subfamily C member 8 [Source:HGNC Symbol;Acc:HGNC:2622]	HGLibA_12120;HGLibA_12122
ENSG00000138794	CASP6	3	-3.1146	-2.4151	0.024815	0.83016	0.01573	0.76097	caspase 6 [Source:HGNC Symbol;Acc:HGNC:1507]	HGLibA_07319;HGLibA_07320;HGLibA_07318
ENSG00000186446	ZNF501	3	-2.9019	-0.58171	0.029824	0.83236	0.56076	0.95326	zinc finger protein 501 [Source:HGNC Symbol;Acc:HGNC:23717]	HGLibA_56111;HGLibA_56109;HGLibA_56110
ENSG00000168924	LETM1	3	-2.5457	-2.9211	0.038096	0.83236	0.0034885	0.31369	leucine zipper and EF-hand containing transmembrane protein 1 [Source:HGNC Symbol;Acc:HGNC:6556]	HGLibA_26332;HGLibA_26333;HGLibA_26334
ENSG00000100410	PHF5A	1	-2.3592	-1.8672	0.043288	0.84121	0.061879	0.95326	PHD finger protein 5A [Source:HGNC Symbol;Acc:HGNC:18000]	HGLibA_36381
ENSG00000102452	NALCN	3	-2.7646	-3.4213	0.033362	0.83236	0.00062327	0.099728	non-selective [Source:HGNC Symbol;Acc:HGNC:19082]"	HGLibA_31085;HGLibA_31083;HGLibA_31084
ENSG00000107937	GTPBP4	2	-3.5376	-1.7035	0.014751	0.82103	0.088471	0.95326	GTP binding protein 4 [Source:HGNC Symbol;Acc:HGNC:21535]	HGLibA_20611;HGLibA_20609
ENSG00000156990	RPUSD3	2	-3.4907	-3.7406	0.01567	0.82103	0.0001836	0.042056	RNA pseudouridine synthase D3 [Source:HGNC Symbol;Acc:HGNC:28437]	HGLibA_42212;HGLibA_42211
ENSG00000137804	NUSAP1	3	-3.1199	-1.1013	0.024723	0.83016	0.27076	0.95326	nucleolar and spindle associated protein 1 [Source:HGNC Symbol;Acc:HGNC:18538]	HGLibA_33097;HGLibA_33096;HGLibA_33095
ENSG00000134146	DPH6	1	-3.3322	-2.4768	0.018519	0.82766	0.013258	0.67724	diphthamine biosynthesis 6 [Source:HGNC Symbol;Acc:HGNC:30543]	HGLibA_13851
ENSG00000143742	SRP9	3	-3.6169	-3.2704	0.013005	0.82103	0.0010738	0.14884	signal recognition particle 9 [Source:HGNC Symbol;Acc:HGNC:11304]	HGLibA_47015;HGLibA_47014;HGLibA_47016
ENSG00000167272	POP5	1	-3.3322	-2.4768	0.018519	0.82766	0.013258	0.67724	ribonuclease P/MRP subunit [Source:HGNC Symbol;Acc:HGNC:17689]"	HGLibA_37693
ENSG00000204839	MROH6	3	-2.2371	-2.7548	0.04724	0.85738	0.0058733	0.43434	maestro heat like repeat family member 6 [Source:HGNC Symbol;Acc:HGNC:27814]	HGLibA_29872;HGLibA_29873;HGLibA_29874
ENSG00000133226	SRRM1	2	-2.8203	-1.5594	0.0318	0.83236	0.11891	0.95326	serine and arginine repetitive matrix 1 [Source:HGNC Symbol;Acc:HGNC:16638]	HGLibA_47045;HGLibA_47046
CCRN4L	CCRN4L	1	-2.5122	-1.9593	0.038969	0.83236	0.050081	0.95326	-	HGLibA_08180
ENSG00000115541	HSPE1	1	-2.7615	-2.1127	0.033362	0.83236	0.034628	0.95326	heat shock protein family E (Hsp10) member 1 [Source:HGNC Symbol;Acc:HGNC:5269]	HGLibA_22335
ENSG00000147697	GSDMC	2	-3.8655	-3.6211	0.0082717	0.81716	0.00029337	0.056	gasdermin C [Source:HGNC Symbol;Acc:HGNC:7151]	HGLibA_20410;HGLibA_20411
ENSG00000155761	SPAG17	1	-4.2405	-3.0836	0.0035384	0.81716	0.0020453	0.22942	sperm associated antigen 17 [Source:HGNC Symbol;Acc:HGNC:26620]	HGLibA_46353
hsa-mir-1279	hsa-mir-1279	2	-3.4163	-4.2195	0.017049	0.82119	2.45E-05	0.0084562	-	HGLibA_57569;HGLibA_57570
ENSG00000139515	PDX1	2	-2.8866	-0.84513	0.030008	0.83236	0.39804	0.95326	pancreatic and duodenal homeobox 1 [Source:HGNC Symbol;Acc:HGNC:6107]	HGLibA_35955;HGLibA_35954
ENSG00000179115	FARSA	1	-3.1637	-2.3676	0.022793	0.83016	0.017904	0.81508	phenylalanyl-tRNA synthetase subunit alpha [Source:HGNC Symbol;Acc:HGNC:3592]	HGLibA_16883

ENSG00000077549	CAPZB	2	-3.4295	-3.2761	0.016681	0.82103	0.0010525	0.14776	capping actin protein of muscle Z-line subunit beta [Source:HGNC Symbol;Acc:HGNC:1491]	HGLibA_07215;HGLibA_07214
ENSG00000138642	HERC6	2	-2.3961	-2.5321	0.042645	0.84121	0.011338	0.62459	HECT and RLD domain containing E3 ubiquitin protein ligase family member 6 [Source:HGNC Symbol;Acc:HGNC:26072]	HGLibA_21167;HGLibA_21169
ENSG00000230178	OR4F3	3	-3.3985	-4.5789	0.017095	0.82119	4.67E-06	0.0022949	olfactory receptor family 4 subfamily F member 3 [Source:HGNC Symbol;Acc:HGNC:8300]	HGLibA_34003;HGLibA_34004;HGLibA_34005
ENSG00000162961	DPY30	2	-2.3982	-2.7179	0.042553	0.84121	0.0065695	0.46116	histone methyltransferase complex regulatory subunit [Source:HGNC Symbol;Acc:HGNC:24590]"	HGLibA_13916;HGLibA_13917
ENSG00000125484	GTF3C4	2	-3.1355	-2.4252	0.023804	0.83016	0.015302	0.74997	general transcription factor IIIC subunit 4 [Source:HGNC Symbol;Acc:HGNC:4667]	HGLibA_20588;HGLibA_20589
ENSG00000173612	GPRC6A	2	-2.9386	-0.84759	0.028675	0.83201	0.39667	0.95326	G protein-coupled receptor class C group 6 member A [Source:HGNC Symbol;Acc:HGNC:18510]	HGLibA_20136;HGLibA_20138
HARS	HARS	3	-2.8118	-2.5135	0.032443	0.83236	0.011953	0.64222	-	HGLibA_20855;HGLibA_20857;HGLibA_20856
ENSG00000151779	NBAS	3	-3.6607	-2.5596	0.011902	0.82103	0.01048	0.6033	neuroblastoma amplified sequence [Source:HGNC Symbol;Acc:HGNC:15625]	HGLibA_31202;HGLibA_31203;HGLibA_31204
ENSG00000130940	CASZ1	3	-3.409	-4.0586	0.017095	0.82119	4.94E-05	0.015303	castor zinc finger 1 [Source:HGNC Symbol;Acc:HGNC:26002]	HGLibA_07349;HGLibA_07350;HGLibA_07348
ENSG00000162775	RBM15	2	-3.2632	-1.781	0.020312	0.83016	0.074913	0.95326	RNA binding motif protein 15 [Source:HGNC Symbol;Acc:HGNC:14959]	HGLibA_40492;HGLibA_40491
ENSG00000136122	BORA	2	-3.2123	-3.2551	0.021598	0.83016	0.0011334	0.15424	aurora kinase A activator [Source:HGNC Symbol;Acc:HGNC:24724]"	HGLibA_04699;HGLibA_04697
ENSG00000163795	ZNF513	1	-3.7943	-2.7821	0.0092367	0.81716	0.0054008	0.4095	zinc finger protein 513 [Source:HGNC Symbol;Acc:HGNC:26498]	HGLibA_56137
ENSG00000124380	SNRNP27	2	-3.0194	-3.7629	0.027021	0.83016	0.00016798	0.039305	small nuclear ribonucleoprotein U4/U6.U5 subunit 27 [Source:HGNC Symbol;Acc:HGNC:30240]	HGLibA_45947;HGLibA_45946
ENSG00000012048	BRCA1	3	-3.5487	-4.61	0.014659	0.82103	4.03E-06	0.0022714	DNA repair associated [Source:HGNC Symbol;Acc:HGNC:1100]"	HGLibA_04762;HGLibA_04760;HGLibA_04761
ENSG00000174547	MRPL11	3	-2.1515	-2.0587	0.049676	0.86375	0.039525	0.95326	mitochondrial ribosomal protein L11 [Source:HGNC Symbol;Acc:HGNC:14042]	HGLibA_29894;HGLibA_29895;HGLibA_29893
C17orf89	C17orf89	2	-2.7142	-2.5944	0.03419	0.83236	0.0094765	0.57764	-	HGLibA_05592;HGLibA_05591
ENSG00000134153	EMC7	3	-2.8473	-3.9071	0.031157	0.83236	9.34E-05	0.024787	ER membrane protein complex subunit 7 [Source:HGNC Symbol;Acc:HGNC:24301]	HGLibA_15019;HGLibA_15020;HGLibA_15021
ENSG00000128245	YWHAH	3	-3.2491	-4.5124	0.020449	0.83016	6.41E-06	0.0027903	tyrosine 3-monooxygenase/tryptophan 5-monooxygenase activation protein eta [Source:HGNC Symbol;Acc:HGNC:12853]	HGLibA_54748;HGLibA_54749;HGLibA_54747
ENSG00000244187	TMEM141	3	-2.1967	-2.5153	0.048068	0.85995	0.011893	0.64058	transmembrane protein 141 [Source:HGNC Symbol;Acc:HGNC:28211]	HGLibA_49964;HGLibA_49963;HGLibA_49965
ENSG00000141298	SSH2	3	-3.1831	-3.1142	0.022425	0.83016	0.0018444	0.21349	slingshot protein phosphatase 2 [Source:HGNC Symbol;Acc:HGNC:30580]	HGLibA_47134;HGLibA_47136;HGLibA_47135
ENSG00000166091	CMTM5	2	-2.6865	-2.8625	0.035063	0.83236	0.004203	0.3545	CKLF like MARVEL transmembrane domain containing 5 [Source:HGNC Symbol;Acc:HGNC:19176]	HGLibA_10220;HGLibA_10222
ENSG00000070756	PABPC1	3	-2.3928	-1.0031	0.042645	0.84121	0.31581	0.95326	poly(A) binding protein cytoplasmic 1 [Source:HGNC Symbol;Acc:HGNC:8554]	HGLibA_34921;HGLibA_34922;HGLibA_34920
CASC5	CASC5	2	-3.1675	-0.98095	0.022701	0.83016	0.32662	0.95326	-	HGLibA_07281;HGLibA_07280
ENSG00000161040	FBXL13	2	-3.4427	-4.0856	0.016543	0.82103	4.40E-05	0.013868	F-box and leucine rich repeat protein 13 [Source:HGNC Symbol;Acc:HGNC:21658]	HGLibA_16975;HGLibA_16976
ENSG00000158006	PAFAH2	3	-2.1528	-3.1422	0.049676	0.86375	0.0016769	0.19619	platelet activating factor acetylhydrolase 2 [Source:HGNC Symbol;Acc:HGNC:8579]	HGLibA_34998;HGLibA_34997;HGLibA_34999
ENSG00000256188	TAS2R30	2	-2.6256	-3.2421	0.036487	0.83236	0.0011864	0.15838	taste 2 receptor member 30 [Source:HGNC Symbol;Acc:HGNC:19112]	HGLibA_48386;HGLibA_48388
ENSG00000197283	SYNGAP1	3	-2.9549	-3.7215	0.028353	0.83201	0.00019806	0.044433	synaptic Ras GTPase activating protein 1 [Source:HGNC Symbol;Acc:HGNC:11497]	HGLibA_47988;HGLibA_47987;HGLibA_47989
ENSG00000107897	ACBD5	3	-2.6589	-3.2149	0.035384	0.83236	0.0013051	0.16457	acyl-CoA binding domain containing 5 [Source:HGNC Symbol;Acc:HGNC:23338]	HGLibA_00405;HGLibA_00404;HGLibA_00403
ENSG00000167550	RHEBL1	3	-2.5141	-1.8933	0.038877	0.83236	0.058318	0.95326	RHEB like 1 [Source:HGNC Symbol;Acc:HGNC:21166]	HGLibA_41158;HGLibA_41156;HGLibA_41157
ENSG00000091879	ANGPT2	2	-3.0136	-2.3932	0.027113	0.83016	0.0167	0.78733	angiopoietin 2 [Source:HGNC Symbol;Acc:HGNC:485]	HGLibA_01904;HGLibA_01903
ENSG00000091651	ORC6	1	-2.5806	-2.001	0.037131	0.83236	0.045392	0.95326	origin recognition complex subunit 6 [Source:HGNC Symbol;Acc:HGNC:17151]	HGLibA_34616
ENSG00000230301	OR5H6	3	-2.3734	-3.2327	0.043013	0.84121	0.0012261	0.16268	olfactory receptor family 5 subfamily H member 6 (gene/pseudogene) [Source:HGNC Symbol;Acc:HGNC:14767]	HGLibA_34302;HGLibA_34301;HGLibA_34300
ENSG00000173530	TNFRSF10D	1	-2.5122	-1.9593	0.038969	0.83236	0.050081	0.95326	TNF receptor superfamily member 10d [Source:HGNC Symbol;Acc:HGNC:11907]	HGLibA_50743
ENSG00000147130	ZMYM3	3	-2.1576	-2.6329	0.049538	0.86375	0.0084653	0.5418	zinc finger MYM-type containing 3 [Source:HGNC Symbol;Acc:HGNC:13054]	HGLibA_55396;HGLibA_55397;HGLibA_55395
ENSG00000167968	DNASE1L2	3	-2.9637	-0.55613	0.028032	0.83016	0.57812	0.95326	deoxyribonuclease 1 like 2 [Source:HGNC Symbol;Acc:HGNC:2958]	HGLibA_13660;HGLibA_13658;HGLibA_13659
ENSG00000143252	SDHC	3	-3.1442	-1.5613	0.023528	0.83016	0.11845	0.95326	succinate dehydrogenase complex subunit C [Source:HGNC Symbol;Acc:HGNC:10682]	HGLibA_43146;HGLibA_43145;HGLibA_43144
ENSG00000173113	TRMT112	2	-2.6991	-2.6839	0.034879	0.83236	0.0072768	0.49331	tRNA methyltransferase subunit 11-2 [Source:HGNC Symbol;Acc:HGNC:26940]	HGLibA_51673;HGLibA_51672
ENSG00000060566	CREB3L3	3	-3.4646	-1.2656	0.016176	0.82103	0.20567	0.95326	cAMP responsive element binding protein 3 like 3 [Source:HGNC Symbol;Acc:HGNC:18855]	HGLibA_11098;HGLibA_11099;HGLibA_11100
ENSG00000235098	ANKRD65	2	-2.3134	-2.3922	0.044621	0.85175	0.016749	0.78733	ankyrin repeat domain 65 [Source:HGNC Symbol;Acc:HGNC:42950]	HGLibA_02137;HGLibA_02139
ENSG00000135407	AVIL	2	-2.4097	-2.5733	0.042094	0.84121	0.010074	0.58686	advillin [Source:HGNC Symbol;Acc:HGNC:14188]	HGLibA_03890;HGLibA_03892
ENSG00000163923	RPL39L	1	-2.9606	-2.2378	0.028078	0.83016	0.025235	0.95326	ribosomal protein L39 like [Source:HGNC Symbol;Acc:HGNC:17094]	HGLibA_41975
ENSG00000163634	THOC7	2	-3.8505	-4.5964	0.0086393	0.81716	4.30E-06	0.002282	THO complex 7 [Source:HGNC Symbol;Acc:HGNC:29874]	HGLibA_49379;HGLibA_49378
ENSG00000176102	CSTF3	1	-3.0047	-2.2658	0.027251	0.83016	0.023464	0.92867	cleavage stimulation factor subunit 3 [Source:HGNC Symbol;Acc:HGNC:2485]	HGLibA_11493
hsa-mir-4801	hsa-mir-4801	2	-3.8145	-2.1117	0.008961	0.81716	0.034713	0.95326	-	HGLibA_61309;HGLibA_61308
ENSG00000132467	UTP3	3	-2.9919	-3.9443	0.027664	0.83016	8.00E-05	0.022045	small subunit processome component [Source:HGNC Symbol;Acc:HGNC:24477]"	HGLibA_53451;HGLibA_53452;HGLibA_53453
ENSG00000168297	PXK	2	-3.2139	-3.6451	0.021552	0.83016	0.00026732	0.054602	PX domain containing serine/threonine kinase like [Source:HGNC Symbol;Acc:HGNC:23326]	HGLibA_39655;HGLibA_39656
ENSG00000122406	RPL5	1	-2.8152	-2.1462	0.031984	0.83236	0.031859	0.95326	ribosomal protein L5 [Source:HGNC Symbol;Acc:HGNC:10360]	HGLibA_41988
hsa-mir-4328	hsa-mir-4328	3	-4.141	-5.6033	0.0045494	0.81716	2.10E-08	5.72E-05	-	HGLibA_60037;HGLibA_60034;HGLibA_60035
ENSG00000136718	IMP4	1	-3.0047	-2.2658	0.027251	0.83016	0.023464	0.92867	U3 small nucleolar ribonucleoprotein [Source:HGNC Symbol;Acc:HGNC:30856]"	HGLibA_23240
ENSG00000136710	CCDC115	1	-4.4314	-3.2141	0.0021598	0.79661	0.0013083	0.16457	coiled-coil domain containing 115 [Source:HGNC Symbol;Acc:HGNC:28178]	HGLibA_07554
ENSG00000243147	MRPL33	2	-3.5589	-4.5291	0.0142	0.82103	5.92E-06	0.0026859	mitochondrial ribosomal protein L33 [Source:HGNC Symbol;Acc:HGNC:14487]	HGLibA_29953;HGLibA_29955
ENSG00000154310	TNIK	2	-4.0471	-5.0719	0.0057902	0.81716	3.94E-07	0.00042039	TRAF2 and NCK interacting kinase [Source:HGNC Symbol;Acc:HGNC:30765]	HGLibA_50832;HGLibA_50833
ENSG00000083067	TRPM3	2	-4.1352	-5.0489	0.0047332	0.81716	4.44E-07	0.00042039	transient receptor potential cation channel subfamily M member 3 [Source:HGNC Symbol;Acc:HGNC:17992]	HGLibA_51759;HGLibA_51757
ENSG00000134574	DDB2	2	-2.4471	-2.916	0.040761	0.83741	0.003545	0.31712	damage specific DNA binding protein 2 [Source:HGNC Symbol;Acc:HGNC:2718]	HGLibA_12597;HGLibA_12598
SELRC1	SELRC1	2	-3.5067	-1.7316	0.015349	0.82103	0.083343	0.95326	-	HGLibA_43309;HGLibA_43311
hsa-mir-449b	hsa-mir-449b	2	-2.2307	-1.4959	0.047378	0.85774	0.13467	0.95326	-	HGLibA_60421;HGLibA_60420
ENSG00000174448	STARD6	1	-3.5026	-2.5884	0.015395	0.82103	0.0096418	0.58121	StAR related lipid transfer domain containing 6 [Source:HGNC Symbol;Acc:HGNC:18066]	HGLibA_47369
ENSG00000131174	COX7B	3	-2.9224	-4.1779	0.028905	0.83201	2.94E-05	0.010003	cytochrome c oxidase subunit 7B [Source:HGNC Symbol;Acc:HGNC:2291]	HGLibA_10867;HGLibA_10866;HGLibA_10865
ENSG00000198382	UVRAG	1	-2.1783	-1.7604	0.048849	0.86282	0.078347	0.95326	UV radiation resistance associated [Source:HGNC Symbol;Acc:HGNC:12640]	HGLibA_53474
ENSG00000156709	AIFM1	1	-2.866	-2.1781	0.030513	0.83236	0.029399	0.95326	apoptosis inducing factor mitochondria associated 1 [Source:HGNC Symbol;Acc:HGNC:8768]	HGLibA_01337
ENSG00000164935	DCSTAMP	2	-3.0953	-0.29782	0.025229	0.83016	0.76584	0.95774	dendrocyte expressed seven transmembrane protein [Source:HGNC Symbol;Acc:HGNC:18549]	HGLibA_12536;HGLibA_12535
ENSG00000186976	EFCAB6	3	-3.1321	-3.7678	0.02385	0.83016	0.00016467	0.03895	EF-hand calcium binding domain 6 [Source:HGNC Symbol;Acc:HGNC:24204]	HGLibA_14550;HGLibA_14548;HGLibA_14549
hsa-mir-4771-1	hsa-mir-4771-1	1	-2.9606	-2.2378	0.028078	0.83016	0.025235	0.95326	-	HGLibA_61180
hsa-mir-4539	hsa-mir-4539	2	-4.273	-2.533	0.0032168	0.81716	0.011311	0.62459	-	HGLibA_60617;HGLibA_60618
MESDC1	MESDC1	3	-2.1381	-2.6609	0.049906	0.86465	0.0077925	0.51542	-	HGLibA_28909;HGLibA_28911;HGLibA_28910
ENSG00000063177	RPL18	2	-2.4493	-2.8741	0.040715	0.83741	0.0040519	0.34504	ribosomal protein L18 [Source:HGNC Symbol;Acc:HGNC:10310]	HGLibA_41885;HGLibA_41886
ENSG00000143457	GOLPH3L	2	-2.4634	-2.7475	0.040256	0.83524	0.0060043	0.43699	golgi phosphoprotein 3 like [Source:HGNC Symbol;Acc:HGNC:24882]	HGLibA_19659;HGLibA_19660
ENSG00000134255	CEPT1	3	-3.9302	-4.4375	0.0074445	0.81716	9.10E-06	0.0037363	choline/ethanolamine phosphotransferase 1 [Source:HGNC Symbol;Acc:HGNC:24289]	HGLibA_09186;HGLibA_09184;HGLibA_09185
ENSG00000184402	SS18L1	2	-3.822	-4.5711	0.008915	0.81716	4.85E-06	0.0022949	nBAF chromatin remodeling complex subunit [Source:HGNC Symbol;Acc:HGNC:15592]"	HGLibA_47105;HGLibA_47104
ENSG00000116783	TNNI3K	2	-4.597	-4.0039	0.0010569	0.78846	6.23E-05	0.018325	TNNI3 interacting kinase [Source:HGNC Symbol;Acc:HGNC:19661]	HGLibA_50879;HGLibA_50880
ENSG00000144524	COPS7B	3	-2.4592	-2.1968	0.040301	0.83524	0.028037	0.95326	COP9 signalosome subunit 7B [Source:HGNC Symbol;Acc:HGNC:16760]	HGLibA_10730;HGLibA_10731;HGLibA_10732

Table S2 Negative candidate genes in 2nd screen

Gene	Gene Name	sgRNA	group|beta	group|z	group|p-value	group|fdr	group|wald-p-value	group|wald-fdr	Description	sgRNA ID
ENSG00000105875	WDR91	3	-2.6786	-0.93251	0.04151	0.87558	0.35107	0.95153	WD repeat domain 91 [Source:HGNC Symbol;Acc:HGNC:24997]	HGLibA_54218;HGLibA_54217;HGLibA_54216
ENSG00000149792	MRPL49	2	-2.7067	-2.8713	0.040316	0.87097	0.0040878	0.29774	mitochondrial ribosomal protein L49 [Source:HGNC Symbol;Acc:HGNC:1176]	HGLibA_30004;HGLibA_30006
ENSG00000177700	POLR2L	2	-2.4029	-2.2747	0.049454	0.87787	0.022922	0.88197	RNA polymerase II subunit L [Source:HGNC Symbol;Acc:HGNC:9199]	HGLibA_37603;HGLibA_37604
ENSG00000181938	GINS3	3	-3.1692	-1.1943	0.028561	0.84371	0.23234	0.95153	GINS complex subunit 3 [Source:HGNC Symbol;Acc:HGNC:25851]	HGLibA_19109;HGLibA_19108;HGLibA_19110
ENSG00000174429	ABRA	1	-3.7147	-2.729	0.014235	0.84371	0.0063535	0.39205	actin binding Rho activating protein [Source:HGNC Symbol;Acc:HGNC:30655]	HGLibA_00325
ENSG00000111341	MGP	2	-4.2221	-5.0271	0.004454	0.784	4.98E-07	0.0003189	matrix Gla protein [Source:HGNC Symbol;Acc:HGNC:7060]	HGLibA_29167;HGLibA_29168
ENSG00000160087	UBE2J2	2	-4.0199	-5.0521	0.0076224	0.80193	4.37E-07	0.00029142	ubiquitin conjugating enzyme E2 J2 [Source:HGNC Symbol;Acc:HGNC:19268]	HGLibA_52620;HGLibA_52622
ENSG00000176399	DMRTA1	2	-2.6073	-3.4541	0.043117	0.87558	0.00055204	0.073307	DMRT like family A1 [Source:HGNC Symbol;Acc:HGNC:13826]	HGLibA_13427;HGLibA_13426
ENSG00000247626	MARS2	2	-2.4318	-3.233	0.04826	0.87787	0.0012251	0.13612	mitochondrial [Source:HGNC Symbol;Acc:HGNC:25133]"	HGLibA_28379;HGLibA_28378
ENSG00000187398	LUZP2	1	-3.1803	-2.3783	0.02801	0.84371	0.017394	0.74422	leucine zipper protein 2 [Source:HGNC Symbol;Acc:HGNC:23206]	HGLibA_27640
ENSG00000173728	C1orf100	3	-3.0833	-0.50994	0.03026	0.84371	0.6101	0.95153	chromosome 1 open reading frame 100 [Source:HGNC Symbol;Acc:HGNC:30435]	HGLibA_05728;HGLibA_05730;HGLibA_05729
ENSG00000143314	MRPL24	3	-3.3716	-4.077	0.023464	0.84371	4.56E-05	0.011162	mitochondrial ribosomal protein L24 [Source:HGNC Symbol;Acc:HGNC:14037]	HGLibA_29937;HGLibA_29935;HGLibA_29936
ENSG00000120948	TARDBP	2	-3.8682	-4.7086	0.010883	0.84371	2.49E-06	0.0010977	TAR DNA binding protein [Source:HGNC Symbol;Acc:HGNC:11571]	HGLibA_48335;HGLibA_48336
ENSG00000189129	PLAC9	3	-3.7162	-2.6735	0.014235	0.84371	0.0075074	0.43483	placenta specific 9 [Source:HGNC Symbol;Acc:HGNC:19255]	HGLibA_36947;HGLibA_36948;HGLibA_36949
ENSG00000136522	MRPL47	2	-3.7985	-3.4812	0.012536	0.84371	0.00049908	0.067553	mitochondrial ribosomal protein L47 [Source:HGNC Symbol;Acc:HGNC:16652]	HGLibA_29999;HGLibA_30000
C16orf70	C16orf70	3	-2.457	-2.2074	0.047755	0.87787	0.027287	0.95153	-	HGLibA_05472;HGLibA_05470;HGLibA_05471
ENSG00000134184	GSTM1	2	-2.9068	-3.134	0.034806	0.85011	0.0017242	0.17465	glutathione S-transferase mu 1 [Source:HGNC Symbol;Acc:HGNC:4632]	HGLibA_20471;HGLibA_20472
ENSG00000078487	ZCWPW1	2	-3.0917	-0.29266	0.030214	0.84371	0.76978	0.95707	zinc finger CW-type and PWWP domain containing 1 [Source:HGNC Symbol;Acc:HGNC:23486]	HGLibA_55057;HGLibA_55056
ENSG00000187801	ZFP69B	2	-3.4274	-2.9979	0.022132	0.84371	0.0027188	0.23496	ZFP69 zinc finger protein B [Source:HGNC Symbol;Acc:HGNC:28053]	HGLibA_55233;HGLibA_55235
ENSG00000082482	KCNK2	1	-3.3728	-2.5033	0.023326	0.84371	0.012305	0.59552	potassium two pore domain channel subfamily K member 2 [Source:HGNC Symbol;Acc:HGNC:6277]	HGLibA_24319
ENSG00000071082	RPL31	3	-3.6578	-3.6	0.015428	0.84371	0.00031824	0.047147	ribosomal protein L31 [Source:HGNC Symbol;Acc:HGNC:10334]	HGLibA_41936;HGLibA_41937;HGLibA_41938
ENSG00000139780	METTL21C	3	-2.8564	-4.5306	0.036137	0.8559	5.88E-06	0.0020662	methyltransferase like 21C [Source:HGNC Symbol;Acc:HGNC:33717]	HGLibA_28981;HGLibA_28982;HGLibA_28983
ENSG00000176231	OR10H4	3	-3.1931	-3.4724	0.02778	0.84371	0.0005158	0.068915	olfactory receptor family 10 subfamily H member 4 [Source:HGNC Symbol;Acc:HGNC:15388]	HGLibA_33514;HGLibA_33515;HGLibA_33516
ENSG00000273559	CWC25	2	-3.7381	-3.2432	0.013684	0.84371	0.001182	0.13312	CWC25 spliceosome associated protein homolog [Source:HGNC Symbol;Acc:HGNC:25989]	HGLibA_11818;HGLibA_11819
ENSG00000119772	DNMT3A	1	-2.6853	-2.0654	0.040913	0.87097	0.038888	0.95153	DNA methyltransferase 3 alpha [Source:HGNC Symbol;Acc:HGNC:2978]	HGLibA_13701
ENSG00000173928	SWSAP1	3	-2.9234	-1.7021	0.034393	0.85011	0.088733	0.95153	SWIM-type zinc finger 7 associated protein 1 [Source:HGNC Symbol;Acc:HGNC:26638]	HGLibA_47900;HGLibA_47902;HGLibA_47901
ENSG00000116670	MAD2L2	1	-4.2333	-3.0787	0.0042244	0.784	0.0020792	0.19602	mitotic arrest deficient 2 like 2 [Source:HGNC Symbol;Acc:HGNC:6764]	HGLibA_27850
ENSG00000185158	LRRC37B	3	-2.6048	-3.1669	0.043117	0.87558	0.0015407	0.16093	leucine rich repeat containing 37B [Source:HGNC Symbol;Acc:HGNC:29070]	HGLibA_27334;HGLibA_27336;HGLibA_27335
SF3B14	SF3B14	3	-3.1795	-4.8152	0.02824	0.84371	1.47E-06	0.0008005	-	HGLibA_43702;HGLibA_43704;HGLibA_43703
ENSG00000131495	NDUFA2	1	-3.0679	-2.3061	0.030489	0.84371	0.021105	0.83392	NADH:ubiquinone oxidoreductase subunit A2 [Source:HGNC Symbol;Acc:HGNC:7685]	HGLibA_31452
ENSG00000114268	PFKFB4	1	-2.7286	-2.0839	0.039719	0.87097	0.037172	0.95153	6-biphosphatase 4 [Source:HGNC Symbol;Acc:HGNC:8875]"	HGLibA_36162
hsa-mir-6722	hsa-mir-6722	4	-2.6306	-1.2003	0.042428	0.87558	0.23004	0.95153	-	HGLibA_63048;HGLibA_63050;HGLibA_63051;HGLibA_63049
ENSG00000141255	SPATA22	2	-2.9564	-3.8497	0.033199	0.84561	0.00011827	0.022204	spermatogenesis associated 22 [Source:HGNC Symbol;Acc:HGNC:30705]	HGLibA_46452;HGLibA_46454
ENSG00000105771	SMG9	1	-3.9843	-2.9098	0.0083111	0.83871	0.0036171	0.27835	nonsense mediated mRNA decay factor [Source:HGNC Symbol;Acc:HGNC:25763]"	HGLibA_45713
ENSG00000136770	DNAJC1	2	-3.3442	-1.5141	0.024107	0.84371	0.12999	0.95153	DnaJ heat shock protein family (Hsp40) member C1 [Source:HGNC Symbol;Acc:HGNC:20090]	HGLibA_13560;HGLibA_13561
C11orf31	C11orf31	3	-2.5772	-0.50382	0.04353	0.87616	0.61439	0.95153	-	HGLibA_05130;HGLibA_05131;HGLibA_05129
ENSG00000112041	TULP1	1	-3.8129	-2.7946	0.01203	0.84371	0.0051968	0.34252	tubby like protein 1 [Source:HGNC Symbol;Acc:HGNC:12423]	HGLibA_52330
ENSG00000197756	RPL37A	2	-2.6309	-1.7672	0.042428	0.87558	0.077187	0.95153	ribosomal protein L37a [Source:HGNC Symbol;Acc:HGNC:10348]	HGLibA_41967;HGLibA_41968
ENSG00000146263	MMS22L	2	-2.9332	-3.7874	0.034025	0.85011	0.00015226	0.025603	DNA repair protein [Source:HGNC Symbol;Acc:HGNC:21475]"	HGLibA_29555;HGLibA_29556
ENSG00000221995	TIAF1	2	-3.1229	-2.8809	0.029342	0.84371	0.0039654	0.29274	TGFB1-induced anti-apoptotic factor 1 [Source:HGNC Symbol;Acc:HGNC:11803]	HGLibA_49432;HGLibA_49433
ENSG00000149932	TMEM219	2	-2.9368	-0.99585	0.033841	0.85011	0.31932	0.95153	transmembrane protein 219 [Source:HGNC Symbol;Acc:HGNC:25201]	HGLibA_50232;HGLibA_50230
ENSG00000130305	NSUN5	2	-2.9978	-1.3546	0.032234	0.84371	0.17553	0.95153	NOP2/Sun RNA methyltransferase family member 5 [Source:HGNC Symbol;Acc:HGNC:16385]	HGLibA_32790;HGLibA_32791
ENSG00000171877	FRMD5	1	-3.2487	-2.4225	0.026127	0.84371	0.015414	0.6925	FERM domain containing 5 [Source:HGNC Symbol;Acc:HGNC:28214]	HGLibA_18019
DUX2	DUX2	1	-3.3127	-2.4641	0.024658	0.84371	0.013737	0.64199	-	HGLibA_14202
ENSG00000121957	GPSM2	3	-3.8689	-4.6166	0.010883	0.84371	3.90E-06	0.0014909	G protein signaling modulator 2 [Source:HGNC Symbol;Acc:HGNC:29501]	HGLibA_20158;HGLibA_20159;HGLibA_20157
hsa-mir-1301	hsa-mir-1301	2	-2.8614	-2.8912	0.036046	0.8559	0.0038382	0.28785	-	HGLibA_57678;HGLibA_57680
ENSG00000170509	HSD17B13	2	-3.1407	-3.8412	0.029158	0.84371	0.00012243	0.022507	hydroxysteroid 17-beta dehydrogenase 13 [Source:HGNC Symbol;Acc:HGNC:18685]	HGLibA_22180;HGLibA_22181
ENSG00000109971	HSPA8	1	-4.1438	-3.0177	0.0054642	0.79902	0.0025467	0.22637	heat shock protein family A (Hsp70) member 8 [Source:HGNC Symbol;Acc:HGNC:5241]	HGLibA_22296
ENSG00000119760	SUPT7L	3	-2.9774	-2.9732	0.032923	0.84371	0.002947	0.2459	STAGA complex gamma subunit [Source:HGNC Symbol;Acc:HGNC:30632]"	HGLibA_47825;HGLibA_47826;HGLibA_47827
ENSG00000173614	NMNAT1	1	-4.2333	-3.0787	0.0042244	0.784	0.0020792	0.19602	nicotinamide nucleotide adenylyltransferase 1 [Source:HGNC Symbol;Acc:HGNC:17877]	HGLibA_32183
ENSG00000132744	ACY3	2	-2.6933	-0.83302	0.040454	0.87097	0.40483	0.95153	aminoacylase 3 [Source:HGNC Symbol;Acc:HGNC:24104]	HGLibA_00706;HGLibA_00708
ENSG00000245848	CEBPA	2	-2.6956	-2.8119	0.040408	0.87097	0.0049246	0.33406	CCAAT enhancer binding protein alpha [Source:HGNC Symbol;Acc:HGNC:1833]	HGLibA_08955;HGLibA_08957
ENSG00000144744	UBA3	2	-2.8072	-3.0744	0.037193	0.85956	0.0021093	0.198	ubiquitin like modifier activating enzyme 3 [Source:HGNC Symbol;Acc:HGNC:12470]	HGLibA_52519;HGLibA_52520
ENSG00000186889	TMEM17	1	-3.1068	-2.331	0.029663	0.84371	0.019753	0.80279	transmembrane protein 17 [Source:HGNC Symbol;Acc:HGNC:26623]	HGLibA_50050
ENSG00000174233	ADCY6	2	-2.4732	-2.4932	0.047387	0.87787	0.012661	0.60736	adenylate cyclase 6 [Source:HGNC Symbol;Acc:HGNC:237]	HGLibA_00922;HGLibA_00923
ENSG00000182180	MRPS16	2	-3.4605	-4.3829	0.021076	0.84371	1.17E-05	0.0038065	mitochondrial ribosomal protein S16 [Source:HGNC Symbol;Acc:HGNC:14048]	HGLibA_30044;HGLibA_30043
HIST2H2BF	HIST2H2BF	2	-2.8794	-2.3077	0.03554	0.85525	0.021013	0.83392	-	HGLibA_21536;HGLibA_21538
ENSG00000111445	RFC5	1	-3.9023	-2.8545	0.010377	0.84371	0.0043099	0.30977	replication factor C subunit 5 [Source:HGNC Symbol;Acc:HGNC:9973]	HGLibA_40914
ENSG00000140522	RLBP1	3	-2.4786	-2.1297	0.047295	0.87787	0.033194	0.95153	retinaldehyde binding protein 1 [Source:HGNC Symbol;Acc:HGNC:10024]	HGLibA_41342;HGLibA_41343;HGLibA_41344
ENSG00000167103	PIP5KL1	3	-3.2767	-2.5046	0.025668	0.84371	0.012258	0.59552	phosphatidylinositol-4-phosphate 5-kinase like 1 [Source:HGNC Symbol;Acc:HGNC:28711]	HGLibA_36722;HGLibA_36723;HGLibA_36724
ENSG00000082516	GEMIN5	1	-3.3354	-2.4655	0.024382	0.84371	0.013681	0.64199	gem nuclear organelle associated protein 5 [Source:HGNC Symbol;Acc:HGNC:20043]	HGLibA_18918
ENSG00000196946	ZNF705A	1	-3.9359	-2.8772	0.0094591	0.84371	0.0040128	0.29425	zinc finger protein 705A [Source:HGNC Symbol;Acc:HGNC:32281]	HGLibA_56588
ENSG00000133119	RFC3	2	-3.9079	-2.8119	0.010332	0.84371	0.0049256	0.33406	replication factor C subunit 3 [Source:HGNC Symbol;Acc:HGNC:9971]	HGLibA_40909;HGLibA_40907
ENSG00000108559	NUP88	2	-2.6993	-3.5374	0.040362	0.87097	0.00040402	0.057886	nucleoporin 88 [Source:HGNC Symbol;Acc:HGNC:8067]	HGLibA_33073;HGLibA_33071
ENSG00000119537	KDSR	2	-3.8496	-4.6054	0.011571	0.84371	4.12E-06	0.0015457	3-ketodihydrosphingosine reductase [Source:HGNC Symbol;Acc:HGNC:4021]	HGLibA_24546;HGLibA_24545
ENSG00000173976	RAX2	2	-3.8828	-3.8293	0.010791	0.84371	0.00012851	0.023322	retina and anterior neural fold homeobox 2 [Source:HGNC Symbol;Acc:HGNC:18286]	HGLibA_40411;HGLibA_40409
ENSG00000136636	KCTD3	2	-3.4179	-4.1644	0.022316	0.84371	3.12E-05	0.0081922	potassium channel tetramerization domain containing 3 [Source:HGNC Symbol;Acc:HGNC:21305]	HGLibA_24452;HGLibA_24453
HIAT1	HIAT1	2	-3.9003	-3.7951	0.010653	0.84371	0.0001476	0.025311	-	HGLibA_21276;HGLibA_21275
ENSG00000161999	JMJD8	2	-4.2035	-5.1408	0.0046836	0.79902	2.74E-07	0.00019857	jumonji domain containing 8 [Source:HGNC Symbol;Acc:HGNC:14148]	HGLibA_23968;HGLibA_23967
ENSG00000125814	NAPB	1	-4.1959	-3.0532	0.0047755	0.79902	0.0022641	0.20717	NSF attachment protein beta [Source:HGNC Symbol;Acc:HGNC:15751]	HGLibA_31128
ENSG00000182568	SATB1	2	-3.7935	-4.7189	0.012673	0.84371	2.37E-06	0.0010977	SATB homeobox 1 [Source:HGNC Symbol;Acc:HGNC:10541]	HGLibA_42777;HGLibA_42776
ENSG00000165194	PCDH19	3	-3.3509	-1.6693	0.023969	0.84371	0.095068	0.95153	protocadherin 19 [Source:HGNC Symbol;Acc:HGNC:14270]	HGLibA_35408;HGLibA_35409;HGLibA_35410
ENSG00000213420	GPC2	3	-2.6557	-1.7406	0.041831	0.87558	0.081758	0.95153	glypican 2 [Source:HGNC Symbol;Acc:HGNC:4450]	HGLibA_19764;HGLibA_19765;HGLibA_19766
ENSG00000101327	PDYN	1	-3.0679	-2.3061	0.030489	0.84371	0.021105	0.83392	prodynorphin [Source:HGNC Symbol;Acc:HGNC:8820]	HGLibA_35966
ENSG00000149636	DSN1	2	-2.8044	-2.4223	0.037193	0.85956	0.015422	0.6925	MIS12 kinetochore complex component [Source:HGNC Symbol;Acc:HGNC:16165]"	HGLibA_14034;HGLibA_14035
ENSG00000172247	C1QTNF4	3	-2.4855	-2.9848	0.047158	0.87787	0.0028379	0.24184	C1q and TNF related 4 [Source:HGNC Symbol;Acc:HGNC:14346]	HGLibA_05944;HGLibA_05946;HGLibA_05945
ENSG00000185633	NDUFA4L2	2	-2.5203	-0.83431	0.045688	0.87787	0.40411	0.95153	mitochondrial complex associated like 2 [Source:HGNC Symbol;Acc:HGNC:29836]"	HGLibA_31460;HGLibA_31461
ENSG00000204301	NOTCH4	2	-2.8422	-0.67898	0.036551	0.85868	0.49715	0.95153	notch 4 [Source:HGNC Symbol;Acc:HGNC:7884]	HGLibA_32351;HGLibA_32352
hsa-mir-6783	hsa-mir-6783	3	-3.6773	-4.4264	0.015153	0.84371	9.58E-06	0.0031612	-	HGLibA_63311;HGLibA_63308;HGLibA_63309
ENSG00000157895	C12orf43	1	-3.0274	-2.2802	0.031362	0.84371	0.022593	0.87251	chromosome 12 open reading frame 43 [Source:HGNC Symbol;Acc:HGNC:25719]	HGLibA_05243
ENSG00000005436	GCFC2	3	-2.6321	-3.511	0.042428	0.87558	0.00044649	0.062331	GC-rich sequence DNA-binding factor 2 [Source:HGNC Symbol;Acc:HGNC:1317]	HGLibA_18772;HGLibA_18773;HGLibA_18774
ENSG00000107443	CCNJ	2	-2.91	-3.0679	0.03453	0.85011	0.0021555	0.20061	cyclin J [Source:HGNC Symbol;Acc:HGNC:23434]	HGLibA_08109;HGLibA_08108
hsa-mir-1273h	hsa-mir-1273h	2	-4.0504	-2.5655	0.007255	0.79902	0.010304	0.53556	-	HGLibA_57553;HGLibA_57550
ENSG00000198129	DEFB107B	1	-3.1068	-2.331	0.029663	0.84371	0.019753	0.80279	defensin beta 107B [Source:HGNC Symbol;Acc:HGNC:31918]	HGLibA_12839
ENSG00000111640	GAPDH	2	-2.9292	-2.9242	0.034209	0.85011	0.0034533	0.27052	glyceraldehyde-3-phosphate dehydrogenase [Source:HGNC Symbol;Acc:HGNC:4141]	HGLibA_18614;HGLibA_18613
ENSG00000012048	BRCA1	3	-3.6148	-4.2189	0.016071	0.84371	2.46E-05	0.0069438	DNA repair associated [Source:HGNC Symbol;Acc:HGNC:1100]"	HGLibA_04762;HGLibA_04760;HGLibA_04761
hsa-mir-1299	hsa-mir-1299	1	-3.6507	-2.6864	0.015474	0.84371	0.0072228	0.42284	-	HGLibA_57674
ENSG00000100316	RPL3	1	-3.3728	-2.5033	0.023326	0.84371	0.012305	0.59552	ribosomal protein L3 [Source:HGNC Symbol;Acc:HGNC:10332]	HGLibA_41932
ENSG00000213281	NRAS	1	-2.9852	-2.2534	0.032464	0.84371	0.024235	0.91789	GTPase [Source:HGNC Symbol;Acc:HGNC:7989]"	HGLibA_32649
ENSG00000125743	SNRPD2	2	-3.8057	-2.6118	0.012352	0.84371	0.009007	0.48577	small nuclear ribonucleoprotein D2 polypeptide [Source:HGNC Symbol;Acc:HGNC:11159]	HGLibA_45978;HGLibA_45979
ENSG00000005102	MEOX1	2	-2.5941	-1.5181	0.043301	0.87558	0.12898	0.95153	mesenchyme homeobox 1 [Source:HGNC Symbol;Acc:HGNC:7013]	HGLibA_28890;HGLibA_28889
ENSG00000265354	TIMM23	1	-3.3728	-2.5033	0.023326	0.84371	0.012305	0.59552	translocase of inner mitochondrial membrane 23 [Source:HGNC Symbol;Acc:HGNC:17312]	HGLibA_49515
ENSG00000046647	GEMIN8	2	-3.3076	-2.6944	0.025071	0.84371	0.0070506	0.42001	gem nuclear organelle associated protein 8 [Source:HGNC Symbol;Acc:HGNC:26044]	HGLibA_18926;HGLibA_18925
ENSG00000162761	LMX1A	3	-3.1828	-1.4763	0.027964	0.84371	0.13986	0.95153	LIM homeobox transcription factor 1 alpha [Source:HGNC Symbol;Acc:HGNC:6653]	HGLibA_26733;HGLibA_26732;HGLibA_26731
TCEB1	TCEB1	2	-3.5481	-3.6042	0.018505	0.84371	0.00031307	0.046699	-	HGLibA_48734;HGLibA_48733
ENSG00000149506	ZP1	3	-3.0708	-3.5066	0.030444	0.84371	0.00045391	0.062963	zona pellucida glycoprotein 1 [Source:HGNC Symbol;Acc:HGNC:13187]	HGLibA_56950;HGLibA_56951;HGLibA_56949
ENSG00000163728	TTC14	2	-3.723	-4.7065	0.014143	0.84371	2.52E-06	0.0010977	tetratricopeptide repeat domain 14 [Source:HGNC Symbol;Acc:HGNC:24697]	HGLibA_52055;HGLibA_52053
ENSG00000187068	C3orf70	2	-2.5647	-0.40445	0.043989	0.87787	0.68588	0.95153	chromosome 3 open reading frame 70 [Source:HGNC Symbol;Acc:HGNC:33731]	HGLibA_06262;HGLibA_06263
ENSG00000116984	MTR	2	-2.5922	-2.0233	0.043301	0.87558	0.043041	0.95153	5-methyltetrahydrofolate-homocysteine methyltransferase [Source:HGNC Symbol;Acc:HGNC:7468]	HGLibA_30462;HGLibA_30463
ENSG00000197063	MAFG	2	-3.2806	-3.8259	0.025576	0.84371	0.00013027	0.023446	MAF bZIP transcription factor G [Source:HGNC Symbol;Acc:HGNC:6781]	HGLibA_27881;HGLibA_27882
ENSG00000179046	TRIML2	1	-3.4016	-2.5221	0.022546	0.84371	0.011667	0.57992	tripartite motif family like 2 [Source:HGNC Symbol;Acc:HGNC:26378]	HGLibA_51623
ENSG00000099800	TIMM13	1	-2.895	-2.1963	0.034898	0.85011	0.028072	0.95153	translocase of inner mitochondrial membrane 13 [Source:HGNC Symbol;Acc:HGNC:11816]	HGLibA_49499
ENSG00000163702	IL17RC	3	-2.6458	-2.1404	0.042107	0.87558	0.032319	0.95153	interleukin 17 receptor C [Source:HGNC Symbol;Acc:HGNC:18358]	HGLibA_23011;HGLibA_23010;HGLibA_23009
hsa-let-7i	hsa-let-7i	2	-2.9214	-2.5921	0.034393	0.85011	0.009539	0.50545	-	HGLibA_57138;HGLibA_57137
ENSG00000204661	C5orf60	1	-3.6057	-2.6565	0.016393	0.84371	0.0078957	0.44998	chromosome 5 open reading frame 60 [Source:HGNC Symbol;Acc:HGNC:27753]	HGLibA_06417
ENSG00000126457	PRMT1	2	-2.9327	-3.6845	0.034071	0.85011	0.00022918	0.037247	protein arginine methyltransferase 1 [Source:HGNC Symbol;Acc:HGNC:5187]	HGLibA_38616;HGLibA_38618
ENSG00000100263	RHBDD3	2	-4.3179	-4.89	0.003352	0.784	1.01E-06	0.00057777	rhomboid domain containing 3 [Source:HGNC Symbol;Acc:HGNC:1308]	HGLibA_41131;HGLibA_41129
ENSG00000185650	ZFP36L1	2	-3.1906	-2.7093	0.027826	0.84371	0.0067424	0.40901	ZFP36 ring finger protein like 1 [Source:HGNC Symbol;Acc:HGNC:1107]	HGLibA_55207;HGLibA_55206
ENSG00000155621	C9orf85	1	-3.7748	-2.7691	0.012995	0.84371	0.0056217	0.36222	chromosome 9 open reading frame 85 [Source:HGNC Symbol;Acc:HGNC:28784]	HGLibA_06768
ENSG00000101444	AHCY	2	-3.2102	-3.2113	0.027551	0.84371	0.0013212	0.14174	adenosylhomocysteinase [Source:HGNC Symbol;Acc:HGNC:343]	HGLibA_01286;HGLibA_01287
ENSG00000205208	C4orf46	3	-3.5796	-5.194	0.017495	0.84371	2.06E-07	0.0001601	chromosome 4 open reading frame 46 [Source:HGNC Symbol;Acc:HGNC:27320]	HGLibA_06334;HGLibA_06335;HGLibA_06336
ENSG00000146215	CRIP3	2	-4.8429	-5.6452	0.00055101	0.59091	1.65E-08	2.20E-05	cysteine rich protein 3 [Source:HGNC Symbol;Acc:HGNC:17751]	HGLibA_11156;HGLibA_11157

ENSG00000103852	TTC23	2	-3.367	-4.1801	0.02351	0.84371	2.91E-05	0.0077388	tetratricopeptide repeat domain 23 [Source:HGNC Symbol;Acc:HGNC:25730]	HGLibA_52079;HGLibA_52078
ENSG00000164054	SHISA5	1	-3.9192	-2.8659	0.0099642	0.84371	0.0041577	0.30082	shisa family member 5 [Source:HGNC Symbol;Acc:HGNC:30376]	HGLibA_44042
ENSG00000171960	PPIH	1	-2.9411	-2.2254	0.033658	0.84936	0.026053	0.95153	peptidylprolyl isomerase H [Source:HGNC Symbol;Acc:HGNC:14651]	HGLibA_37936
ENSG00000241945	PWP2	2	-2.4692	-3.2408	0.047525	0.87787	0.0011919	0.13312	small subunit processome component [Source:HGNC Symbol;Acc:HGNC:9711]"	HGLibA_39636;HGLibA_39637
ENSG00000075914	EXOSC7	2	-3.1218	-3.8476	0.029387	0.84371	0.00011929	0.022205	exosome component 7 [Source:HGNC Symbol;Acc:HGNC:28112]	HGLibA_15790;HGLibA_15791
ENSG00000132141	CCT6B	3	-2.6103	-0.49182	0.043117	0.87558	0.62284	0.95153	chaperonin containing TCP1 subunit 6B [Source:HGNC Symbol;Acc:HGNC:1621]	HGLibA_08210;HGLibA_08209;HGLibA_08211
ENSG00000184206	GOLGA6L4	3	-2.8464	-1.1088	0.036413	0.8573	0.26752	0.95153	golgin A6 family-like 4 [Source:HGNC Symbol;Acc:HGNC:27256]	HGLibA_19625;HGLibA_19624;HGLibA_19626
ENSG00000157184	CPT2	2	-2.9332	-3.7874	0.034025	0.85011	0.00015226	0.025603	carnitine palmitoyltransferase 2 [Source:HGNC Symbol;Acc:HGNC:2330]	HGLibA_11026;HGLibA_11027
ENSG00000165480	SKA3	1	-3.1068	-2.331	0.029663	0.84371	0.019753	0.80279	spindle and kinetochore associated complex subunit 3 [Source:HGNC Symbol;Acc:HGNC:20262]	HGLibA_44266
ENSG00000165487	MICU2	3	-4.4672	-5.218	0.0021122	0.76812	1.81E-07	0.00015579	mitochondrial calcium uptake 2 [Source:HGNC Symbol;Acc:HGNC:31830]	HGLibA_29225;HGLibA_29224;HGLibA_29226
ENSG00000100401	RANGAP1	2	-4.4412	-4.0913	0.0023877	0.76812	4.29E-05	0.010615	Ran GTPase activating protein 1 [Source:HGNC Symbol;Acc:HGNC:9854]	HGLibA_40215;HGLibA_40214
ENSG00000168701	TMEM208	2	-3.1938	-3.5112	0.02778	0.84371	0.00044613	0.062331	transmembrane protein 208 [Source:HGNC Symbol;Acc:HGNC:25015]	HGLibA_50202;HGLibA_50200
ENSG00000080345	RIF1	3	-2.5769	-3.9751	0.04353	0.87616	7.04E-05	0.015023	replication timing regulatory factor 1 [Source:HGNC Symbol;Acc:HGNC:23207]	HGLibA_41243;HGLibA_41244;HGLibA_41245
hsa-mir-3923	hsa-mir-3923	4	-2.8697	-0.81243	0.035862	0.8559	0.41654	0.95153	-	HGLibA_59568;HGLibA_59570;HGLibA_59569;HGLibA_59567
ENSG00000198815	FOXJ3	1	-3.6725	-2.7009	0.015199	0.84371	0.0069158	0.41605	forkhead box J3 [Source:HGNC Symbol;Acc:HGNC:29178]	HGLibA_17891
FAM173A	FAM173A	1	-3.1442	-2.355	0.028928	0.84371	0.01852	0.78167	-	HGLibA_16276
ENSG00000130382	MLLT1	3	-2.4775	-1.8786	0.047295	0.87787	0.060302	0.95153	super elongation complex subunit [Source:HGNC Symbol;Acc:HGNC:7134]"	HGLibA_29408;HGLibA_29407;HGLibA_29409
ENSG00000175334	BANF1	2	-3.3811	-3.3692	0.023097	0.84371	0.00075389	0.093819	barrier to autointegration factor 1 [Source:HGNC Symbol;Acc:HGNC:17397]	HGLibA_04139;HGLibA_04141;HGLibA_04140
ENSG00000136444	RSAD1	3	-2.6198	-1.1184	0.043071	0.87558	0.26339	0.95153	radical S-adenosyl methionine domain containing 1 [Source:HGNC Symbol;Acc:HGNC:25634]	HGLibA_42295;HGLibA_42296;HGLibA_42297
ENSG00000101452	DHX35	2	-2.443	-0.45644	0.047938	0.87787	0.64807	0.95153	DEAH-box helicase 35 [Source:HGNC Symbol;Acc:HGNC:15861]	HGLibA_13188;HGLibA_13186
ENSG00000103319	EEF2K	1	-4.08	-2.963	0.006704	0.79902	0.0030467	0.25207	eukaryotic elongation factor 2 kinase [Source:HGNC Symbol;Acc:HGNC:24615]	HGLibA_14510
ENSG00000162746	FCRLB	3	-2.4976	-3.1072	0.046285	0.87787	0.0018888	0.18529	Fc receptor like B [Source:HGNC Symbol;Acc:HGNC:26431]	HGLibA_17246;HGLibA_17245;HGLibA_17247
ENSG00000124787	RPP40	3	-2.3998	-2.0488	0.049454	0.87787	0.040481	0.95153	ribonuclease P/MRP subunit p40 [Source:HGNC Symbol;Acc:HGNC:20992]	HGLibA_42041;HGLibA_42042;HGLibA_42040
ENSG00000128944	KNSTRN	2	-3.4983	-3.8817	0.019974	0.84371	0.00010372	0.020534	kinetochore localized astrin (SPAG5) binding protein [Source:HGNC Symbol;Acc:HGNC:30767]	HGLibA_25347;HGLibA_25349
ENSG00000131264	CDX4	3	-2.8195	-1.2966	0.036918	0.85956	0.19476	0.95153	caudal type homeobox 4 [Source:HGNC Symbol;Acc:HGNC:1808]	HGLibA_08902;HGLibA_08901;HGLibA_08903
ENSG00000130559	CAMSAP1	2	-2.4202	-0.89326	0.048581	0.87787	0.37172	0.95153	calmodulin regulated spectrin associated protein 1 [Source:HGNC Symbol;Acc:HGNC:19946]	HGLibA_07105;HGLibA_07106
ENSG00000112339	HBS1L	2	-3.8715	-4.0314	0.010883	0.84371	5.54E-05	0.013033	HBS1 like translational GTPase [Source:HGNC Symbol;Acc:HGNC:4834]	HGLibA_20941;HGLibA_20939
G6PC	G6PC	3	-3.2466	-0.46502	0.026449	0.84371	0.64191	0.95153	-	HGLibA_18304;HGLibA_18306;HGLibA_18305
ENSG00000122692	SMU1	2	-2.9645	-1.1699	0.032969	0.84371	0.24203	0.95153	DNA replication regulator and spliceosomal factor [Source:HGNC Symbol;Acc:HGNC:18247]"	HGLibA_45839;HGLibA_45838
ENSG00000117448	AKR1A1	1	-4.2963	-3.1096	0.0034898	0.784	0.0018732	0.18459	aldo-keto reductase family 1 member A1 [Source:HGNC Symbol;Acc:HGNC:380]	HGLibA_01468
ENSG00000137959	TMPRSS3	3	-3.6488	-4.0242	0.01552	0.84371	5.72E-05	0.013103	transmembrane serine protease 3 [Source:HGNC Symbol;Acc:HGNC:11877]	HGLibA_50639;HGLibA_50638;HGLibA_50640
ENSG00000108883	EFTUD2	1	-3.2487	-2.4225	0.026127	0.84371	0.015414	0.6925	elongation factor Tu GTP binding domain containing 2 [Source:HGNC Symbol;Acc:HGNC:30858]	HGLibA_14618
ENSG00000071205	ARHGAP10	1	-3.9684	-2.8991	0.0087244	0.84371	0.0037429	0.28303	Rho GTPase activating protein 10 [Source:HGNC Symbol;Acc:HGNC:26099]	HGLibA_02700
ENSG00000136104	RNASEH2B	2	-2.8804	-2.5593	0.035495	0.85525	0.010489	0.53915	ribonuclease H2 subunit B [Source:HGNC Symbol;Acc:HGNC:25671]	HGLibA_41429;HGLibA_41431
ENSG00000183474	GTF2H2C	3	-2.6197	-3.9177	0.043071	0.87558	8.94E-05	0.018545	GTF2H2 family member C [Source:HGNC Symbol;Acc:HGNC:31394]	HGLibA_20550;HGLibA_20549;HGLibA_20551
ENSG00000167311	ART5	3	-4.2834	-3.2202	0.0035816	0.784	0.0012808	0.13947	ADP-ribosyltransferase 5 [Source:HGNC Symbol;Acc:HGNC:24049]	HGLibA_03168;HGLibA_03167;HGLibA_03166
ENSG00000198947	DMD	2	-3.0428	-3.8985	0.030995	0.84371	9.68E-05	0.019341	dystrophin [Source:HGNC Symbol;Acc:HGNC:2928]	HGLibA_13402;HGLibA_13403
hsa-mir-548u	hsa-mir-548u	3	-3.2912	-1.6178	0.025255	0.84371	0.1057	0.95153	-	HGLibA_62072;HGLibA_62069;HGLibA_62070
ENSG00000203778	FAM229B	3	-4.0457	-6.252	0.0073009	0.79902	4.05E-10	1.26E-06	family with sequence similarity 229 member B [Source:HGNC Symbol;Acc:HGNC:33858]	HGLibA_16544;HGLibA_16543;HGLibA_16542
ENSG00000180090	OR3A1	3	-2.9813	-3.803	0.032739	0.84371	0.00014295	0.024905	olfactory receptor family 3 subfamily A member 1 [Source:HGNC Symbol;Acc:HGNC:8282]	HGLibA_33914;HGLibA_33913;HGLibA_33915
TMEM194B	TMEM194B	3	-3.3032	-5.3242	0.025117	0.84371	1.01E-07	9.60E-05	-	HGLibA_50157;HGLibA_50155;HGLibA_50156
ENSG00000156162	DPY19L4	3	-3.0083	-1.3183	0.032005	0.84371	0.18739	0.95153	dpy-19 like 4 [Source:HGNC Symbol;Acc:HGNC:27829]	HGLibA_13915;HGLibA_13913;HGLibA_13914
ENSG00000054267	ARID4B	2	-3.6122	-4.5858	0.016209	0.84371	4.52E-06	0.0016693	AT-rich interaction domain 4B [Source:HGNC Symbol;Acc:HGNC:15550]	HGLibA_02902;HGLibA_02903
ENSG00000198415	PLAGL1	2	-3.9381	-4.7801	0.0094132	0.84371	1.75E-06	0.00088754	PLAG1 like zinc finger 1 [Source:HGNC Symbol;Acc:HGNC:9046]	HGLibA_36955;HGLibA_36953
ENSG00000101442	ACTR5	2	-2.9855	-2.9426	0.032464	0.84371	0.0032548	0.26011	ARP5 actin related protein 5 homolog [Source:HGNC Symbol;Acc:HGNC:14671]	HGLibA_00667;HGLibA_00669
hsa-mir-3118-2	hsa-mir-3118-2	1	-3.7552	-2.756	0.01327	0.84371	0.0058521	0.37157	-	HGLibA_58515
hsa-mir-3118-1	hsa-mir-3118-1	1	-2.5612	-1.9891	0.044081	0.87787	0.046694	0.95153	-	HGLibA_58514
ENSG00000152253	SPC25	2	-3.3884	-3.9909	0.022959	0.84371	6.58E-05	0.01478	NDC80 kinetochore complex component [Source:HGNC Symbol;Acc:HGNC:24031]"	HGLibA_46556;HGLibA_46554
ENSG00000134697	GNL2	2	-2.4692	-3.2408	0.047525	0.87787	0.0011919	0.13312	G protein nucleolar 2 [Source:HGNC Symbol;Acc:HGNC:29925]	HGLibA_19546;HGLibA_19547
ENSG00000198691	ABCA4	3	-3.0896	-3.801	0.030214	0.84371	0.00014413	0.024911	ATP binding cassette subfamily A member 4 [Source:HGNC Symbol;Acc:HGNC:34]	HGLibA_00114;HGLibA_00113;HGLibA_00112
ENSG00000075702	WDR62	1	-3.4016	-2.5221	0.022546	0.84371	0.011667	0.57992	WD repeat domain 62 [Source:HGNC Symbol;Acc:HGNC:24502]	HGLibA_54146
ENSG00000069849	ATP1B3	3	-3.3902	-4.1327	0.022959	0.84371	3.59E-05	0.0091874	ATPase Na+/K+ transporting subunit beta 3 [Source:HGNC Symbol;Acc:HGNC:806]	HGLibA_03600;HGLibA_03599;HGLibA_03598
ENSG00000148513	ANKRD30A	3	-3.5252	-3.5899	0.019423	0.84371	0.00033077	0.048672	ankyrin repeat domain 30A [Source:HGNC Symbol;Acc:HGNC:17234]	HGLibA_02059;HGLibA_02061;HGLibA_02060
ENSG00000279782	PPIAL4F	2	-3.6389	-2.4567	0.015658	0.84371	0.014023	0.65395	peptidylprolyl isomerase A like 4F [Source:HGNC Symbol;Acc:HGNC:33999]	HGLibA_37912;HGLibA_37913
ENSG00000126216	TUBGCP3	1	-2.7383	-2.0826	0.039627	0.87097	0.037292	0.95153	tubulin gamma complex associated protein 3 [Source:HGNC Symbol;Acc:HGNC:18598]	HGLibA_52311
ENSG00000179862	CITED4	1	-3.9023	-2.8545	0.010377	0.84371	0.0043099	0.30977	Cbp/p300 interacting transactivator with Glu/Asp rich carboxy-terminal domain 4 [Source:HGNC Symbol;Acc:HGNC:18696]	HGLibA_09747
ENSG00000284194	SCO2	2	-2.6066	-3.3286	0.043117	0.87558	0.00087273	0.10386	cytochrome c oxidase assembly protein [Source:NCBI gene;Acc:9997]"	HGLibA_43029;HGLibA_43028
ENSG00000118508	RAB32	3	-2.6849	-0.88514	0.041326	0.87558	0.37608	0.95153	member RAS oncogene family [Source:HGNC Symbol;Acc:HGNC:9772]"	HGLibA_39878;HGLibA_39877;HGLibA_39876
H2AFZ	H2AFZ	1	-2.7421	-2.1005	0.03903	0.86735	0.035682	0.95153	-	HGLibA_20770
ENSG00000145247	OCIAD2	2	-3.1416	-4.0296	0.029158	0.84371	5.59E-05	0.013033	OCIA domain containing 2 [Source:HGNC Symbol;Acc:HGNC:28685]	HGLibA_33250;HGLibA_33249
ENSG00000162669	HFM1	2	-2.9086	-3.5265	0.034668	0.85011	0.00042108	0.059547	ATP dependent DNA helicase homolog [Source:HGNC Symbol;Acc:HGNC:20193]"	HGLibA_21230;HGLibA_21231
ENSG00000137776	SLTM	2	-3.2607	-4.0503	0.025852	0.84371	5.11E-05	0.01224	SAFB like transcription modulator [Source:HGNC Symbol;Acc:HGNC:20709]	HGLibA_45548;HGLibA_45549
ENSG00000137955	RABGGTB	2	-2.6671	-3.0362	0.041693	0.87558	0.0023955	0.21737	Rab geranylgeranyltransferase subunit beta [Source:HGNC Symbol;Acc:HGNC:9796]	HGLibA_40020;HGLibA_40022
ENSG00000126261	UBA2	1	-3.534	-2.6091	0.018872	0.84371	0.0090784	0.48577	ubiquitin like modifier activating enzyme 2 [Source:HGNC Symbol;Acc:HGNC:30661]	HGLibA_52517
ENSG00000242028	HYPK	1	-3.69	-2.698	0.014969	0.84371	0.006976	0.41852	huntingtin interacting protein K [Source:HGNC Symbol;Acc:HGNC:18418]	HGLibA_22463
ENSG00000074582	BCS1L	3	-2.854	-1.5334	0.036183	0.8559	0.12517	0.95153	ubiquinol-cytochrome c reductase complex chaperone [Source:HGNC Symbol;Acc:HGNC:1020]"	HGLibA_04372;HGLibA_04370;HGLibA_04371
ENSG00000188694	KRTAP24-1	2	-3.5852	-3.7612	0.017036	0.84371	0.00016913	0.027905	keratin associated protein 24-1 [Source:HGNC Symbol;Acc:HGNC:33902]	HGLibA_25726;HGLibA_25725
ENSG00000086475	SEPHS1	3	-2.5293	-3.9072	0.045275	0.87787	9.34E-05	0.019183	selenophosphate synthetase 1 [Source:HGNC Symbol;Acc:HGNC:19685]	HGLibA_43412;HGLibA_43411;HGLibA_43413
ENSG00000131469	RPL27	2	-3.0743	-1.7436	0.030444	0.84371	0.081221	0.95153	ribosomal protein L27 [Source:HGNC Symbol;Acc:HGNC:10328]	HGLibA_41918;HGLibA_41920
hsa-mir-1206	hsa-mir-1206	1	-3.8315	-2.807	0.011709	0.84371	0.0050007	0.33406	-	HGLibA_57280
ENSG00000132646	PCNA	2	-2.8086	-3.3643	0.037102	0.85956	0.00076729	0.094944	proliferating cell nuclear antigen [Source:HGNC Symbol;Acc:HGNC:8729]	HGLibA_35635;HGLibA_35633
ENSG00000115816	CEBPZ	3	-3.0168	-3.9782	0.031959	0.84371	6.94E-05	0.014971	CCAAT enhancer binding protein zeta [Source:HGNC Symbol;Acc:HGNC:24218]	HGLibA_08971;HGLibA_08972;HGLibA_08970
ENSG00000128731	HERC2	2	-2.6367	-3.0612	0.042244	0.87558	0.0022042	0.20387	HECT and RLD domain containing E3 ubiquitin protein ligase 2 [Source:HGNC Symbol;Acc:HGNC:4868]	HGLibA_21155;HGLibA_21157
ENSG00000100027	YPEL1	1	-3.534	-2.6091	0.018872	0.84371	0.0090784	0.48577	yippee like 1 [Source:HGNC Symbol;Acc:HGNC:12845]	HGLibA_54705
ENSG00000166741	NNMT	2	-4.1139	-2.3491	0.0062448	0.79902	0.01882	0.79277	nicotinamide N-methyltransferase [Source:HGNC Symbol;Acc:HGNC:7861]	HGLibA_32223;HGLibA_32222
ENSG00000241370	RPP21	1	-3.1068	-2.331	0.029663	0.84371	0.019753	0.80279	ribonuclease P/MRP subunit p21 [Source:HGNC Symbol;Acc:HGNC:21300]	HGLibA_42026
ENSG00000163743	RCHY1	3	-3.8123	-2.0099	0.012352	0.84371	0.044447	0.95153	ring finger and CHY zinc finger domain containing 1 [Source:HGNC Symbol;Acc:HGNC:17479]	HGLibA_40694;HGLibA_40695;HGLibA_40696
ENSG00000189403	HMGB1	3	-3.0297	-2.3684	0.03127	0.84371	0.017866	0.76291	high mobility group box 1 [Source:HGNC Symbol;Acc:HGNC:4983]	HGLibA_21691;HGLibA_21689;HGLibA_21690
ENSG00000152464	RPP38	3	-2.9992	-1.4498	0.032234	0.84371	0.14712	0.95153	ribonuclease P/MRP subunit p38 [Source:HGNC Symbol;Acc:HGNC:30329]	HGLibA_42038;HGLibA_42037;HGLibA_42039
ENSG00000101546	RBFA	2	-3.7334	-4.7227	0.013959	0.84371	2.33E-06	0.0010977	ribosome binding factor A [Source:HGNC Symbol;Acc:HGNC:26120]	HGLibA_40451;HGLibA_40453
IARS	IARS	1	-2.5612	-1.9891	0.044081	0.87787	0.046694	0.95153	-	HGLibA_22472
ENSG00000116120	FARSB	1	-2.895	-2.1963	0.034898	0.85011	0.028072	0.95153	phenylalanyl-tRNA synthetase subunit beta [Source:HGNC Symbol;Acc:HGNC:17800]	HGLibA_16887
ENSG00000081803	CADPS2	2	-2.6409	-2.8327	0.042153	0.87558	0.0046159	0.32114	calcium dependent secretion activator 2 [Source:HGNC Symbol;Acc:HGNC:16018]	HGLibA_06981;HGLibA_06979
ENSG00000226887	ERVMER34-1	2	-2.7455	-0.66352	0.038847	0.86735	0.50699	0.95153	envelope [Source:HGNC Symbol;Acc:HGNC:42970]"	HGLibA_15541;HGLibA_15543
ENSG00000108826	MRPL27	2	-2.5699	-2.4767	0.043806	0.87764	0.01326	0.63051	mitochondrial ribosomal protein L27 [Source:HGNC Symbol;Acc:HGNC:14483]	HGLibA_29939;HGLibA_29940
ENSG00000149136	SSRP1	3	-3.9045	-3.9804	0.010332	0.84371	6.88E-05	0.014971	structure specific recognition protein 1 [Source:HGNC Symbol;Acc:HGNC:11327]	HGLibA_47164;HGLibA_47166;HGLibA_47165
HIST1H2BH	HIST1H2BH	3	-2.4349	-0.8624	0.048076	0.87787	0.38847	0.95153	-	HGLibA_21431;HGLibA_21432;HGLibA_21433
HIST1H2BI	HIST1H2BI	3	-2.8603	-1.3524	0.036046	0.8559	0.17626	0.95153	-	HGLibA_21435;HGLibA_21434;HGLibA_21436
ENSG00000129933	MAU2	2	-3.1413	-3.958	0.029158	0.84371	7.56E-05	0.015978	MAU2 sister chromatid cohesion factor [Source:HGNC Symbol;Acc:HGNC:29140]	HGLibA_28442;HGLibA_28441
hsa-mir-4679-2	hsa-mir-4679-2	1	-3.0679	-2.3061	0.030489	0.84371	0.021105	0.83392	-	HGLibA_60832
hsa-mir-4679-1	hsa-mir-4679-1	1	-2.895	-2.1963	0.034898	0.85011	0.028072	0.95153	-	HGLibA_60831
ENSG00000164934	DCAF13	2	-2.5686	-2.1816	0.043806	0.87764	0.029138	0.95153	DDB1 and CUL4 associated factor 13 [Source:HGNC Symbol;Acc:HGNC:24535]	HGLibA_12427;HGLibA_12428
hsa-mir-5096	hsa-mir-5096	2	-2.9786	-3.8401	0.032877	0.84371	0.00012298	0.022507	-	HGLibA_61543;HGLibA_61541
ENSG00000147679	UTP23	3	-3.1786	-1.5145	0.028331	0.84371	0.1299	0.95153	small subunit processome component [Source:HGNC Symbol;Acc:HGNC:28224]"	HGLibA_53450;HGLibA_53448;HGLibA_53449
ENSG00000163902	RPN1	3	-2.9618	-1.6539	0.032969	0.84371	0.098139	0.95153	ribophorin I [Source:HGNC Symbol;Acc:HGNC:10381]	HGLibA_42018;HGLibA_42017;HGLibA_42016
ENSG00000187688	TRPV2	1	-3.4831	-2.5756	0.020296	0.84371	0.010007	0.52324	transient receptor potential cation channel subfamily V member 2 [Source:HGNC Symbol;Acc:HGNC:18082]	HGLibA_51784
hsa-mir-4442	hsa-mir-4442	3	-4.1084	-5.8911	0.0062448	0.79902	3.84E-09	6.43E-06	-	HGLibA_60169;HGLibA_60172;HGLibA_60171
ENSG00000165280	VCP	2	-3.4428	-3.4112	0.021627	0.84371	0.00064687	0.083357	valosin containing protein [Source:HGNC Symbol;Acc:HGNC:12666]	HGLibA_53576;HGLibA_53574
hsa-mir-1254-1	hsa-mir-1254-1	2	-3.8227	-2.3257	0.011939	0.84371	0.020033	0.81024	-	HGLibA_57413;HGLibA_57415
ENSG00000064547	LPAR2	2	-2.583	-0.41644	0.043484	0.87616	0.67709	0.95153	lysophosphatidic acid receptor 2 [Source:HGNC Symbol;Acc:HGNC:3168]	HGLibA_27057;HGLibA_27056
ENSG00000178691	SUZ12	3	-2.7559	-1.3233	0.038663	0.86735	0.18574	0.95153	polycomb repressive complex 2 subunit [Source:HGNC Symbol;Acc:HGNC:17101]"	HGLibA_47871;HGLibA_47870;HGLibA_47872
ENSG00000106330	MOSPD3	2	-3.0805	-0.84913	0.030352	0.84371	0.39581	0.95153	motile sperm domain containing 3 [Source:HGNC Symbol;Acc:HGNC:25078]	HGLibA_29691;HGLibA_29690
ENSG00000183160	TMEM119	2	-2.4472	-3.1302	0.047938	0.87787	0.001747	0.17614	transmembrane protein 119 [Source:HGNC Symbol;Acc:HGNC:27884]	HGLibA_49892;HGLibA_49891
ENSG00000118482	PHF3	1	-3.534	-2.6091	0.018872	0.84371	0.0090784	0.48577	PHD finger protein 3 [Source:HGNC Symbol;Acc:HGNC:8921]	HGLibA_36377
ENSG00000186417	GLDN	2	-4.3426	-5.4368	0.0029847	0.784	5.43E-08	6.22E-05	gliomedin [Source:HGNC Symbol;Acc:HGNC:29514]	HGLibA_19237;HGLibA_19238
ENSG00000181555	SETD2	1	-2.7421	-2.1005	0.03903	0.86735	0.035682	0.95153	SET domain containing 2 [Source:HGNC Symbol;Acc:HGNC:18420]	HGLibA_43643
ENSG00000204264	PSMB8	1	-4.7613	-3.4414	0.00073469	0.6129	0.00057863	0.075458	proteasome subunit beta 8 [Source:HGNC Symbol;Acc:HGNC:9545]	HGLibA_39121
ENSG00000158526	TSR2	2	-3.7103	-4.185	0.014556	0.84371	2.85E-05	0.0076674	ribosome maturation factor [Source:HGNC Symbol;Acc:HGNC:25455]"	HGLibA_51998;HGLibA_51997
ENSG00000279012	OR51B2	2	-3.1502	-1.4643	0.028836	0.84371	0.14312	0.95153	olfactory receptor family 51 subfamily B member 2 (gene/pseudogene) [Source:HGNC Symbol;Acc:HGNC:14703]	HGLibA_34081;HGLibA_34083
ENSG00000030304	MUSK	3	-2.8698	-1.9105	0.035862	0.8559	0.056074	0.95153	muscle associated receptor tyrosine kinase [Source:HGNC Symbol;Acc:HGNC:7525]	HGLibA_30578;HGLibA_30576;HGLibA_30577
ENSG00000219481	NBPF1	2	-2.6896	-2.7082	0.040637	0.87097	0.0067643	0.4092	NBPF member 1 [Source:HGNC Symbol;Acc:HGNC:26088]	HGLibA_31222;HGLibA_31220
ENSG00000048544	MRPS10	2	-3.4322	-4.2107	0.021765	0.84371	2.55E-05	0.0071076	mitochondrial ribosomal protein S10 [Source:HGNC Symbol;Acc:HGNC:14502]	HGLibA_30030;HGLibA_30028
ENSG00000066032	CTNNA2	3	-3.1339	-1.4026	0.02925	0.84371	0.16075	0.95153	catenin alpha 2 [Source:HGNC Symbol;Acc:HGNC:2510]	HGLibA_11641;HGLibA_11642;HGLibA_11640
hsa-mir-4467	hsa-mir-4467	2	-4.2582	-4.7103	0.0037653	0.784	2.47E-06	0.0010977	-	HGLibA_60275;HGLibA_60273
ENSG00000124571	XPO5	1	-2.9852	-2.2534	0.032464	0.84371	0.024235	0.91789	exportin 5 [Source:HGNC Symbol;Acc:HGNC:17675]	HGLibA_54567
ENSG00000198743	SLC5A3	2	-2.7188	-0.83364	0.039994	0.87097	0.40448	0.95153	solute carrier family 5 member 3 [Source:HGNC Symbol;Acc:HGNC:11038]	HGLibA_45269;HGLibA_45267
ENSG00000101040	ZMYND8	2	-3.0411	-3.5452	0.03104	0.84371	0.00039227	0.056575	zinc finger MYND-type containing 8 [Source:HGNC Symbol;Acc:HGNC:9397]	HGLibA_55426;HGLibA_55427
ENSG00000170448	NFXL1	3	-2.5653	-0.57228	0.043989	0.87787	0.56713	0.95153	X-box binding like 1 [Source:HGNC Symbol;Acc:HGNC:18726]"	HGLibA_31851;HGLibA_31852;HGLibA_31850
hsa-mir-6859-2	hsa-mir-6859-2	1	-2.4927	-1.9474	0.046469	0.87787	0.051484	0.95153	-	HGLibA_63612
ENSG00000164638	SLC29A4	2	-3.9015	-4.1346	0.010653	0.84371	3.56E-05	0.0091874	solute carrier family 29 member 4 [Source:HGNC Symbol;Acc:HGNC:23097]	HGLibA_44848;HGLibA_44849
ENSG00000076108	BAZ2A	3	-3.2855	-0.44187	0.025347	0.84371	0.65858	0.95153	bromodomain adjacent to zinc finger domain 2A [Source:HGNC Symbol;Acc:HGNC:962]	HGLibA_04191;HGLibA_04192;HGLibA_04190
ENSG00000108604	SMARCD2	2	-3.2236	-1.1017	0.026908	0.84371	0.27057	0.95153	member 2 [Source:HGNC Symbol;Acc:HGNC:11107]"	HGLibA_45633;HGLibA_45631
ENSG00000138398	PPIG	2	-4.1573	-5.127	0.0052806	0.79902	2.94E-07	0.00020679	peptidylprolyl isomerase G [Source:HGNC Symbol;Acc:HGNC:14650]	HGLibA_37933;HGLibA_37934
ENSG00000205323	SARNP	3	-2.8849	-3.6387	0.035403	0.85525	0.00027397	0.042424	SAP domain containing ribonucleoprotein [Source:HGNC Symbol;Acc:HGNC:24432]	HGLibA_42745;HGLibA_42747;HGLibA_42746
ENSG00000166592	RRAD	2	-2.4245	-2.1055	0.048443	0.87787	0.035248	0.95153	Ras related glycolysis inhibitor and calcium channel regulator [Source:HGNC Symbol;Acc:HGNC:10446]"	HGLibA_42221;HGLibA_42222
hsa-mir-3134	hsa-mir-3134	2	-3.1156	-3.8094	0.029525	0.84371	0.00013931	0.024667	-	HGLibA_58592;HGLibA_58589
ENSG00000125977	EIF2S2	3	-3.1614	-3.2738	0.028699	0.84371	0.0010611	0.12162	eukaryotic translation initiation factor 2 subunit beta [Source:HGNC Symbol;Acc:HGNC:3266]	HGLibA_14756;HGLibA_14757;HGLibA_14755
ENSG00000161016	RPL8	2	-2.883	-3.1606	0.035449	0.85525	0.0015743	0.16326	ribosomal protein L8 [Source:HGNC Symbol;Acc:HGNC:10368]	HGLibA_42001;HGLibA_42002
ENSG00000197901	SLC22A6	2	-2.919	-0.84208	0.034484	0.85011	0.39974	0.95153	solute carrier family 22 member 6 [Source:HGNC Symbol;Acc:HGNC:10970]	HGLibA_44594;HGLibA_44595
ENSG00000058804	NDC1	2	-2.9323	-3.6082	0.034071	0.85011	0.00030829	0.046303	NDC1 transmembrane nucleoporin [Source:HGNC Symbol;Acc:HGNC:25525]	HGLibA_31380;HGLibA_31381
ENSG00000075303	SLC25A40	2	-3.39	-4.335	0.022959	0.84371	1.46E-05	0.0045347	solute carrier family 25 member 40 [Source:HGNC Symbol;Acc:HGNC:29680]	HGLibA_44735;HGLibA_44736
NUPL2	NUPL2	3	-3.6445	-1.555	0.015658	0.84371	0.11995	0.95153	-	HGLibA_33084;HGLibA_33083;HGLibA_33085
ENSG00000142168	SOD1	2	-3.1338	-2.1474	0.02925	0.84371	0.031761	0.95153	superoxide dismutase 1 [Source:HGNC Symbol;Acc:HGNC:11179]	HGLibA_46144;HGLibA_46143
ENSG00000129534	MIS18BP1	3	-3.4962	-5.2058	0.019974	0.84371	1.93E-07	0.00015579	MIS18 binding protein 1 [Source:HGNC Symbol;Acc:HGNC:20190]	HGLibA_29308;HGLibA_29310;HGLibA_29309
ENSG00000067704	IARS2	3	-2.4259	-1.3941	0.048443	0.87787	0.16329	0.95153	mitochondrial [Source:HGNC Symbol;Acc:HGNC:29685]"	HGLibA_22475;HGLibA_22476;HGLibA_22477
ENSG00000104885	DOT1L	2	-3.9304	-4.9571	0.0098723	0.84371	7.16E-07	0.00043287	DOT1 like histone lysine methyltransferase [Source:HGNC Symbol;Acc:HGNC:24948]	HGLibA_13805;HGLibA_13807
ENSG00000023330	ALAS1	1	-2.6853	-2.0654	0.040913	0.87097	0.038888	0.95153	5'-aminolevulinate synthase 1 [Source:HGNC Symbol;Acc:HGNC:396]	HGLibA_01522
ENSG00000130175	PRKCSH	3	-4.2846	-5.3419	0.0035357	0.784	9.20E-08	9.33E-05	protein kinase C substrate 80K-H [Source:HGNC Symbol;Acc:HGNC:9411]	HGLibA_38561;HGLibA_38560;HGLibA_38559
ENSG00000146067	FAM193B	3	-3.0419	-2.1548	0.03104	0.84371	0.031179	0.95153	family with sequence similarity 193 member B [Source:HGNC Symbol;Acc:HGNC:25524]	HGLibA_16367;HGLibA_16366;HGLibA_16365
ENSG00000104980	TIMM44	2	-2.6453	-2.6331	0.042107	0.87558	0.0084613	0.47008	translocase of inner mitochondrial membrane 44 [Source:HGNC Symbol;Acc:HGNC:17316]	HGLibA_49517;HGLibA_49518
ENSG00000169484	OR4K14	3	-2.9836	-1.8041	0.032739	0.84371	0.07121	0.95153	olfactory receptor family 4 subfamily K member 14 [Source:HGNC Symbol;Acc:HGNC:15352]	HGLibA_34021;HGLibA_34022;HGLibA_34023
ENSG00000075624	ACTB	2	-3.083	-3.8555	0.030306	0.84371	0.0001155	0.021989	actin beta [Source:HGNC Symbol;Acc:HGNC:132]	HGLibA_00602;HGLibA_00601
ENSG00000168286	THAP11	1	-3.8315	-2.807	0.011709	0.84371	0.0050007	0.33406	THAP domain containing 11 [Source:HGNC Symbol;Acc:HGNC:23194]	HGLibA_49292
hsa-mir-4719	hsa-mir-4719	1	-4.4322	-3.2146	0.0023877	0.76812	0.0013061	0.14091	-	HGLibA_60975
ENSG00000147162	OGT	1	-3.3432	-2.4839	0.024153	0.84371	0.012994	0.62056	O-linked N-acetylglucosamine (GlcNAc) transferase [Source:HGNC Symbol;Acc:HGNC:8127]	HGLibA_33329
ENSG00000165733	BMS1	1	-3.4567	-2.5582	0.021122	0.84371	0.010522	0.53915	ribosome biogenesis factor [Source:HGNC Symbol;Acc:HGNC:23505]"	HGLibA_04638
ENSG00000115685	PPP1R7	1	-3.5584	-2.6253	0.01777	0.84371	0.0086585	0.47023	protein phosphatase 1 regulatory subunit 7 [Source:HGNC Symbol;Acc:HGNC:9295]	HGLibA_38122
ENSG00000214290	COLCA2	2	-3.9146	-2.9614	0.010194	0.84371	0.0030628	0.25207	colorectal cancer associated 2 [Source:HGNC Symbol;Acc:HGNC:26978]	HGLibA_10630;HGLibA_10628
ENSG00000138378	STAT4	3	-3.6788	-4.0672	0.015153	0.84371	4.76E-05	0.011513	signal transducer and activator of transcription 4 [Source:HGNC Symbol;Acc:HGNC:11365]	HGLibA_47391;HGLibA_47390;HGLibA_47389
ENSG00000116560	SFPQ	1	-2.4927	-1.9474	0.046469	0.87787	0.051484	0.95153	splicing factor proline and glutamine rich [Source:HGNC Symbol;Acc:HGNC:10774]	HGLibA_43731
ENSG00000182472	CAPN12	2	-4.9226	-6.1027	0.00041326	0.5625	1.04E-09	2.52E-06	calpain 12 [Source:HGNC Symbol;Acc:HGNC:13249]	HGLibA_07151;HGLibA_07150
hsa-mir-548aj-2	hsa-mir-548aj-2	3	-2.8443	-2.1617	0.036459	0.85745	0.030645	0.95153	-	HGLibA_61892;HGLibA_61893;HGLibA_61891
ENSG00000151005	TKTL2	2	-2.8975	-3.0805	0.034898	0.85011	0.0020668	0.19602	transketolase like 2 [Source:HGNC Symbol;Acc:HGNC:25313]	HGLibA_49593;HGLibA_49592
ENSG00000198783	ZNF830	2	-2.8845	-1.5089	0.035403	0.85525	0.13132	0.95153	zinc finger protein 830 [Source:HGNC Symbol;Acc:HGNC:28291]	HGLibA_56833;HGLibA_56834
ENSG00000253729	PRKDC	2	-3.5966	-3.6359	0.01676	0.84371	0.00027698	0.042479	catalytic subunit [Source:HGNC Symbol;Acc:HGNC:9413]"	HGLibA_38576;HGLibA_38574
ENSG00000142676	RPL11	2	-2.6994	-3.5686	0.040362	0.87097	0.00035888	0.052104	ribosomal protein L11 [Source:HGNC Symbol;Acc:HGNC:10301]	HGLibA_41861;HGLibA_41863
ENSG00000152620	NADK2	1	-3.3728	-2.5033	0.023326	0.84371	0.012305	0.59552	mitochondrial [Source:HGNC Symbol;Acc:HGNC:26404]"	HGLibA_31048
ENSG00000183808	RBM12B	3	-4.2316	-6.5359	0.0044081	0.784	6.32E-11	4.31E-07	RNA binding motif protein 12B [Source:HGNC Symbol;Acc:HGNC:32310]	HGLibA_40482;HGLibA_40481;HGLibA_40483
ENSG00000174007	CEP19	2	-3.0302	-2.9342	0.03127	0.84371	0.0033445	0.2639	centrosomal protein 19 [Source:HGNC Symbol;Acc:HGNC:28209]	HGLibA_09123;HGLibA_09121
ENSG00000176387	HSD11B2	1	-2.6853	-2.0654	0.040913	0.87097	0.038888	0.95153	hydroxysteroid 11-beta dehydrogenase 2 [Source:HGNC Symbol;Acc:HGNC:5209]	HGLibA_22167
ENSG00000176386	CDC26	2	-3.0006	-3.8986	0.032188	0.84371	9.67E-05	0.019341	cell division cycle 26 [Source:HGNC Symbol;Acc:HGNC:17839]	HGLibA_08536;HGLibA_08537
hsa-mir-4469	hsa-mir-4469	2	-3.3003	-1.9016	0.025117	0.84371	0.05723	0.95153	-	HGLibA_60281;HGLibA_60284
ENSG00000127837	AAMP	2	-2.5059	-3.3603	0.045964	0.87787	0.00077858	0.095796	angio associated migratory cell protein [Source:HGNC Symbol;Acc:HGNC:18]	HGLibA_00056;HGLibA_00057
hsa-mir-3668	hsa-mir-3668	1	-3.3728	-2.5033	0.023326	0.84371	0.012305	0.59552	-	HGLibA_59222
ENSG00000121406	ZNF549	2	-3.7425	-4.4506	0.013592	0.84371	8.56E-06	0.0028692	zinc finger protein 549 [Source:HGNC Symbol;Acc:HGNC:26632]	HGLibA_56209;HGLibA_56210
ENSG00000119915	ELOVL3	3	-2.5409	-1.7099	0.044908	0.87787	0.087282	0.95153	ELOVL fatty acid elongase 3 [Source:HGNC Symbol;Acc:HGNC:18047]	HGLibA_14962;HGLibA_14963;HGLibA_14964
ENSG00000107164	FUBP3	2	-3.2256	-1.7873	0.026862	0.84371	0.073894	0.95153	far upstream element binding protein 3 [Source:HGNC Symbol;Acc:HGNC:4005]	HGLibA_18153;HGLibA_18151
ENSG00000084070	SMAP2	2	-3.3589	-4.0132	0.023694	0.84371	6.04E-05	0.013703	small ArfGAP2 [Source:HGNC Symbol;Acc:HGNC:25082]	HGLibA_45598;HGLibA_45600
ENSG00000143443	C1orf56	2	-3.6284	-2.7591	0.015934	0.84371	0.0057962	0.37018	chromosome 1 open reading frame 56 [Source:HGNC Symbol;Acc:HGNC:26045]	HGLibA_05877;HGLibA_05875
ENSG00000159445	THEM4	3	-2.5935	-1.3675	0.043301	0.87558	0.17147	0.95153	thioesterase superfamily member 4 [Source:HGNC Symbol;Acc:HGNC:17947]	HGLibA_49340;HGLibA_49339;HGLibA_49341
ENSG00000185442	FAM174B	2	-2.9586	-0.84399	0.033153	0.84543	0.39867	0.95153	family with sequence similarity 174 member B [Source:HGNC Symbol;Acc:HGNC:34339]	HGLibA_16286;HGLibA_16285
LARS	LARS	1	-2.6853	-2.0654	0.040913	0.87097	0.038888	0.95153	-	HGLibA_26045
ENSG00000136021	SCYL2	2	-2.8784	-1.0032	0.03554	0.85525	0.31576	0.95153	SCY1 like pseudokinase 2 [Source:HGNC Symbol;Acc:HGNC:19286]	HGLibA_43087;HGLibA_43089
ENSG00000172689	MS4A10	2	-4.0034	-2.2999	0.0078979	0.82692	0.021451	0.84229	membrane spanning 4-domains A10 [Source:HGNC Symbol;Acc:HGNC:13368]	HGLibA_30129;HGLibA_30130
ENSG00000118260	CREB1	2	-2.6385	-3.4096	0.042153	0.87558	0.00065069	0.083357	cAMP responsive element binding protein 1 [Source:HGNC Symbol;Acc:HGNC:2345]	HGLibA_11087;HGLibA_11088
ENSG00000116580	GON4L	3	-3.0024	-3.5747	0.032188	0.84371	0.00035068	0.051256	gon-4 like [Source:HGNC Symbol;Acc:HGNC:25973]	HGLibA_19668;HGLibA_19670;HGLibA_19669
FAM65A	FAM65A	3	-4.2789	-5.5695	0.0036734	0.784	2.56E-08	3.09E-05	-	HGLibA_16667;HGLibA_16669;HGLibA_16668
ENSG00000113558	SKP1	1	-2.4927	-1.9474	0.046469	0.87787	0.051484	0.95153	S-phase kinase associated protein 1 [Source:HGNC Symbol;Acc:HGNC:10899]	HGLibA_44295
ENSG00000075188	NUP37	3	-2.5517	-1.8447	0.04477	0.87787	0.065082	0.95153	nucleoporin 37 [Source:HGNC Symbol;Acc:HGNC:29929]	HGLibA_33051;HGLibA_33052;HGLibA_33050
ENSG00000198502	HLA-DRB5	2	-4.2295	-5.3043	0.0044081	0.784	1.13E-07	0.00010261	DR beta 5 [Source:HGNC Symbol;Acc:HGNC:4953]"	HGLibA_21639;HGLibA_21640
ENSG00000183251	OR51B4	2	-2.4409	-0.40783	0.047938	0.87787	0.6834	0.95153	olfactory receptor family 51 subfamily B member 4 [Source:HGNC Symbol;Acc:HGNC:14708]	HGLibA_34084;HGLibA_34085
ENSG00000128563	PRKRIP1	2	-4.1142	-4.6712	0.0062448	0.79902	2.99E-06	0.0012305	PRKR interacting protein 1 [Source:HGNC Symbol;Acc:HGNC:21894]	HGLibA_38588;HGLibA_38587
C16orf62	C16orf62	2	-3.226	-2.8413	0.026862	0.84371	0.0044929	0.31861	-	HGLibA_05469;HGLibA_05467
ENSG00000214021	TTLL3	2	-2.7649	-0.56256	0.038525	0.86735	0.57373	0.95153	tubulin tyrosine ligase like 3 [Source:HGNC Symbol;Acc:HGNC:24483]	HGLibA_52202;HGLibA_52201
ENSG00000065154	OAT	2	-4.1675	-4.2321	0.0052806	0.79902	2.32E-05	0.0066342	ornithine aminotransferase [Source:HGNC Symbol;Acc:HGNC:8091]	HGLibA_33211;HGLibA_33210

ENSG00000139263	LRIG3	3	-4.074	-5.7401	0.0069336	0.79902	9.46E-09	1.47E-05	leucine rich repeats and immunoglobulin like domains 3 [Source:HGNC Symbol;Acc:HGNC:30991]	HGLibA_27182;HGLibA_27181;HGLibA_27183
hsa-mir-4759	hsa-mir-4759	3	-2.8464	-3.7864	0.036413	0.8573	0.00015283	0.025603	-	HGLibA_61132;HGLibA_61134;HGLibA_61133
ENSG00000090924	PLEKHG2	3	-3.7603	-3.1662	0.01327	0.84371	0.0015444	0.16093	pleckstrin homology and RhoGEF domain containing G2 [Source:HGNC Symbol;Acc:HGNC:29515]	HGLibA_37104;HGLibA_37103;HGLibA_37105
ENSG00000115020	PIKFYVE	1	-3.2151	-2.4007	0.027046	0.84371	0.016362	0.71696	FYVE-type zinc finger containing [Source:HGNC Symbol;Acc:HGNC:23785]"	HGLibA_36667
ENSG00000153914	SREK1	3	-3.9218	-6.1234	0.0098723	0.84371	9.16E-10	2.49E-06	splicing regulatory glutamic acid and lysine rich protein 1 [Source:HGNC Symbol;Acc:HGNC:17882]	HGLibA_46956;HGLibA_46955;HGLibA_46954
AHSA2	AHSA2	3	-2.4272	-2.5175	0.048397	0.87787	0.011819	0.58367	-	HGLibA_01316;HGLibA_01315;HGLibA_01317
ENSG00000213782	DDX47	1	-2.7421	-2.1005	0.03903	0.86735	0.035682	0.95153	DEAD-box helicase 47 [Source:HGNC Symbol;Acc:HGNC:18682]	HGLibA_12724
ENSG00000155903	RASA2	3	-3.4608	-2.3309	0.021076	0.84371	0.019758	0.80279	RAS p21 protein activator 2 [Source:HGNC Symbol;Acc:HGNC:9872]	HGLibA_40300;HGLibA_40299;HGLibA_40298
ENSG00000143324	XPR1	2	-2.4198	-2.8124	0.048627	0.87787	0.0049171	0.33406	xenotropic and polytropic retrovirus receptor 1 [Source:HGNC Symbol;Acc:HGNC:12827]	HGLibA_54579;HGLibA_54580
ENSG00000188612	SUMO2	2	-3.369	-2.7656	0.023464	0.84371	0.0056825	0.36398	small ubiquitin-like modifier 2 [Source:HGNC Symbol;Acc:HGNC:11125]	HGLibA_47778;HGLibA_47780
ENSG00000146457	WTAP	2	-3.3585	-1.3129	0.023694	0.84371	0.18921	0.95153	WT1 associated protein [Source:HGNC Symbol;Acc:HGNC:16846]	HGLibA_54431;HGLibA_54430
ENSG00000186141	POLR3C	2	-3.8406	-4.6496	0.011663	0.84371	3.33E-06	0.0013166	RNA polymerase III subunit C [Source:HGNC Symbol;Acc:HGNC:30076]	HGLibA_37614;HGLibA_37616
ENSG00000151366	NDUFC2	3	-3.3296	-1.7148	0.024474	0.84371	0.086389	0.95153	NADH:ubiquinone oxidoreductase subunit C2 [Source:HGNC Symbol;Acc:HGNC:7706]	HGLibA_31539;HGLibA_31538;HGLibA_31540
hsa-mir-92a-1	hsa-mir-92a-1	2	-2.3998	-1.2467	0.049454	0.87787	0.21252	0.95153	-	HGLibA_64291;HGLibA_64290
ENSG00000104897	SF3A2	2	-3.7437	-4.7202	0.013592	0.84371	2.36E-06	0.0010977	splicing factor 3a subunit 2 [Source:HGNC Symbol;Acc:HGNC:10766]	HGLibA_43695;HGLibA_43694
ENSG00000180219	FAM71C	3	-2.7263	-1.4696	0.039765	0.87097	0.14167	0.95153	family with sequence similarity 71 member C [Source:HGNC Symbol;Acc:HGNC:28594]	HGLibA_16691;HGLibA_16692;HGLibA_16693
ENSG00000062822	POLD1	1	-2.895	-2.1963	0.034898	0.85011	0.028072	0.95153	catalytic subunit [Source:HGNC Symbol;Acc:HGNC:9175]"	HGLibA_37492
ENSG00000152684	PELO	3	-3.469	-4.8086	0.02103	0.84371	1.52E-06	0.0008073	pelota mRNA surveillance and ribosome rescue factor [Source:HGNC Symbol;Acc:HGNC:8829]	HGLibA_36042;HGLibA_36041;HGLibA_36043
ENSG00000162734	PEA15	1	-2.7421	-2.1005	0.03903	0.86735	0.035682	0.95153	proliferation and apoptosis adaptor protein 15 [Source:HGNC Symbol;Acc:HGNC:8822]	HGLibA_36002
ENSG00000090061	CCNK	2	-2.4686	-3.1439	0.047525	0.87787	0.0016669	0.17044	cyclin K [Source:HGNC Symbol;Acc:HGNC:1596]	HGLibA_08113;HGLibA_08114
FAM105B	FAM105B	1	-2.9789	-2.2404	0.032785	0.84371	0.025062	0.93619	-	HGLibA_15997
ENSG00000164587	RPS14	2	-3.5642	-4.5306	0.017724	0.84371	5.88E-06	0.0020662	ribosomal protein S14 [Source:HGNC Symbol;Acc:HGNC:10387]	HGLibA_42074;HGLibA_42075
GCN1L1	GCN1L1	2	-3.414	-3.1791	0.022362	0.84371	0.0014774	0.15619	-	HGLibA_18805;HGLibA_18807
ENSG00000159307	SCUBE1	2	-2.766	-0.83517	0.038525	0.86735	0.40362	0.95153	CUB domain and EGF like domain containing 1 [Source:HGNC Symbol;Acc:HGNC:13441]"	HGLibA_43069;HGLibA_43070
TAZ	TAZ	2	-3.4669	-1.9017	0.02103	0.84371	0.057211	0.95153	-	HGLibA_48456;HGLibA_48455
ENSG00000163029	SMC6	3	-2.7595	-4.2897	0.038571	0.86735	1.79E-05	0.0053384	structural maintenance of chromosomes 6 [Source:HGNC Symbol;Acc:HGNC:20466]	HGLibA_45660;HGLibA_45658;HGLibA_45659
ENSG00000179344	HLA-DQB1	3	-4.0708	-5.9198	0.0069336	0.79902	3.22E-09	5.85E-06	DQ beta 1 [Source:HGNC Symbol;Acc:HGNC:4944]"	HGLibA_21628;HGLibA_21626;HGLibA_21627
ENSG00000088038	CNOT3	2	-2.8342	-3.3478	0.036642	0.85944	0.00081463	0.099112	CCR4-NOT transcription complex subunit 3 [Source:HGNC Symbol;Acc:HGNC:7879]	HGLibA_10337;HGLibA_10339
ENSG00000137868	STRA6	3	-3.1417	-1.3208	0.029158	0.84371	0.18658	0.95153	stimulated by retinoic acid 6 [Source:HGNC Symbol;Acc:HGNC:30650]	HGLibA_47568;HGLibA_47566;HGLibA_47567
hsa-mir-4703	hsa-mir-4703	1	-2.8466	-2.1658	0.036229	0.8559	0.030324	0.95153	-	HGLibA_60915
ENSG00000114446	IFT57	1	-3.8754	-2.8213	0.010883	0.84371	0.0047823	0.32854	intraflagellar transport 57 [Source:HGNC Symbol;Acc:HGNC:17367]	HGLibA_22766
ENSG00000111247	RAD51AP1	3	-2.9762	-1.8112	0.032923	0.84371	0.070107	0.95153	RAD51 associated protein 1 [Source:HGNC Symbol;Acc:HGNC:16956]	HGLibA_40082;HGLibA_40081;HGLibA_40080
C19orf26	C19orf26	1	-3.4567	-2.5582	0.021122	0.84371	0.010522	0.53915	-	HGLibA_05646
ENSG00000104129	DNAJC17	2	-2.557	-0.83297	0.044632	0.87787	0.40486	0.95153	DnaJ heat shock protein family (Hsp40) member C17 [Source:HGNC Symbol;Acc:HGNC:25556]	HGLibA_13584;HGLibA_13585
ENSG00000235109	ZSCAN31	3	-2.599	-3.3066	0.043209	0.87558	0.00094424	0.11115	zinc finger and SCAN domain containing 31 [Source:HGNC Symbol;Acc:HGNC:14097]	HGLibA_57020;HGLibA_57018;HGLibA_57019
ENSG00000053524	MCF2L2	2	-2.6336	-0.4026	0.042336	0.87558	0.68724	0.95153	MCF.2 cell line derived transforming sequence-like 2 [Source:HGNC Symbol;Acc:HGNC:30319]	HGLibA_28584;HGLibA_28582
hsa-mir-548ac	hsa-mir-548ac	4	-2.454	-3.9791	0.047846	0.87787	6.92E-05	0.014971	-	HGLibA_61861;HGLibA_61860;HGLibA_61862;HGLibA_61859
ENSG00000179796	LRRC3B	3	-2.5253	-0.51888	0.045596	0.87787	0.60385	0.95153	leucine rich repeat containing 3B [Source:HGNC Symbol;Acc:HGNC:28105]	HGLibA_27344;HGLibA_27343;HGLibA_27345
ENSG00000089220	PEBP1	1	-4.4221	-3.2077	0.0024336	0.76812	0.0013378	0.14282	phosphatidylethanolamine binding protein 1 [Source:HGNC Symbol;Acc:HGNC:8630]	HGLibA_36013
ENSG00000163106	HPGDS	3	-3.1511	-4.2913	0.028836	0.84371	1.78E-05	0.0053384	hematopoietic prostaglandin D synthase [Source:HGNC Symbol;Acc:HGNC:17890]	HGLibA_22033;HGLibA_22034;HGLibA_22035
ENSG00000167130	DOLPP1	3	-2.424	-0.70238	0.048443	0.87787	0.48244	0.95153	dolichyldiphosphatase 1 [Source:HGNC Symbol;Acc:HGNC:29565]	HGLibA_13792;HGLibA_13791;HGLibA_13790
ENSG00000168488	ATXN2L	3	-3.1046	-2.8687	0.029938	0.84371	0.0041213	0.29918	ataxin 2 like [Source:HGNC Symbol;Acc:HGNC:31326]	HGLibA_03839;HGLibA_03841;HGLibA_03840
ENSG00000112685	EXOC2	2	-2.6187	-2.2193	0.043071	0.87558	0.02647	0.95153	exocyst complex component 2 [Source:HGNC Symbol;Acc:HGNC:24968]	HGLibA_15735;HGLibA_15734
ENSG00000154529	CNTNAP3B	2	-3.0998	-3.2144	0.030076	0.84371	0.001307	0.14091	contactin associated protein like 3B [Source:HGNC Symbol;Acc:HGNC:32035]	HGLibA_10429;HGLibA_10428
ENSG00000145736	GTF2H2	2	-2.7272	-3.4003	0.039719	0.87097	0.0006731	0.085226	general transcription factor IIH subunit 2 [Source:HGNC Symbol;Acc:HGNC:4656]	HGLibA_20548;HGLibA_20547
ENSG00000120327	PCDHB14	3	-2.6152	-0.92867	0.043071	0.87558	0.35306	0.95153	protocadherin beta 14 [Source:HGNC Symbol;Acc:HGNC:8685]	HGLibA_35483;HGLibA_35485;HGLibA_35484
ENSG00000103365	GGA2	3	-3.3136	-1.5203	0.024612	0.84371	0.12845	0.95153	ARF binding protein 2 [Source:HGNC Symbol;Acc:HGNC:16064]"	HGLibA_18982;HGLibA_18984;HGLibA_18983
ENSG00000124802	EEF1E1	2	-3.4329	-4.3681	0.021765	0.84371	1.25E-05	0.0040131	eukaryotic translation elongation factor 1 epsilon 1 [Source:HGNC Symbol;Acc:HGNC:3212]	HGLibA_14502;HGLibA_14501
ENSG00000160818	GPATCH4	3	-2.7384	-2.9997	0.039627	0.87097	0.0027026	0.23478	G-patch domain containing 4 [Source:HGNC Symbol;Acc:HGNC:25982]	HGLibA_19747;HGLibA_19748;HGLibA_19746
ENSG00000125755	SYMPK	2	-3.5762	-4.4903	0.017541	0.84371	7.11E-06	0.0024198	symplekin [Source:HGNC Symbol;Acc:HGNC:22935]	HGLibA_47953;HGLibA_47951
ENSG00000132704	FCRL2	2	-2.606	-3.2227	0.043117	0.87558	0.0012697	0.13947	Fc receptor like 2 [Source:HGNC Symbol;Acc:HGNC:14875]	HGLibA_17228;HGLibA_17227
ENSG00000109079	TNFAIP1	2	-4.7666	-5.9428	0.00073469	0.6129	2.80E-09	5.55E-06	TNF alpha induced protein 1 [Source:HGNC Symbol;Acc:HGNC:11894]	HGLibA_50705;HGLibA_50707
LEPRE1	LEPRE1	1	-3.1068	-2.331	0.029663	0.84371	0.019753	0.80279	-	HGLibA_26314
LOC388813	LOC388813	2	-3.0297	-0.84704	0.03127	0.84371	0.39697	0.95153	-	HGLibA_26923;HGLibA_26924
ENSG00000203963	C1orf141	3	-3.5805	-5.3375	0.017495	0.84371	9.43E-08	9.33E-05	chromosome 1 open reading frame 141 [Source:HGNC Symbol;Acc:HGNC:32044]	HGLibA_05772;HGLibA_05770;HGLibA_05771
ENSG00000120049	KCNIP2	1	-4.0304	-2.9409	0.0073928	0.79902	0.0032726	0.26011	potassium voltage-gated channel interacting protein 2 [Source:HGNC Symbol;Acc:HGNC:15522]	HGLibA_24237
ENSG00000125251	FASTKD5	3	-3.113	-2.0138	0.029571	0.84371	0.044028	0.95153	FAST kinase domains 5 [Source:HGNC Symbol;Acc:HGNC:25790]	HGLibA_16910;HGLibA_16909;HGLibA_16911
ENSG00000172809	RPL38	2	-3.5195	-2.6503	0.019469	0.84371	0.0080417	0.45489	ribosomal protein L38 [Source:HGNC Symbol;Acc:HGNC:10349]	HGLibA_41969;HGLibA_41970
C7orf66	C7orf66	3	-2.8789	-0.79929	0.03554	0.85525	0.42412	0.95153	-	HGLibA_06578;HGLibA_06579;HGLibA_06577
ENSG00000138621	PPCDC	2	-2.9949	-2.6132	0.03228	0.84371	0.0089698	0.48577	phosphopantothenoylcysteine decarboxylase [Source:HGNC Symbol;Acc:HGNC:28107]	HGLibA_37857;HGLibA_37859
ENSG00000127515	OR7A10	3	-2.5453	-2.6586	0.044816	0.87787	0.0078459	0.44998	olfactory receptor family 7 subfamily A member 10 [Source:HGNC Symbol;Acc:HGNC:8356]	HGLibA_34458;HGLibA_34457;HGLibA_34456
ENSG00000085231	AK6	2	-3.0819	-3.619	0.030306	0.84371	0.00029574	0.044727	adenylate kinase 6 [Source:HGNC Symbol;Acc:HGNC:49151]	HGLibA_01390;HGLibA_01391
ENSG00000180376	CCDC66	2	-4.1758	-4.733	0.0050969	0.79902	2.21E-06	0.0010951	coiled-coil domain containing 66 [Source:HGNC Symbol;Acc:HGNC:27709]	HGLibA_07835;HGLibA_07834
ENSG00000164061	BSN	3	-3.6712	-4.9318	0.015337	0.84371	8.15E-07	0.00047957	bassoon presynaptic cytomatrix protein [Source:HGNC Symbol;Acc:HGNC:1117]	HGLibA_04869;HGLibA_04870;HGLibA_04868
ENSG00000100162	CENPM	2	-3.2294	-3.6957	0.02677	0.84371	0.00021928	0.035906	centromere protein M [Source:HGNC Symbol;Acc:HGNC:18352]	HGLibA_09070;HGLibA_09072
ENSG00000168389	MFSD2A	2	-2.6922	-2.0103	0.040454	0.87097	0.044395	0.95153	major facilitator superfamily domain containing 2A [Source:HGNC Symbol;Acc:HGNC:25897]	HGLibA_29094;HGLibA_29093
ENSG00000184436	THAP7	2	-2.8231	-1.0113	0.036918	0.85956	0.31187	0.95153	THAP domain containing 7 [Source:HGNC Symbol;Acc:HGNC:23190]	HGLibA_49310;HGLibA_49309
ENSG00000197054	ZNF763	3	-3.6479	-4.5733	0.01552	0.84371	4.80E-06	0.0017423	zinc finger protein 763 [Source:HGNC Symbol;Acc:HGNC:27614]	HGLibA_56687;HGLibA_56685;HGLibA_56686
ENSG00000173486	FKBP2	2	-2.8641	-0.8994	0.036	0.8559	0.36844	0.95153	FK506 binding protein 2 [Source:HGNC Symbol;Acc:HGNC:3718]	HGLibA_17571;HGLibA_17570
ENSG00000140025	EFCAB11	3	-3.1272	-3.8752	0.029296	0.84371	0.00010655	0.020906	EF-hand calcium binding domain 11 [Source:HGNC Symbol;Acc:HGNC:20357]	HGLibA_14523;HGLibA_14524;HGLibA_14522
ENSG00000232196	MTRNR2L4	2	-4.2156	-4.9677	0.0045	0.784	6.78E-07	0.00042157	MT-RNR2 like 4 [Source:HGNC Symbol;Acc:HGNC:37161]	HGLibA_30480;HGLibA_30481
ENSG00000111432	FZD10	3	-4.2194	-6.7142	0.0045	0.784	1.89E-11	3.76E-07	frizzled class receptor 10 [Source:HGNC Symbol;Acc:HGNC:4039]	HGLibA_18262;HGLibA_18263;HGLibA_18264
ENSG00000160994	CCDC105	1	-3.0274	-2.2802	0.031362	0.84371	0.022593	0.87251	coiled-coil domain containing 105 [Source:HGNC Symbol;Acc:HGNC:26866]	HGLibA_07519
ENSG00000100271	TTLL1	3	-4.0699	-5.951	0.0069336	0.79902	2.66E-09	5.55E-06	tubulin tyrosine ligase like 1 [Source:HGNC Symbol;Acc:HGNC:1312]	HGLibA_52187;HGLibA_52185;HGLibA_52186
ENSG00000117036	ETV3	2	-3.3767	-1.1965	0.023189	0.84371	0.2315	0.95153	ETS variant 3 [Source:HGNC Symbol;Acc:HGNC:3492]	HGLibA_15654;HGLibA_15653
VPRBP	VPRBP	1	-3.8675	-2.8312	0.010883	0.84371	0.0046378	0.32114	-	HGLibA_53718
ENSG00000092051	JPH4	1	-4.1571	-3.0268	0.0052806	0.79902	0.0024717	0.22244	junctophilin 4 [Source:HGNC Symbol;Acc:HGNC:20156]	HGLibA_23989
ENSG00000221858	OR2A12	3	-2.5579	-1.3996	0.044632	0.87787	0.16163	0.95153	olfactory receptor family 2 subfamily A member 12 [Source:HGNC Symbol;Acc:HGNC:15082]	HGLibA_33723;HGLibA_33722;HGLibA_33721
ENSG00000035115	SH3YL1	3	-2.7018	-0.93371	0.040362	0.87097	0.35049	0.95153	SH3 and SYLF domain containing 1 [Source:HGNC Symbol;Acc:HGNC:29546]	HGLibA_43984;HGLibA_43985;HGLibA_43986
ENSG00000180251	SLC9A4	2	-2.9662	-0.84435	0.032969	0.84371	0.39847	0.95153	solute carrier family 9 member A4 [Source:HGNC Symbol;Acc:HGNC:11077]	HGLibA_45414;HGLibA_45415
ENSG00000110422	HIPK3	3	-3.7164	-5.6594	0.014235	0.84371	1.52E-08	2.20E-05	homeodomain interacting protein kinase 3 [Source:HGNC Symbol;Acc:HGNC:4915]	HGLibA_21346;HGLibA_21344;HGLibA_21345
ENSG00000084623	EIF3I	1	-3.1068	-2.331	0.029663	0.84371	0.019753	0.80279	eukaryotic translation initiation factor 3 subunit I [Source:HGNC Symbol;Acc:HGNC:3272]	HGLibA_14789
C22orf28	C22orf28	1	-4.1438	-3.0177	0.0054642	0.79902	0.0025467	0.22637	-	HGLibA_06064
ENSG00000198900	TOP1	3	-3.1539	-1.3573	0.028791	0.84371	0.17468	0.95153	DNA topoisomerase I [Source:HGNC Symbol;Acc:HGNC:11986]	HGLibA_50987;HGLibA_50988;HGLibA_50989
ENSG00000083937	CHMP2B	2	-3.5061	-1.4114	0.019837	0.84371	0.15814	0.95153	charged multivesicular body protein 2B [Source:HGNC Symbol;Acc:HGNC:24537]	HGLibA_09481;HGLibA_09482
ENSG00000006116	CACNG3	2	-2.9624	-0.32089	0.032969	0.84371	0.74829	0.95525	calcium voltage-gated channel auxiliary subunit gamma 3 [Source:HGNC Symbol;Acc:HGNC:1407]	HGLibA_06935;HGLibA_06936
ENSG00000025796	SEC63	2	-3.7954	-2.5859	0.012627	0.84371	0.0097121	0.51338	protein translocation regulator [Source:HGNC Symbol;Acc:HGNC:21082]"	HGLibA_43262;HGLibA_43261
ENSG00000107014	RLN2	3	-2.8195	-0.88523	0.036918	0.85956	0.37603	0.95153	relaxin 2 [Source:HGNC Symbol;Acc:HGNC:10027]	HGLibA_41354;HGLibA_41356;HGLibA_41355
ENSG00000172086	KRCC1	2	-2.6377	-0.83229	0.042199	0.87558	0.40524	0.95153	lysine rich coiled-coil 1 [Source:HGNC Symbol;Acc:HGNC:28039]	HGLibA_25399;HGLibA_25398
hsa-mir-5007	hsa-mir-5007	3	-2.4472	-3.114	0.047938	0.87787	0.0018455	0.18352	-	HGLibA_61433;HGLibA_61430;HGLibA_61432
ENSG00000136802	LRRC8A	1	-3.4016	-2.5221	0.022546	0.84371	0.011667	0.57992	leucine rich repeat containing 8 VRAC subunit A [Source:HGNC Symbol;Acc:HGNC:19027]	HGLibA_27428
ENSG00000075089	ACTR6	3	-2.9069	-1.5619	0.034806	0.85011	0.1183	0.95153	ARP6 actin related protein 6 homolog [Source:HGNC Symbol;Acc:HGNC:24025]	HGLibA_00672;HGLibA_00670;HGLibA_00671
ENSG00000120800	UTP20	2	-3.6354	-4.6165	0.01575	0.84371	3.90E-06	0.0014909	small subunit processome component [Source:HGNC Symbol;Acc:HGNC:17897]"	HGLibA_53446;HGLibA_53445
SHFM1	SHFM1	1	-3.8315	-2.807	0.011709	0.84371	0.0050007	0.33406	-	HGLibA_44028
ENSG00000162923	WDR26	3	-2.8299	-3.8227	0.03678	0.85944	0.000132	0.023563	WD repeat domain 26 [Source:HGNC Symbol;Acc:HGNC:21208]	HGLibA_54055;HGLibA_54056;HGLibA_54054
ENSG00000167680	SEMA6B	2	-3.9106	-3.0369	0.010332	0.84371	0.0023903	0.21737	semaphorin 6B [Source:HGNC Symbol;Acc:HGNC:10739]	HGLibA_43367;HGLibA_43366
ENSG00000148690	FRA10AC1	2	-3.5519	-4.5193	0.018367	0.84371	6.20E-06	0.0021445	FRA10A associated CGG repeat 1 [Source:HGNC Symbol;Acc:HGNC:1162]	HGLibA_17972;HGLibA_17973
ENSG00000137575	SDCBP	2	-3.4417	-1.1741	0.021627	0.84371	0.24035	0.95153	syndecan binding protein [Source:HGNC Symbol;Acc:HGNC:10662]	HGLibA_43108;HGLibA_43109
ENSG00000168172	HOOK3	3	-2.6248	-3.2971	0.042933	0.87558	0.00097685	0.11438	hook microtubule tethering protein 3 [Source:HGNC Symbol;Acc:HGNC:23576]	HGLibA_21881;HGLibA_21882;HGLibA_21883
ENSG00000155858	LSM11	2	-3.1833	-1.4443	0.027964	0.84371	0.14866	0.95153	U7 small nuclear RNA associated [Source:HGNC Symbol;Acc:HGNC:30860]"	HGLibA_27524;HGLibA_27523
ENSG00000071539	TRIP13	2	-2.3909	-2.881	0.049729	0.87787	0.0039646	0.29274	thyroid hormone receptor interactor 13 [Source:HGNC Symbol;Acc:HGNC:12307]	HGLibA_51642;HGLibA_51643
ENSG00000119421	NDUFA8	1	-3.0274	-2.2802	0.031362	0.84371	0.022593	0.87251	NADH:ubiquinone oxidoreductase subunit A8 [Source:HGNC Symbol;Acc:HGNC:7692]	HGLibA_31473
ENSG00000117407	ARTN	2	-3.8654	-3.9522	0.011112	0.84371	7.74E-05	0.016217	artemin [Source:HGNC Symbol;Acc:HGNC:727]	HGLibA_03169;HGLibA_03171
ENSG00000171094	ALK	2	-3.9092	-3.8703	0.010332	0.84371	0.0001087	0.021137	ALK receptor tyrosine kinase [Source:HGNC Symbol;Acc:HGNC:427]	HGLibA_01644;HGLibA_01643
ENSG00000160049	DFFA	3	-4.639	-6.4419	0.0011479	0.67568	1.18E-10	4.31E-07	DNA fragmentation factor subunit alpha [Source:HGNC Symbol;Acc:HGNC:2772]	HGLibA_13020;HGLibA_13018;HGLibA_13019
ENSG00000092010	PSME1	2	-3.1933	-3.382	0.02778	0.84371	0.00071954	0.090579	proteasome activator subunit 1 [Source:HGNC Symbol;Acc:HGNC:9568]	HGLibA_39191;HGLibA_39189
ENSG00000205683	DPF3	3	-2.7608	-1.414	0.038571	0.86735	0.15735	0.95153	double PHD fingers 3 [Source:HGNC Symbol;Acc:HGNC:17427]	HGLibA_13832;HGLibA_13833;HGLibA_13834
ENSG00000143882	ATP6V1C2	1	-3.0679	-2.3061	0.030489	0.84371	0.021105	0.83392	ATPase H+ transporting V1 subunit C2 [Source:HGNC Symbol;Acc:HGNC:18264]	HGLibA_03743
ENSG00000122435	TRMT13	2	-3.7714	-4.1048	0.013224	0.84371	4.05E-05	0.010131	tRNA methyltransferase 13 homolog [Source:HGNC Symbol;Acc:HGNC:25502]	HGLibA_51678;HGLibA_51677
ENSG00000055130	CUL1	2	-2.6627	-2.0518	0.041693	0.87558	0.040184	0.95153	cullin 1 [Source:HGNC Symbol;Acc:HGNC:2551]	HGLibA_11773;HGLibA_11772
ENSG00000100410	PHF5A	2	-3.4703	-3.4936	0.02103	0.84371	0.00047651	0.06568	PHD finger protein 5A [Source:HGNC Symbol;Acc:HGNC:18000]	HGLibA_36380;HGLibA_36381
ENSG00000151503	NCAPD3	1	-2.6853	-2.0654	0.040913	0.87097	0.038888	0.95153	non-SMC condensin II complex subunit D3 [Source:HGNC Symbol;Acc:HGNC:28952]	HGLibA_31266
MEF2BNB	MEF2BNB	3	-3.5444	-3.0887	0.018643	0.84371	0.0020102	0.19457	-	HGLibA_28831;HGLibA_28833;HGLibA_28832
ENSG00000143622	RIT1	3	-2.8047	-2.058	0.037193	0.85956	0.039594	0.95153	Ras like without CAAX 1 [Source:HGNC Symbol;Acc:HGNC:10023]	HGLibA_41338;HGLibA_41337;HGLibA_41336
ENSG00000139746	RBM26	2	-4.37	-5.3876	0.002801	0.784	7.14E-08	7.77E-05	RNA binding motif protein 26 [Source:HGNC Symbol;Acc:HGNC:20327]	HGLibA_40521;HGLibA_40520
ENSG00000243130	PSG11	3	-2.6096	-3.6486	0.043117	0.87558	0.00026365	0.041608	pregnancy specific beta-1-glycoprotein 11 [Source:HGNC Symbol;Acc:HGNC:9516]	HGLibA_39036;HGLibA_39037;HGLibA_39038
ENSG00000137807	KIF23	1	-3.2487	-2.4225	0.026127	0.84371	0.015414	0.6925	kinesin family member 23 [Source:HGNC Symbol;Acc:HGNC:6392]	HGLibA_24908
ENSG00000093009	CDC45	1	-2.9852	-2.2534	0.032464	0.84371	0.024235	0.91789	cell division cycle 45 [Source:HGNC Symbol;Acc:HGNC:1739]	HGLibA_08587
ENSG00000118513	MYB	1	-3.2487	-2.4225	0.026127	0.84371	0.015414	0.6925	transcription factor [Source:HGNC Symbol;Acc:HGNC:7545]"	HGLibA_30636
ENSG00000143742	SRP9	3	-3.5556	-4.8176	0.018275	0.84371	1.45E-06	0.000805	signal recognition particle 9 [Source:HGNC Symbol;Acc:HGNC:11304]	HGLibA_47015;HGLibA_47014;HGLibA_47016
ENSG00000147655	RSPO2	2	-2.6525	-2.7023	0.041877	0.87558	0.0068856	0.41539	R-spondin 2 [Source:HGNC Symbol;Acc:HGNC:28583]	HGLibA_42347;HGLibA_42346;HGLibA_42348
ENSG00000132792	CTNNBL1	2	-3.4809	-4.2707	0.020663	0.84371	1.95E-05	0.0056845	catenin beta like 1 [Source:HGNC Symbol;Acc:HGNC:15879]	HGLibA_11656;HGLibA_11655
CCRN4L	CCRN4L	1	-3.6057	-2.6565	0.016393	0.84371	0.0078957	0.44998	-	HGLibA_08179
ENSG00000176490	DIRAS1	2	-3.8233	-2.9545	0.011939	0.84371	0.0031317	0.25528	DIRAS family GTPase 1 [Source:HGNC Symbol;Acc:HGNC:19127]	HGLibA_13257;HGLibA_13256
ENSG00000164109	MAD2L1	2	-3.0085	-1.3491	0.032005	0.84371	0.1773	0.95153	mitotic arrest deficient 2 like 1 [Source:HGNC Symbol;Acc:HGNC:6763]	HGLibA_27846;HGLibA_27844
ENSG00000096063	SRPK1	2	-2.4709	-0.36133	0.047479	0.87787	0.71785	0.95266	SRSF protein kinase 1 [Source:HGNC Symbol;Acc:HGNC:11305]	HGLibA_47019;HGLibA_47017
ENSG00000124813	RUNX2	2	-3.1235	-1.4812	0.029296	0.84371	0.13856	0.95153	runt related transcription factor 2 [Source:HGNC Symbol;Acc:HGNC:10472]	HGLibA_42460;HGLibA_42461
ENSG00000130706	ADRM1	3	-2.942	-3.4042	0.033658	0.84936	0.0006637	0.084526	adhesion regulating molecule 1 [Source:HGNC Symbol;Acc:HGNC:15759]	HGLibA_01069;HGLibA_01070;HGLibA_01071
ENSG00000147432	CHRNB3	3	-3.0241	-1.9013	0.031775	0.84371	0.057267	0.95153	cholinergic receptor nicotinic beta 3 subunit [Source:HGNC Symbol;Acc:HGNC:1963]	HGLibA_09594;HGLibA_09596;HGLibA_09595
ENSG00000135314	KHDC1	3	-3.1741	-1.2903	0.028423	0.84371	0.19696	0.95153	KH domain containing 1 [Source:HGNC Symbol;Acc:HGNC:21366]	HGLibA_24556;HGLibA_24558;HGLibA_24557
ENSG00000115289	PCGF1	3	-3.9401	-2.9881	0.0094132	0.84371	0.0028072	0.24086	polycomb group ring finger 1 [Source:HGNC Symbol;Acc:HGNC:17615]	HGLibA_35593;HGLibA_35592;HGLibA_35591
ENSG00000129559	NEDD8	1	-4.6145	-3.34	0.0013316	0.725	0.00083766	0.10023	developmentally down-regulated 8 [Source:HGNC Symbol;Acc:HGNC:7732]"	HGLibA_31609
ENSG00000112081	SRSF3	2	-3.1765	-3.6427	0.028331	0.84371	0.00026974	0.042262	serine and arginine rich splicing factor 3 [Source:HGNC Symbol;Acc:HGNC:10785]	HGLibA_47076;HGLibA_47074
ENSG00000160209	PDXK	3	-3.3183	-2.7123	0.024612	0.84371	0.0066824	0.40651	pyridoxal kinase [Source:HGNC Symbol;Acc:HGNC:8819]	HGLibA_35962;HGLibA_35961;HGLibA_35960
ENSG00000100298	APOBEC3H	3	-3.2233	-1.3364	0.026908	0.84371	0.18142	0.95153	apolipoprotein B mRNA editing enzyme catalytic subunit 3H [Source:HGNC Symbol;Acc:HGNC:24100]	HGLibA_02494;HGLibA_02493;HGLibA_02495
ENSG00000164815	ORC5	1	-3.4831	-2.5756	0.020296	0.84371	0.010007	0.52324	origin recognition complex subunit 5 [Source:HGNC Symbol;Acc:HGNC:8491]	HGLibA_34612
ENSG00000102030	NAA10	2	-2.7491	-0.90282	0.038847	0.86735	0.36662	0.95153	NatA catalytic subunit [Source:HGNC Symbol;Acc:HGNC:18704]"	HGLibA_30970;HGLibA_30971
ENSG00000109846	CRYAB	1	-4.1166	-2.9993	0.0061071	0.79902	0.0027059	0.23478	crystallin alpha B [Source:HGNC Symbol;Acc:HGNC:2389]	HGLibA_11248
ENSG00000118579	MED28	2	-3.8633	-3.3433	0.011112	0.84371	0.00082792	0.10017	mediator complex subunit 28 [Source:HGNC Symbol;Acc:HGNC:24628]	HGLibA_28795;HGLibA_28796
ENSG00000162775	RBM15	1	-3.8675	-2.8312	0.010883	0.84371	0.0046378	0.32114	RNA binding motif protein 15 [Source:HGNC Symbol;Acc:HGNC:14959]	HGLibA_40492
ENSG00000099194	SCD	1	-3.2151	-2.4007	0.027046	0.84371	0.016362	0.71696	stearoyl-CoA desaturase [Source:HGNC Symbol;Acc:HGNC:10571]	HGLibA_42883
ENSG00000163795	ZNF513	3	-2.528	-0.65114	0.045367	0.87787	0.51496	0.95153	zinc finger protein 513 [Source:HGNC Symbol;Acc:HGNC:26498]	HGLibA_56137;HGLibA_56136;HGLibA_56138
ENSG00000163798	SLC4A1AP	3	-2.4026	-2.7287	0.049454	0.87787	0.0063593	0.39205	solute carrier family 4 member 1 adaptor protein [Source:HGNC Symbol;Acc:HGNC:13813]	HGLibA_45210;HGLibA_45212;HGLibA_45211
ENSG00000196497	IPO4	2	-3.2163	-1.7959	0.027046	0.84371	0.072518	0.95153	importin 4 [Source:HGNC Symbol;Acc:HGNC:19426]	HGLibA_23467;HGLibA_23466
ENSG00000216588	IGSF23	3	-3.6395	-2.983	0.015658	0.84371	0.002854	0.24184	immunoglobulin superfamily member 23 [Source:HGNC Symbol;Acc:HGNC:40040]	HGLibA_22889;HGLibA_22891;HGLibA_22890
hsa-mir-7641-1	hsa-mir-7641-1	1	-2.4192	-1.903	0.048627	0.87787	0.057035	0.95153	-	HGLibA_63914
ENSG00000101246	ARFRP1	1	-3.9523	-2.8882	0.0090917	0.84371	0.0038747	0.28799	ADP ribosylation factor related protein 1 [Source:HGNC Symbol;Acc:HGNC:662]	HGLibA_02681
ENSG00000047662	FAM184B	2	-2.432	-0.59709	0.04826	0.87787	0.55045	0.95153	family with sequence similarity 184 member B [Source:HGNC Symbol;Acc:HGNC:29235]	HGLibA_16330;HGLibA_16329
ENSG00000215018	COL28A1	3	-3.1078	-3.4811	0.029663	0.84371	0.00049941	0.067553	collagen type XXVIII alpha 1 chain [Source:HGNC Symbol;Acc:HGNC:22442]	HGLibA_10557;HGLibA_10556;HGLibA_10558
ENSG00000120868	APAF1	2	-3.455	-0.94271	0.02149	0.84371	0.34583	0.95153	apoptotic peptidase activating factor 1 [Source:HGNC Symbol;Acc:HGNC:576]	HGLibA_02365;HGLibA_02366
ENSG00000179271	GADD45GIP1	1	-3.7352	-2.7426	0.013729	0.84371	0.0060957	0.38086	GADD45G interacting protein 1 [Source:HGNC Symbol;Acc:HGNC:29996]	HGLibA_18426
ENSG00000136319	TTC5	2	-2.7277	-3.4856	0.039719	0.87097	0.00049102	0.067254	tetratricopeptide repeat domain 5 [Source:HGNC Symbol;Acc:HGNC:19274]	HGLibA_52146;HGLibA_52148
ENSG00000188800	TMCO2	1	-3.2812	-2.4436	0.025484	0.84371	0.014542	0.66812	transmembrane and coiled-coil domains 2 [Source:HGNC Symbol;Acc:HGNC:23312]	HGLibA_49784
hsa-mir-3646	hsa-mir-3646	4	-2.7082	-1.5875	0.04027	0.87097	0.11239	0.95153	-	HGLibA_59133;HGLibA_59135;HGLibA_59134;HGLibA_59132
ENSG00000188846	RPL14	2	-3.0006	-3.8986	0.032188	0.84371	9.67E-05	0.019341	ribosomal protein L14 [Source:HGNC Symbol;Acc:HGNC:10305]	HGLibA_41874;HGLibA_41873
C17orf89	C17orf89	2	-3.5074	-2.8429	0.019791	0.84371	0.0044711	0.31821	-	HGLibA_05592;HGLibA_05591
ENSG00000135744	AGT	2	-3.3285	-4.028	0.024474	0.84371	5.63E-05	0.013033	angiotensinogen [Source:HGNC Symbol;Acc:HGNC:333]	HGLibA_01255;HGLibA_01257
ENSG00000198918	RPL39	3	-3.2108	-3.9873	0.027505	0.84371	6.68E-05	0.014851	ribosomal protein L39 [Source:HGNC Symbol;Acc:HGNC:10350]	HGLibA_41973;HGLibA_41972;HGLibA_41974
ENSG00000169189	NSMCE1	1	-3.2812	-2.4436	0.025484	0.84371	0.014542	0.66812	SMC5-SMC6 complex component [Source:HGNC Symbol;Acc:HGNC:29897]"	HGLibA_32767
ENSG00000132423	COQ3	3	-3.849	-5.1466	0.011571	0.84371	2.65E-07	0.00019857	methyltransferase [Source:HGNC Symbol;Acc:HGNC:18175]"	HGLibA_10751;HGLibA_10752;HGLibA_10753
ENSG00000161547	SRSF2	3	-2.6927	-0.66307	0.040454	0.87097	0.50729	0.95153	serine and arginine rich splicing factor 2 [Source:HGNC Symbol;Acc:HGNC:10783]	HGLibA_47071;HGLibA_47073;HGLibA_47072
ENSG00000066044	ELAVL1	2	-3.4081	-1.1858	0.022408	0.84371	0.2357	0.95153	ELAV like RNA binding protein 1 [Source:HGNC Symbol;Acc:HGNC:3312]	HGLibA_14878;HGLibA_14880
C3orf30	C3orf30	2	-3.9953	-4.7845	0.0081734	0.83871	1.71E-06	0.00088754	-	HGLibA_06228;HGLibA_06226
ENSG00000141627	DYM	3	-2.4379	-2.8925	0.047984	0.87787	0.0038213	0.28785	dymeclin [Source:HGNC Symbol;Acc:HGNC:21317]	HGLibA_14225;HGLibA_14226;HGLibA_14227
ENSG00000100029	PES1	2	-3.4951	-3.0273	0.02002	0.84371	0.0024674	0.22244	pescadillo ribosomal biogenesis factor 1 [Source:HGNC Symbol;Acc:HGNC:8848]	HGLibA_36067;HGLibA_36065
ENSG00000164144	ARFIP1	3	-2.6945	-0.64471	0.040408	0.87097	0.51911	0.95153	ADP ribosylation factor interacting protein 1 [Source:HGNC Symbol;Acc:HGNC:21496]	HGLibA_02676;HGLibA_02675;HGLibA_02677
RNF219	RNF219	3	-3.3595	-2.668	0.023694	0.84371	0.007631	0.43965	-	HGLibA_41637;HGLibA_41636;HGLibA_41638
ENSG00000166396	SERPINB7	2	-3.7517	-4.2049	0.0135	0.84371	2.61E-05	0.0071999	serpin family B member 7 [Source:HGNC Symbol;Acc:HGNC:13902]	HGLibA_43562;HGLibA_43561
ENSG00000181273	OR5AK2	2	-3.023	-0.84691	0.031775	0.84371	0.39705	0.95153	olfactory receptor family 5 subfamily AK member 2 [Source:HGNC Symbol;Acc:HGNC:15251]	HGLibA_34238;HGLibA_34239
ENSG00000091651	ORC6	1	-2.4927	-1.9474	0.046469	0.87787	0.051484	0.95153	origin recognition complex subunit 6 [Source:HGNC Symbol;Acc:HGNC:17151]	HGLibA_34616
ENSG00000101460	MAP1LC3A	3	-4.2276	-6.626	0.004454	0.784	3.45E-11	3.76E-07	microtubule associated protein 1 light chain 3 alpha [Source:HGNC Symbol;Acc:HGNC:6838]	HGLibA_28121;HGLibA_28122;HGLibA_28120
C19orf40	C19orf40	3	-4.2601	-6.4556	0.0037653	0.784	1.08E-10	4.31E-07	-	HGLibA_05657;HGLibA_05658;HGLibA_05656
ENSG00000133398	MED10	3	-3.5565	-3.0016	0.018275	0.84371	0.002686	0.23478	mediator complex subunit 10 [Source:HGNC Symbol;Acc:HGNC:28760]	HGLibA_28736;HGLibA_28735;HGLibA_28737
ENSG00000185306	C12orf56	3	-3.5053	-5.2118	0.019837	0.84371	1.87E-07	0.00015579	chromosome 12 open reading frame 56 [Source:HGNC Symbol;Acc:HGNC:26967]	HGLibA_05267;HGLibA_05268;HGLibA_05266
ENSG00000134748	PRPF38A	2	-2.9085	-0.99928	0.034668	0.85011	0.31766	0.95153	pre-mRNA processing factor 38A [Source:HGNC Symbol;Acc:HGNC:25930]	HGLibA_38723;HGLibA_38722
FAM194A	FAM194A	2	-3.39	-4.335	0.022959	0.84371	1.46E-05	0.0045347	-	HGLibA_16369;HGLibA_16368
ENSG00000163161	ERCC3	1	-2.7421	-2.1005	0.03903	0.86735	0.035682	0.95153	TFIIH core complex helicase subunit [Source:HGNC Symbol;Acc:HGNC:3435]"	HGLibA_15437
ENSG00000163472	TMEM79	2	-2.8996	-1.4287	0.034898	0.85011	0.1531	0.95153	transmembrane protein 79 [Source:HGNC Symbol;Acc:HGNC:28196]	HGLibA_50515;HGLibA_50516
ENSG00000183098	GPC6	3	-2.5344	-1.1535	0.045183	0.87787	0.24871	0.95153	glypican 6 [Source:HGNC Symbol;Acc:HGNC:4454]	HGLibA_19778;HGLibA_19776;HGLibA_19777
ENSG00000205339	IPO7	3	-3.7353	-4.1993	0.013729	0.84371	2.68E-05	0.0072892	importin 7 [Source:HGNC Symbol;Acc:HGNC:9852]	HGLibA_23473;HGLibA_23472;HGLibA_23471
hsa-mir-5691	hsa-mir-5691	1	-2.5612	-1.9891	0.044081	0.87787	0.046694	0.95153	-	HGLibA_62283
ENSG00000070731	ST6GALNAC2	3	-4.3123	-6.4409	0.0033979	0.784	1.19E-10	4.31E-07	6-sialyltransferase 2 [Source:HGNC Symbol;Acc:HGNC:10867]"	HGLibA_47268;HGLibA_47266;HGLibA_47267
ENSG00000198026	ZNF335	1	-3.4016	-2.5221	0.022546	0.84371	0.011667	0.57992	zinc finger protein 335 [Source:HGNC Symbol;Acc:HGNC:15807]	HGLibA_55824
ENSG00000164611	PTTG1	1	-3.534	-2.6091	0.018872	0.84371	0.0090784	0.48577	pituitary tumor-transforming 1 [Source:HGNC Symbol;Acc:HGNC:9690]	HGLibA_39563
ENSG00000197442	MAP3K5	3	-3.3863	-1.3533	0.022959	0.84371	0.17597	0.95153	mitogen-activated protein kinase kinase kinase 5 [Source:HGNC Symbol;Acc:HGNC:6857]	HGLibA_28189;HGLibA_28191;HGLibA_28190

ENSG00000147082	CCNB3	2	-2.766	-0.83517	0.038525	0.86735	0.40362	0.95153	cyclin B3 [Source:HGNC Symbol;Acc:HGNC:18709]	HGLibA_08067;HGLibA_08066
ENSG00000095485	CWF19L1	2	-3.7959	-2.6275	0.012627	0.84371	0.0086006	0.47023	CWF19 like cell cycle control factor 1 [Source:HGNC Symbol;Acc:HGNC:25613]	HGLibA_11824;HGLibA_11823
ENSG00000137207	YIPF3	3	-3.1223	-5.0501	0.029387	0.84371	4.42E-07	0.00029142	Yip1 domain family member 3 [Source:HGNC Symbol;Acc:HGNC:21023]	HGLibA_54676;HGLibA_54677;HGLibA_54675
ENSG00000112282	MED23	1	-2.895	-2.1963	0.034898	0.85011	0.028072	0.95153	mediator complex subunit 23 [Source:HGNC Symbol;Acc:HGNC:2372]	HGLibA_28782
ENSG00000196805	SPRR2B	3	-2.942	-1.906	0.033658	0.84936	0.056649	0.95153	small proline rich protein 2B [Source:HGNC Symbol;Acc:HGNC:11262]	HGLibA_46810;HGLibA_46812;HGLibA_46811
ENSG00000151876	FBXO4	2	-3.6989	-3.6381	0.014831	0.84371	0.00027467	0.042424	F-box protein 4 [Source:HGNC Symbol;Acc:HGNC:13583]	HGLibA_17089;HGLibA_17091
ENSG00000122406	RPL5	2	-3.3752	-4.3142	0.023234	0.84371	1.60E-05	0.0049126	ribosomal protein L5 [Source:HGNC Symbol;Acc:HGNC:10360]	HGLibA_41988;HGLibA_41986
ENSG00000036549	ZZZ3	2	-2.398	-1.0418	0.049591	0.87787	0.29752	0.95153	zinc finger ZZ-type containing 3 [Source:NCBI gene;Acc:26009]	HGLibA_57094;HGLibA_57095
ENSG00000185267	CDNF	2	-3.1109	-0.97821	0.029617	0.84371	0.32797	0.95153	cerebral dopamine neurotrophic factor [Source:HGNC Symbol;Acc:HGNC:24913]	HGLibA_08852;HGLibA_08851
ENSG00000241794	SPRR2A	3	-3.027	-2.1537	0.031729	0.84371	0.031262	0.95153	small proline rich protein 2A [Source:HGNC Symbol;Acc:HGNC:11261]	HGLibA_46807;HGLibA_46809;HGLibA_46808
ENSG00000196981	WDR5B	2	-3.5383	-4.2697	0.018735	0.84371	1.96E-05	0.0056845	WD repeat domain 5B [Source:HGNC Symbol;Acc:HGNC:17826]	HGLibA_54134;HGLibA_54132
ENSG00000103415	HMOX2	2	-3.407	-1.9661	0.022408	0.84371	0.049292	0.95153	heme oxygenase 2 [Source:HGNC Symbol;Acc:HGNC:5014]	HGLibA_21750;HGLibA_21751
ENSG00000139697	SBNO1	2	-3.3743	-4.1264	0.023234	0.84371	3.68E-05	0.0093305	strawberry notch homolog 1 [Source:HGNC Symbol;Acc:HGNC:22973]	HGLibA_42807;HGLibA_42806
ENSG00000083067	TRPM3	2	-3.9626	-4.6919	0.0089081	0.84371	2.71E-06	0.0011335	transient receptor potential cation channel subfamily M member 3 [Source:HGNC Symbol;Acc:HGNC:17992]	HGLibA_51759;HGLibA_51757
ENSG00000204435	CSNK2B	3	-2.4702	-0.69703	0.047479	0.87787	0.48579	0.95153	casein kinase 2 beta [Source:HGNC Symbol;Acc:HGNC:2460]	HGLibA_11411;HGLibA_11410;HGLibA_11412
ENSG00000110713	NUP98	3	-2.6639	-1.8936	0.041693	0.87558	0.058273	0.95153	nucleoporin 98 [Source:HGNC Symbol;Acc:HGNC:8068]	HGLibA_33077;HGLibA_33079;HGLibA_33078
ENSG00000058262	SEC61A1	2	-3.4303	-3.7756	0.021857	0.84371	0.00015964	0.026539	Sec61 translocon alpha 1 subunit [Source:HGNC Symbol;Acc:HGNC:18276]	HGLibA_43248;HGLibA_43247
ENSG00000167986	DDB1	2	-2.7836	-3.6491	0.038112	0.86735	0.00026319	0.041608	damage specific DNA binding protein 1 [Source:HGNC Symbol;Acc:HGNC:2717]	HGLibA_12593;HGLibA_12594
hsa-mir-3145	hsa-mir-3145	2	-2.4576	-0.86847	0.047709	0.87787	0.38514	0.95153	-	HGLibA_58638;HGLibA_58639
ENSG00000175073	VCPIP1	3	-2.7357	-3.8542	0.039673	0.87097	0.00011611	0.021989	valosin containing protein interacting protein 1 [Source:HGNC Symbol;Acc:HGNC:30897]	HGLibA_53578;HGLibA_53579;HGLibA_53577
ENSG00000205639	MFSD2B	2	-4.5339	-4.6506	0.001653	0.76812	3.31E-06	0.0013166	major facilitator superfamily domain containing 2B [Source:HGNC Symbol;Acc:HGNC:37207]	HGLibA_29095;HGLibA_29097
ENSG00000189171	S100A13	2	-4.4102	-4.6949	0.0025255	0.77465	2.67E-06	0.0011335	S100 calcium binding protein A13 [Source:HGNC Symbol;Acc:HGNC:10490]	HGLibA_42542;HGLibA_42541
ENSG00000170348	TMED10	2	-3.6738	-2.9675	0.015199	0.84371	0.0030021	0.24954	transmembrane p24 trafficking protein 10 [Source:HGNC Symbol;Acc:HGNC:16998]	HGLibA_49802;HGLibA_49801
ENSG00000167371	PRRT2	3	-2.993	-1.2929	0.032326	0.84371	0.19604	0.95153	proline rich transmembrane protein 2 [Source:HGNC Symbol;Acc:HGNC:30500]	HGLibA_38885;HGLibA_38884;HGLibA_38883
ENSG00000100216	TOMM22	2	-3.2213	-1.9911	0.026954	0.84371	0.046466	0.95153	translocase of outer mitochondrial membrane 22 [Source:HGNC Symbol;Acc:HGNC:18002]	HGLibA_50961;HGLibA_50960
ENSG00000156709	AIFM1	1	-3.1803	-2.3783	0.02801	0.84371	0.017394	0.74422	apoptosis inducing factor mitochondria associated 1 [Source:HGNC Symbol;Acc:HGNC:8768]	HGLibA_01337
ENSG00000115204	MPV17	2	-4.5127	-5.6381	0.0018826	0.76812	1.72E-08	2.20E-05	mitochondrial inner membrane protein MPV17 [Source:HGNC Symbol;Acc:HGNC:7224]	HGLibA_29784;HGLibA_29782
ENSG00000143107	FNDC7	2	-2.6074	-3.4779	0.043117	0.87558	0.00050538	0.067939	fibronectin type III domain containing 7 [Source:HGNC Symbol;Acc:HGNC:26668]	HGLibA_17755;HGLibA_17756
