# Supplementary material for: A genome-wide CRISPR knockout screen identified host genes essential for Brucella invasion and intracellular survival
Source: Emerg Microbes Infect. 2026 Aug 19;15(1):2713322. doi: 10.1080/22221751.2026.2713322 (PMC13491901; doi:10.1080/22221751.2026.2713322)
Supplement: Supplementary file 3 [file TEMI_A_2713322_SM6416.pdf]

Table S3. Biological Process (BP) terms enriched by the 35 candidate genes.

ID	Description	GeneRatio	BgRatio	pvalue	p.adjust	qvalue	geneID	Count
GO:0016226	iron-sulfur cluster assembly	2/28	26/18800	0.000679995	0.151638831	0.127409544	LYRM4/FXN	2
GO:0031163	metallo-sulfur cluster assembly	2/28	26/18800	0.000679995	0.151638831	0.127409544	LYRM4/FXN	2
GO:0006790	sulfur compound metabolic process	4/28	339/18800	0.001510515	0.224563276	0.188681912	GGT6/LYRM4/MTHFD1/FXN	4
GO:0042398	cellular modified amino acid biosynthetic process	2/28	46/18800	0.002126034	0.237052784	0.199175811	GGT6/MTHFD1	2
GO:0090307	mitotic spindle assembly	2/28	69/18800	0.004718261	0.237140808	0.19924977	STAG2/MYBL2	2
GO:0000045	autophagosome assembly	2/28	107/18800	0.011015134	0.237140808	0.19924977	ATG16L1/ATP2A2	2
GO:0015980	energy derivation by oxidation of organic compounds	3/28	321/18800	0.011779872	0.237140808	0.19924977	COX5A/UGP2/FXN	3
GO:1905037	autophagosome organization	2/28	111/18800	0.011814803	0.237140808	0.19924977	ATG16L1/ATP2A2	2
GO:0051225	spindle assembly	2/28	121/18800	0.013922153	0.237140808	0.19924977	STAG2/MYBL2	2
GO:0007052	mitotic spindle organization	2/28	124/18800	0.014583974	0.237140808	0.19924977	STAG2/MYBL2	2
GO:0009396	folic acid-containing compound biosynthetic process	1/28	10/18800	0.014797712	0.237140808	0.19924977	MTHFD1	1
GO:0035999	tetrahydrofolate interconversion	1/28	10/18800	0.014797712	0.237140808	0.19924977	MTHFD1	1
GO:0048703	embryonic viscerocranium morphogenesis	1/28	10/18800	0.014797712	0.237140808	0.19924977	MTHFD1	1
GO:1901748	leukotriene D4 metabolic process	1/28	10/18800	0.014797712	0.237140808	0.19924977	GGT6	1
GO:1901750	leukotriene D4 biosynthetic process	1/28	10/18800	0.014797712	0.237140808	0.19924977	GGT6	1
GO:0006547	histidine metabolic process	1/28	11/18800	0.016265816	0.237140808	0.19924977	MTHFD1	1
GO:0035404	histone-serine phosphorylation	1/28	11/18800	0.016265816	0.237140808	0.19924977	VRK1	1
GO:0040015	negative regulation of multicellular organism growth	1/28	11/18800	0.016265816	0.237140808	0.19924977	FXN	1
GO:0051081	nuclear membrane disassembly	1/28	11/18800	0.016265816	0.237140808	0.19924977	VRK1	1
GO:0006661	phosphatidylinositol biosynthetic process	2/28	134/18800	0.016886462	0.237140808	0.19924977	IPMK/CDIPT	2
GO:0006617	SRP-dependent cotranslational protein targeting to membrane, signal sequence recognition	1/28	12/18800	0.017731809	0.237140808	0.19924977	SRP54	1
GO:0009086	methionine biosynthetic process	1/28	12/18800	0.017731809	0.237140808	0.19924977	MTHFD1	1
GO:0030397	membrane disassembly	1/28	12/18800	0.017731809	0.237140808	0.19924977	VRK1	1
GO:0032957	inositol trisphosphate metabolic process	1/28	12/18800	0.017731809	0.237140808	0.19924977	IPMK	1
GO:0006119	oxidative phosphorylation	2/28	139/18800	0.018092305	0.237140808	0.19924977	COX5A/FXN	2
GO:0098792	xenophagy	1/28	13/18800	0.019195696	0.237140808	0.19924977	ATG16L1	1
GO:1900121	negative regulation of receptor binding	1/28	13/18800	0.019195696	0.237140808	0.19924977	ATP2A2	1
GO:0044272	sulfur compound biosynthetic process	2/28	149/18800	0.02061038	0.237140808	0.19924977	GGT6/MTHFD1	2
GO:0014733	regulation of skeletal muscle adaptation	1/28	14/18800	0.02065748	0.237140808	0.19924977	ATP2A2	1
GO:0043650	dicarboxylic acid biosynthetic process	1/28	14/18800	0.02065748	0.237140808	0.19924977	MTHFD1	1
GO:0046341	CDP-diacylglycerol metabolic process	1/28	14/18800	0.02065748	0.237140808	0.19924977	CDIPT	1
GO:1902850	microtubule cytoskeleton organization involved in mitosis	2/28	151/18800	0.021130716	0.237140808	0.19924977	STAG2/MYBL2	2
GO:0042559	pteridine-containing compound biosynthetic process	1/28	15/18800	0.022117162	0.237140808	0.19924977	MTHFD1	1
GO:0048308	organelle inheritance	1/28	15/18800	0.022117162	0.237140808	0.19924977	VRK1	1
GO:0048313	Golgi inheritance	1/28	15/18800	0.022117162	0.237140808	0.19924977	VRK1	1
GO:0006555	methionine metabolic process	1/28	16/18800	0.023574746	0.237140808	0.19924977	MTHFD1	1
GO:0006750	glutathione biosynthetic process	1/28	16/18800	0.023574746	0.237140808	0.19924977	GGT6	1
GO:0018410	C-terminal protein amino acid modification	1/28	16/18800	0.023574746	0.237140808	0.19924977	ATG16L1	1
GO:0046488	phosphatidylinositol metabolic process	2/28	162/18800	0.0240898	0.237140808	0.19924977	IPMK/CDIPT	2
GO:0000097	sulfur amino acid biosynthetic process	1/28	17/18800	0.025030235	0.237140808	0.19924977	MTHFD1	1
GO:0034497	protein localization to phagophore assembly site	1/28	17/18800	0.025030235	0.237140808	0.19924977	ATG16L1	1
GO:0048791	calcium ion-regulated exocytosis of neurotransmitter	1/28	17/18800	0.025030235	0.237140808	0.19924977	ATP2A2	1
GO:0050884	neuromuscular process controlling posture	1/28	17/18800	0.025030235	0.237140808	0.19924977	FXN	1
GO:0060382	regulation of DNA strand elongation	1/28	17/18800	0.025030235	0.237140808	0.19924977	INO80E	1
GO:0006123	mitochondrial electron transport, cytochrome c to oxygen	1/28	18/18800	0.026483632	0.237140808	0.19924977	COX5A	1
GO:0019184	nonribosomal peptide biosynthetic process	1/28	18/18800	0.026483632	0.237140808	0.19924977	GGT6	1
GO:0006379	mRNA cleavage	1/28	20/18800	0.029384161	0.237140808	0.19924977	CPSF3	1
GO:0008334	histone mRNA metabolic process	1/28	20/18800	0.029384161	0.237140808	0.19924977	CPSF3	1
GO:0046653	tetrahydrofolate metabolic process	1/28	20/18800	0.029384161	0.237140808	0.19924977	MTHFD1	1
GO:0090201	negative regulation of release of cytochrome c from mitochondria	1/28	20/18800	0.029384161	0.237140808	0.19924977	FXN	1
GO:0009067	aspartate family amino acid biosynthetic process	1/28	21/18800	0.030831298	0.237140808	0.19924977	MTHFD1	1
GO:0009070	serine family amino acid biosynthetic process	1/28	21/18800	0.030831298	0.237140808	0.19924977	MTHFD1	1
GO:0019370	leukotriene biosynthetic process	1/28	21/18800	0.030831298	0.237140808	0.19924977	GGT6	1
GO:0009060	aerobic respiration	2/28	187/18800	0.03140269	0.237140808	0.19924977	COX5A/FXN	2
GO:0006575	cellular modified amino acid metabolic process	2/28	188/18800	0.031711515	0.237140808	0.19924977	GGT6/MTHFD1	2
GO:0007033	vacuole organization	2/28	188/18800	0.031711515	0.237140808	0.19924977	ATG16L1/ATP2A2	2
GO:0007051	spindle organization	2/28	188/18800	0.031711515	0.237140808	0.19924977	STAG2/MYBL2	2
GO:0080182	histone H3-K4 trimethylation	1/28	23/18800	0.033719334	0.237140808	0.19924977	NFYA	1
GO:1900120	regulation of receptor binding	1/28	23/18800	0.033719334	0.237140808	0.19924977	ATP2A2	1
GO:0043501	skeletal muscle adaptation	1/28	24/18800	0.035160238	0.237140808	0.19924977	ATP2A2	1
GO:0006091	generation of precursor metabolites and energy	3/28	494/18800	0.036319674	0.237140808	0.19924977	COX5A/UGP2/FXN	3
GO:0006614	SRP-dependent cotranslational protein targeting to membrane	1/28	25/18800	0.036599071	0.237140808	0.19924977	SRP54	1
GO:0032469	endoplasmic reticulum calcium ion homeostasis	1/28	25/18800	0.036599071	0.237140808	0.19924977	ATP2A2	1
GO:0006836	neurotransmitter transport	2/28	207/18800	0.037806006	0.237140808	0.19924977	SYNGR3/ATP2A2	2
GO:0002026	regulation of the force of heart contraction	1/28	26/18800	0.038035834	0.237140808	0.19924977	ATP2A2	1
GO:0010882	regulation of cardiac muscle contraction by calcium ion signaling	1/28	26/18800	0.038035834	0.237140808	0.19924977	ATP2A2	1
GO:0045822	negative regulation of heart contraction	1/28	26/18800	0.038035834	0.237140808	0.19924977	ATP2A2	1
GO:0010460	positive regulation of heart rate	1/28	27/18800	0.03947053	0.237140808	0.19924977	ATP2A2	1
GO:0016540	protein autoprocessing	1/28	27/18800	0.03947053	0.237140808	0.19924977	FXN	1
GO:1903523	negative regulation of blood circulation	1/28	27/18800	0.03947053	0.237140808	0.19924977	ATP2A2	1
GO:0001505	regulation of neurotransmitter levels	2/28	213/18800	0.039817554	0.237140808	0.19924977	SYNGR3/ATP2A2	2
GO:0046474	glycerophospholipid biosynthetic process	2/28	214/18800	0.040156753	0.237140808	0.19924977	IPMK/CDIPT	2
GO:0006760	folic acid-containing compound metabolic process	1/28	28/18800	0.040903164	0.237140808	0.19924977	MTHFD1	1
GO:0032958	inositol phosphate biosynthetic process	1/28	28/18800	0.040903164	0.237140808	0.19924977	IPMK	1
GO:0098901	regulation of cardiac muscle cell action potential	1/28	28/18800	0.040903164	0.237140808	0.19924977	ATP2A2	1
GO:1903779	regulation of cardiac conduction	1/28	28/18800	0.040903164	0.237140808	0.19924977	ATP2A2	1
GO:0007628	adult walking behavior	1/28	30/18800	0.043762252	0.237140808	0.19924977	FXN	1
GO:0003299	muscle hypertrophy in response to stress	1/28	31/18800	0.045188712	0.237140808	0.19924977	ATP2A2	1
GO:0006613	cotranslational protein targeting to membrane	1/28	31/18800	0.045188712	0.237140808	0.19924977	SRP54	1
GO:0006783	heme biosynthetic process	1/28	31/18800	0.045188712	0.237140808	0.19924977	FXN	1
GO:0010039	response to iron ion	1/28	31/18800	0.045188712	0.237140808	0.19924977	FXN	1
GO:0014887	cardiac muscle adaptation	1/28	31/18800	0.045188712	0.237140808	0.19924977	ATP2A2	1
GO:0014898	cardiac muscle hypertrophy in response to stress	1/28	31/18800	0.045188712	0.237140808	0.19924977	ATP2A2	1
GO:0090659	walking behavior	1/28	31/18800	0.045188712	0.237140808	0.19924977	FXN	1
GO:0045333	cellular respiration	2/28	231/18800	0.046090761	0.237140808	0.19924977	COX5A/FXN	2
GO:0045055	regulated exocytosis	2/28	234/18800	0.04717002	0.237140808	0.19924977	SYNGR3/ATP2A2	2
GO:0000096	sulfur amino acid metabolic process	1/28	33/18800	0.048035479	0.237140808	0.19924977	MTHFD1	1
GO:0009225	nucleotide-sugar metabolic process	1/28	33/18800	0.048035479	0.237140808	0.19924977	UGP2	1
GO:0006691	leukotriene metabolic process	1/28	34/18800	0.049455791	0.237140808	0.19924977	GGT6	1
GO:0086004	regulation of cardiac muscle cell contraction	1/28	34/18800	0.049455791	0.237140808	0.19924977	ATP2A2	1
GO:0006779	porphyrin-containing compound biosynthetic process	1/28	35/18800	0.05087406	0.237140808	0.19924977	FXN	1
GO:0033014	tetrapyrrole biosynthetic process	1/28	35/18800	0.05087406	0.237140808	0.19924977	FXN	1
GO:0042558	pteridine-containing compound metabolic process	1/28	35/18800	0.05087406	0.237140808	0.19924977	MTHFD1	1
GO:0030851	granulocyte differentiation	1/28	36/18800	0.052290289	0.237140808	0.19924977	SRP54	1
GO:0039694	viral RNA genome replication	1/28	36/18800	0.052290289	0.237140808	0.19924977	ATG16L1	1
GO:0022616	DNA strand elongation	1/28	37/18800	0.053704479	0.237140808	0.19924977	INO80E	1
GO:0046621	negative regulation of organ growth	1/28	37/18800	0.053704479	0.237140808	0.19924977	FXN	1
GO:0045017	glycerolipid biosynthetic process	2/28	253/18800	0.05421888	0.237140808	0.19924977	IPMK/CDIPT	2
GO:0008654	phospholipid biosynthetic process	2/28	258/18800	0.056133218	0.237140808	0.19924977	IPMK/CDIPT	2
GO:0043687	post-translational protein modification	1/28	39/18800	0.056526758	0.237140808	0.19924977	ATG16L1	1
GO:0045823	positive regulation of heart contraction	1/28	39/18800	0.056526758	0.237140808	0.19924977	ATP2A2	1
GO:0006730	one-carbon metabolic process	1/28	40/18800	0.057934852	0.237140808	0.19924977	MTHFD1	1
GO:0009069	serine family amino acid metabolic process	1/28	40/18800	0.057934852	0.237140808	0.19924977	MTHFD1	1
GO:0017158	regulation of calcium ion-dependent exocytosis	1/28	40/18800	0.057934852	0.237140808	0.19924977	ATP2A2	1
GO:1903115	regulation of actin filament-based movement	1/28	40/18800	0.057934852	0.237140808	0.19924977	ATP2A2	1
GO:1903524	positive regulation of blood circulation	1/28	40/18800	0.057934852	0.237140808	0.19924977	ATP2A2	1
GO:0030488	tRNA methylation	1/28	41/18800	0.05934092	0.237140808	0.19924977	WDR4	1

GO:0070266	necroptotic process	1/28	42/18800	0.060744963	0.237140808	0.19924977	IPMK	1
GO:0070296	sarcoplasmic reticulum calcium ion transport	1/28	42/18800	0.060744963	0.237140808	0.19924977	ATP2A2	1
GO:0071763	nuclear membrane organization	1/28	42/18800	0.060744963	0.237140808	0.19924977	VRK1	1
GO:0090503	RNA phosphodiester bond hydrolysis, exonucleolytic	1/28	42/18800	0.060744963	0.237140808	0.19924977	CPSF3	1
GO:0046034	ATP metabolic process	2/28	273/18800	0.062017851	0.237140808	0.19924977	COX5A/FXN	2
GO:0001504	neurotransmitter uptake	1/28	43/18800	0.062146986	0.237140808	0.19924977	SYNGR3	1
GO:0021762	substantia nigra development	1/28	44/18800	0.06354699	0.237140808	0.19924977	SYNGR3	1
GO:0006378	mRNA polyadenylation	1/28	45/18800	0.06494498	0.237140808	0.19924977	CPSF3	1
GO:0006984	ER-nucleus signaling pathway	1/28	45/18800	0.06494498	0.237140808	0.19924977	ATP2A2	1
GO:0016572	histone phosphorylation	1/28	45/18800	0.06494498	0.237140808	0.19924977	VRK1	1
GO:0045047	protein targeting to ER	1/28	45/18800	0.06494498	0.237140808	0.19924977	SRP54	1
GO:0005978	glycogen biosynthetic process	1/28	46/18800	0.066340956	0.237140808	0.19924977	UGP2	1
GO:0009250	glucan biosynthetic process	1/28	46/18800	0.066340956	0.237140808	0.19924977	UGP2	1
GO:0042168	heme metabolic process	1/28	46/18800	0.066340956	0.237140808	0.19924977	FXN	1
GO:0043631	RNA polyadenylation	1/28	46/18800	0.066340956	0.237140808	0.19924977	CPSF3	1
GO:0048701	embryonic cranial skeleton morphogenesis	1/28	46/18800	0.066340956	0.237140808	0.19924977	MTHFD1	1
GO:0090199	regulation of release of cytochrome c from mitochondria	1/28	46/18800	0.066340956	0.237140808	0.19924977	FXN	1
GO:0034599	cellular response to oxidative stress	2/28	284/18800	0.066463231	0.237140808	0.19924977	FXN/ATP2A2	2
GO:0035272	exocrine system development	1/28	48/18800	0.069126883	0.24091285	0.202419104	SRP54	1
GO:0140014	mitotic nuclear division	2/28	293/18800	0.070178672	0.24091285	0.202419104	STAG2/MYBL2	2
GO:0009066	aspartate family amino acid metabolic process	1/28	49/18800	0.070516838	0.24091285	0.202419104	MTHFD1	1
GO:0072599	establishment of protein localization to endoplasmic reticulum	1/28	49/18800	0.070516838	0.24091285	0.202419104	SRP54	1
GO:0097300	programmed necrotic cell death	1/28	49/18800	0.070516838	0.24091285	0.202419104	IPMK	1
GO:1901570	fatty acid derivative biosynthetic process	1/28	50/18800	0.071904793	0.24091285	0.202419104	GGT6	1
GO:0010823	negative regulation of mitochondrion organization	1/28	51/18800	0.073290748	0.24091285	0.202419104	FXN	1
GO:0051349	positive regulation of lyase activity	1/28	51/18800	0.073290748	0.24091285	0.202419104	FXN	1
GO:0043525	positive regulation of neuron apoptotic process	1/28	52/18800	0.074674708	0.24091285	0.202419104	MYBL2	1
GO:0065002	intracellular protein transmembrane transport	1/28	52/18800	0.074674708	0.24091285	0.202419104	SRP54	1
GO:0016236	macroautophagy	2/28	306/18800	0.075664751	0.24091285	0.202419104	ATG16L1/ATP2A2	2
GO:0006778	porphyrin-containing compound metabolic process	1/28	53/18800	0.076056674	0.24091285	0.202419104	FXN	1
GO:1900087	positive regulation of G1/S transition of mitotic cell cycle	1/28	53/18800	0.076056674	0.24091285	0.202419104	CPSF3	1
GO:0006650	glycerophospholipid metabolic process	2/28	309/18800	0.076950161	0.24091285	0.202419104	IPMK/CDIPT	2
GO:0014888	striated muscle adaptation	1/28	54/18800	0.07743665	0.24091285	0.202419104	ATP2A2	1
GO:0043647	inositol phosphate metabolic process	1/28	55/18800	0.078814639	0.24091285	0.202419104	IPMK	1
GO:0097352	autophagosome maturation	1/28	55/18800	0.078814639	0.24091285	0.202419104	ATP2A2	1
GO:0046394	carboxylic acid biosynthetic process	2/28	316/18800	0.079976892	0.24091285	0.202419104	GGT6/MTHFD1	2
GO:0001836	release of cytochrome c from mitochondria	1/28	56/18800	0.080190643	0.24091285	0.202419104	FXN	1
GO:0046148	pigment biosynthetic process	1/28	56/18800	0.080190643	0.24091285	0.202419104	FXN	1
GO:2000300	regulation of synaptic vesicle exocytosis	1/28	56/18800	0.080190643	0.24091285	0.202419104	ATP2A2	1
GO:0016053	organic acid biosynthetic process	2/28	318/18800	0.080848619	0.24091285	0.202419104	GGT6/MTHFD1	2
GO:0006998	nuclear envelope organization	1/28	57/18800	0.081564664	0.24091285	0.202419104	VRK1	1
GO:0018023	peptidyl-lysine trimethylation	1/28	57/18800	0.081564664	0.24091285	0.202419104	NFYA	1
GO:0046456	icosanoid biosynthetic process	1/28	57/18800	0.081564664	0.24091285	0.202419104	GGT6	1
GO:0098900	regulation of action potential	1/28	57/18800	0.081564664	0.24091285	0.202419104	ATP2A2	1
GO:0035065	regulation of histone acetylation	1/28	59/18800	0.084306772	0.245756997	0.20648924	NFYA	1
GO:0051353	positive regulation of oxidoreductase activity	1/28	59/18800	0.084306772	0.245756997	0.20648924	FXN	1
GO:0043414	macromolecule methylation	2/28	331/18800	0.086587911	0.24912848	0.209322018	WDR4/NFYA	2
GO:0062197	cellular response to chemical stress	2/28	332/18800	0.08703453	0.24912848	0.209322018	FXN/ATP2A2	2
GO:0010506	regulation of autophagy	2/28	336/18800	0.08882816	0.24912848	0.209322018	ATG16L1/MAGEA6	2
GO:0007062	sister chromatid cohesion	1/28	63/18800	0.089767324	0.24912848	0.209322018	STAG2	1
GO:0031124	mRNA 3'-end processing	1/28	63/18800	0.089767324	0.24912848	0.209322018	CPSF3	1
GO:0046173	polyol biosynthetic process	1/28	63/18800	0.089767324	0.24912848	0.209322018	IPMK	1
GO:0071806	protein transmembrane transport	1/28	63/18800	0.089767324	0.24912848	0.209322018	SRP54	1
GO:0040014	regulation of multicellular organism growth	1/28	64/18800	0.091127548	0.24912848	0.209322018	FXN	1
GO:0048857	neural nucleus development	1/28	64/18800	0.091127548	0.24912848	0.209322018	SYNGR3	1
GO:0006749	glutathione metabolic process	1/28	65/18800	0.092485812	0.24912848	0.209322018	GGT6	1
GO:0017156	calcium-ion regulated exocytosis	1/28	65/18800	0.092485812	0.24912848	0.209322018	ATP2A2	1
GO:0006879	cellular iron ion homeostasis	1/28	66/18800	0.093842118	0.24912848	0.209322018	FXN	1
GO:0033013	tetrapyrrole metabolic process	1/28	66/18800	0.093842118	0.24912848	0.209322018	FXN	1
GO:0070265	necrotic cell death	1/28	66/18800	0.093842118	0.24912848	0.209322018	IPMK	1
GO:1902808	positive regulation of cell cycle G1/S phase transition	1/28	66/18800	0.093842118	0.24912848	0.209322018	CPSF3	1
GO:0033692	cellular polysaccharide biosynthetic process	1/28	67/18800	0.09519647	0.251228553	0.211086535	UGP2	1
GO:0046854	phosphatidylinositol phosphate biosynthetic process	1/28	68/18800	0.096548869	0.251817518	0.211581394	IPMK	1
GO:2000756	regulation of peptidyl-lysine acetylation	1/28	68/18800	0.096548869	0.251817518	0.211581394	NFYA	1
GO:0006887	exocytosis	2/28	357/18800	0.098425993	0.254025204	0.213436329	SYNGR3/ATP2A2	2
GO:1901607	alpha-amino acid biosynthetic process	1/28	70/18800	0.099247823	0.254025204	0.213436329	MTHFD1	1
GO:1904888	cranial skeletal system development	1/28	70/18800	0.099247823	0.254025204	0.213436329	MTHFD1	1
GO:0051568	histone H3-K4 methylation	1/28	71/18800	0.100594382	0.254025204	0.213436329	NFYA	1
GO:0005977	glycogen metabolic process	1/28	73/18800	0.10328168	0.254025204	0.213436329	UGP2	1
GO:0000271	polysaccharide biosynthetic process	1/28	74/18800	0.104622424	0.254025204	0.213436329	UGP2	1
GO:0006073	cellular glucan metabolic process	1/28	74/18800	0.104622424	0.254025204	0.213436329	UGP2	1
GO:0044042	glucan metabolic process	1/28	74/18800	0.104622424	0.254025204	0.213436329	UGP2	1
GO:1901568	fatty acid derivative metabolic process	1/28	74/18800	0.104622424	0.254025204	0.213436329	GGT6	1
GO:0086003	cardiac muscle cell contraction	1/28	75/18800	0.105961235	0.254025204	0.213436329	ATP2A2	1
GO:0140056	organelle localization by membrane tethering	1/28	75/18800	0.105961235	0.254025204	0.213436329	ATP2A2	1
GO:0008344	adult locomotory behavior	1/28	76/18800	0.107298116	0.254025204	0.213436329	FXN	1
GO:0008652	cellular amino acid biosynthetic process	1/28	76/18800	0.107298116	0.254025204	0.213436329	MTHFD1	1
GO:0061912	selective autophagy	1/28	76/18800	0.107298116	0.254025204	0.213436329	ATG16L1	1
GO:0032259	methylation	2/28	376/18800	0.107356397	0.254025204	0.213436329	WDR4/NFYA	2
GO:0051339	regulation of lyase activity	1/28	77/18800	0.108633068	0.254025204	0.213436329	FXN	1
GO:0055117	regulation of cardiac muscle contraction	1/28	77/18800	0.108633068	0.254025204	0.213436329	ATP2A2	1
GO:0070972	protein localization to endoplasmic reticulum	1/28	77/18800	0.108633068	0.254025204	0.213436329	SRP54	1
GO:0086001	cardiac muscle cell action potential	1/28	77/18800	0.108633068	0.254025204	0.213436329	ATP2A2	1
GO:0140694	non-membrane-bounded organelle assembly	2/28	379/18800	0.108786578	0.254025204	0.213436329	STAG2/MYBL2	2
GO:0042440	pigment metabolic process	1/28	78/18800	0.109966096	0.255442077	0.21462681	FXN	1
GO:0034637	cellular carbohydrate biosynthetic process	1/28	79/18800	0.111297201	0.257194568	0.216099283	UGP2	1
GO:0006644	phospholipid metabolic process	2/28	388/18800	0.11310855	0.25942756	0.217975482	IPMK/CDIPT	2
GO:0031016	pancreas development	1/28	81/18800	0.113953654	0.25942756	0.217975482	SRP54	1
GO:1901983	regulation of protein acetylation	1/28	82/18800	0.115279008	0.25942756	0.217975482	NFYA	1
GO:0001510	RNA methylation	1/28	83/18800	0.11660245	0.25942756	0.217975482	WDR4	1
GO:0090502	RNA phosphodiester bond hydrolysis, endonucleolytic	1/28	83/18800	0.11660245	0.25942756	0.217975482	CPSF3	1
GO:0046486	glycerolipid metabolic process	2/28	396/18800	0.116988693	0.25942756	0.217975482	IPMK/CDIPT	2
GO:0019646	aerobic electron transport chain	1/28	84/18800	0.117923983	0.25942756	0.217975482	COX5A	1
GO:0022406	membrane docking	1/28	84/18800	0.117923983	0.25942756	0.217975482	ATP2A2	1
GO:0061053	somite development	1/28	84/18800	0.117923983	0.25942756	0.217975482	MTHFD1	1
GO:0008543	fibroblast growth factor receptor signaling pathway	1/28	85/18800	0.11924361	0.25942756	0.217975482	KLB	1
GO:0010507	negative regulation of autophagy	1/28	85/18800	0.11924361	0.25942756	0.217975482	MAGEA6	1
GO:0055072	iron ion homeostasis	1/28	85/18800	0.11924361	0.25942756	0.217975482	FXN	1
GO:0006112	energy reserve metabolic process	1/28	86/18800	0.120561332	0.259760165	0.218254942	UGP2	1
GO:0046928	regulation of neurotransmitter secretion	1/28	86/18800	0.120561332	0.259760165	0.218254942	ATP2A2	1
GO:1901216	positive regulation of neuron death	1/28	87/18800	0.121877154	0.261332743	0.219576249	MYBL2	1
GO:0006400	tRNA modification	1/28	88/18800	0.123191077	0.262886221	0.220881507	WDR4	1
GO:0001843	neural tube closure	1/28	90/18800	0.125813237	0.264682566	0.222390827	MTHFD1	1
GO:0030901	midbrain development	1/28	90/18800	0.125813237	0.264682566	0.222390827	SYNGR3	1
GO:0070301	cellular response to hydrogen peroxide	1/28	90/18800	0.125813237	0.264682566	0.222390827	FXN	1
GO:0060606	tube closure	1/28	91/18800	0.127121481	0.264766207	0.222461104	MTHFD1	1
GO:0070509	calcium ion import	1/28	91/18800	0.127121481	0.264766207	0.222461104	ATP2A2	1
GO:0042773	ATP synthesis coupled electron transport	1/28	92/18800	0.128427836	0.264766207	0.222461104	COX5A	1
GO:0042775	mitochondrial ATP synthesis coupled electron transport	1/28	92/18800	0.128427836	0.264766207	0.222461104	COX5A	1

GO:0048704	embryonic skeletal system morphogenesis	1/28	94/18800	0.131034893	0.264766207	0.222461104	MTHFD1	1
GO:0043502	regulation of muscle adaptation	1/28	95/18800	0.132335599	0.264766207	0.222461104	ATP2A2	1
GO:0043648	dicarboxylic acid metabolic process	1/28	95/18800	0.132335599	0.264766207	0.222461104	MTHFD1	1
GO:0003300	cardiac muscle hypertrophy	1/28	96/18800	0.133634429	0.264766207	0.222461104	ATP2A2	1
GO:0006497	protein lipidation	1/28	96/18800	0.133634429	0.264766207	0.222461104	ATG16L1	1
GO:0006942	regulation of striated muscle contraction	1/28	96/18800	0.133634429	0.264766207	0.222461104	ATP2A2	1
GO:0014020	primary neural tube formation	1/28	96/18800	0.133634429	0.264766207	0.222461104	MTHFD1	1
GO:0032091	negative regulation of protein binding	1/28	96/18800	0.133634429	0.264766207	0.222461104	ATP2A2	1
GO:0072594	establishment of protein localization to organelle	2/28	431/18800	0.134356125	0.264766207	0.222461104	IPO8/SRP54	2
GO:0044264	cellular polysaccharide metabolic process	1/28	97/18800	0.134931383	0.264766207	0.222461104	UGP2	1
GO:0006979	response to oxidative stress	2/28	433/18800	0.135366452	0.264766207	0.222461104	FXN/ATP2A2	2
GO:0061337	cardiac conduction	1/28	98/18800	0.136226465	0.264766207	0.222461104	ATP2A2	1
GO:0014897	striated muscle hypertrophy	1/28	99/18800	0.137519678	0.264766207	0.222461104	ATP2A2	1
GO:0051588	regulation of neurotransmitter transport	1/28	99/18800	0.137519678	0.264766207	0.222461104	ATP2A2	1
GO:1990830	cellular response to leukemia inhibitory factor	1/28	99/18800	0.137519678	0.264766207	0.222461104	MYBL2	1
GO:1901992	positive regulation of mitotic cell cycle phase transition	1/28	100/18800	0.138811023	0.264766207	0.222461104	CPSF3	1
GO:1990823	response to leukemia inhibitory factor	1/28	100/18800	0.138811023	0.264766207	0.222461104	MYBL2	1
GO:0014896	muscle hypertrophy	1/28	101/18800	0.140100504	0.264766207	0.222461104	ATP2A2	1
GO:0042158	lipoprotein biosynthetic process	1/28	101/18800	0.140100504	0.264766207	0.222461104	ATG16L1	1
GO:0046620	regulation of organ growth	1/28	101/18800	0.140100504	0.264766207	0.222461104	FXN	1
GO:0000280	nuclear division	2/28	446/18800	0.141976899	0.267180156	0.224489345	STAG2/MYBL2	2
GO:0070252	actin-mediated cell contraction	1/28	103/18800	0.142673882	0.267363662	0.22464353	ATP2A2	1
GO:0001841	neural tube formation	1/28	104/18800	0.143957785	0.268640888	0.225716677	MTHFD1	1
GO:0002027	regulation of heart rate	1/28	105/18800	0.145239834	0.269904025	0.226777986	ATP2A2	1
GO:0030593	neutrophil chemotaxis	1/28	106/18800	0.146520031	0.270032784	0.226886172	SRP54	1
GO:0051341	regulation of oxidoreductase activity	1/28	106/18800	0.146520031	0.270032784	0.226886172	FXN	1
GO:0008637	apoptotic mitochondrial changes	1/28	107/18800	0.147798379	0.270156054	0.226989745	FXN	1
GO:0016079	synaptic vesicle exocytosis	1/28	107/18800	0.147798379	0.270156054	0.226989745	ATP2A2	1
GO:0005976	polysaccharide metabolic process	1/28	108/18800	0.149074881	0.270456651	0.227242313	UGP2	1
GO:0022411	cellular component disassembly	2/28	460/18800	0.149175642	0.270456651	0.227242313	VRK1/ATP2A2	2
GO:0048640	negative regulation of developmental growth	1/28	111/18800	0.152893333	0.276074601	0.23196261	FXN	1
GO:0022904	respiratory electron transport chain	1/28	113/18800	0.155429782	0.278400333	0.23391673	COX5A	1
GO:0044344	cellular response to fibroblast growth factor stimulus	1/28	113/18800	0.155429782	0.278400333	0.23391673	KLB	1
GO:0018107	peptidyl-threonine phosphorylation	1/28	115/18800	0.157958907	0.280675987	0.235828774	VRK1	1
GO:0046916	cellular transition metal ion homeostasis	1/28	115/18800	0.157958907	0.280675987	0.235828774	FXN	1
GO:0019751	polyol metabolic process	1/28	116/18800	0.15922073	0.281788996	0.236763943	IPMK	1
GO:0031123	RNA 3'-end processing	1/28	117/18800	0.160480729	0.281788996	0.236763943	CPSF3	1
GO:0032411	positive regulation of transporter activity	1/28	117/18800	0.160480729	0.281788996	0.236763943	SYNGR3	1
GO:0043500	muscle adaptation	1/28	118/18800	0.161738907	0.282884519	0.23768442	ATP2A2	1
GO:0071774	response to fibroblast growth factor	1/28	119/18800	0.162995266	0.283968316	0.238595045	KLB	1
GO:0006690	icosanoid metabolic process	1/28	121/18800	0.165502541	0.286101291	0.240387207	GGT6	1
GO:1903008	organelle disassembly	1/28	121/18800	0.165502541	0.286101291	0.240387207	VRK1	1
GO:0048285	organelle fission	2/28	493/18800	0.166437371	0.286411431	0.240647792	STAG2/MYBL2	2
GO:0016570	histone modification	2/28	494/18800	0.166966305	0.286411431	0.240647792	VRK1/NFYA	2
GO:1901989	positive regulation of cell cycle phase transition	1/28	123/18800	0.168002572	0.287016902	0.241156519	CPSF3	1
GO:0001838	embryonic epithelial tube formation	1/28	124/18800	0.169249877	0.287016902	0.241156519	MTHFD1	1
GO:0018210	peptidyl-threonine modification	1/28	124/18800	0.169249877	0.287016902	0.241156519	VRK1	1
GO:0008033	tRNA processing	1/28	125/18800	0.17049538	0.287787435	0.241803934	WDR4	1
GO:0034968	histone lysine methylation	1/28	127/18800	0.172980985	0.287787435	0.241803934	NFYA	1
GO:0048706	embryonic skeletal system development	1/28	127/18800	0.172980985	0.287787435	0.241803934	MTHFD1	1
GO:0042542	response to hydrogen peroxide	1/28	128/18800	0.174221093	0.287787435	0.241803934	FXN	1
GO:0045931	positive regulation of mitotic cell cycle	1/28	128/18800	0.174221093	0.287787435	0.241803934	CPSF3	1
GO:0071621	granulocyte chemotaxis	1/28	128/18800	0.174221093	0.287787435	0.241803934	SRP54	1
GO:1990266	neutrophil migration	1/28	128/18800	0.174221093	0.287787435	0.241803934	SRP54	1
GO:0006888	endoplasmic reticulum to Golgi vesicle-mediated transport	1/28	129/18800	0.175459407	0.288763453	0.242624001	TRAPPC2	1
GO:0006275	regulation of DNA replication	1/28	131/18800	0.177930667	0.291753961	0.245136677	INO80E	1
GO:0006612	protein targeting to membrane	1/28	132/18800	0.179163617	0.29256754	0.24582026	SRP54	1
GO:0030048	actin filament-based movement	1/28	133/18800	0.180394783	0.29256754	0.24582026	ATP2A2	1
GO:1902600	proton transmembrane transport	1/28	133/18800	0.180394783	0.29256754	0.24582026	COX5A	1
GO:0019079	viral genome replication	1/28	134/18800	0.181624169	0.293494129	0.246598796	ATG16L1	1
GO:0010508	positive regulation of autophagy	1/28	136/18800	0.184077609	0.294260263	0.247242515	ATG16L1	1
GO:0030534	adult behavior	1/28	136/18800	0.184077609	0.294260263	0.247242515	FXN	1
GO:0072175	epithelial tube formation	1/28	136/18800	0.184077609	0.294260263	0.247242515	MTHFD1	1
GO:0006997	nucleus organization	1/28	138/18800	0.186523955	0.296048697	0.248745188	VRK1	1
GO:0060048	cardiac muscle contraction	1/28	138/18800	0.186523955	0.296048697	0.248745188	ATP2A2	1
GO:0055076	transition metal ion homeostasis	1/28	139/18800	0.187744474	0.29675211	0.249336208	FXN	1
GO:0042157	lipoprotein metabolic process	1/28	140/18800	0.188963227	0.29675211	0.249336208	ATG16L1	1
GO:1903305	regulation of regulated secretory pathway	1/28	140/18800	0.188963227	0.29675211	0.249336208	ATP2A2	1
GO:0055123	digestive system development	1/28	142/18800	0.191395446	0.297244209	0.249749678	SRP54	1
GO:0001508	action potential	1/28	143/18800	0.192608916	0.297244209	0.249749678	ATP2A2	1
GO:0018022	peptidyl-lysine methylation	1/28	143/18800	0.192608916	0.297244209	0.249749678	NFYA	1
GO:0035264	multicellular organism growth	1/28	143/18800	0.192608916	0.297244209	0.249749678	FXN	1
GO:0046165	alcohol biosynthetic process	1/28	143/18800	0.192608916	0.297244209	0.249749678	IPMK	1
GO:0007269	neurotransmitter secretion	1/28	145/18800	0.195030591	0.298912864	0.25115171	ATP2A2	1
GO:0099643	signal release from synapse	1/28	145/18800	0.195030591	0.298912864	0.25115171	ATP2A2	1
GO:0002181	cytoplasmic translation	1/28	146/18800	0.1962388	0.299734605	0.251842151	UBA52	1
GO:0034614	cellular response to reactive oxygen species	1/28	147/18800	0.19744526	0.300548075	0.252525643	FXN	1
GO:0010821	regulation of mitochondrion organization	1/28	148/18800	0.198649974	0.301353362	0.253202259	FXN	1
GO:0016331	morphogenesis of embryonic epithelium	1/28	150/18800	0.201054173	0.303966648	0.255397986	MTHFD1	1
GO:0035148	tube formation	1/28	152/18800	0.203451416	0.304494401	0.255841413	MTHFD1	1
GO:0050905	neuromuscular process	1/28	152/18800	0.203451416	0.304494401	0.255841413	FXN	1
GO:0090501	RNA phosphodiester bond hydrolysis	1/28	152/18800	0.203451416	0.304494401	0.255841413	CPSF3	1
GO:0021915	neural tube development	1/28	154/18800	0.205841722	0.306018027	0.25712159	MTHFD1	1
GO:0097530	granulocyte migration	1/28	154/18800	0.205841722	0.306018027	0.25712159	SRP54	1
GO:0007009	plasma membrane organization	1/28	156/18800	0.208225111	0.308532888	0.259234619	ATP2A2	1
GO:0016571	histone methylation	1/28	158/18800	0.210601601	0.311020908	0.261325096	NFYA	1
GO:0006606	protein import into nucleus	1/28	159/18800	0.211787265	0.311426757	0.261666097	IPO8	1
GO:0007030	Golgi organization	1/28	160/18800	0.212971212	0.311426757	0.261666097	VRK1	1
GO:2000045	regulation of G1/S transition of mitotic cell cycle	1/28	160/18800	0.212971212	0.311426757	0.261666097	CPSF3	1
GO:0030307	positive regulation of cell growth	1/28	163/18800	0.216512773	0.314542987	0.264284407	FXN	1
GO:0051170	import into nucleus	1/28	163/18800	0.216512773	0.314542987	0.264284407	IPO8	1
GO:0035637	multicellular organismal signaling	1/28	164/18800	0.217689875	0.315226248	0.264858495	ATP2A2	1
GO:0051100	negative regulation of binding	1/28	165/18800	0.218865271	0.315902624	0.265426798	ATP2A2	1
GO:0009451	RNA modification	1/28	166/18800	0.220038964	0.316218227	0.265691973	WDR4	1
GO:0016573	histone acetylation	1/28	167/18800	0.221210957	0.316218227	0.265691973	NFYA	1
GO:0022900	electron transport chain	1/28	167/18800	0.221210957	0.316218227	0.265691973	COX5A	1
GO:0030041	actin filament polymerization	1/28	169/18800	0.22354985	0.31854068	0.267643338	MSRB1	1
GO:0006937	regulation of muscle contraction	1/28	170/18800	0.224716754	0.31918367	0.268183589	ATP2A2	1
GO:0018393	internal peptidyl-lysine acetylation	1/28	173/18800	0.228207331	0.323112603	0.271484745	NFYA	1
GO:0006475	internal protein amino acid acetylation	1/28	175/18800	0.230525958	0.324336206	0.272512838	NFYA	1
GO:0035265	organ growth	1/28	175/18800	0.230525958	0.324336206	0.272512838	FXN	1
GO:0031056	regulation of histone modification	1/28	176/18800	0.231682752	0.324938702	0.273019065	NFYA	1
GO:0006941	striated muscle contraction	1/28	178/18800	0.233991309	0.327147724	0.274875123	ATP2A2	1
GO:0055001	muscle cell development	1/28	183/18800	0.239733477	0.333087635	0.279865938	ATP2A2	1
GO:0099504	synaptic vesicle cycle	1/28	183/18800	0.239733477	0.333087635	0.279865938	ATP2A2	1
GO:0018394	peptidyl-lysine acetylation	1/28	184/18800	0.240876919	0.333636975	0.280327504	NFYA	1
GO:0006399	tRNA metabolic process	1/28	185/18800	0.242018703	0.334180624	0.280784287	WDR4	1
GO:1902806	regulation of cell cycle G1/S phase transition	1/28	188/18800	0.245434127	0.337850681	0.283867931	CPSF3	1
GO:0060401	cytosolic calcium ion transport	1/28	190/18800	0.247702827	0.339924495	0.285610385	ATP2A2	1

GO:0006164	purine nucleotide biosynthetic process	1/28	192/18800	0.249964948	0.340930786	0.286455888	MTHFD1	1
GO:0007626	locomotory behavior	1/28	192/18800	0.249964948	0.340930786	0.286455888	FXN	1
GO:0043393	regulation of protein binding	1/28	196/18800	0.25446953	0.346016495	0.290728988	ATP2A2	1
GO:0099003	vesicle-mediated transport in synapse	1/28	197/18800	0.255591592	0.346363109	0.291020219	ATP2A2	1
GO:0006479	protein methylation	1/28	199/18800	0.257830834	0.346363109	0.291020219	NFYA	1
GO:0008154	actin polymerization or depolymerization	1/28	199/18800	0.257830834	0.346363109	0.291020219	MSRB1	1
GO:0008213	protein alkylation	1/28	199/18800	0.257830834	0.346363109	0.291020219	NFYA	1
GO:0019722	calcium-mediated signaling	1/28	202/18800	0.261177523	0.348158549	0.292528778	ATP2A2	1
GO:0072522	purine-containing compound biosynthetic process	1/28	202/18800	0.261177523	0.348158549	0.292528778	MTHFD1	1
GO:0000302	response to reactive oxygen species	1/28	203/18800	0.262289848	0.348158549	0.292528778	FXN	1
GO:1901605	alpha-amino acid metabolic process	1/28	203/18800	0.262289848	0.348158549	0.292528778	MTHFD1	1
GO:0017157	regulation of exocytosis	1/28	204/18800	0.263400559	0.348595398	0.292895827	ATP2A2	1
GO:0000819	sister chromatid segregation	1/28	205/18800	0.264509657	0.349027535	0.293258916	STAG2	1
GO:0043523	regulation of neuron apoptotic process	1/28	207/18800	0.266723024	0.350909937	0.294840542	MYBL2	1
GO:0016051	carbohydrate biosynthetic process	1/28	209/18800	0.268929967	0.352145736	0.295878881	UGP2	1
GO:0002573	myeloid leukocyte differentiation	1/28	210/18800	0.270031035	0.352145736	0.295878881	SRP54	1
GO:0017038	protein import	1/28	210/18800	0.270031035	0.352145736	0.295878881	IPO8	1
GO:0008016	regulation of heart contraction	1/28	211/18800	0.271130504	0.352548702	0.29621746	ATP2A2	1
GO:0033044	regulation of chromosome organization	1/28	212/18800	0.272228376	0.352947255	0.296552332	INO80E	1
GO:0006473	protein acetylation	1/28	216/18800	0.27660394	0.357580746	0.30044547	NFYA	1
GO:0046777	protein autophosphorylation	1/28	224/18800	0.285279091	0.367729695	0.308972791	VRK1	1
GO:0000082	G1/S transition of mitotic cell cycle	1/28	225/18800	0.286356405	0.36805463	0.309245806	CPSF3	1
GO:0048705	skeletal system morphogenesis	1/28	228/18800	0.28957896	0.371127058	0.311827313	MTHFD1	1
GO:0097529	myeloid leukocyte migration	1/28	229/18800	0.290650023	0.371432408	0.312083873	SRP54	1
GO:2001234	negative regulation of apoptotic signaling pathway	1/28	230/18800	0.291719529	0.371734029	0.312337301	FXN	1
GO:0030595	leukocyte chemotaxis	1/28	236/18800	0.298103987	0.378787403	0.318263666	SRP54	1
GO:0098657	import into cell	1/28	238/18800	0.300219781	0.380392109	0.319611968	SYNGR3	1
GO:0032984	protein-containing complex disassembly	1/28	239/18800	0.30127537	0.380648201	0.319827141	ATP2A2	1
GO:0051402	neuron apoptotic process	1/28	241/18800	0.303381944	0.382226969	0.321153649	MYBL2	1
GO:0016485	protein processing	1/28	243/18800	0.305482393	0.382711088	0.321560414	FXN	1
GO:1901617	organic hydroxy compound biosynthetic process	1/28	243/18800	0.305482393	0.382711088	0.321560414	IPMK	1
GO:0090305	nucleic acid phosphodiester bond hydrolysis	1/28	244/18800	0.306530326	0.38294825	0.321759682	CPSF3	1
GO:0045926	negative regulation of growth	1/28	245/18800	0.307576734	0.383182188	0.32195624	FXN	1
GO:0060047	heart contraction	1/28	247/18800	0.309664985	0.383640509	0.322341329	ATP2A2	1
GO:0090068	positive regulation of cell cycle process	1/28	247/18800	0.309664985	0.383640509	0.322341329	CPSF3	1
GO:0090257	regulation of muscle system process	1/28	250/18800	0.312785978	0.386433646	0.324688171	ATP2A2	1
GO:0009165	nucleotide biosynthetic process	1/28	255/18800	0.317957421	0.388901638	0.32676182	MTHFD1	1
GO:0044843	cell cycle G1/S phase transition	1/28	255/18800	0.317957421	0.388901638	0.32676182	CPSF3	1
GO:0045927	positive regulation of growth	1/28	256/18800	0.318987197	0.388901638	0.32676182	FXN	1
GO:0003015	heart process	1/28	257/18800	0.320015473	0.388901638	0.32676182	ATP2A2	1
GO:0034976	response to endoplasmic reticulum stress	1/28	257/18800	0.320015473	0.388901638	0.32676182	ATP2A2	1
GO:1901293	nucleoside phosphate biosynthetic process	1/28	257/18800	0.320015473	0.388901638	0.32676182	MTHFD1	1
GO:0043543	protein acylation	1/28	258/18800	0.321042252	0.389089251	0.326919456	NFYA	1
GO:0090150	establishment of protein localization to membrane	1/28	259/18800	0.322067536	0.389274041	0.32707472	SRP54	1
GO:1903522	regulation of blood circulation	1/28	260/18800	0.323091327	0.389456032	0.327227632	ATP2A2	1
GO:0031330	negative regulation of cellular catabolic process	1/28	265/18800	0.32818796	0.394533235	0.331493584	MAGEA6	1
GO:0006338	chromatin remodeling	1/28	266/18800	0.329202836	0.394689422	0.331624815	INO80E	1
GO:0051258	protein polymerization	1/28	277/18800	0.340269356	0.406863626	0.34185379	MSRB1	1
GO:0006260	DNA replication	1/28	280/18800	0.343256811	0.409338336	0.343933083	INO80E	1
GO:0006520	cellular amino acid metabolic process	1/28	285/18800	0.348206936	0.414134116	0.34796258	MTHFD1	1
GO:0044262	cellular carbohydrate metabolic process	1/28	287/18800	0.350176895	0.414267627	0.348074758	UGP2	1
GO:0098813	nuclear chromosome segregation	1/28	287/18800	0.350176895	0.414267627	0.348074758	STAG2	1
GO:0051607	defense response to virus	1/28	290/18800	0.353121073	0.416645499	0.350072687	ATG16L1	1
GO:0140546	defense response to symbiont	1/28	291/18800	0.354099604	0.416697687	0.350116537	ATG16L1	1
GO:0034504	protein localization to nucleus	1/28	294/18800	0.357026641	0.416842622	0.350238314	IPO8	1
GO:0048193	Golgi vesicle transport	1/28	294/18800	0.357026641	0.416842622	0.350238314	TRAPPC2	1
GO:0048562	embryonic organ morphogenesis	1/28	294/18800	0.357026641	0.416842622	0.350238314	MTHFD1	1
GO:0048511	rhythmic process	1/28	297/18800	0.359940884	0.419147871	0.352175223	NFYA	1
GO:0019932	second-messenger-mediated signaling	1/28	303/18800	0.365731209	0.42478156	0.356908745	ATP2A2	1
GO:0032409	regulation of transporter activity	1/28	305/18800	0.367650067	0.425901117	0.357849416	SYNGR3	1
GO:0006913	nucleocytoplasmic transport	1/28	308/18800	0.370517863	0.427005082	0.358776987	IPO8	1
GO:0051169	nuclear transport	1/28	308/18800	0.370517863	0.427005082	0.358776987	IPO8	1
GO:1901214	regulation of neuron death	1/28	313/18800	0.375269675	0.429072603	0.360514153	MYBL2	1
GO:0051604	protein maturation	1/28	314/18800	0.376215877	0.429072603	0.360514153	FXN	1
GO:0070588	calcium ion transmembrane transport	1/28	314/18800	0.376215877	0.429072603	0.360514153	ATP2A2	1
GO:0018105	peptidyl-serine phosphorylation	1/28	315/18800	0.377160698	0.429072603	0.360514153	VRK1	1
GO:0060326	cell chemotaxis	1/28	315/18800	0.377160698	0.429072603	0.360514153	SRP54	1
GO:0006605	protein targeting	1/28	317/18800	0.379046201	0.429072603	0.360514153	SRP54	1
GO:0019058	viral life cycle	1/28	317/18800	0.379046201	0.429072603	0.360514153	ATG16L1	1
GO:1901990	regulation of mitotic cell cycle phase transition	1/28	321/18800	0.382800709	0.432225611	0.363163364	CPSF3	1
GO:0006310	DNA recombination	1/28	324/18800	0.385602215	0.433195435	0.363978227	INO80E	1
GO:0009895	negative regulation of catabolic process	1/28	324/18800	0.385602215	0.433195435	0.363978227	MAGEA6	1
GO:0060562	epithelial tube morphogenesis	1/28	328/18800	0.389318487	0.435122241	0.365597163	MTHFD1	1
GO:0045787	positive regulation of cell cycle	1/28	329/18800	0.390244163	0.435122241	0.365597163	CPSF3	1
GO:0048638	regulation of developmental growth	1/28	329/18800	0.390244163	0.435122241	0.365597163	FXN	1
GO:0018209	peptidyl-serine modification	1/28	338/18800	0.398514582	0.44323567	0.372414205	VRK1	1
GO:0007059	chromosome segregation	1/28	348/18800	0.40757707	0.452060335	0.37982884	STAG2	1
GO:0006936	muscle contraction	1/28	349/18800	0.408476043	0.452060335	0.37982884	ATP2A2	1
GO:0010038	response to metal ion	1/28	351/18800	0.410270043	0.45292188	0.380552724	FXN	1
GO:0070997	neuron death	1/28	353/18800	0.412058797	0.453773391	0.381268178	MYBL2	1
GO:0008544	epidermis development	1/28	355/18800	0.413842318	0.454473036	0.381856032	ATP2A2	1
GO:0010639	negative regulation of organelle organization	1/28	356/18800	0.414732121	0.454473036	0.381856032	FXN	1
GO:0006066	alcohol metabolic process	1/28	361/18800	0.419161633	0.458201197	0.384988497	IPMK	1
GO:0051098	regulation of binding	1/28	366/18800	0.423558811	0.461875867	0.388076017	ATP2A2	1
GO:2001233	regulation of apoptotic signaling pathway	1/28	370/18800	0.427053424	0.464550797	0.39032354	FXN	1
GO:0050900	leukocyte migration	1/28	384/18800	0.439124409	0.476519431	0.400379791	SRP54	1
GO:0042692	muscle cell differentiation	1/28	387/18800	0.441678954	0.478128188	0.401731496	ATP2A2	1
GO:0030099	myeloid cell differentiation	1/28	391/18800	0.445067567	0.478723783	0.402231926	SRP54	1
GO:0009615	response to virus	1/28	392/18800	0.445911617	0.478723783	0.402231926	ATG16L1	1
GO:0018205	peptidyl-lysine modification	1/28	392/18800	0.445911617	0.478723783	0.402231926	NFYA	1
GO:0032970	regulation of actin filament-based process	1/28	393/18800	0.446754428	0.478723783	0.402231926	ATP2A2	1
GO:0006163	purine nucleotide metabolic process	1/28	394/18800	0.447596004	0.478723783	0.402231926	MTHFD1	1
GO:0001558	regulation of cell growth	1/28	415/18800	0.464986579	0.494636719	0.415602247	FXN	1
GO:1901987	regulation of cell cycle phase transition	1/28	415/18800	0.464986579	0.494636719	0.415602247	CPSF3	1
GO:0072521	purine-containing compound metabolic process	1/28	416/18800	0.465801394	0.494636719	0.415602247	MTHFD1	1
GO:0016032	viral process	1/28	418/18800	0.467427436	0.49518441	0.416062426	ATG16L1	1
GO:0006816	calcium ion transport	1/28	424/18800	0.472276976	0.497627742	0.418115355	ATP2A2	1
GO:0034470	ncRNA processing	1/28	424/18800	0.472276976	0.497627742	0.418115355	WDR4	1
GO:0042391	regulation of membrane potential	1/28	425/18800	0.473081082	0.497627742	0.418115355	ATP2A2	1
GO:0050804	modulation of chemical synaptic transmission	1/28	429/18800	0.476285705	0.4988081	0.419107112	ATP2A2	1
GO:0099177	regulation of trans-synaptic signaling	1/28	430/18800	0.47708392	0.4988081	0.419107112	ATP2A2	1
GO:0048732	gland development	1/28	431/18800	0.477880961	0.4988081	0.419107112	SRP54	1
GO:0050911	detection of chemical stimulus involved in sensory perception of smell	1/28	432/18800	0.478676831	0.4988081	0.419107112	OR5M9	1
GO:0044772	mitotic cell cycle phase transition	1/28	440/18800	0.485001813	0.504220999	0.423655123	CPSF3	1
GO:0003012	muscle system process	1/28	449/18800	0.49202897	0.507225273	0.426179366	ATP2A2	1
GO:0031331	positive regulation of cellular catabolic process	1/28	449/18800	0.49202897	0.507225273	0.426179366	ATG16L1	1
GO:0048568	embryonic organ development	1/28	449/18800	0.49202897	0.507225273	0.426179366	MTHFD1	1
GO:0007015	actin filament organization	1/28	450/18800	0.492804034	0.507225273	0.426179366	MSRB1	1
GO:0023061	signal release	1/28	451/18800	0.493577957	0.507225273	0.426179366	ATP2A2	1

GO:0006874	cellular calcium ion homeostasis	1/28	456/18800	0.497430522	0.510009225	0.428518489	ATP2A2	1
GO:0007608	sensory perception of smell	1/28	458/18800	0.498963621	0.510407741	0.42885333	OR5M9	1
GO:0055074	calcium ion homeostasis	1/28	468/18800	0.506561721	0.516994342	0.434387505	ATP2A2	1
GO:0051052	regulation of DNA metabolic process	1/28	472/18800	0.509569745	0.518876955	0.435969308	INO80E	1
GO:0007346	regulation of mitotic cell cycle	1/28	478/18800	0.514048652	0.521058406	0.437802201	CPSF3	1
GO:0050907	detection of chemical stimulus involved in sensory perception	1/28	478/18800	0.514048652	0.521058406	0.437802201	OR5M9	1
GO:0016049	cell growth	1/28	484/18800	0.518488102	0.524366652	0.440581846	FXN	1
GO:0009117	nucleotide metabolic process	1/28	487/18800	0.520693136	0.525405291	0.441454528	MTHFD1	1
GO:0043410	positive regulation of MAPK cascade	1/28	491/18800	0.523618039	0.527163985	0.442932213	KLB	1
GO:0072503	cellular divalent inorganic cation homeostasis	1/28	494/18800	0.525800414	0.527708931	0.443390086	ATP2A2	1
GO:0006753	nucleoside phosphate metabolic process	1/28	495/18800	0.526525727	0.527708931	0.443390086	MTHFD1	1
GO:0006397	mRNA processing	1/28	500/18800	0.530136277	0.530136277	0.445429584	CPSF3	1

Table S3. Molecular Function (MF) terms enriched by the 35 candidate genes.

ID	Description	GeneRatio	BgRatio	pvalue	p.adjust	qvalue	geneID	Count
GO:0052813	phosphatidylinositol bisphosphate kinase activity	1/32	10/18410	0.017250712	0.163398776	0.123624074	IPMK	1
GO:0005536	glucose binding	1/32	11/18410	0.018959841	0.163398776	0.123624074	UGP2	1
GO:0008199	ferric iron binding	1/32	11/18410	0.018959841	0.163398776	0.123624074	FXN	1
GO:0044325	transmembrane transporter binding	2/32	127/18410	0.020460426	0.163398776	0.123624074	TRAPPC2/ATP2A2	2
GO:0035004	phosphatidylinositol 3-kinase activity	1/32	12/18410	0.020666091	0.163398776	0.123624074	IPMK	1
GO:0016423	tRNA (guanine) methyltransferase activity	1/32	13/18410	0.022369465	0.163398776	0.123624074	WDR4	1
GO:0031386	protein tag	1/32	13/18410	0.022369465	0.163398776	0.123624074	UBA52	1
GO:0008242	omega peptidase activity	1/32	14/18410	0.02406997	0.163398776	0.123624074	GGT6	1
GO:0019992	diacylglycerol binding	1/32	14/18410	0.02406997	0.163398776	0.123624074	CDIPT	1
GO:0044548	S100 protein binding	1/32	14/18410	0.02406997	0.163398776	0.123624074	ATP2A2	1
GO:0035173	histone kinase activity	1/32	16/18410	0.027462386	0.163398776	0.123624074	VRK1	1
GO:0016722	oxidoreductase activity, acting on metal ions	1/32	17/18410	0.029154307	0.163398776	0.123624074	FXN	1
GO:0016755	aminoacyltransferase activity	1/32	17/18410	0.029154307	0.163398776	0.123624074	GGT6	1
GO:0008409	5'-3' exonuclease activity	1/32	18/18410	0.030843377	0.163398776	0.123624074	CPSF3	1
GO:0015399	primary active transmembrane transporter activity	2/32	162/18410	0.032121051	0.163398776	0.123624074	COX5A/ATP2A2	2
GO:0004129	cytochrome-c oxidase activity	1/32	19/18410	0.0325296	0.163398776	0.123624074	COX5A	1
GO:0016646	oxidoreductase activity, acting on the CH-NH group of donors, NAD or NADP as acceptor	1/32	19/18410	0.0325296	0.163398776	0.123624074	MTHFD1	1
GO:0015662	P-type ion transporter activity	1/32	20/18410	0.034212981	0.163398776	0.123624074	ATP2A2	1
GO:0016675	oxidoreductase activity, acting on a heme group of donors	1/32	20/18410	0.034212981	0.163398776	0.123624074	COX5A	1
GO:0140358	P-type transmembrane transporter activity	1/32	20/18410	0.034212981	0.163398776	0.123624074	ATP2A2	1
GO:0016780	phosphotransferase activity, for other substituted phosphate groups	1/32	22/18410	0.037571234	0.163398776	0.123624074	CDIPT	1
GO:0017134	fibroblast growth factor binding	1/32	23/18410	0.039246115	0.163398776	0.123624074	KLB	1
GO:0051537	2 iron, 2 sulfur cluster binding	1/32	24/18410	0.040918173	0.163398776	0.123624074	FXN	1
GO:0070003	threonine-type peptidase activity	1/32	24/18410	0.040918173	0.163398776	0.123624074	GGT6	1
GO:0005104	fibroblast growth factor receptor binding	1/32	25/18410	0.042587411	0.163398776	0.123624074	KLB	1
GO:0008198	ferrous iron binding	1/32	26/18410	0.044253835	0.163398776	0.123624074	FXN	1
GO:0016645	oxidoreductase activity, acting on the CH-NH group of donors	1/32	29/18410	0.049236265	0.175062276	0.132448432	MTHFD1	1
GO:0008175	tRNA methyltransferase activity	1/32	33/18410	0.055840378	0.191452726	0.144849102	WDR4	1
GO:0016814	hydrolase activity, acting on carbon-nitrogen (but not peptide) bonds, in cyclic amidines	1/32	36/18410	0.060764278	0.201150713	0.152186395	MTHFD1	1
GO:0016896	exoribonuclease activity, producing 5'-phosphomonoesters	1/32	38/18410	0.064033048	0.204905752	0.155027378	CPSF3	1
GO:0004532	exoribonuclease activity	1/32	40/18410	0.067290795	0.206746642	0.156420157	CPSF3	1
GO:0042169	SH2 domain binding	1/32	41/18410	0.068915547	0.206746642	0.156420157	SYNGR3	1
GO:0022853	active ion transmembrane transporter activity	2/32	254/18410	0.071757643	0.208301279	0.157596362	COX5A/ATP2A2	2
GO:0019829	ATPase-coupled cation transmembrane transporter activity	1/32	44/18410	0.07377337	0.208301279	0.157596362	ATP2A2	1
GO:0030246	carbohydrate binding	2/32	270/18410	0.07974001	0.213899431	0.161831806	CDIPT/UGP2	2
GO:0016879	ligase activity, forming carbon-nitrogen bonds	1/32	48/18410	0.080212287	0.213899431	0.161831806	MTHFD1	1
GO:0005048	signal sequence binding	1/32	50/18410	0.083415463	0.216429309	0.163745859	SRP54	1
GO:0051020	GTPase binding	2/32	298/18410	0.094339993	0.232718031	0.176069563	ATG16L1/IPO8	2
GO:0016667	oxidoreductase activity, acting on a sulfur group of donors	1/32	57/18410	0.0945417	0.232718031	0.176069563	MSRB1	1
GO:0016796	exonuclease activity, active with either ribo- or deoxyribonucleic acids and producing 5'-phosphomor	1/32	59/18410	0.097696519	0.234091097	0.177108396	CPSF3	1
GO:0008173	RNA methyltransferase activity	1/32	65/18410	0.107097215	0.234091097	0.177108396	WDR4	1
GO:0030145	manganese ion binding	1/32	65/18410	0.107097215	0.234091097	0.177108396	CDIPT	1
GO:0051536	iron-sulfur cluster binding	1/32	67/18410	0.110209643	0.234091097	0.177108396	FXN	1
GO:0051540	metal cluster binding	1/32	67/18410	0.110209643	0.234091097	0.177108396	FXN	1
GO:0004521	endoribonuclease activity	1/32	71/18410	0.116403	0.234091097	0.177108396	CPSF3	1
GO:0048029	monosaccharide binding	1/32	71/18410	0.116403	0.234091097	0.177108396	UGP2	1
GO:0015453	oxidoreduction-driven active transmembrane transporter activity	1/32	72/18410	0.117944802	0.234091097	0.177108396	COX5A	1
GO:0019003	GDP binding	1/32	73/18410	0.119483997	0.234091097	0.177108396	SRP54	1
GO:0140104	molecular carrier activity	1/32	73/18410	0.119483997	0.234091097	0.177108396	FXN	1
GO:0004527	exonuclease activity	1/32	80/18410	0.130185744	0.249956629	0.189111923	CPSF3	1
GO:0043178	alcohol binding	1/32	89/18410	0.143760116	0.263802663	0.199587541	CDIPT	1
GO:0042626	ATPase-coupled transmembrane transporter activity	1/32	90/18410	0.145255649	0.263802663	0.199587541	ATP2A2	1
GO:0140098	catalytic activity, acting on RNA	2/32	388/18410	0.145641054	0.263802663	0.199587541	CPSF3/WDR4	2
GO:0004553	hydrolase activity, hydrolyzing O-glycosyl compounds	1/32	94/18410	0.15121252	0.268822257	0.203385261	KLB	1
GO:0022804	active transmembrane transporter activity	2/32	404/18410	0.155305736	0.271079103	0.205092743	COX5A/ATP2A2	2
GO:0047485	protein N-terminus binding	1/32	105/18410	0.167387288	0.286949637	0.217100054	SYNGR3	1
GO:0004540	ribonuclease activity	1/32	117/18410	0.184691921	0.296161841	0.224069814	CPSF3	1
GO:0005549	odorant binding	1/32	119/18410	0.187541946	0.296161841	0.224069814	OR5M9	1
GO:0015078	proton transmembrane transporter activity	1/32	120/18410	0.188963337	0.296161841	0.224069814	COX5A	1
GO:0004519	endonuclease activity	1/32	124/18410	0.194624848	0.296161841	0.224069814	CPSF3	1
GO:0009055	electron transfer activity	1/32	125/18410	0.196034232	0.296161841	0.224069814	COX5A	1
GO:0140101	catalytic activity, acting on a tRNA	1/32	125/18410	0.196034232	0.296161841	0.224069814	WDR4	1
GO:0016810	hydrolase activity, acting on carbon-nitrogen (but not peptide) bonds	1/32	126/18410	0.197441227	0.296161841	0.224069814	MTHFD1	1
GO:0042826	histone deacetylase binding	1/32	126/18410	0.197441227	0.296161841	0.224069814	MAGEA6	1
GO:0016779	nucleotidyltransferase activity	1/32	132/18410	0.205833238	0.30399986	0.229999894	UGP2	1
GO:0015085	calcium ion transmembrane transporter activity	1/32	135/18410	0.209997303	0.304258587	0.230195641	ATP2A2	1
GO:0019838	growth factor binding	1/32	139/18410	0.215516499	0.304258587	0.230195641	KLB	1
GO:0070851	growth factor receptor binding	1/32	139/18410	0.215516499	0.304258587	0.230195641	KLB	1
GO:0016798	hydrolase activity, acting on glycosyl bonds	1/32	144/18410	0.222362983	0.309374585	0.234066298	KLB	1
GO:0043021	ribonucleoprotein complex binding	1/32	150/18410	0.230502377	0.313488533	0.237178825	SRP54	1
GO:0005506	iron ion binding	1/32	151/18410	0.231850895	0.313488533	0.237178825	FXN	1
GO:0008757	S-adenosylmethionine-dependent methyltransferase activity	1/32	157/18410	0.23989405	0.319858733	0.241998383	WDR4	1
GO:0016874	ligase activity	1/32	165/18410	0.250491441	0.329413402	0.249227245	MTHFD1	1
GO:0003735	structural constituent of ribosome	1/32	181/18410	0.271258515	0.351902938	0.266242354	UBA52	1
GO:0004518	nuclease activity	1/32	204/18410	0.300137477	0.38417597	0.290659451	CPSF3	1
GO:0008168	methyltransferase activity	1/32	214/18410	0.312344849	0.394540861	0.29850131	WDR4	1
GO:0016741	transferase activity, transferring one-carbon groups	1/32	225/18410	0.325534712	0.405861459	0.307066236	WDR4	1
GO:0016746	acyltransferase activity	1/32	244/18410	0.347742109	0.427990287	0.323808441	GGT6	1
GO:0031267	small GTPase binding	1/32	267/18410	0.373678103	0.454089847	0.343554818	IPO8	1
GO:0031625	ubiquitin protein ligase binding	1/32	298/18410	0.407059471	0.488471365	0.369567151	UBA52	1
GO:0044389	ubiquitin-like protein ligase binding	1/32	317/18410	0.426660068	0.505419958	0.3823901	UBA52	1
GO:0042277	peptide binding	1/32	322/18410	0.431712881	0.505419958	0.3823901	SRP54	1
GO:0003924	GTPase activity	1/32	336/18410	0.445632333	0.515430169	0.389963614	SRP54	1
GO:0106310	protein serine kinase activity	1/32	360/18410	0.468729462	0.535690814	0.405292392	VRK1	1
GO:0005525	GTP binding	1/32	379/18410	0.486350854	0.549290376	0.415581534	SRP54	1
GO:0019001	guanyl nucleotide binding	1/32	401/18410	0.506047634	0.553009454	0.418395311	SRP54	1
GO:0032561	guanyl ribonucleotide binding	1/32	401/18410	0.506047634	0.553009454	0.418395311	SRP54	1
GO:0033218	amide binding	1/32	402/18410	0.506925333	0.553009454	0.418395311	SRP54	1
GO:0046873	metal ion transmembrane transporter activity	1/32	428/18410	0.529222458	0.560062593	0.423731567	ATP2A2	1
GO:0004674	protein serine/threonine kinase activity	1/32	430/18410	0.530896565	0.560062593	0.423731567	VRK1	1
GO:0004984	olfactory receptor activity	1/32	430/18410	0.530896565	0.560062593	0.423731567	OR5M9	1
GO:0003779	actin binding	1/32	439/18410	0.538358946	0.560062593	0.423731567	MSRB1	1
GO:0004712	protein serine/threonine/tyrosine kinase activity	1/32	446/18410	0.544083387	0.560062593	0.423731567	VRK1	1
GO:0019787	ubiquitin-like protein transferase activity	1/32	458/18410	0.553737109	0.560062593	0.423731567	ATG16L1	1

GO:0001228	DNA-binding transcription activator activity, RNA polymerase II-specific	1/32	462/18410	0.556910786	0.560062593	0.423731567	MYBL2	1
GO:0001216	DNA-binding transcription activator activity	1/32	466/18410	0.560062593	0.560062593	0.423731567	MYBL2	1

Table S3. Cellular Component (CC) terms enriched by the 35 candidate genes.

ID	Description	GeneRatio	BgRatio	pvalue	p.adjust	qvalue	geneID	Count
GO:0097470	ribbon synapse	1/34	10/19594	0.017221305	0.239127528	0.205099634	ATP2A2	1
GO:0031094	platelet dense tubular network	1/34	11/19594	0.018927518	0.239127528	0.205099634	ATP2A2	1
GO:1990062	RPAP3/R2TP/prefoldin-like complex	1/34	12/19594	0.020630856	0.239127528	0.205099634	RPAP3	1
GO:0008278	cohesin complex	1/34	14/19594	0.024028925	0.239127528	0.205099634	STAG2	1
GO:0005847	mRNA cleavage and polyadenylation specificity factor complex	1/34	15/19594	0.025723665	0.239127528	0.205099634	CPSF3	1
GO:0031011	Ino80 complex	1/34	15/19594	0.025723665	0.239127528	0.205099634	INO80E	1
GO:0005786	signal recognition particle, endoplasmic reticulum targeting	1/34	16/19594	0.027415549	0.239127528	0.205099634	SRP54	1
GO:0048500	signal recognition particle	1/34	16/19594	0.027415549	0.239127528	0.205099634	SRP54	1
GO:0030008	TRAPP complex	1/34	17/19594	0.029104581	0.239127528	0.205099634	TRAPPC2	1
GO:0005849	mRNA cleavage factor complex	1/34	19/19594	0.032474109	0.239127528	0.205099634	CPSF3	1
GO:0036020	endolysosome membrane	1/34	19/19594	0.032474109	0.239127528	0.205099634	ATG16L1	1
GO:0005751	mitochondrial respiratory chain complex IV	1/34	25/19594	0.042514732	0.256484044	0.219986314	COX5A	1
GO:0097346	INO80-type complex	1/34	26/19594	0.044178307	0.256484044	0.219986314	INO80E	1
GO:0036019	endolysosome	1/34	28/19594	0.047497045	0.256484044	0.219986314	ATG16L1	1
GO:0045277	respiratory chain complex IV	1/34	28/19594	0.047497045	0.256484044	0.219986314	COX5A	1
GO:0000228	nuclear chromosome	2/34	228/19594	0.059291566	0.277843742	0.238306523	INO80E/STAG2	2
GO:0097431	mitotic spindle pole	1/34	36/19594	0.060660509	0.277843742	0.238306523	STAG2	1
GO:0033017	sarcoplasmic reticulum membrane	1/34	41/19594	0.068797828	0.277843742	0.238306523	ATP2A2	1
GO:0044232	organelle membrane contact site	1/34	41/19594	0.068797828	0.277843742	0.238306523	ATG16L1	1
GO:0070069	cytochrome complex	1/34	42/19594	0.070417062	0.277843742	0.238306523	COX5A	1
GO:0022627	cytosolic small ribosomal subunit	1/34	43/19594	0.072033563	0.277843742	0.238306523	UBA52	1
GO:0000421	autophagosome membrane	1/34	51/19594	0.084867716	0.312467502	0.268003315	ATG16L1	1
GO:0022625	cytosolic large ribosomal subunit	1/34	58/19594	0.095956142	0.324370587	0.27821259	UBA52	1
GO:0099023	vesicle tethering complex	1/34	61/19594	0.100668304	0.324370587	0.27821259	TRAPPC2	1
GO:0031594	neuromuscular junction	1/34	67/19594	0.110021216	0.324370587	0.27821259	SYNGR3	1
GO:0015935	small ribosomal subunit	1/34	69/19594	0.113117822	0.324370587	0.27821259	UBA52	1
GO:0016529	sarcoplasmic reticulum	1/34	71/19594	0.116203969	0.324370587	0.27821259	ATP2A2	1
GO:0000794	condensed nuclear chromosome	1/34	74/19594	0.120813654	0.324370587	0.27821259	STAG2	1
GO:0016528	sarcoplasm	1/34	78/19594	0.126923616	0.324370587	0.27821259	ATP2A2	1
GO:0070603	SWI/SNF superfamily-type complex	1/34	86/19594	0.139020145	0.324370587	0.27821259	INO80E	1
GO:1904949	ATPase complex	1/34	88/19594	0.142018767	0.324370587	0.27821259	INO80E	1
GO:0098803	respiratory chain complex	1/34	91/19594	0.146497701	0.324370587	0.27821259	COX5A	1
GO:0034708	methyltransferase complex	1/34	92/19594	0.14798563	0.324370587	0.27821259	WDR4	1
GO:0005746	mitochondrial respirasome	1/34	94/19594	0.150953939	0.324370587	0.27821259	COX5A	1
GO:0005819	spindle	2/34	402/19594	0.153747302	0.324370587	0.27821259	STAG2/VRK1	2
GO:0005765	lysosomal membrane	2/34	404/19594	0.154959503	0.324370587	0.27821259	ATG16L1/UBA52	2
GO:0098852	lytic vacuole membrane	2/34	404/19594	0.154959503	0.324370587	0.27821259	ATG16L1/UBA52	2
GO:0070469	respirasome	1/34	101/19594	0.161264185	0.324370587	0.27821259	COX5A	1
GO:0022626	cytosolic ribosome	1/34	102/19594	0.162727122	0.324370587	0.27821259	UBA52	1
GO:0030672	synaptic vesicle membrane	1/34	103/19594	0.164187581	0.324370587	0.27821259	SYNGR3	1
GO:0099501	exocytic vesicle membrane	1/34	103/19594	0.164187581	0.324370587	0.27821259	SYNGR3	1
GO:0016363	nuclear matrix	1/34	109/19594	0.172898554	0.326247625	0.279822525	STAG2	1
GO:0005776	autophagosome	1/34	112/19594	0.177220932	0.326247625	0.279822525	ATG16L1	1
GO:0015934	large ribosomal subunit	1/34	112/19594	0.177220932	0.326247625	0.279822525	UBA52	1
GO:0005774	vacuolar membrane	2/34	449/19594	0.182703588	0.328866458	0.282068697	ATG16L1/UBA52	2
GO:0005759	mitochondrial matrix	2/34	473/19594	0.197806905	0.332880187	0.285511271	LYRM4/FXN	2
GO:0034399	nuclear periphery	1/34	127/19594	0.198506061	0.332880187	0.285511271	STAG2	1
GO:0005793	endoplasmic reticulum-Golgi intermediate compartment	1/34	131/19594	0.20409122	0.332880187	0.285511271	TRAPPC2	1
GO:0005930	axoneme	1/34	131/19594	0.20409122	0.332880187	0.285511271	ATG16L1	1
GO:0097014	ciliary plasm	1/34	132/19594	0.205481597	0.332880187	0.285511271	ATG16L1	1
GO:0005795	Golgi stack	1/34	153/19594	0.234140837	0.368888575	0.316395659	VRK1	1
GO:0098800	inner mitochondrial membrane protein complex	1/34	155/19594	0.236817357	0.368888575	0.316395659	COX5A	1
GO:0072686	mitotic spindle	1/34	160/19594	0.243468998	0.372094129	0.319145062	STAG2	1
GO:0000922	spindle pole	1/34	169/19594	0.25530043	0.382950645	0.328456693	STAG2	1
GO:0044391	ribosomal subunit	1/34	178/19594	0.266952189	0.393147769	0.337202764	UBA52	1
GO:0030666	endocytic vesicle membrane	1/34	194/19594	0.287230997	0.399728961	0.342847452	UBA52	1
GO:0008021	synaptic vesicle	1/34	196/19594	0.289727237	0.399728961	0.342847452	SYNGR3	1
GO:0044815	DNA packaging complex	1/34	198/19594	0.292214992	0.399728961	0.342847452	STAG2	1
GO:0005741	mitochondrial outer membrane	1/34	205/19594	0.300855708	0.399728961	0.342847452	UBA52	1
GO:0030658	transport vesicle membrane	1/34	205/19594	0.300855708	0.399728961	0.342847452	SYNGR3	1
GO:0070382	exocytic vesicle	1/34	214/19594	0.31181487	0.399728961	0.342847452	SYNGR3	1
GO:0032838	plasma membrane bounded cell projection cytoplasm	1/34	220/19594	0.319028153	0.399728961	0.342847452	ATG16L1	1
GO:0032993	protein-DNA complex	1/34	220/19594	0.319028153	0.399728961	0.342847452	NFYA	1
GO:0000775	chromosome, centromeric region	1/34	227/19594	0.327350923	0.399728961	0.342847452	STAG2	1
GO:0005840	ribosome	1/34	229/19594	0.329710668	0.399728961	0.342847452	UBA52	1
GO:0090575	RNA polymerase II transcription regulator complex	1/34	230/19594	0.330887525	0.399728961	0.342847452	NFYA	1
GO:0031968	organelle outer membrane	1/34	232/19594	0.333235225	0.399728961	0.342847452	UBA52	1
GO:0019867	outer membrane	1/34	234/19594	0.33557493	0.399728961	0.342847452	UBA52	1
GO:0000793	condensed chromosome	1/34	255/19594	0.359665673	0.421381972	0.361419236	STAG2	1
GO:0099568	cytoplasmic region	1/34	259/19594	0.36415726	0.421381972	0.361419236	ATG16L1	1
GO:0098798	mitochondrial protein-containing complex	1/34	281/19594	0.388319656	0.443012565	0.379971791	COX5A	1
GO:0030139	endocytic vesicle	1/34	342/19594	0.450747133	0.507090525	0.434931444	UBA52	1
GO:0098687	chromosomal region	1/34	366/19594	0.473573948	0.525472463	0.450697629	STAG2	1
GO:0098791	Golgi apparatus subcompartment	1/34	385/19594	0.490989559	0.537434518	0.460957481	VRK1	1
GO:0030133	transport vesicle	1/34	402/19594	0.506096949	0.546584705	0.468805595	SYNGR3	1
GO:0016607	nuclear speck	1/34	411/19594	0.513917856	0.547728241	0.469786405	SRP54	1
GO:0005635	nuclear envelope	1/34	479/19594	0.569246512	0.584321379	0.501172333	IPO8	1
GO:0005667	transcription regulator complex	1/34	483/19594	0.572303323	0.584321379	0.501172333	NFYA	1
GO:0005743	mitochondrial inner membrane	1/34	491/19594	0.578353908	0.584321379	0.501172333	COX5A	1
GO:0098793	presynapse	1/34	492/19594	0.579104364	0.584321379	0.501172333	SYNGR3	1
GO:0015629	actin cytoskeleton	1/34	499/19594	0.584321379	0.584321379	0.501172333	MSRB1	1

Table S3. KEGG pathway enrichment analysis of the 35 candidate genes.

ID	Description	GeneRatio	BgRatio	pvalue	p.adjust	qvalue	geneID	Count
hsa00562	Inositol phosphate metabolism	2/18	73/8390	0.010440879	0.270483624	0.240916588	IPMK/CDIPT	2
hsa04260	Cardiac muscle contraction	2/18	87/8390	0.014601808	0.270483624	0.240916588	COX5A/ATP2A2	2
hsa04070	Phosphatidylinositol signaling system	2/18	97/8390	0.017944949	0.270483624	0.240916588	IPMK/CDIPT	2
hsa00430	Taurine and hypotaurine metabolism	1/18	16/8390	0.033809485	0.270483624	0.240916588	GGT6	1
hsa05017	Spinocerebellar ataxia	2/18	143/8390	0.036925623	0.270483624	0.240916588	NFYA/ATP2A2	2
hsa01240	Biosynthesis of cofactors	2/18	153/8390	0.041761278	0.270483624	0.240916588	UGP2/MTHFD1	2
hsa00670	One carbon pool by folate	1/18	20/8390	0.042091563	0.270483624	0.240916588	MTHFD1	1
hsa03060	Protein export	1/18	23/8390	0.048259072	0.270483624	0.240916588	SRP54	1
hsa00052	Galactose metabolism	1/18	31/8390	0.064522929	0.270483624	0.240916588	UGP2	1
hsa04136	Autophagy - other	1/18	32/8390	0.066537355	0.270483624	0.240916588	ATG16L1	1
hsa05415	Diabetic cardiomyopathy	2/18	203/8390	0.069156554	0.270483624	0.240916588	COX5A/ATP2A2	2
hsa00040	Pentose and glucuronate interconversions	1/18	35/8390	0.072556081	0.270483624	0.240916588	UGP2	1
hsa00500	Starch and sucrose metabolism	1/18	36/8390	0.074554165	0.270483624	0.240916588	UGP2	1
hsa01250	Biosynthesis of nucleotide sugars	1/18	37/8390	0.076548183	0.270483624	0.240916588	UGP2	1
hsa05022	Pathways of neurodegeneration - multiple diseases	3/18	476/8390	0.078540144	0.270483624	0.240916588	COX5A/UBA52/ATP2A2	3
hsa04714	Thermogenesis	2/18	232/8390	0.087163566	0.270483624	0.240916588	KLB/COX5A	2
hsa00860	Porphyrin metabolism	1/18	43/8390	0.088427339	0.270483624	0.240916588	FXN	1
hsa05131	Shigellosis	2/18	247/8390	0.096992169	0.274127768	0.244162385	ATG16L1/UBA52	2
hsa00520	Amino sugar and nucleotide sugar metabolism	1/18	49/8390	0.100162069	0.274127768	0.244162385	UGP2	1
hsa05012	Parkinson disease	2/18	266/8390	0.109887986	0.285708763	0.254477441	COX5A/UBA52	2

hsa00480	Glutathione metabolism	1/18	57/8390	0.11558657	0.286214364	0.254927774	GGT6	1
hsa04137	Mitophagy - animal	1/18	72/8390	0.143836824	0.33560907	0.298923058	UBA52	1
hsa05412	Arrhythmogenic right ventricular cardiomyopathy	1/18	77/8390	0.153062635	0.33560907	0.298923058	ATP2A2	1
hsa04612	Antigen processing and presentation	1/18	78/8390	0.154896494	0.33560907	0.298923058	NFYA	1
hsa05410	Hypertrophic cardiomyopathy	1/18	90/8390	0.176612309	0.343412948	0.305873881	ATP2A2	1
hsa05414	Dilated cardiomyopathy	1/18	96/8390	0.187271543	0.343412948	0.305873881	ATP2A2	1
hsa03015	mRNA surveillance pathway	1/18	97/8390	0.189035362	0.343412948	0.305873881	CPSF3	1
hsa00564	Glycerophospholipid metabolism	1/18	99/8390	0.192552159	0.343412948	0.305873881	CDIPT	1
hsa04972	Pancreatic secretion	1/18	102/8390	0.197800359	0.343412948	0.305873881	ATP2A2	1
hsa05010	Alzheimer disease	2/18	384/8390	0.198122855	0.343412948	0.305873881	COX5A/ATP2A2	2
hsa03013	Nucleocytoplasmic transport	1/18	108/8390	0.20820026	0.349239146	0.311063207	IPO8	1
hsa04919	Thyroid hormone signaling pathway	1/18	121/8390	0.230298191	0.374234561	0.333326329	ATP2A2	1
hsa00190	Oxidative phosphorylation	1/18	134/8390	0.251812758	0.384656534	0.342609059	COX5A	1
hsa04140	Autophagy - animal	1/18	141/8390	0.263160996	0.384656534	0.342609059	ATG16L1	1
hsa04120	Ubiquitin mediated proteolysis	1/18	142/8390	0.264768839	0.384656534	0.342609059	UBA52	1
hsa04261	Adrenergic signaling in cardiomyocytes	1/18	154/8390	0.283806306	0.384656534	0.342609059	ATP2A2	1
hsa04932	Non-alcoholic fatty liver disease	1/18	155/8390	0.285371567	0.384656534	0.342609059	COX5A	1
hsa04218	Cellular senescence	1/18	156/8390	0.286933596	0.384656534	0.342609059	MYBL2	1
hsa04110	Cell cycle	1/18	157/8390	0.288492401	0.384656534	0.342609059	STAG2	1
hsa03010	Ribosome	1/18	167/8390	0.303904444	0.385439782	0.343306689	UBA52	1
hsa04022	cGMP-PKG signaling pathway	1/18	167/8390	0.303904444	0.385439782	0.343306689	ATP2A2	1
hsa05152	Tuberculosis	1/18	180/8390	0.323469078	0.400485525	0.356707755	NFYA	1
hsa04621	NOD-like receptor signaling pathway	1/18	186/8390	0.332322677	0.401878586	0.357948538	ATG16L1	1
hsa05167	Kaposi sarcoma-associated herpesvirus infection	1/18	194/8390	0.34395735	0.406495051	0.362060369	UBA52	1
hsa05208	Chemical carcinogenesis - reactive oxygen species	1/18	223/8390	0.384548052	0.43777006	0.389916653	COX5A	1
hsa04024	cAMP signaling pathway	1/18	225/8390	0.38725813	0.43777006	0.389916653	ATP2A2	1
hsa05171	Coronavirus disease - COVID-19	1/18	232/8390	0.396654916	0.438852247	0.390880544	UBA52	1
hsa04020	Calcium signaling pathway	1/18	240/8390	0.407227412	0.44116303	0.392938731	ATP2A2	1
hsa05020	Prion disease	1/18	273/8390	0.449017522	0.476508391	0.424420429	COX5A	1
hsa05016	Huntington disease	1/18	306/8390	0.488014121	0.507534686	0.452055186	COX5A	1
hsa05014	Amyotrophic lateral sclerosis	1/18	364/8390	0.550313544	0.561104006	0.49976875	COX5A	1
hsa04740	Olfactory transduction	1/18	439/8390	0.620301952	0.620301952	0.552495666	OR5M9	1
