# Supplementary material for: A genome-wide CRISPR knockout screen identified host genes essential for Brucella invasion and intracellular survival
Source: Emerg Microbes Infect. 2026 Aug 19;15(1):2713322. doi: 10.1080/22221751.2026.2713322 (PMC13491901; doi:10.1080/22221751.2026.2713322)
Supplement: Supplementary file 4 [file TEMI_A_2713322_SM6418.pdf]

Table S4

qpcr primer

primer	sequence
MTHFD1-qp-f	GGAGCAAGTACCTGGTTTCAC
MTHFD1-qp-r	AGTGGCTTTGATCCCAATCTCT
ZNF532-qp-f	GGTCAAGGTTCACAAGGCAC
ZNF532-qp-r	AACAGATAGCTGAGTGGGCG
WDR4-qp-f	GATTCTGGCGTCCACCTTCT
WDR4-qp-r	CTTGCCACGGTCCTGACAC
βactin-qp-f	CACCATTGGCAATGAGCGGTTC
βactin-qpcr-r	AGGTCTTTGCGGATGTCCACGT
TRAPPC2-qp-f	CTGTGGACAAGTTCAACGAGT
TRAPPC2-qp-r	CGAATAGGAGAATTGGGTTC

sgRNA sequences

sgRNA	sequence
ZNF532-sg1-F	CACCGTTGATAAACAGATAGCTGAG
ZNF532-sg1-R	AAACCTCAGCTATCTGTTTATCAAC
ZNF532-sg2-F	CACCGTGATAAACAGATAGCTGAGT
ZNF532-sg2-R	AAACACTCAGCTATCTGTTTATCAC
MTHFD1-sg1-F	CACCGCAGTTGAAGGAGCAAGTACC
MTHFD1-sg1-R	AAACGGTACTTGCTCCTTCAACTGC
MTHFD1-sg2-F	CACCGCAATGCTATTGCACCCGAGA
MTHFD1-sg2-R	AAACTCTCGGGTGCAATAGCATTGC
WDR4-sg1-F	CACCGAGGACGCGCCCTTGGACCA
WDR4-sg1-R	AAACTGGTCCAAGGGCGCGTCCTC
WDR4-sg2-F	CACCGGAGGACGCGCCCTTGGACC
WDR4-sg2-R	AAACGGTCCAAGGGCGCGTCCTCC
TRAPPC2-sg1-F	CACCGTGTCGGCATTTGTCACTGCG
TRAPPC2-sg1-R	AAACCGCAGTGACAAATGCCGACAC
TRAPPC2-sg2-F	CACCGTGTCGGCATTTGTCACTGC
TRAPPC2-sg2-R	AAACGCAGTGACAAATGCCGACAC
GGT6-sg1-F	CACCGCGCTGGTTCTAAACCCCCGG
GGT6-sg1-R	AAACCCGGGGGTTTAGAACCAGCGC
GGT6-sg2-F	CACCGTCTAAACCCCCGGAGGCACC
GGT6-sg2-R	AAACGGTGCCTCCGGGGGTTTAGAC
OR5M9-sg1-F	CACCGAGTGATCATGTAAACCGCT
OR5M9-sg1-R	AAACAGCGGTTTACATGATCACTC
OR5M9-sg2-F	CACCGCATTGCCGTTGTCCACGTGG
OR5M9-sg2-R	AAACCCACGTGGACAACGGCAATGC
ATGL16L1-sg1-F	CACCGCTGAATTACACAAGAAACGT
ATGL16L1-sg1-R	AAACACGTTTCTTGTGTAATTCAGC
ATGL16L1-sg2-F	CACCGCTTGGTGCTTAATCCTCAGT
ATGL16L1-sg2-R	AAACACTGAGGATTAAGCACCAAGC
UGP2-sg1-F	CACCGATAGCTTCCGAAATCCATCC
UGP2-sg1-R	AAACGGATGGATTTCGGAAGCTATC
UGP2-sg2-F	CACCGAAGGCCCTAAAAGTCTGAT
UGP2-sg2-R	AAACATCAGACTTTTAGGGCCTTTC
NFYA-sg1-F	CACCGCCACTGACCTGCACCATTAA
NFYA-sg1-R	AAACTTAATGGTGCAGGTCAGTGGC
NFYA-sg2-F	CACCGGCCAGTTGATGTGATTAGC
NFYA-sg2-R	AAACGCTAATCACATCAACTGGCC
BOLA1-sg1-F	CACCGCTCCAACTTCGTGCGAATGG
BOLA1-sg1-R	AAACCCATTCGCACGAAGTTGGAGC
BOLA1-sg2-F	CACCGGCCGCCATTCGCACGAAGT
BOLA1-sg2-R	AAACACTTCGTGCGAATGGCGGCC
MSRB1-sg1-F	CACCGTGTGCATACTTCGAGCGGC
MSRB1-sg1-R	AAACGCCGCTCGAAGTATGCACAC
MSRB1-sg2-F	CACCGACCATTCACGCCGACAGCG
MSRB1-sg2-R	AAACCGCTGTCGGCGTGAATGGTC
IPMK-sg1-F	CACCGTCAGGGGTGGACTCGATCGC
IPMK-sg1-R	AAACGCGATCGAGTCCACCCCTGAC
IPMK-sg2-F	CACCGCCCGGCCACCTGATGCGAG
IPMK-sg2-R	AAACCTCGCATCAGGTGGCCGGGC

Sanger sequencing primer

primer	sequence
ZNF532-Sanger-F	CAGATCCTTGCTTTTCAGTGC
ZNF532-Sanger-R	ATGGTCACATGGGACAGGGA
MTHFD1-Sanger-F	GCTCCTGGCCAGTGTTTTTGA
MTHFD1-Sanger-R	TTCCAAGCATCCCTTAGGCG
WDR4-Sanger-F	AGGGCCATGTGTCATTGAGG
WDR4-Sanger-R	GGCTCCGTGTCAACCACTTA
TRAPPC2-Sanger-F	CTGTGGTTGCTCACACTGTC
TRAPPC2-Sanger-R	CACCCTTGATAGGGTCCAGA
GGT6-Sanger-F	GAGACACAGCCGGAAAGG
GGT6-Sanger-R	CGTACAGTGCCTGGAGCTAAA
OR5M9-Sanger-F	TGCCTAATTTCACGGATGTGAC
OR5M9-Sanger-R	AGGCACAGAGATGAGCCGAA
ATGL16L1-Sanger-F	CCCTGTTGGTGCCTGTTACT
ATGL16L1-Sanger-R	AAAGCCTCTCCACTCTTGCC
UGP2-Sanger-F	TCCCCCTTCTCGTACTCCATT
UGP2-Sanger-R	AATAAGGCCACTGCCCCAAA
NFYA-Sanger-F	GGATTTAGCCCGTGTTTGGTG
NFYA-Sanger-R	AAATGTGGGTTAGGAAGCAGTT
BOLA1-Sanger-F	CGTGCGGGCCTTTCTAATCT
BOLA1-Sanger-R	GGGTTCAGGGGGTTCCTAGA
MSRB1-Sanger-F	TGGATTTGGGAGCTTGACCC
MSRB1-Sanger-R	CCACATTTGCACCAGAAGGC
IPMK-Sanger-F	ATGGCAACAGAGCCACCATC
IPMK-Sanger-R	GAATTGTCCCACTTTCCGAGC
